# A high-accuracy consensus map of yeast protein complexes reveals modular nature of gene essentiality

**DOI:** 10.1186/1471-2105-8-236

**Published:** 2007-07-02

**Authors:** G Traver Hart, Insuk Lee, Edward R Marcotte

**Affiliations:** 1 Center for Systems and Synthetic Biology Institute for Cellular and Molecular Biology University of Texas at Austin 2500 Speedway, MBB 3.210 Austin, Texas, 78712, USA

## Abstract

**Background:**

Identifying all protein complexes in an organism is a major goal of systems biology. In the past 18 months, the results of two genome-scale tandem affinity purification-mass spectrometry (TAP-MS) assays in yeast have been published, along with corresponding complex maps. For most complexes, the published data sets were surprisingly uncorrelated. It is therefore useful to consider the raw data from each study and generate an accurate complex map from a high-confidence data set that integrates the results of these and earlier assays.

**Results:**

Using an unsupervised probabilistic scoring scheme, we assigned a confidence score to each interaction in the matrix-model interpretation of the large-scale yeast mass-spectrometry data sets. The scoring metric proved more accurate than the filtering schemes used in the original data sets. We then took a high-confidence subset of these interactions and derived a set of complexes using MCL. The complexes show high correlation with existing annotations. Hierarchical organization of some protein complexes is evident from inter-complex interactions.

**Conclusion:**

We demonstrate that our scoring method can generate an integrated high-confidence subset of observed matrix-model interactions, which we subsequently used to derive an accurate map of yeast complexes. Our results indicate that essentiality is a product of the protein complex rather than the individual protein, and that we have achieved near saturation of the yeast high-abundance, rich-media-expressed "complex-ome."

## Background

The molecular machines that carry out basic cellular processes are typically not individual proteins but protein complexes. Even in the relatively simple model organism *Saccharomyces cerevisiae*, most machines that process and store biological information are in fact large protein complexes comprised of many subunits.

The path from measuring protein interactions to defining complexes has been well studied. Experimental and computational methods have provided over 50,000 putative yeast protein-protein interactions to date, although a substantial fraction of these may be spurious[1,2]. An array of analytical methods aimed at generating high-quality complexes from these data have been applied, including both unsupervised [3-5] and trained [6,7] techniques. Other genomic and proteomic data sets, such as gene expression, knockout phenotype, subcellular localization, and genetic interaction profiles, and phylogenetic profiles [5,6,8-10], have also been integrated with the raw interaction data in an effort to broaden and deepen our ability to accurately define protein complexes.

Two recent genome-scale tandem affinity purification/mass spectrometry (TAP-MS) experiments perfomed by Gavin *et al*. [11] and Krogan *et al*. [12], have produced an enormous amount of new data, allowing a more complete analysis of the universe of yeast protein complexes. However, the complex maps published independently by the two groups show a surprising lack of correlation, which can only be partially explained by the different analytical methods applied after generating the raw data [1,13].

TAP-MS data typically consist of a tagged "bait" protein and the associated "prey" proteins that co-purify with the bait. Interaction data sets are generated from this raw data using either the spoke method, which considers bait-prey interactions, or the matrix method, which includes all prey-prey interactions from a given bait pull-down [14]. As the affinity purification process generally isolates stable complexes, there is no clear-cut way to differentiate between direct physical interactions and indirect interactions mediated by other members of the complex – or, for that matter, other proteins that appear simply a result of experimental noise. Thus, the spoke model contains both direct physical interactions and a sampling of the indirect interactions within a complex, plus some amount of noise, while the matrix model captures a much larger number of true indirect interactions at the price of decreased accuracy from linking every spurious protein to every "real" one, as well as linking proteins from heterogeneous complexes that each contain the bait. While some efforts have been made to use a filtered subset of matrix-model interactions to improve accuracy [9,15,16], analysis of mass spectrometry interaction data has typically been carried out using the spoke model [3,5].

Here we offer a simple yet robust statistical scoring scheme for assigning confidence to observed interactions. The scheme is based on comparing observed versus expected numbers of interactions in the matrix model of protein-protein interactions, and provides greatly increased recall and/or precision over the standard spoke model interpretation. A further advantage of the system is that it can be used to integrate data sets from different sources. We use the scoring scheme to combine the Gavin *et al*. [11], Krogan *et al*. [12], and Ho *et al*. [17] co-complex data sets and define a high-quality subset comprised of 1689 proteins in 390 complexes. We further show that essential proteins strongly cluster together, supporting a complex-centric rather than gene-centric basis for essentiality for a large fraction of essential genes.

## Results

**Figure 1:**
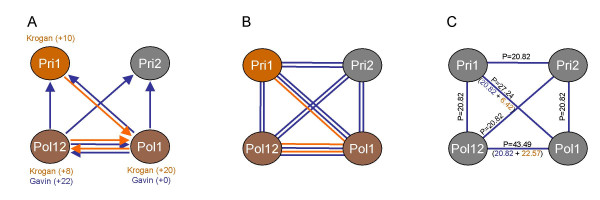
**Applying the matrix-model scoring algorithm**. The four subunits of the DNA primase core complex are detected using the scoring algorithm. (A) In the Gavin *et al*. TAP-MS data set, Pol1 and Pol12 were purified as bait and their corresponding bait-prey, spoke model interactions are shown in blue (plus number of additional prey identified shown in parentheses). In the Krogan *et al*. assay (shown in orange), the same baits plus Pri1 were purified. (B) In the matrix model, both bait-prey and prey-prey interactions are considered. Within a given dataset, the total number of links observed between each pair of proteins is recorded and the P-value calculated as described in the text. The PICO network was generated by multiplying P-values for the same interaction derived from different data sets, e.g. Pol1–Pol12 is discovered in both Gavin and Krogan and scored accordingly. (C) The PICO network integrates probability scores from all data sources, here represented as -ln(P-value). Values in black are final PICO scores; separate scores from Gavin *et al*. (blue) and Krogan *et al*. (orange) are shown where applicable. No data from Ho *et al*. was relevant to this example.

In a large-scale interaction assay, we consider each protein's interactions to be a random sample from the population of observed interactions. A simple and general theoretical error model, based on the hypergeometric distribution, can be used to calculate the probability of observing each interaction from a random background. This model builds on related models that have previously been applied to several linkage and interaction types [18-21]. Within a given dataset, the probability (P-value) of an interaction between proteins A and B being observed at random is:

p(#interactions≥k|n,m,N)=∑i=kmin⁡(n,m)p(i|n,m,N)
 MathType@MTEF@5@5@+=feaafiart1ev1aaatCvAUfKttLearuWrP9MDH5MBPbIqV92AaeXatLxBI9gBaebbnrfifHhDYfgasaacH8akY=wiFfYdH8Gipec8Eeeu0xXdbba9frFj0=OqFfea0dXdd9vqai=hGuQ8kuc9pgc9s8qqaq=dirpe0xb9q8qiLsFr0=vr0=vr0dc8meaabaqaciaacaGaaeqabaqabeGadaaakeaacqWGWbaCcqGGOaakcqGGJaWicqqGPbqAcqqGUbGBcqqG0baDcqqGLbqzcqqGYbGCcqqGHbqycqqGJbWycqqG0baDcqqGPbqAcqqGVbWBcqqGUbGBcqqGZbWCcqGHLjYScqWGRbWAcqGG8baFcqWGUbGBcqGGSaalcqWGTbqBcqGGSaalcqWGobGtcqGGPaqkcqGH9aqpdaaeWbqaaiabdchaWjabcIcaOiabdMgaPjabcYha8jabd6gaUjabcYcaSiabd2gaTjabcYcaSiabd6eaojabcMcaPaWcbaGaemyAaKMaeyypa0Jaem4AaSgabaGagiyBa0MaeiyAaKMaeiOBa4MaeiikaGIaemOBa4MaeiilaWIaemyBa0MaeiykaKcaniabggHiLdaaaa@6788@

where

p(i|n,m,N)=(ni)(N−nm−i)(Nm)
 MathType@MTEF@5@5@+=feaafiart1ev1aaatCvAUfKttLearuWrP9MDH5MBPbIqV92AaeXatLxBI9gBaebbnrfifHhDYfgasaacH8akY=wiFfYdH8Gipec8Eeeu0xXdbba9frFj0=OqFfea0dXdd9vqai=hGuQ8kuc9pgc9s8qqaq=dirpe0xb9q8qiLsFr0=vr0=vr0dc8meaabaqaciaacaGaaeqabaqabeGadaaakeaacqWGWbaCcqGGOaakcqWGPbqAcqGG8baFcqWGUbGBcqGGSaalcqWGTbqBcqGGSaalcqWGobGtcqGGPaqkcqGH9aqpdaWcaaqaamaabmaabaqbaeqabiqaaaqaaiabd6gaUbqaaiabdMgaPbaaaiaawIcacaGLPaaadaqadaqaauaabeqaceaaaeaacqWGobGtcqGHsislcqWGUbGBaeaacqWGTbqBcqGHsislcqWGPbqAaaaacaGLOaGaayzkaaaabaWaaeWaaeaafaqabeGabaaabaGaemOta4eabaGaemyBa0gaaaGaayjkaiaawMcaaaaaaaa@4A91@

where *k *= the number of times the interaction between A and B is observed, *n *and *m *are the total number of interactions for proteins A and B, and *N *is the total number of interactions observed in the entire data set. When applied to the matrix model interpretation of protein interactions, the scoring scheme can identify highly accurate subsets of interactions. The process is illustrated in Figure 1.

**Figure 2:**
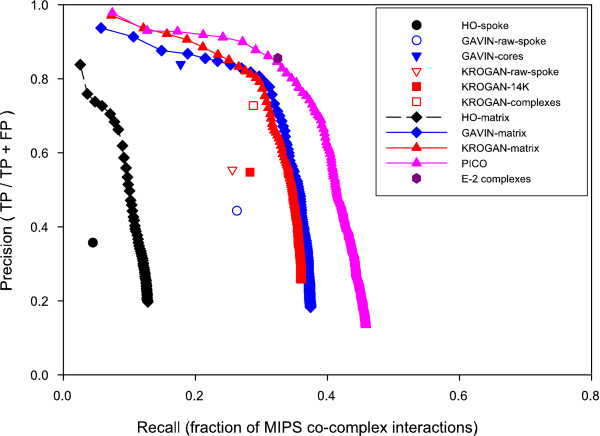
**Performance curves of the probabilistic scoring method**. We measured the performance of the various datasets against a reference set consisting of a matrix-model interaction set generated from MIPS curated complexes, excluding the large and small ribosomal subunits (which would otherwise account for over half of the interactions in this set). Single points represent an entire dataset. Curves represent a dataset that has been scored using the hypergeometric scoring algorithm, rank ordered, and plotted with each symbol representing the cumulative addition of the 500 next highest scoring interactions (i.e. tail of the curve represents the entire dataset). The scoring scheme outperforms the raw data as well as the filtered, published sets in all cases; the integrated PICO net outperforms the individual scored data sets, and the derived complexes are slightly more accurate than PICO (for all thresholds; data not shown).

We generated matrix-model interpretations of the Ho, Gavin, and Krogan datasets. The only other TAP-MS data set of significant scale [22] is a subset of [11] and was omitted. We then applied the scoring method to each, applying to each interaction in a dataset a P-value calculated from the observations within that set. We then evaluated the quality of the scoring by calculating recall and precision versus the set of protein complexes manually defined from literature sources by the Munich Information center on Protein Sequences (MIPS) [23]. Recall was scored as TP/(TP + FN), where TP, true positives, are experimental interactions that are in the MIPS set and FN, false negatives, are the MIPS interactions not present in the experimental data. Precision was defined as TP/(TP + FP), where TP is as above and FP, false positives, are interactions observed experimentally where both corresponding proteins are in the MIPS set, but the interaction is not. For all three data sets, the method displays improved recall and/or precision relative not only to the spoke model interpretation of the same dataset, but also to the group's published complexes (Figure 2). As each co-complex data set represents an independent experimental observation, the probabilities can be combined to provide higher confidence in repeated observations. We therefore combined the three scored data sets by multiplying the P-values for a given interaction across all three datasets, applying a P-value of 1 if the interaction was missing from a dataset. The combined interaction dataset, which we call the Probabilistic Integrated Co-complex (PICO) network, is more accurate and provides greater coverage than any of the individual datasets it comprises.

**Figure 3:**
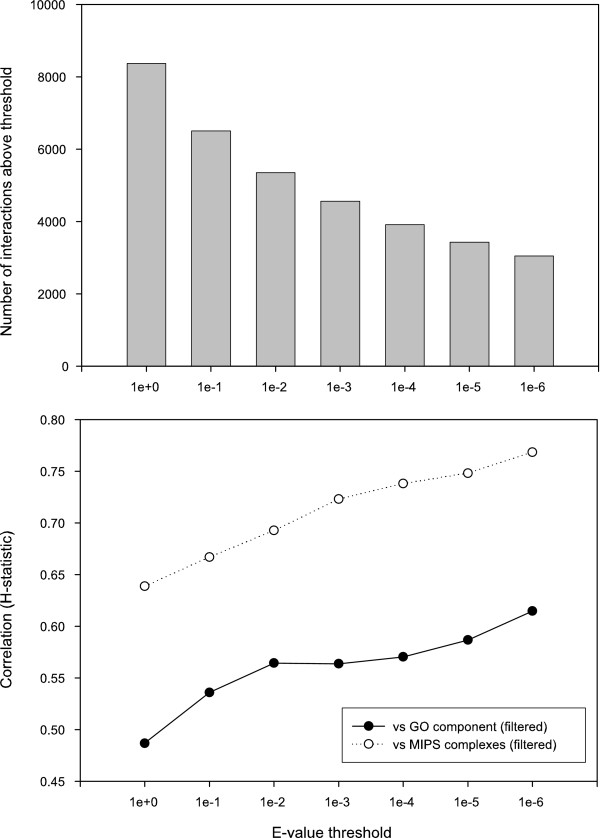
**Effect of thresholds on network size and derived complex accuracy**. (A) Interactions in the PICO network were rank ordered, and the E-value was calculated as the sum of P-values. The number of interactions at each E-value threshold was counted; the total decreases as an increasingly stringent threshold is applied. (B) At each E-value threshold, the subset of interactions was clustered with MCL with parameters that optimized correlation with the filtered set of GO component annotations [see Methods]. The correlation with GO component (filled circles) and MIPS complexes (hollow circles) generally improves with the stringency of the E-value cutoff. We judged that the 10^-2 ^cutoff provides a reasonable tradeoff between increasing accuracy and decreasing coverage, and chose this subset for further study.

The PICO network contains a large number (~160,000) of protein-protein interactions, each with a relative confidence measure as described by the P-value. The full list is available for download [see Additional File 1]. We filtered out low-confidence interactions before deriving complexes from the data, beginning by rank-ordering the interactions by P-value, lowest to highest. We then applied a series of increasingly stringent expected (E) value thresholds, where E=∑i=1nPi
 MathType@MTEF@5@5@+=feaafiart1ev1aaatCvAUfKttLearuWrP9MDH5MBPbIqV92AaeXatLxBI9gBaebbnrfifHhDYfgasaacH8akY=wiFfYdH8Gipec8Eeeu0xXdbba9frFj0=OqFfea0dXdd9vqai=hGuQ8kuc9pgc9s8qqaq=dirpe0xb9q8qiLsFr0=vr0=vr0dc8meaabaqaciaacaGaaeqabaqabeGadaaakeaacqWGfbqrcqGH9aqpdaaeWbqaaiabdcfaqnaaBaaaleaacqWGPbqAaeqaaaqaaiabdMgaPjabg2da9iabigdaXaqaaiabd6gaUbqdcqGHris5aaaa@3862@, starting with E = 1 and tightening in order of magnitude increments to E = 10^-6^. The number of interactions in the PICO network at each threshold is shown in Figure 3A.

We derived a set of complexes at each threshold by using MCL [24], an implementation of a Markov clustering algorithm. MCL was evaluated in [25] and was used to derive complexes from the raw data in [12]. To evaluate the accuracy of each set of complexes, we measured the Hubert statistic, H, of the derived complexes versus a reference set of complexes [26]. Briefly, calculating H involves generating a matrix M of protein pairs (i, j) where M(i, j) = 1 if the proteins are in the same complex and 0 otherwise. The correlation between the experimental and reference matrices is then measured, resulting in a score from -1 to 1, with 1 implying identical complex assignments and values near zero indicating random assignment. We measured the Hubert statistic of complexes measured at each threshold against the set of curated MIPS complexes [23] with ribosomal subunits removed and against a filtered set of Gene Ontology (GO) Cellular Component (CC) annotations (see Methods). The correlations generally improve with increasing stringency (Figure 3B), although the rate of increase in correlation with GO component drops off sharply after the 10^-2 ^cutoff. This improvement in accuracy comes at the price of decreasing coverage, reflected in the decreasing number of interactions at each threshold as shown in Figure 3A. In an attempt to balance accuracy and coverage, we selected the complexes derived from the E = 10^-2 ^threshold, hereafter called the E-2 complexes, for further study.

### Features of the E-2 complexes

**Figure 4:**
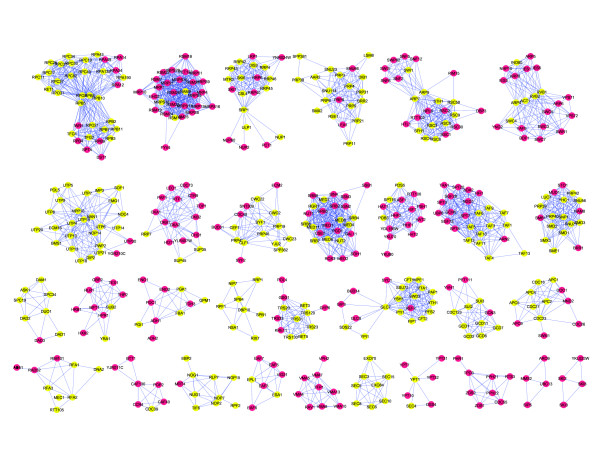
**A subset of the E-2 complex map**. After applying the E = 10^-2 ^threshold to the PICO interaction set, the subset of 5,352 interactions was clustered with MCL, using parameters that maximized correlation with a filtered set of GO component annotations. Interactions within clusters (4,411) were plotted with Cytoscape using the included "organic" layout algorithm. Interactions between clusters (941) were omitted for clarity. Yellow nodes indicate essential proteins; red, nonessential. For the full image please see Additional File 4.

The E-2 complexes contain 1689 proteins grouped into 390 clusters of sizes ranging from two to 35 subunits. A network view of the complexes, generated using Cytoscape [27], is shown in Figure 4; the Cytoscape file is available for download [see Additional File 2]. To measure the accuracy of individual complexes, we tested each for significant enrichment of GO component annotation. GO component annotations enriched at P <0.01 (with Bonferroni correction for multiple hypothesis testing) are noted for each complex [see Additional File 3]. The Simpson coefficient of each enriched annotation is also listed as an easily understood metric for measuring the completeness with which any GO term describes a complex (or vice versa).

The large fraction of E-2 complexes that correspond to existing annotations suggest that the data set is highly accurate. Of the 132 complexes with four or more subunits, 69% (91) are highly enriched for one or more specific GO component annotations; of the 44 complexes of size eight or larger, 84% (37) are so annotated. Furthermore, there are virtually no uncharacterized genes in these large complexes, and the few that appear have relatively weak connections to the other members of their respective clusters. This suggests that the yeast community has achieved a fairly complete description of a large fraction of the "complex-ome," at least for complexes containing many proteins. In fact, only one complex of size four or greater consists entirely of unnamed subunits and thus could be considered truly novel (complex C132, composed of proteins YAL049C, YDL025C, YGR016W, and YHR009C).

Several E-2 clusters represent amalgamations of known complexes. The MCL algorithm assigns each protein to exactly one complex, so protein complexes with shared subunits are sometimes found combined into a single cluster in the E-2 complexes. The C1 cluster, for example, includes RNA polymerase I, II, and III, largely because all three enzymes contain the Rpb5, Rpb8, Rpb10, and Rpo26 subunits. Likewise, complex C7 contains the TAFIID complex and the SAGA transcription factor/chromatin remodeling complex; these complexes share the Taf5, 6, 9, 10, and 12 proteins. It seems clear from the RNA polymerase case that the E-2 clusters occasionally contain discrete complexes that presumably do not physically interact.

Even the clusters that lack significant GO terms tend to have subunits that share similar free-text descriptions in the Saccharomyces Genome Database (SGD) [28]. For example, complex C44 contains eight proteins, all of which are essential. Of these, seven are explicitly described in SGD as being involved in 60 S ribosome biogenesis or as components of 66 S pre-ribosomal particles, and the eighth is involved in export of pre-ribosomal large subunits from the nucleus. No GO term enrichment is found because the CC annotation is typically "nucleolus," a weak term excluded from our analysis (see Methods). Likewise, unannotated complexes C20, C30, and C78 contain 13, 10, and 5 proteins, respectively (10, 9, and 5 essential), that are all known or suspected to be involved in ribosome biogenesis. Other unannotated complexes include C43, eight largely nonessential proteins in the well-described cyclin/cyclin-dependent kinase group; C51, seven nonessential proteins involved in catabolite inactivation of FBPase; and C72, six proteins (five essential), of which five are involved in retrograde Golgi-to-ER trafficking and the sixth, Sec39, is of unknown function but "proposed to be involved in protein secretion."

### Hierarchical structure of co-complex network

**Figure 5:**
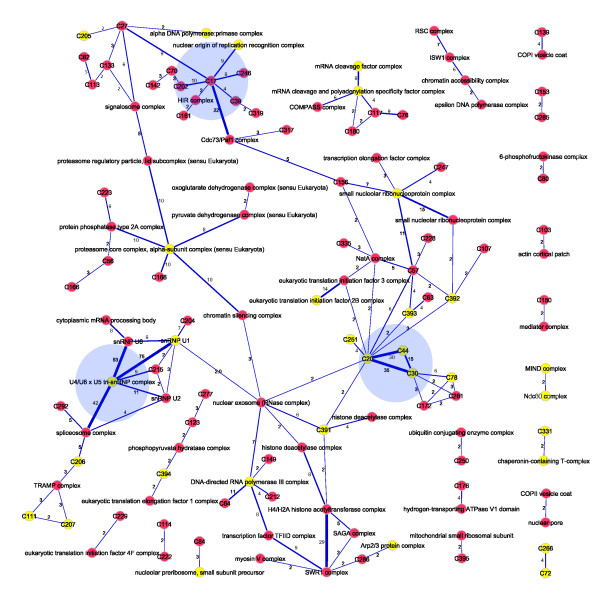
**Inter-complex interactions**. Interactions in the E-2 complex map represent 4,411 of the 5,352 interactions in the PICO network at the E = 10^-2 ^threshold. The 941 remaining protein-protein interactions (PPI) collapse to 248 complex-complex interactions. Here we map 128 inter-complex interactions, each comprising two or more protein-protein interactions (821 PPI total); singletons are omitted for clarity. Nodes represent E-2 complexes: yellow indicates >70% essential subunits; labels indicate highest-scoring GO component, where applicable. Edge thickness reflects number of interactions between complex subunits, ranging from two (thinnest) to 24 or more (thickest) PPI; number of interactions is shown on each edge. Density of PPI between complexes of similar function (e.g. 190 PPI from U4/U6/U5 tri-snRNP complex to neighbors; 86 PPI between C20/C30/C44/C78 ribosome biogenesis modules; 64 PPI linking C17 histone-associated complex to neighbors; shaded in blue) illustrates hierarchical nature of yeast complex network.

The high-confidence subset of the PICO network from which the E-2 complexes were derived contains 5,352 interactions; of these, 4,411 are present in the E-2 complex map of 390 complexes. The remaining 941 interactions all occur between subunits of different complexes. We examined the structure of these interactions by collapsing each complex into a single node and looking at the interactions between complexes. The resulting intercomplex network, depicted in Figure 5, suggests a hierarchical organization of protein complexes in the cell. Over one-third of the interactions (341, or 36%) appear in just three clusters: the U4/U6 × U5 tri-snRNP complex and its neighbors (191 interactions), the C20/C30/C44/C78 ribosome biogenesis nexus (86 interactions), and the C17 histone-associated complex (64 interactions). In all three cases, the intercomplex interactions link complexes that are involved in closely related physiological processes. Taken together, these observations suggest that yeast proteins complexes exhibit a hierarchical organization, with complexes interacting with each other in a well-ordered fashion.

### Essentiality of protein complexes

**Figure 6:**
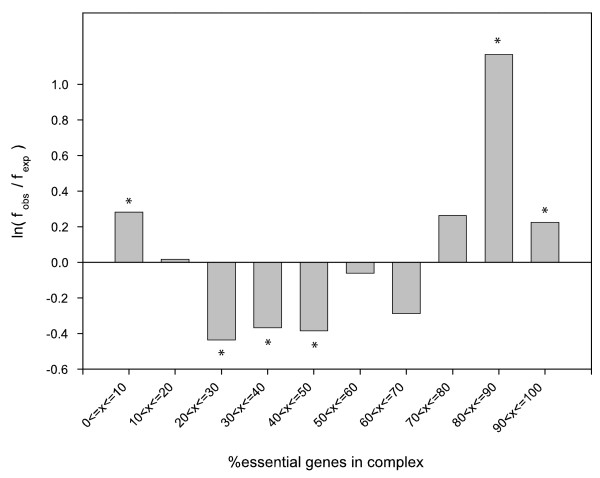
**Essential proteins are concentrated in a subset of complexes**. The distribution of essential proteins in complexes was compared to a randomized background. The fraction of essential proteins in each complex was calculated, sorted into equal-sized bins, and compared to an expected background generated by randomly assigning essential proteins to the same set of complexes. The log ratio of observed to expected frequency for each bin is plotted here: positive values indicate observed frequency above random; negatives indicate below random. The distribution illustrates the concentration of essential proteins in some complexes, and a corresponding absence of essentials in others. Bars marked with an asterisk represent statistically significant deviations from random expectation (P <10^-3^).

The E-2 network shows an enrichment of essential genes in general: the 1689 proteins in the network comprise 29% of all yeast proteins, but contain 58% of all essential proteins (602 essentials out of 1033 total). The descriptions above, as well as a glance at the complex map in Figure 4, suggests concentration of essential proteins into some complexes, and exclusion from others (see Additional file 4). To measure whether there is such a concentration, we considered the distribution of complexes with respect to the fraction of essential proteins in each and sorted this distribution into ten uniformly spaced bins. We bootstrapped a background distribution by randomly assigning the same number of essential genes to an identical set of complexes, repeating this process 10,000 times, and calculating the mean for each bin. We then took the log of the ratio of the observed to the random frequencies in each bin. The results, plotted in Figure 6, show clear enrichment for complexes either mostly essential (>70%) or almost completely nonessential (<10%), with underrepresentation in intermediate values.

## Discussion

### Modular nature of essentiality

The concentration of essential proteins into complexes suggests that essentiality is, in many cases, a product of complex function rather than individual protein function. This phenomenon has been observed by the Barabasi group [5] in an analysis of Ho and Gavin 2002[22]. In using the raw data from these assays, the prior study assigns each bait pull-down to a discrete complex and does not correct for sampling the same complex with multiple baits. Thus, for example, purifications derived from TAP-tagged Nsp1, Nup60, Nup82, and Nup116 are all considered to be discrete complexes with a high fraction of essential proteins, while in reality these factors are all constituents of the same nuclear pore complex.

The current analysis provides both more accurate definition of complexes and, owing to the breadth of the raw data, greater coverage of yeast proteins. The corresponding signal for essentiality of complexes becomes very strong. In the E2 complex set, there are 64 complexes with >70% essential subunits, containing 330 essential out of 379 total proteins – accounting for 32% of all essential genes in yeast. Of these complexes, the 35 largest contain 271 essential proteins (of 320 total), or 26% of all essential genes (Table 1). Other complexes that show strong essentiality include C2, which corresponds to the 26 S proteasome complex. The complex is 58% essential but the diagram of the cluster reveals that it has a number of loosely connected proteins that are not annotated as proteasomal. The 24 core subunits in the diagram are 71% essential. Also, the previously described C7 complex is comprised of the nonessential SAGA complex and the essential TAFIID complex (Figure 4).

**Table 1:** Essential Complexes. Selected essential complexes from the E-2 complex set. Complexes listed are composed of at least 4 subunits, of which >70% are essential. For each complex, the table lists the E-2 complex identifier, the size of the complex, the fraction of essential proteins, the most significant GO cellular component annotation for the complex, and the list of proteins in the complex. Twenty-six percent of all essential genes in yeast are represented in these complexes

**Complex ID**	**Size**	**% Essential**	**Most significantly enriched GO CC term**	**Complex members**
C1	35	74%	DNA-directed RNA polymerase III complex	DST1, IWR1, RET1, RPA12, RPA135, RPA14, RPA190, RPA34, RPA43, RPA49, RPB10, RPB11, RPB2, RPB3, RPB4, RPB5, RPB7, RPB8, RPB9, RPC11, RPC17, RPC19, RPC25, RPC31, RPC34, RPC37, RPC40, RPC53, RPC82, RPO21, RPO26, RPO31, SPT4, TFG1, TFG2
C4	27	93%	small nucleolar ribonucleoprotein complex	BMS1, DIP2, ECM16, EMG1, IMP3, MPP10, NAN1, NOC4, NOP14, POL5, PWP2, SOF1, UTP10, UTP13, UTP14, UTP15, UTP18, UTP20, UTP21, UTP30, UTP4, UTP5, UTP6, UTP7, UTP8, UTP9, YGR210C
C11	20	75%	mRNA cleavage and polyadenylation specificity factor complex	BUD14, CFT1, CFT2, FIP1, GIP3, GLC7, GLC8, MPE1, PAP1, PFS2, PTA1, PTI1, REF2, SDS22, SSU72, SWD2, SYC1, YPI1, YSH1, YTH1
C12	20	85%	U4/U6 × U5 tri-snRNP complex	AAR2, BRR2, DIB1, LEA1, LSM8, PRP11, PRP21, PRP3, PRP31, PRP38, PRP4, PRP6, PRP8, PRP9, RSE1, SMX2, SNU114, SNU23, SNU66, SPP381
C13	18	72%	proteasome core complex, alpha-subunit complex (sensu Eukaryota)	FLC2, GRH1, OSM1, PRE1, PRE10, PRE2, PRE3, PRE4, PRE5, PRE6, PRE7, PRE8, PRE9, PUP1, PUP2, PUP3, RED1, SCL1
C14	18	72%	snRNP U1	BRR1, LUC7, MUD1, NAM8, PRP39, PRP40, PRP42, SMB1, SMD1, SMD2, SMD3, SME1, SMX3, SNP1, SNU56, SNU71, STO1, YHC1
C20	13	77%	(no significant annotation)	BRX1, CIC1, DRS1, ERB1, FPR4, HAS1, MAK5, NOC2, NOC3, PUF6, PWP1, RRP5, YTM1
C26	11	73%	eukaryotic translation initiation factor 2B complex	CDC123, GCD1, GCD11, GCD2, GCD6, GCD7, GCN3, PET111, SUI2, SUI3, YVH1
C30	10	90%	(no significant annotation)	EBP2, MRT4, NOG1, NOP15, NOP2, NOP7, NUG1, RLP7, RPF2, TIF6
C38	8	88%	nuclear pore	GLE2, NIC96, NSP1, NUP116, NUP159, NUP49, NUP57, NUP82
C41	8	88%	DASH complex	ASK1, DAD1, DAD2, DAD3, DAM1, DUO1, SPC19, SPC34
C42	8	100%	exocyst	EXO70, EXO84, SEC10, SEC15, SEC3, SEC5, SEC6, SEC8
C44	8	100%	(no significant annotation)	DBP10, NIP7, NSA1, RIX7, RPF1, RRP1, SPB1, SPB4
C46	7	86%	Arp2/3 protein complex	ARC15, ARC18, ARC19, ARC35, ARC40, ARP2, ARP3
C48	7	71%	DNA replication factor C complex	CTF18, ELG1, RFC1, RFC2, RFC3, RFC4, RFC5
C53	7	100%	transcription factor TFIIH complex	CCL1, KIN28, RAD3, SSL1, TFB1, TFB3, TFB4
C54	7	86%	signal recognition particle (sensu Eukaryota)	LHP1, SEC65, SRP14, SRP21, SRP54, SRP68, SRP72
C55	7	100%	nucleolar ribonuclease P complex	POP1, POP3, POP4, POP5, POP7, POP8, RPP1
C65	6	100%	nuclear origin of replication recognition complex	ORC1, ORC2, ORC3, ORC4, ORC5, ORC6
C67	6	100%	transcription factor TFIIIC complex	TFC1, TFC3, TFC4, TFC6, TFC7, TFC8
C72	6	83%	(no significant annotation)	DSL1, SEC22, SEC39, TIP20, UFE1, USE1
C74	6	100%	chaperonin-containing T-complex	CCT2, CCT3, CCT4, CCT5, CCT6, TCP1
C78	5	100%	(no significant annotation)	IPI1, IPI3, RIX1, RSA4, SDA1
C79	5	100%	nuclear cohesin complex	CDC5, IRR1, MCD1, SMC1, SMC3
C85	5	80%	GINS complex	CTF4, PSF1, PSF2, PSF3, SLD5
C86	5	100%	nuclear condensin complex	BRN1, SMC2, SMC4, YCG1, YCS4
C89	5	80%	nucleolar preribosome, small subunit precursor	ENP1, HRR25, LTV1, RIO2, TSR1
C101	4	100%	MIND complex	DSN1, MTW1, NNF1, NSL1
C106	4	100%	alpha DNA polymerase:primase complex	POL1, POL12, PRI1, PRI2
C110	4	75%	(no significant annotation)	CIA1, MET18, NAR1, YHR122W
C111	4	75%	(no significant annotation)	NAB3, NAB6, NRD1, SEN1
C115	4	100%	mRNA cleavage factor complex	CLP1, PCF11, RNA14, RNA15
C124	4	75%	transcription factor TFIIE complex	DBP2, PPN1, TFA1, TFA2
C92	4	75%	outer plaque of spindle pole body	SPC72, SPC97, SPC98, TUB4
C93	4	100%	Ndc80 complex	NUF2, SPC24, SPC25, TID3

### Comparison to Collins et al

**Figure 7:**
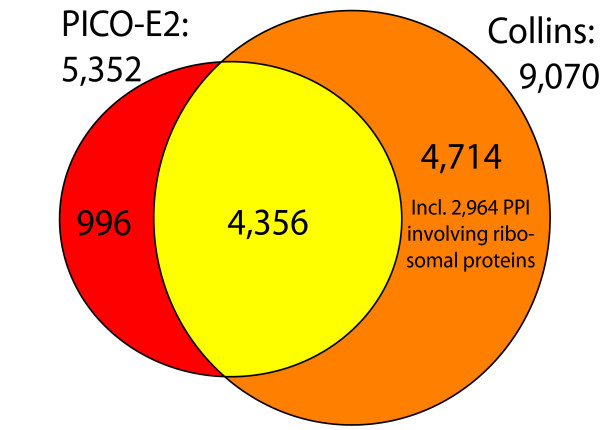


After submission of this article, a study by Collins *et al*. was published in which the Gavin and Krogan TAP-MS data sets were re-analyzed [29]. Using a supervised algorithm derived from Bayesian methods and optimized with empirically-derived parameters, the study posited over 9,000 high-confidence interactions while labeling many previously published interactions as being of lower confidence. Comparing the PICO network at the E = 10^-2 ^threshold (E-2; 5,352 interactions) to the Collins results shows an overlap of 4,356 interactions (Figure 7). The interactions that are unique to the Collins data set are highly enriched for ribosomal proteins: of the 4,714 interactions found in Collins but not PICO, 2,964 involve ribosomal proteins. As these proteins are commonly co-purified with tagged baits in TAP purifications (and subsequently identified by mass spectrometry), they are interpreted as promiscuous interactors in the matrix model of protein connectivity, which considers bait-prey as well as all prey-prey interactions in a given purification. Such high-degree interactors are penalized under the hypergeometric scoring model; therefore, while all such interactions are scored in our model, virtually none exceed the stringent score threshold we applied.

Further comparison shows that the hypergeometric scoring method and the Collins method yielded data sets of nearly equal accuracy. We rank-ordered the two sets of interactions by their respective scores, divided each into bins of 500 interactions, and then plotted the cumulative recall and precision of each versus MIPS co-complex interactions [see Additional File 5]. The Collins data set achieves greater coverage than the PICO network, at somewhat lower overall accuracy, when performance is calculated against the entire MIPS reference. The difference is due almost entirely to the inclusion of ribosomal protein interactions in Collins: when the ribosome is removed from the MIPS reference set, both networks provide nearly identical recall (~34%) and precision (~81%). That the networks generated by the two methods overlap so strongly, despite our inclusion of the Ho dataset and use of a much higher confidence threshold for the Krogan raw data, suggests the networks capture a highly accurate subset of yeast co-complex interactions, and that the simple probabilistic method offered in this study is an effective tool for assigning relative confidence rankings to observations in large-scale data sets.

It is worth noting that even the highest-scoring interactions in the two analyses do not reach 100% precision versus the MIPS reference. This is in part due to the incompleteness of the reference set. An interaction is defined as a false positive if and only if both its corresponding proteins are present in the reference set but the interaction is not. Thus, true interactions that are detected experimentally but absent from the reference set will be scored as false positives (provided the proteins are present in the reference set). We observe several cases of this. For example, the Tub4 gamma tubulin complex is composed of Spc97, Spc98, and Tub4, as defined by GO Cellular Component and MIPS annotation. The E2 derived complex also includes Spc72, the spindle pole body component which interacts with the Tub4p complex [30]. The MIPS reference does not include Spc72 in the gamma tubulin complex but does include the protein in the "Spindle Pole Body Components" collection of proteins. Thus interactions between Spc72 and other members of the gamma tubulin complex, while almost certainly "true" co-complex interactions, are scored as false positives when calculating precision versus MIPS. All such experimentally detected inter-complex interactions are absent in the MIPS reference set. Thus the incompleteness of the reference set prevents a high-accuracy experimental data set from achieving perfect precision.

## Conclusion

We have described a simple yet robust unsupervised method of assigning confidence levels to interactions observed in a large-scale assay, as well as combining data from independent assays into an integrated whole that can be used for further study. We used this method to integrate data from three large-scale affinity purification-mass spectrometry assays in yeast to generate a high-confidence subset of interactions, from which we derived an accurate set of protein complexes. The recall of MIPS co-complex interactions indicates that no more than 46% of the total co-complex interactome in yeast has been assayed by TAP-MS methods (with only 34% in the high confidence E2 set). Nonetheless, the high proportion of complexes that correspond to existing annotations and the small number of uncharacterized genes present in our high-confidence data strongly suggest that the community has largely saturated the fraction of the complex-ome that is accessible to the methods (TAP-MS) and conditions (aerobic growth in rich media) that have been explored so far. Therefore, it would likely be fruitful to explore other conditions and growth states to extend the interactome.

Our complex data also support the notion that, in many cases, essentiality is tied not to the protein or gene itself, but to the molecular machine to which that protein belongs. We can clearly separate the majority of complexes into essential and nonessential. The few that are mixed – for example, the SAGA/TAFIID complex – lead to interesting questions about the essentiality of specific interactions [31]. We anticipate that the complex descriptions offered here, as well as the general scoring method, can be used in other functional genomics and systems biology studies.

## Methods

### Data sources

Data from Ho *et al*. were taken from Table S1 of [17]. Interactions from Gavin *et al*. were taken from Supplementary Table 1 of [11]. In both cases, bait-prey pairs were generated from the list of purifications, with the bait removed from the prey list if applicable. Interactions from Krogan *et al*. [12] were taken from the raw LCMS and MALDI purification data. Bait-prey pairs from LCMS purifications with confidence > = 99.6 and MALDI purifications with score > = 3.4 were included. Matrix-model data sets were generated by considering all prey-prey pairs if both prey were purified from the same bait.

### Reference data sets

MIPS filtered data: The MIPS curated complex data were downloaded from mpact[23]. All high-throughput data, as well as the large and small ribosomal subunits, were excluded. An all-by-all set of interactions was generated from each complex and used as a reference to calculate recall/precision curves of experimental data. The co-complex data was used to calculate the Hubert statistic.

GO filtered reference set: The complete yeast GO Cellular Component ontology was downloaded from the Saccharomyces Genome Database [28] on 5 December 2006. Annotations were sorted by the number of genes to which they applied; all annotations equal to or larger than the size of the "small cytoplasmic ribosomal subunit" were discarded. The resulting set of annotations is mostly complexes, with a small number of discrete cellular localizations included. This annotation set was used to calculate GO term enrichment and the Hubert statistic.

### Analaytic techniques

The MCL program was downloaded from [24]. For each E-value threshold of the PICO network, MCL was run with the following parameter space: -I, 1.8 to 3.0 in 0.2 increments; -C, 0.5 to 1.5 in 0.25 increments; -S, 0 to 7. The Hubert statistic (H) was calculated for each MCL result against the GO filtered reference set and the MCL result with the highest H score was considered the optimal result for that E-value. The -S parameter was found to have no effect on our results.

Calculation of the Hubert statistic, H, was performed as described in [26]. As the matrices must be equal size, the calculation was performed on the potential interaction space defined by the set of proteins present in both the experimental and reference protein sets.

The Simpson coefficient, C_s _of similarity between sets of proteins A and B, is:

C_s _= (# proteins in A and B)/min(# proteins in A, # proteins in B)

The list of essential ORFS was downloaded from the Saccharomyces Genome Database. We considered only verified or uncharacterized ORFs.

## Authors' contributions

IL developed the probabilistic scoring method. TH conceived of the study, performed the data collection and analysis, and drafted the manuscript under the guidance and supervision of EMM. All authors read an approved the final manuscript.

## Supplementary Material

Additional File 1 This table gives all co-complex interactions in the combined PICO network and their associated scores, given as -ln(p-value). Click here for file

Additional File 2 Click here for file

Additional File 3 Each complex is shown on a single line containing the following information: Complex ID, Size, %Essential, List of Proteins(| - deliminated). If the constituent proteins are enriched for a GO Cellular Component annotation (see Methods) then subsequent lines contain the following information: Enrichment score(-ln(p)),  Simpson coefficient, Annotation name/GO_ID/(Number of proteins in GO with this annotation) Click here for file

Additional File 4 **A subset of the E-2 complex map**. After applying the E = 10^-2 ^threshold to the PICO interaction set, the subset of 5,352 interactions was clustered with MCL, using parameters that maximized correlation with a filtered set of GO component annotations. Interactions within clusters (4,411) were plotted with Cytoscape using the included "organic" layout algorithm. Interactions between clusters (941) were omitted for clarity. Yellow nodes indicate essential proteins; red, nonessential. Click here for file

Additional File 5 Click here for file
