# Supplementary material for: A high-accuracy consensus map of yeast protein complexes reveals modular nature of gene essentiality
Source: BMC Bioinformatics. 2007 Jul 2;8:236. doi: 10.1186/1471-2105-8-236 (PMC1940025; doi:10.1186/1471-2105-8-236)
Supplement: Additional File 1 — This table gives all co-complex interactions in the combined PICO network and their associated scores, given as -ln(p-value). [file 1471-2105-8-236-S1.htm]

```
This table gives all co-complex interactions in the combined PICO network
and their associated scores, given as -ln(p-value).

YEL056W	YPL001W	429.1028
YEL056W	YLL022C	376.432
YER022W	YNL236W	344.5258
YMR093W	YPL126W	320.9165
YDR324C	YPL126W	320.349
YLL022C	YPL001W	308.8954
YDR324C	YMR093W	295.7511
YGL019W	YOR061W	288.5397
YJL115W	YPL001W	280.4891
YEL056W	YGL207W	280.2776
YGL207W	YPL001W	279.0765
YEL056W	YJL115W	278.3226
YGR128C	YPL126W	271.9738
YGR128C	YMR093W	255.0709
YGL207W	YJL115W	248.3042
YDR324C	YGR128C	246.9095
YGL207W	YML069W	238.0334
YGL019W	YOR039W	213.8673
YJL115W	YLL022C	211.8089
YGL207W	YLL022C	211.4861
YEL056W	YML069W	190.8622
YOL054W	YPL001W	190.4976
YML069W	YPL001W	190.0981
YEL056W	YOL054W	187.9355
YDL132W	YGL252C	181.8612
YOR039W	YOR061W	179.4388
YIL035C	YOR061W	176.1191
YER022W	YHR041C	175.8077
YEL056W	YNL030W	174.0323
YNL030W	YPL001W	171.0643
YHR196W	YMR093W	169.1952
YDR190C	YPL235W	167.9251
YGL207W	YOL054W	167.508
YJL115W	YML069W	165.1006
YHR069C	YOR001W	164.1527
YDR427W	YOR261C	163.8362
YCR035C	YOR001W	163.2719
YDR427W	YPR108W	159.0632
YGL019W	YIL035C	158.5409
YJL115W	YOL054W	155.4349
YCR035C	YNL232W	154.6363
YNL232W	YOR001W	151.4426
YGL011C	YOR362C	151.2522
YGR253C	YOR362C	150.1781
YBL041W	YOR362C	149.7987
YDR427W	YFR004W	149.1561
YGL207W	YNL030W	147.3666
YHR041C	YNL236W	146.8017
YOL021C	YOR001W	146.5766
YCR035C	YHR069C	146.2172
YHR196W	YPL126W	145.8177
YNL330C	YPL139C	145.1704
YOL038W	YOR362C	144.8574
YGR135W	YOR362C	143.9478
YGL011C	YGR135W	143.0454
YLR033W	YMR033W	143.0165
YLL022C	YOL054W	142.7928
YLL022C	YML069W	142.6214
YGL011C	YOL038W	142.0534
YER022W	YGL151W	141.8378
YLR347C	YNL189W	140.8258
YJL115W	YNL030W	140.2083
YER094C	YGL011C	138.7064
YDL147W	YDR427W	138.531
YDR427W	YFR052W	138.3285
YER094C	YOR362C	138.2743
YCR035C	YOL021C	138.1692
YGL151W	YNL236W	135.6283
YGL011C	YGR253C	135.3784
YBL041W	YGR253C	134.543
YGR135W	YGR253C	134.1731
YOL142W	YOR001W	133.8803
YHR069C	YNL232W	133.8509
YGR253C	YOL038W	133.5754
YNL232W	YOL021C	133.3246
YHR069C	YOL021C	132.8121
YML092C	YOR362C	132.7029
YDR427W	YIL075C	132.5536
YBL041W	YOL038W	132.3032
YNL232W	YOL142W	132.2306
YGR135W	YOL038W	132.0366
YBL041W	YGL011C	131.9009
YDR324C	YHR196W	131.4322
YLL022C	YNL030W	130.7036
YCR035C	YOL142W	130.3796
YDR138W	YML062C	130.3086
YFR004W	YOR261C	129.4336
YGR253C	YML092C	128.1357
YDR075W	YNL201C	127.895
YDR427W	YGL048C	127.023
YGR195W	YOR001W	126.7552
YDR427W	YOR117W	126.1558
YER094C	YGR253C	125.7953
YCR035C	YGR195W	124.5855
YFR037C	YLR033W	124.0018
YML092C	YOL038W	123.8011
YOL021C	YOL142W	123.7132
YGL011C	YML092C	123.4181
YBL041W	YGR135W	123.3787
YDR427W	YER021W	123.278
YER094C	YOL038W	123.2415
YJL001W	YOR362C	122.8067
YFR004W	YPR108W	122.7836
YDL111C	YOR001W	122.0648
YOR261C	YPR108W	121.8887
YDR449C	YLR222C	121.8262
YDL147W	YOR261C	121.3934
YAL016W	YGL190C	121.3075
YBL041W	YER094C	121.0396
YGR195W	YNL232W	120.971
YDL147W	YFR004W	120.013
YFR052W	YIL075C	119.6611
YFR037C	YMR033W	119.2365
YCR035C	YDL111C	119.2323
YML069W	YOL054W	119.0543
YFR004W	YFR052W	118.8321
YER094C	YGR135W	118.7838
YGL048C	YIL075C	118.683
YDL147W	YPR108W	118.492
YMR146C	YMR309C	118.4505
YGR135W	YML092C	118.1668
YER094C	YML092C	118.1502
YBR009C	YPL001W	117.1228
YDR394W	YDR427W	116.7246
YDL111C	YNL232W	116.0949
YFR052W	YOR261C	115.8242
YHR069C	YOL142W	115.7241
YBL041W	YML092C	115.5615
YDR427W	YHR027C	114.765
YMR314W	YOR362C	114.5936
YCR052W	YLR033W	114.4504
YER021W	YOR261C	113.09
YFR050C	YGL011C	112.9816
YBL041W	YJL001W	112.4206
YMR033W	YPR034W	112.3561
YBR009C	YEL056W	112.3049
YKR008W	YLR033W	111.21
YFR050C	YOR362C	110.9588
YDR449C	YLR129W	110.7941
YFR037C	YLR357W	110.5313
YDL097C	YDR427W	110.5062
YLL004W	YPR162C	110.5025
YLR033W	YLR357W	110.2712
YFR037C	YMR091C	110.2673
YLR033W	YML127W	110.0642
YDR394W	YIL075C	110.0231
YGR253C	YJL001W	109.5632
YGR195W	YOL142W	108.8221
YMR049C	YOR272W	108.8209
YGL011C	YMR314W	108.5435
YDL132W	YJL047C	108.3995
YCR052W	YMR033W	108.0505
YGR135W	YMR314W	107.8821
YIL075C	YPR108W	107.4879
YGR195W	YOL021C	107.2441
YLR357W	YMR033W	107.0151
YGR128C	YHR196W	106.8088
YLR115W	YLR277C	106.75
YCR052W	YFR037C	106.72
YJL001W	YOL038W	106.539
YGL011C	YJL001W	106.3593
YFR004W	YIL075C	106.0832
YDR394W	YGL048C	105.996
YDL097C	YFR052W	105.6473
YCR052W	YKR008W	105.546
YGR095C	YOR001W	105.4259
YLR033W	YMR091C	104.5406
YER021W	YFR004W	104.5192
YER012W	YER094C	104.4794
YGR195W	YHR069C	104.401
YER021W	YFR052W	104.2964
YHR027C	YIL075C	104.1099
YFR050C	YOL038W	103.9997
YGR135W	YJL001W	103.8017
YFR050C	YGR135W	103.7886
YOR117W	YPR108W	103.7834
YMR314W	YOL038W	103.6755
YDR280W	YOR001W	103.5793
YIL035C	YOR039W	103.4946
YBR198C	YGL112C	103.4493
YDL147W	YFR052W	102.7728
YER094C	YFR050C	102.6151
YDR394W	YPR108W	102.6111
YFR052W	YPR108W	102.4077
YCR035C	YDR280W	102.3515
YER021W	YPR108W	102.228
YER012W	YGL011C	102.2267
YJL001W	YML092C	102.118
YBL041W	YFR050C	101.986
YJR065C	YKL013C	101.8638
YGL048C	YPR108W	101.7304
YIL075C	YOR261C	101.717
YLL004W	YNL261W	101.6896
YGR253C	YMR314W	101.4071
YCR002C	YJR076C	101.0564
YDL007W	YDR427W	100.7384
YMR146C	YPR041W	100.6312
YDL147W	YER021W	100.5632
YER021W	YIL075C	100.4221
YDR394W	YOR261C	100.3631
YDL111C	YHR069C	100.3352
YBL041W	YMR314W	100.1997
YFR052W	YGL048C	100.0699
YKR002W	YLR277C	99.9096
YDL111C	YOL021C	99.8046
YKL013C	YNR035C	99.7744
YPR110C	YPR190C	99.5879
YCR052W	YLR357W	99.5223
YCR035C	YGR095C	99.4839
YGR095C	YNL232W	99.4096
YIL075C	YKL145W	99.3264
YFR004W	YGL048C	99.2096
YBR055C	YKL173W	98.3898
YER012W	YOR362C	98.2259
YDR138W	YNL253W	98.0802
YOR340C	YPR110C	97.4261
YHR027C	YOR261C	97.4126
YJR007W	YPL237W	97.3574
YDR280W	YNL232W	97.259
YDL097C	YIL075C	97.1924
YDL111C	YGR195W	97.1383
YPR010C	YPR110C	96.6697
YGL048C	YOR117W	96.444
YNL030W	YOL054W	96.3554
YFR051C	YIL076W	95.93
YJR065C	YNR035C	95.8077
YML069W	YNL030W	95.7786
YNL261W	YPR162C	95.3457
YER094C	YJL001W	95.2758
YLR357W	YMR091C	95.2573
YDR394W	YFR004W	95.2067
YAL043C	YLR277C	95.0734
YBL041W	YER012W	95.0515
YGL048C	YOR261C	94.9051
YIL075C	YOR117W	94.4954
YER012W	YGR253C	94.4453
YDR429C	YMR146C	94.4055
YDR280W	YHR069C	94.2191
YFR050C	YGR253C	94.0772
YMR033W	YMR091C	94.0601
YLR129W	YLR222C	93.6422
YBR079C	YMR309C	93.6158
YDR394W	YOR117W	93.3477
YLR033W	YLR321C	93.2342
YLR033W	YPR034W	93.2152
YGL244W	YLR418C	93.2131
YMR309C	YPR041W	93.1231
YOR117W	YOR261C	92.7852
YDL097C	YPR108W	92.736
YKL059C	YLR115W	92.686
YDL111C	YOL142W	92.0191
YDR427W	YKL145W	91.984
YLR115W	YNL317W	91.9488
YCR052W	YMR091C	91.849
YLR277C	YNL317W	91.7487
YDL097C	YFR004W	91.564
YBR009C	YGL207W	91.1493
YGR095C	YOL142W	91.1217
YML092C	YMR314W	91.0226
YBR154C	YPR110C	90.8693
YDL097C	YDL147W	90.8554
YOR207C	YPR190C	90.5797
YOR340C	YPR010C	90.3952
YDR190C	YLR085C	90.3089
YDR429C	YMR309C	90.3065
YAL043C	YLR115W	90.3052
YFR004W	YOR117W	90.0275
YKR002W	YNL317W	89.9909
YCR002C	YHR107C	89.9527
YBL046W	YNL201C	89.7856
YBR087W	YJR068W	89.5419
YDR394W	YFR052W	89.4262
YKR002W	YLR115W	89.3096
YBL045C	YPR191W	89.1028
YBR009C	YJL115W	89.0722
YKR008W	YMR033W	89.0618
YDR427W	YOR259C	88.9273
YDR280W	YOL021C	88.8559
YDL097C	YGL048C	88.8345
YEL056W	YNL206C	88.8025
YDR280W	YOL142W	88.5873
YNL206C	YPL001W	88.5084
YCR052W	YPR034W	88.4974
YDR394W	YKL145W	88.2294
YDR394W	YER021W	88.1402
YDR145W	YGL112C	88.0245
YGR095C	YOL021C	87.9406
YER094C	YMR314W	87.9256
YFR050C	YML092C	87.5149
YIL061C	YKL012W	87.0059
YFR037C	YKR008W	86.6204
YER012W	YOL038W	86.522
YBR279W	YLR418C	86.3516
YCR002C	YLR314C	86.1638
YML127W	YMR033W	85.9524
YDL097C	YOR261C	85.9485
YBR087W	YNL290W	85.6049
YDL097C	YKL145W	85.591
YFR052W	YOR259C	85.141
YJR076C	YLR314C	84.9608
YDL097C	YER021W	84.772
YER021W	YGL048C	84.7301
YNL248C	YOR340C	84.5896
YKL059C	YLR277C	84.5645
YHR027C	YKL145W	84.4457
YER012W	YML092C	84.4215
YBL046W	YDR075W	84.3549
YFR052W	YHR027C	84.2688
YFR037C	YML127W	84.2426
YHR027C	YPR108W	84.1803
YKR008W	YLR357W	83.9137
YGR095C	YHR069C	83.9066
YAL043C	YKL018W	83.8845
YFR004W	YHR027C	83.8817
YKR008W	YPR034W	83.8444
YDL147W	YIL075C	83.5828
YFR004W	YOR259C	83.578
YDR477W	YGL115W	83.5722
YDR238C	YIL076W	83.5512
YDL111C	YGR095C	83.5438
YHR107C	YJR076C	83.5433
YLR418C	YOL145C	83.5334
YGL048C	YKL145W	83.0669
YFR052W	YOR117W	82.72
YDR394W	YHR027C	82.5794
YOR259C	YOR261C	82.5006
YLR321C	YMR033W	82.2934
YMR290C	YOR206W	82.2826
YPL139C	YPR023C	82.2185
YOR207C	YPR110C	82.0764
YIL021W	YOR151C	81.954
YFR050C	YMR314W	81.9489
YBR103W	YIL112W	81.8901
YDR138W	YHR167W	81.8647
YER021W	YKL145W	81.8589
YAR007C	YNL312W	81.7568
YDL147W	YOR117W	81.6488
YCR052W	YLR321C	81.6103
YDR427W	YHR200W	81.4141
YER012W	YGR135W	81.2971
YDL084W	YDR138W	81.2515
YAL043C	YNL317W	81.2503
YDL029W	YJR065C	81.0996
YBR009C	YLL022C	80.8284
YFR052W	YHR200W	80.7632
YDL007W	YGL048C	80.7246
YGR013W	YML046W	80.7147
YLR357W	YML127W	80.6357
YAL016W	YDL134C	80.5893
YDR427W	YFR010W	80.55
YFR037C	YPR034W	80.4835
YFR052W	YKL145W	80.4317
YDL007W	YFR004W	80.2373
YBR079C	YMR146C	80.2289
YLR321C	YPR034W	80.2072
YCR052W	YML127W	80.1596
YJL001W	YMR314W	80.0327
YFR010W	YIL075C	80.0193
YNR003C	YPR190C	79.9242
YNL248C	YPR110C	79.8846
YFR004W	YHR200W	79.6959
YJL041W	YJL061W	79.5118
YKL059C	YKR002W	79.4761
YKL059C	YNL317W	79.4733
YGR005C	YGR186W	79.4246
YGR095C	YGR195W	79.248
YJR068W	YOL094C	79.2341
YER021W	YHR027C	79.2113
YAL043C	YGR156W	79.1581
YIL021W	YJL140W	79.0311
YDL029W	YKL013C	78.5256
YDL007W	YOR261C	78.4435
YNL330C	YPR023C	78.2139
YCR057C	YDR449C	78.1919
YJR068W	YNL290W	78.106
YCR035C	YOR076C	78.101
YBR198C	YDR145W	77.9053
YDL147W	YDR394W	77.7759
YGL048C	YHR027C	77.6716
YAL043C	YKL059C	77.478
YDL007W	YFR052W	77.446
YKL018W	YLR277C	77.3827
YDL132W	YDR328C	77.27
YDL147W	YHR027C	77.0893
YAL043C	YKR002W	76.978
YFR004W	YFR010W	76.9651
YFR004W	YKL145W	76.8614
YKL145W	YOR261C	76.8361
YBR060C	YLL004W	76.7501
YBR193C	YER022W	76.4524
YJL069C	YLR222C	76.3233
YER021W	YOR117W	76.3168
YKR008W	YMR091C	76.2625
YCR057C	YLR222C	76.2099
YDR240C	YML046W	76.0071
YIL075C	YOR259C	75.8278
YML127W	YMR091C	75.8218
YKL145W	YOR117W	75.7646
YHR027C	YOR117W	75.738
YGL151W	YHR041C	75.6663
YDL007W	YOR117W	75.5958
YHR107C	YLR314C	75.5731
YGL048C	YOR259C	75.4985
YKR008W	YLR321C	75.4907
YER021W	YOR259C	75.4484
YDL147W	YGL048C	75.411
YDL147W	YFR010W	75.3805
YDR394W	YOR259C	75.183
YDR190C	YJL081C	75.151
YDR190C	YFL039C	75.0475
YKL145W	YPR108W	74.941
YDL134C	YGL190C	74.7767
YDL007W	YIL075C	74.6839
YBL008W	YOR038C	74.3249
YER012W	YMR314W	74.2754
YDL007W	YPR108W	74.03
YGR078C	YNL153C	73.9966
YDL145C	YIL076W	73.9769
YIL062C	YKL013C	73.8947
YDL097C	YHR200W	73.8824
YKR008W	YML127W	73.7474
YGR103W	YMR049C	73.7008
YBR087W	YOL094C	73.655
YFR010W	YFR052W	73.5968
YGR013W	YKL012W	73.4748
YDL007W	YER021W	73.2231
YER022W	YGR104C	73.2059
YDL097C	YDR394W	73.1463
YDR235W	YML046W	73.1417
YKL018W	YKR002W	73.1119
YGL207W	YNL206C	73.0244
YFL024C	YGR002C	73.0146
YDL007W	YDR394W	72.9912
YGR156W	YKL059C	72.698
YHR200W	YOR261C	72.6647
YER133W	YLR277C	72.6446
YGR078C	YML094W	72.5991
YKL012W	YML046W	72.545
YLL036C	YLR117C	72.4803
YER012W	YJL001W	72.3941
YDL150W	YPR190C	72.2679
YDL147W	YOR259C	72.2617
YFR050C	YJL001W	72.0176
YIL061C	YML046W	71.9958
YLR293C	YOR185C	71.9658
YNL248C	YPR010C	71.9413
YDR429C	YPR041W	71.8601
YML127W	YPR034W	71.6279
YOR117W	YOR259C	71.4334
YDL147W	YKL145W	71.4017
YER012W	YFR050C	71.3992
YLR321C	YML127W	71.3503
YDL175C	YOL115W	71.0977
YER022W	YLR071C	71.0888
YKL018W	YLR115W	70.9282
YGR013W	YIL061C	70.9277
YKL018W	YNL317W	70.8397
YLR357W	YPR034W	70.7727
YGR005C	YJL140W	70.646
YDR394W	YFR010W	70.6185
YCR012W	YHR174W	70.4766
YDL097C	YOR259C	70.4224
YDR238C	YFR051C	70.3685
YLR321C	YLR357W	70.22
YIL017C	YIL097W	70.1646
YGR156W	YLR115W	70.1004
YER021W	YFR010W	70.0484
YDL014W	YMR290C	69.8307
YBR279W	YOL145C	69.8245
YHR200W	YIL075C	69.766
YDL029W	YNR035C	69.7166
YOR259C	YPR108W	69.6857
YDL132W	YGL143C	69.6761
YGL112C	YGR252W	69.664
YBR055C	YPR178W	69.642
YLR015W	YPL138C	69.6302
YBR279W	YGL244W	69.5932
YKL145W	YOR259C	69.5684
YDL007W	YDL097C	69.5269
YBR060C	YPR162C	69.5086
YDL007W	YOR259C	69.439
YLR321C	YMR091C	69.1425
YHR197W	YNL182C	69.0568
YGR156W	YKR002W	69.014
YOR001W	YOR076C	68.9921
YJL115W	YNL206C	68.9241
YDL145C	YDR238C	68.8832
YFR037C	YLR321C	68.7306
YER021W	YHR200W	68.5632
YDR390C	YPR180W	68.489
YDL111C	YDR280W	68.3593
YDR449C	YJL069C	68.3114
YFL024C	YJL081C	67.9498
YBR009C	YML069W	67.9316
YAL016W	YDL188C	67.8782
YER133W	YKR002W	67.7076
YFR010W	YPR108W	67.7071
YHR069C	YOR076C	67.6439
YFR010W	YOR261C	67.5137
YKL013C	YLR370C	67.5125
YGL048C	YHR200W	67.4304
YOL004W	YPL139C	67.1392
YDR394W	YHR200W	67.0627
YLR418C	YOR123C	67.0392
YDR301W	YKR002W	67.0234
YLR212C	YNL126W	66.8292
YNL113W	YPR110C	66.8086
YDL145C	YNL287W	66.5818
YCR002C	YDL225W	66.4102
YDR138W	YNL139C	66.4001
YER172C	YKL173W	66.359
YDL097C	YOR117W	66.238
YFL013C	YOR141C	66.1987
YDR238C	YNL287W	66.0486
YLR192C	YMR146C	66.0398
YDL002C	YOR141C	66.0069
YGL252C	YJL047C	65.9601
YDR211W	YOR260W	65.9569
YGL019W	YLR418C	65.8723
YMR075W	YPL139C	65.7882
YDR473C	YPR178W	65.5759
YGR156W	YKL018W	65.5235
YGR103W	YOR272W	65.4874
YGR156W	YNL317W	65.4594
YMR075W	YNL330C	65.4057
YBR154C	YOR340C	65.3945
YJL140W	YOR151C	65.3231
YDL007W	YHR027C	65.3158
YGL227W	YIL097W	65.3073
YDR448W	YGR252W	65.2892
YCR052W	YIL126W	65.1742
YFL024C	YNL107W	65.1002
YJL069C	YLR129W	65.0928
YBL041W	YPR103W	65.0179
YDR359C	YFL024C	64.8078
YLL022C	YNL206C	64.7675
YER172C	YHR165C	64.745
YMR091C	YPR034W	64.6252
YDR448W	YGL112C	64.5866
YER133W	YKL018W	64.5316
YML062C	YNL253W	64.445
YDR235W	YDR240C	64.438
YBR060C	YNL261W	64.4201
YER133W	YNL317W	64.3199
YGL244W	YOL145C	64.291
YDL145C	YFR051C	64.2565
YKL018W	YKL059C	64.1411
YIL076W	YPL010W	64.038
YFR010W	YGL048C	63.9857
YBR279W	YOR123C	63.98
YHR081W	YOR001W	63.8752
YDL147W	YHR200W	63.722
YDL007W	YKL145W	63.5998
YIL062C	YJR065C	63.5911
YML010W	YNL201C	63.5902
YLR071C	YNL236W	63.4895
YDR427W	YGR232W	63.4687
YDL007W	YDL147W	63.4231
YGR002C	YNL107W	63.2232
YDR303C	YLR033W	63.1957
YNL330C	YOL004W	63.1766
YHR200W	YKL145W	63.139
YLR370C	YNR035C	63.0661
YGR002C	YJL081C	63.032
YOR341W	YPR010C	62.9173
YGL244W	YOR123C	62.9137
YDL097C	YFR010W	62.8847
YDR301W	YLR277C	62.8508
YJL176C	YNR023W	62.8216
YJR065C	YLR370C	62.7247
YFR010W	YOR259C	62.7218
YDR280W	YGR095C	62.5804
YBR081C	YPL254W	62.5753
YDL097C	YHR027C	62.5651
YOL021C	YOR076C	62.555
YBR193C	YNL236W	62.5063
YIL126W	YLR357W	62.3644
YNL232W	YOR076C	62.3162
YBR079C	YPR041W	62.2945
YCR057C	YLR129W	61.9846
YGR156W	YLR277C	61.9686
YIL126W	YMR091C	61.8829
YBR009C	YOL054W	61.8266
YIL062C	YNR035C	61.7994
YFL008W	YJL074C	61.5792
YJL011C	YPR190C	61.4218
YBR055C	YGR091W	61.3836
YHR200W	YOR259C	61.3459
YIL126W	YMR033W	61.2334
YGL128C	YLR424W	61.2105
YER133W	YKL059C	61.1523
YBR195C	YML102W	61.1376
YDR176W	YGL112C	60.9431
YHR027C	YHR200W	60.849
YDR311W	YPR056W	60.7731
YIL084C	YNL097C	60.6827
YDL087C	YGR013W	60.5989
YJR007W	YKR026C	60.5869
YCL011C	YDR138W	60.5557
YLR052W	YOR141C	60.492
YGR195W	YOR076C	60.4319
YDL188C	YGL190C	60.4109
YDR280W	YGR195W	60.3541
YFR037C	YIL126W	60.2695
YFR010W	YKL145W	60.2614
YKR026C	YOR260W	60.2067
YGR083C	YKR026C	60.167
YHR027C	YOR259C	60.1509
YHR200W	YPR108W	60.1217
YGL137W	YIL076W	60.0823
YDR301W	YLR115W	60.0221
YOR362C	YPR103W	59.9616
YGR005C	YIL021W	59.9165
YIL097W	YMR135C	59.7878
YGR104C	YNL236W	59.7036
YDL087C	YML046W	59.6613
YMR075W	YPR023C	59.619
YFL039C	YJL081C	59.6109
YDL126C	YEL056W	59.5983
YBR081C	YGL112C	59.5754
YIL126W	YLR321C	59.5673
YFR052W	YGR232W	59.4809
YDR303C	YKR008W	59.3474
YDL126C	YPL001W	59.3431
YGR200C	YPL086C	59.3097
YJR093C	YKR002W	59.3001
YFL024C	YPR023C	59.2382
YBR079C	YDR429C	59.2195
YGL133W	YOR304W	59.1347
YDR145W	YMR236W	59.1048
YDR118W	YHR166C	59.0146
YDL145C	YGL137W	58.9719
YHR166C	YLR127C	58.8662
YCR077C	YJL124C	58.7919
YGL241W	YKR048C	58.7606
YHR167W	YNL253W	58.7211
YFL024C	YOR244W	58.7059
YOR124C	YOR138C	58.648
YFR051C	YPL010W	58.5933
YML094W	YNL153C	58.5819
YKL173W	YPR178W	58.4683
YAR003W	YPL138C	58.4401
YDR235W	YGR013W	58.3653
YNL290W	YOL094C	58.2961
YLR192C	YPR041W	58.2892
YOR141C	YPL235W	58.2727
YIL084C	YPL139C	58.2522
YIL126W	YKR008W	58.2293
YOR341W	YPR110C	58.2099
YCR052W	YDR303C	58.1428
YKR025W	YOR207C	58.0658
YIL144W	YOL069W	58.0464
YDL150W	YPR110C	58.0426
YER133W	YLR115W	58.0384
YIL084C	YNL330C	58.0023
YDL007W	YHR200W	58.0011
YGR200C	YPL101W	57.9844
YGR180C	YJL026W	57.9165
YJL081C	YPL235W	57.8663
YKR026C	YLR291C	57.8486
YBR154C	YOR224C	57.7561
YER018C	YOL069W	57.641
YBR154C	YJL140W	57.5968
YBR154C	YNL248C	57.5936
YLR147C	YLR275W	57.4878
YDR190C	YOR141C	57.4692
YIL035C	YLR418C	57.4205
YDR211W	YGR083C	57.3947
YGR083C	YOR260W	57.3527
YBR234C	YKL013C	57.3487
YDL225W	YJR076C	57.1405
YDL111C	YOR076C	57.1353
YDR176W	YGR252W	57.1299
YBR055C	YDR473C	57.0502
YDR255C	YIL097W	57.0367
YER029C	YLR147C	56.9927
YDR324C	YJL109C	56.9473
YOR116C	YOR207C	56.9427
YER022W	YOR174W	56.8964
YDR404C	YJL140W	56.8345
YBR234C	YJR065C	56.8247
YDR240C	YGR013W	56.82
YER025W	YJR007W	56.8182
YDL140C	YIL021W	56.801
YDL225W	YLR314C	56.7878
YNR003C	YPR110C	56.773
YDR301W	YNL317W	56.7244
YOR174W	YPR070W	56.691
YJR093C	YKL059C	56.6266
YGR252W	YLR055C	56.5748
YBR009C	YNL030W	56.5485
YDR425W	YJL036W	56.5249
YDL225W	YHR107C	56.5245
YMR236W	YPL011C	56.4217
YBR251W	YDR175C	56.3705
YDL150W	YOR207C	56.314
YIL076W	YNL287W	56.2228
YHR118C	YLL004W	56.2112
YJL001W	YPR103W	56.1871
YHR118C	YPR162C	56.0832
YGR005C	YOR151C	56.0179
YNR003C	YOR207C	55.9663
YHR165C	YKL173W	55.953
YAL043C	YER133W	55.9063
YLR192C	YMR309C	55.9002
YDR404C	YGR005C	55.8923
YFL039C	YPL235W	55.7889
YFR051C	YGL137W	55.7361
YDL150W	YJL011C	55.6268
YBR154C	YPR187W	55.5598
YDR145W	YML015C	55.4972
YLR291C	YOR260W	55.4315
YBR081C	YDR448W	55.3223
YDR308C	YOR174W	55.3009
YDR308C	YPR070W	55.2641
YJL011C	YOR207C	55.2283
YDR322W	YNL005C	55.2076
YBR095C	YIL084C	55.158
YJR007W	YOR260W	55.1401
YHR081W	YOL021C	55.1075
YBR103W	YGL194C	55.0787
YGR083C	YJR007W	55.0124
YBL046W	YML010W	54.9293
YHR119W	YLR015W	54.9199
YAL043C	YDR195W	54.8987
YFR010W	YHR027C	54.8912
YDR211W	YKR026C	54.8097
YDR303C	YFR037C	54.6778
YEL018W	YFL024C	54.6311
YDR145W	YPL011C	54.5734
YJL109C	YPL126W	54.5484
YBR081C	YDR176W	54.4676
YDR097C	YOL090W	54.4625
YGL011C	YOR157C	54.4559
YNL032W	YNL099C	54.3953
YML069W	YNL206C	54.3864
YER094C	YPR103W	54.3334
YDL134C	YDL188C	54.2939
YDR176W	YOL148C	54.2128
YEL056W	YMR284W	54.1364
YDR238C	YGL137W	54.1332
YDL087C	YIL061C	54.0811
YMR284W	YPL001W	53.9474
YGR186W	YJL140W	53.936
YDR175C	YGL129C	53.802
YBR154C	YIL021W	53.6929
YLR418C	YOR061W	53.667
YKR026C	YPL237W	53.546
YDR429C	YLR192C	53.5377
YBR095C	YNL330C	53.5249
YGL112C	YMR236W	53.5169
YCL011C	YDL084W	53.4709
YDL092W	YPR088C	53.4339
YDR301W	YKL018W	53.4323
YAR003W	YLR015W	53.4241
YDL150W	YKR025W	53.3998
YBR154C	YNL113W	53.3177
YGR156W	YJR093C	53.2988
YOR157C	YOR362C	53.2108
YHR172W	YNL126W	53.1483
YIL126W	YPR034W	53.1073
YDR176W	YPL254W	53.0897
YGR047C	YOR110W	53.0425
YBR103W	YKR029C	52.9033
YBR195C	YPR018W	52.892
YBR193C	YPR070W	52.891
YDL002C	YLR052W	52.869
YDR075W	YML010W	52.8431
YCR033W	YIL112W	52.7351
YER133W	YGR156W	52.6461
YDL216C	YOL117W	52.6238
YFR010W	YHR200W	52.6068
YDR303C	YMR091C	52.6027
YDL097C	YGR232W	52.5803
YMR125W	YPL178W	52.558
YKR038C	YML036W	52.5013
YDL126C	YJL115W	52.4296
YIL126W	YLR033W	52.4025
YIL033C	YJL164C	52.3876
YCR035C	YGR158C	52.3859
YIL115C	YJL061W	52.3782
YDL084W	YHR167W	52.349
YER022W	YPR070W	52.3441
YDR176W	YLR055C	52.3065
YGL112C	YPL254W	52.2346
YDR311W	YLR005W	52.2152
YIL035C	YML069W	52.2019
YBR081C	YLR055C	52.1804
YHR058C	YPR070W	52.1755
YOL038W	YPR103W	52.1716
YJR007W	YMR309C	52.1355
YDL132W	YIL046W	52.1256
YDR176W	YDR448W	52.0719
YOL142W	YOR076C	52.0541
YJR093C	YLR115W	52.0442
YDR211W	YJR007W	52.0325
YDL087C	YKL012W	52.0021
YDR176W	YDR392W	51.8561
YGL011C	YPR103W	51.8278
YGR074W	YLR275W	51.8093
YNL113W	YOR340C	51.7935
YHR172W	YLR212C	51.7474
YGR186W	YIL021W	51.734
YBR198C	YDR176W	51.6943
YLR115W	YPR107C	51.6732
YHR062C	YNL221C	51.5256
YDR240C	YIL061C	51.4972
YLL004W	YML065W	51.356
YDR280W	YOR076C	51.2973
YLR200W	YNL153C	51.2703
YDR235W	YKL012W	51.2638
YLL036C	YPL151C	51.2496
YML065W	YPR162C	51.1877
YDL047W	YFR040W	51.1863
YDR254W	YPL018W	51.1859
YIL115C	YJL041W	51.0699
YMR290C	YNL132W	51.0589
YLR005W	YPR056W	51.05
YGL227W	YIL017C	50.9788
YDR448W	YPL254W	50.976
YGR020C	YPR036W	50.9614
YDL150W	YNR003C	50.9087
YML062C	YNL139C	50.8667
YDL126C	YGL207W	50.7463
YGL105W	YGR264C	50.7344
YDR195W	YKL059C	50.7192
YBR103W	YCR033W	50.698
YDR301W	YGR156W	50.6372
YHR167W	YML062C	50.6083
YNL151C	YPR190C	50.5906
YKL135C	YPL259C	50.5872
YFR004W	YGR232W	50.5332
YEL018W	YPR023C	50.4828
YJL081C	YNL107W	50.3936
YHR081W	YOL142W	50.2952
YGL120C	YGL128C	50.2022
YDL002C	YFL013C	50.1907
YJL050W	YOL115W	50.1749
YGL207W	YMR284W	50.1747
YDR238C	YPL010W	50.0247
YDR235W	YIL061C	49.961
YGL112C	YLR055C	49.9351
YBR193C	YOR174W	49.8934
YDR416W	YLL036C	49.7553
YML092C	YPR103W	49.7344
YHR118C	YNL261W	49.6989
YJL011C	YPR110C	49.6787
YOR340C	YOR341W	49.6599
YBR198C	YMR236W	49.6546
YDR303C	YML127W	49.6074
YLR222C	YLR409C	49.5924
YPL259C	YPR029C	49.5201
YGR083C	YLR291C	49.5051
YPL243W	YPR088C	49.423
YLR384C	YPL101W	49.3707
YIL062C	YLR370C	49.3469
YJL081C	YOR141C	49.2669
YGR135W	YOR157C	49.1203
YJL148W	YOR340C	49.0862
YOL038W	YOR157C	49.0858
YAL001C	YDR362C	49.0746
YNL236W	YPR070W	49.0663
YBR198C	YML015C	49.0184
YDR074W	YML100W	49.004
YBR234C	YNR035C	48.9735
YBR081C	YGR252W	48.9315
YDR211W	YLR291C	48.9039
YDR362C	YOR110W	48.8846
YGL207W	YIL035C	48.8645
YMR314W	YPR103W	48.8617
YGR200C	YLR384C	48.8169
YGR083C	YPL237W	48.7815
YGR074W	YLR147C	48.7642
YGL150C	YOR141C	48.7431
YDR303C	YLR321C	48.6682
YHR013C	YOR253W	48.6629
YGL112C	YML015C	48.6354
YDL145C	YPL010W	48.6296
YIL061C	YLR275W	48.5967
YFL013C	YLR052W	48.5441
YMR125W	YNL189W	48.4814
YBR211C	YGR179C	48.4046
YDR255C	YIL017C	48.2878
YCL010C	YDR176W	48.2791
YDL084W	YNL253W	48.2436
YGL194C	YIL112W	48.204
YDL029W	YLR370C	48.1644
YDR473C	YKL173W	48.136
YJR093C	YPR107C	48.133
YJL011C	YNL151C	48.1299
YDL087C	YDR235W	48.0425
YDL047W	YJL098W	48.0046
YER027C	YGL115W	47.9503
YBR288C	YGR261C	47.8667
YER029C	YLR275W	47.8365
YML015C	YMR236W	47.7927
YLR117C	YPL151C	47.7604
YOR260W	YPL237W	47.7331
YGR253C	YPR103W	47.7188
YNL206C	YOL054W	47.6927
YDL092W	YML105C	47.6678
YHR200W	YOR117W	47.6667
YBR154C	YGR005C	47.6601
YFL022C	YLR060W	47.6313
YDL092W	YPL243W	47.5845
YKR025W	YPR190C	47.5549
YDR301W	YKL059C	47.5251
YJR007W	YLR215C	47.4824
YPR110C	YPR187W	47.4604
YBR193C	YDR308C	47.4532
YJL109C	YMR093W	47.4524
YDR195W	YKL018W	47.4299
YDR404C	YIL021W	47.4234
YFR010W	YOR117W	47.3716
YBR119W	YKL012W	47.3587
YKR025W	YNL151C	47.3143
YBR055C	YER172C	47.2781
YLR418C	YOR039W	47.2731
YGL227W	YMR135C	47.2578
YIL017C	YMR135C	47.2395
YIL034C	YKL007W	47.228
YGL019W	YGL244W	47.2277
YGR135W	YPR103W	47.2203
YMR033W	YNR023W	47.2164
YGR078C	YLR200W	47.176
YER172C	YPR178W	47.1473
YLL008W	YMR049C	47.1226
YDL087C	YDR240C	47.0501
YIL033C	YPL203W	47.029
YJL148W	YNL248C	47.0078
YML071C	YNL041C	46.9675
YAL034W-A	YPL233W	46.9285
YDR175C	YIL093C	46.9243
YDR303C	YPR034W	46.9208
YBR060C	YML065W	46.9175
YGL143C	YGL252C	46.9004
YGR232W	YOR261C	46.8998
YAL043C	YDR301W	46.8678
YBR289W	YJL176C	46.8666
YDR392W	YGL112C	46.7939
YML102W	YPR018W	46.7668
YDR303C	YLR357W	46.7618
YLR381W	YPL018W	46.759
YBR119W	YIL061C	46.7438
YLR085C	YPL235W	46.7362
YOL148C	YPL254W	46.736
YJR068W	YOR217W	46.715
YML049C	YMR288W	46.7079
YDR416W	YLR117C	46.7046
YBR288C	YPL195W	46.6393
YKL059C	YPR107C	46.6364
YHL025W	YNR023W	46.6261
YGR091W	YKL173W	46.5907
YDR195W	YGR156W	46.5685
YGR233C	YPL031C	46.5601
YHR069C	YHR081W	46.4825
YFR051C	YNL287W	46.459
YBR154C	YPR190C	46.4429
YGR253C	YOR157C	46.3948
YNL151C	YOR207C	46.2932
YJL070C	YML035C	46.2701
YML015C	YPL011C	46.2171
YNL113W	YNL248C	46.1923
YDR308C	YHR058C	46.1677
YDL147W	YGR232W	46.1216
YDR303C	YMR033W	46.0684
YDR167W	YGL112C	46.0175
YNL097C	YNL330C	45.9745
YJL011C	YKR025W	45.9574
YDR254W	YLR381W	45.9517
YDR392W	YLR055C	45.938
YKR002W	YPR107C	45.9036
YGR232W	YIL075C	45.8552
YIL021W	YOL005C	45.8497
YER021W	YGR232W	45.8007
YDR145W	YDR176W	45.7955
YDL126C	YLL022C	45.7941
YBR126C	YML100W	45.7921
YML105C	YPR088C	45.7779
YDR092W	YGL087C	45.7475
YDR211W	YPL237W	45.6922
YPL086C	YPL101W	45.6735
YNR010W	YOR174W	45.6704
YLR421C	YOR261C	45.6697
YDR469W	YPL138C	45.6695
YGR104C	YHR041C	45.6478
YAL043C	YJR093C	45.6364
YFL039C	YNL107W	45.5852
YGR285C	YHR064C	45.5628
YDL116W	YGL092W	45.5622
YIL112W	YKR029C	45.5218
YBL058W	YDL126C	45.5153
YGL112C	YOL148C	45.5033
YDR240C	YKL012W	45.3913
YLR117C	YMR213W	45.3807
YBR126C	YDR074W	45.3718
YJR093C	YKL018W	45.3478
YBR193C	YNR010W	45.3365
YBL041W	YOR157C	45.2693
YDR137W	YLR039C	45.1882
YDR176W	YGL066W	45.1867
YGR091W	YPR178W	45.1619
YDR145W	YDR167W	45.138
YDR301W	YJR093C	45.1334
YOL145C	YOR123C	45.1018
YGR186W	YOR151C	45.0975
YER012W	YPR103W	45.0792
YCR009C	YDR388W	45.0518
YBR198C	YPL011C	45.0338
YNR010W	YPR070W	45.0216
YDR392W	YOL148C	45.0164
YBR095C	YPL139C	44.9844
YGL070C	YIL021W	44.921
YDR179C	YMR025W	44.8617
YKR025W	YPR110C	44.7972
YDR359C	YJL081C	44.7318
YIL103W	YKL191W	44.698
YBR095C	YNL097C	44.6532
YDR392W	YPL254W	44.6086
YNL287W	YPL010W	44.5881
YBR170C	YGR048W	44.5753
YBR060C	YHR118C	44.568
YGL112C	YPL011C	44.5578
YCR057C	YJL069C	44.5499
YKL135C	YPR029C	44.5399
YDL029W	YIL062C	44.5286
YHR085W	YHR197W	44.5143
YAL001C	YOR110W	44.4927
YAL001C	YBR123C	44.4568
YDR392W	YDR448W	44.4444
YBR211C	YLR315W	44.3852
YDR201W	YKR037C	44.37
YBR154C	YPR010C	44.3127
YDR359C	YOR244W	44.3038
YBR154C	YNR003C	44.2818
YDR255C	YMR135C	44.2459
YBR105C	YIL097W	44.2446
YLR277C	YPR107C	44.2348
YDR359C	YGR002C	44.2309
YDR477W	YER027C	44.2189
YEL015W	YNL118C	44.1619
YDR448W	YOL148C	44.1615
YLR440C	YNL258C	44.1524
YDL150W	YOR116C	44.139
YBR079C	YLR192C	44.1328
YGL151W	YPR070W	44.1327
YGL092W	YJR042W	44.1286
YJL140W	YOL005C	44.1088
YDL084W	YML062C	44.0967
YCR035C	YHR081W	44.0496
YBR198C	YGR274C	44.0458
YOR308C	YPR178W	43.8665
YDL116W	YJR042W	43.8537
YDL150W	YNL151C	43.8523
YOR116C	YPR190C	43.8337
YLR423C	YPR049C	43.8306
YER029C	YGR074W	43.8209
YDR469W	YHR119W	43.7365
YKL012W	YLR275W	43.718
YBR123C	YDR362C	43.716
YDR175C	YHL004W	43.6695
YDL074C	YPL055C	43.6544
YDL132W	YJR090C	43.641
YBR055C	YOR308C	43.5115
YFR004W	YLR421C	43.4721
YNL030W	YNL206C	43.4444
YHL025W	YJL176C	43.4444
YBL035C	YNL102W	43.3842
YDR195W	YER133W	43.3803
YGR086C	YPL004C	43.3533
YFL013C	YGL150C	43.3405
YJR093C	YNL317W	43.3353
YIL084C	YMR263W	43.2202
YLR384C	YPL086C	43.216
YBR081C	YOL148C	43.1967
YDL014W	YOR206W	43.1683
YDL003W	YFL008W	43.1535
YLL036C	YMR213W	43.1512
YGL115W	YGL208W	43.1346
YDR392W	YGR252W	43.0729
YKL173W	YOR308C	43.0591
YCL011C	YHR167W	43.0078
YHR058C	YOR174W	43.0009
YDR308C	YNR010W	42.9861
YBR234C	YIL062C	42.975
YBR193C	YGL151W	42.9747
YIL084C	YOL004W	42.9478
YGL244W	YIL035C	42.9386
YBR279W	YIL035C	42.9288
YBR229C	YDR221W	42.8386
YNL330C	YPL181W	42.8176
YDR308C	YOL051W	42.7831
YHR119W	YPL138C	42.7317
YNL021W	YPR179C	42.7244
YHR058C	YNR010W	42.696
YGR232W	YPR108W	42.6937
YCL031C	YIL035C	42.6897
YDR303C	YIL126W	42.6414
YDR472W	YOR115C	42.6194
YBR198C	YLR055C	42.6169
YBR119W	YGR013W	42.6128
YER022W	YNR010W	42.6039
YGR084C	YHL004W	42.6001
YBR081C	YCL010C	42.5182
YHR086W	YML046W	42.5153
YBR193C	YHR041C	42.506
YAR007C	YJL173C	42.4881
YJL148W	YPR010C	42.483
YGR005C	YOL005C	42.4235
YOR244W	YPR023C	42.3493
YAL021C	YCR093W	42.3459
YLR200W	YML094W	42.3413
YDR240C	YHR086W	42.3347
YDR301W	YER133W	42.3126
YGR017W	YKR048C	42.257
YAR002C-A	YGL200C	42.2237
YFL024C	YHR090C	42.2221
YGR095C	YOR076C	42.2094
YDL087C	YHR086W	42.1859
YIL126W	YML127W	42.1816
YER025W	YOR260W	42.1682
YGL241W	YLR133W	42.1547
YJL176C	YMR033W	42.1516
YML092C	YOR157C	42.1399
YGL070C	YOL005C	42.1347
YEL015W	YOL149W	42.0826
YHR012W	YJL154C	42.0561
YDL100C	YER083C	42.0426
YBR251W	YGR165W	42.0388
YML015C	YMR005W	42.0314
YFR015C	YLR258W	42.0201
YLR330W	YMR237W	42.0055
YER022W	YHR058C	41.9979
YGR056W	YLR033W	41.9509
YDR145W	YMR005W	41.9373
YBR119W	YML046W	41.9279
YDR179C	YIL071C	41.8632
YJL115W	YMR284W	41.8309
YJL011C	YNR003C	41.8013
YGL066W	YGL112C	41.7922
YBL093C	YDR308C	41.762
YFL049W	YNR023W	41.7495
YGL066W	YPL254W	41.6886
YJL173C	YNL312W	41.6867
YBR081C	YBR198C	41.6729
YDL216C	YIL071C	41.6229
YCL011C	YNL253W	41.5706
YDR235W	YHR086W	41.5421
YNL097C	YPL139C	41.5287
YGR158C	YNL232W	41.4806
YML105C	YPL243W	41.4796
YCL010C	YPL254W	41.4738
YDL147W	YLR421C	41.4275
YKR025W	YOR116C	41.3908
YIL079C	YOL115W	41.3503
YCR095C	YHL029C	41.3405
YDR469W	YLR015W	41.2987
YGL200C	YML012W	41.2593
YGR002C	YPR023C	41.2438
YAL043C	YPR107C	41.2343
YDL002C	YDR190C	41.1932
YNL118C	YOL149W	41.1722
YDR175C	YGR165W	41.1132
YGL151W	YGR104C	41.1088
YDL140C	YJL140W	41.1083
YBR251W	YHL004W	40.9859
YEL053C	YPR051W	40.9718
YGR232W	YKL145W	40.9568
YJR093C	YLR277C	40.9534
YOR151C	YPL225W	40.9442
YGR158C	YOL021C	40.9299
YDL216C	YDR179C	40.9156
YPL139C	YPL181W	40.8673
YDR318W	YLR381W	40.8602
YBR198C	YGR252W	40.8289
YDR347W	YIL093C	40.7521
YGL213C	YLR398C	40.6237
YJL069C	YLR409C	40.623
YDR195W	YLR277C	40.6177
YKL012W	YLR147C	40.6032
YHL004W	YNL137C	40.5824
YGR158C	YOR076C	40.5775
YGR156W	YPR107C	40.5684
YDL030W	YJL203W	40.5366
YGL151W	YLR071C	40.525
YDR195W	YKR002W	40.511
YGR186W	YOL005C	40.4519
YBR154C	YDR404C	40.434
YGR158C	YOR001W	40.4192
YIL071C	YOL117W	40.4147
YGL019W	YML069W	40.3925
YGR104C	YOR174W	40.3775
YHR086W	YIL061C	40.3449
YBR143C	YDR172W	40.336
YGR074W	YIL061C	40.3097
YGL112C	YMR005W	40.2993
YDR166C	YER008C	40.2871
YJR007W	YPR041W	40.262
YHL004W	YJR113C	40.2215
YDL003W	YJL074C	40.2198
YCL010C	YGR252W	40.1691
YDR195W	YLR115W	40.1466
YBR289W	YNR023W	40.1463
YGR056W	YKR008W	40.1198
YDR152W	YGR173W	40.1186
YDR301W	YPR107C	40.0991
YLL022C	YMR284W	40.0899
YDL007W	YFR010W	40.0644
YOR210W	YPR187W	40.0481
YBR055C	YHR165C	40.0468
YJL001W	YOR157C	40.0385
YOL004W	YPL181W	40.0278
YER172C	YGR091W	40.0267
YDR254W	YDR318W	40.0238
YMR192W	YPL249C	40.0216
YGR086C	YKL142W	39.9942
YBR198C	YDR167W	39.9926
YLR291C	YPL237W	39.9891
YGL244W	YOR061W	39.9847
YGR252W	YOL148C	39.9713
YCL010C	YLR055C	39.9647
YGL025C	YHR058C	39.9496
YDR362C	YPL007C	39.9372
YJL081C	YLR052W	39.928
YDL201W	YDR165W	39.9218
YER025W	YGR083C	39.8905
YIL093C	YJR113C	39.8746
YDL185W	YGR020C	39.8636
YGL106W	YOR326W	39.8599
YLR197W	YPR137W	39.8564
YGL025C	YNR010W	39.8377
YER111C	YLR182W	39.8194
YGL043W	YGR005C	39.8096
YBR081C	YGL066W	39.7917
YDR427W	YLR421C	39.7641
YLR335W	YNL189W	39.7626
YEL018W	YGR002C	39.7585
YDR308C	YER022W	39.7291
YDR179C	YOL117W	39.7079
YNL189W	YPL178W	39.6955
YMR240C	YMR288W	39.6602
YGL246C	YOR048C	39.6424
YBR193C	YHR058C	39.64
YBR211C	YJR135C	39.6317
YLR055C	YPL254W	39.6203
YOR038C	YPL153C	39.578
YKR025W	YNR003C	39.5704
YMR290C	YOR063W	39.5484
YNL151C	YPR110C	39.5401
YGR013W	YHR086W	39.5353
YER025W	YKR026C	39.5326
YHR165C	YPR178W	39.53
YHR081W	YNL232W	39.5287
YOL051W	YPR070W	39.5277
YBR109C	YGL106W	39.5074
YHR165C	YLR275W	39.5049
YIL071C	YMR025W	39.5044
YGR103W	YLL008W	39.5029
YCL010C	YGL112C	39.4326
YOR116C	YPR110C	39.4279
YBL026W	YJL124C	39.4155
YFL049W	YJL176C	39.4084
YJL148W	YPR110C	39.392
YGR261C	YPL195W	39.3246
YDR449C	YLR409C	39.3178
YDR280W	YGR158C	39.3069
YKL144C	YOR207C	39.2374
YBR234C	YDL029W	39.2337
YAL001C	YGR047C	39.1939
YAR002C-A	YML012W	39.1865
YDL005C	YOL051W	39.184
YJR113C	YNL306W	39.1493
YGL025C	YOL051W	39.1485
YGL194C	YKR029C	39.0854
YDR477W	YGL208W	39.0835
YDL005C	YDR308C	39.0776
YDR267C	YIL128W	39.0693
YNL317W	YPR107C	39.0553
YEL018W	YNL107W	39.0547
YDR308C	YGL025C	39.0354
YDL002C	YJL081C	38.9965
YBR123C	YOR110W	38.9763
YGL150C	YLR052W	38.9495
YLR418C	YML069W	38.9492
YGR132C	YGR231C	38.9361
YER094C	YOR157C	38.933
YPL210C	YPR088C	38.902
YER133W	YKL193C	38.8889
YGL207W	YKR048C	38.8742
YIL084C	YPL181W	38.8437
YDR201W	YGL061C	38.8407
YLR421C	YOR259C	38.8264
YNL251C	YPL190C	38.8178
YHR086W	YKL012W	38.7879
YML065W	YNL261W	38.7787
YAL021C	YNR052C	38.7628
YCR033W	YKR029C	38.7181
YOL094C	YOR217W	38.6904
YDL175C	YJL050W	38.6901
YDL116W	YKL057C	38.6636
YGL070C	YJL140W	38.6602
YDR359C	YPR023C	38.6042
YGR252W	YPL254W	38.5732
YDL216C	YMR025W	38.5584
YJL124C	YPR178W	38.5518
YER007C-A	YJR014W	38.5489
YLR423C	YPL166W	38.5172
YLR129W	YLR409C	38.4986
YPL210C	YPL243W	38.4969
YDL160C	YJL124C	38.4945
YDR362C	YGR047C	38.4527
YAL013W	YNL097C	38.3871
YLR292C	YOR254C	38.3671
YER136W	YLR262C	38.3318
YDL111C	YGR158C	38.3073
YBR251W	YGL129C	38.2558
YGL145W	YNL258C	38.2505
YGL145W	YLR440C	38.2505
YGL005C	YML071C	38.2469
YHR174W	YKL060C	38.2189
YER092W	YLR052W	38.199
YDR195W	YNL317W	38.169
YFL039C	YGR002C	38.1152
YEL056W	YGL252C	38.0652
YGR195W	YHR081W	38.0407
YHR024C	YLR163C	38.0317
YCL039W	YMR135C	38.009
YCR057C	YLR409C	38.0058
YDL092W	YPL210C	37.9992
YCL010C	YDR392W	37.9749
YAL013W	YBR095C	37.9408
YGL252C	YPL001W	37.8973
YER092W	YOR141C	37.8937
YDR145W	YLR055C	37.8905
YGR232W	YHR027C	37.8661
YBR017C	YGL122C	37.8345
YDL132W	YDL164C	37.8335
YNR010W	YOL051W	37.8318
YDR145W	YGR252W	37.8228
YDR308C	YPR168W	37.816
YGL061C	YKR037C	37.8066
YNR003C	YOR116C	37.8001
YBR081C	YDR145W	37.7905
YDR322W	YMR024W	37.7764
YBR119W	YDL087C	37.7478
YGR158C	YGR195W	37.6621
YGR074W	YKL012W	37.6585
YGL223C	YML071C	37.6447
YGL129C	YKL155C	37.5776
YGR084C	YNL137C	37.5555
YDR255C	YGL227W	37.5417
YDL132W	YDR054C	37.506
YBR198C	YPL254W	37.4753
YBR193C	YGR104C	37.4638
YGL150C	YPL235W	37.4584
YFR050C	YPR103W	37.4303
YBR198C	YMR005W	37.4298
YNL113W	YPR010C	37.397
YGL112C	YGR274C	37.3891
YGR158C	YOL142W	37.3875
YAL033W	YHR062C	37.3875
YMR025W	YOL117W	37.354
YNL236W	YOR174W	37.3344
YDR353W	YHR106W	37.3212
YGL092W	YKL057C	37.2833
YER125W	YOR138C	37.2683
YER029C	YHR165C	37.2109
YNL104C	YOR108W	37.2097
YDR237W	YDR322W	37.1467
YHR041C	YLR071C	37.134
YAL016W	YML109W	37.1221
YEL003W	YNL153C	37.0924
YIL142W	YJL014W	37.0895
YAL038W	YPL106C	37.0874
YGL207W	YGL252C	37.0845
YGL048C	YGR232W	37.0781
YDR099W	YER177W	37.0778
YBR211C	YDR383C	37.0675
YDR175C	YKL155C	37.0674
YGR104C	YLR071C	37.0403
YNL107W	YPR023C	37.021
YDR118W	YLR127C	37.0008
YBR154C	YOR210W	36.9999
YER112W	YJL124C	36.9731
YGR120C	YPR105C	36.9501
YIL093C	YPL118W	36.9329
YBR154C	YOR151C	36.906
YCR052W	YGR056W	36.8566
YLR148W	YMR231W	36.8417
YDR404C	YGR186W	36.8201
YLR170C	YPL259C	36.817
YDR473C	YOR308C	36.8091
YOL076W	YPR131C	36.7816
YDR254W	YJR135C	36.7752
YBR171W	YOR254C	36.759
YBR171W	YLR292C	36.759
YHR200W	YLR421C	36.757
YGL129C	YHL004W	36.7157
YFL049W	YMR033W	36.6978
YBR198C	YMR227C	36.6591
YCL031C	YOR061W	36.6379
YBR087W	YOR217W	36.6347
YMR005W	YPL011C	36.6031
YIL061C	YLR147C	36.5663
YCR077C	YER112W	36.5639
YCR012W	YKL060C	36.5396
YDR448W	YGL066W	36.4875
YER029C	YKL173W	36.458
YGL129C	YNL137C	36.4222
YKL022C	YLR127C	36.4156
YGL129C	YIL093C	36.4005
YGL070C	YGR005C	36.375
YER081W	YIL074C	36.3588
YDR175C	YNL137C	36.3513
YDL030W	YMR288W	36.3074
YFL024C	YFL039C	36.3006
YFR052W	YLR421C	36.2576
YAR003W	YDR469W	36.2287
YDR145W	YGL066W	36.21
YBR251W	YIL093C	36.2035
YJL176C	YOR290C	36.1818
YLR421C	YPR108W	36.1736
YDL100C	YGL020C	36.1067
YDR001C	YER177W	36.1045
YOL051W	YOR174W	36.0827
YLL045C	YMR290C	36.0533
YBR251W	YGR084C	36.0496
YGL112C	YMR223W	36.0199
YGL066W	YGR252W	35.9662
YLR170C	YPR029C	35.9356
YHR166C	YKL022C	35.9132
YBR170C	YDL126C	35.9023
YIL033C	YKL166C	35.7984
YDL030W	YMR240C	35.7976
YBR211C	YDR254W	35.7879
YBR126C	YMR261C	35.7407
YDR175C	YJR113C	35.7256
YAL033W	YNL221C	35.6929
YCL010C	YDR448W	35.685
YBR081C	YDR392W	35.6815
YEL018W	YOR244W	35.6481
YER029C	YKL012W	35.6275
YAL013W	YPL181W	35.6056
YHL004W	YIL093C	35.5928
YDL002C	YGL150C	35.5834
YBR279W	YOR061W	35.5712
YBR105C	YGL227W	35.5458
YBR198C	YOL148C	35.5454
YBR079C	YOR361C	35.543
YDR303C	YGR056W	35.5365
YGL092W	YGL100W	35.5201
YAL013W	YIL084C	35.5173
YER008C	YLR166C	35.5129
YDR166C	YLR166C	35.5129
YMR309C	YOR361C	35.5085
YHR085W	YNL182C	35.5007
YGR071C	YLR373C	35.4866
YCL039W	YIL097W	35.4799
YER029C	YPR178W	35.4774
YGR252W	YMR223W	35.4718
YHR041C	YOR174W	35.4698
YDL126C	YOL054W	35.4533
YML069W	YMR284W	35.3809
YGL137W	YPL010W	35.3781
YJL006C	YML112W	35.3646
YOR110W	YPL007C	35.3629
YOR224C	YPR110C	35.3516
YGR232W	YOR117W	35.301
YDL002C	YPL235W	35.2816
YDR337W	YJR113C	35.2549
YBR146W	YPL118W	35.2178
YBR253W	YER022W	35.2157
YAL001C	YPL007C	35.2092
YBR123C	YPL007C	35.173
YER136W	YOR089C	35.1484
YBL084C	YHR166C	35.1064
YGR095C	YHR081W	35.0888
YDR145W	YGR274C	35.0493
YNL248C	YOR341W	35.0439
YGL137W	YNL287W	35.0315
YBR119W	YDR235W	35.012
YGL005C	YGL223C	34.995
YDR145W	YDR448W	34.9933
YDL005C	YGL127C	34.9913
YHL025W	YMR033W	34.9887
YML105C	YPL210C	34.9678
YDR188W	YIL142W	34.9656
YDR245W	YJL183W	34.9304
YCL010C	YOL148C	34.9137
YDR211W	YER025W	34.9102
YDL188C	YOR014W	34.899
YFR050C	YOR157C	34.8929
YHR187W	YPL101W	34.891
YER008C	YIL068C	34.8867
YDR166C	YIL068C	34.8867
YMR005W	YMR236W	34.8344
YBR279W	YGL019W	34.82
YER021W	YLR421C	34.8187
YGL190C	YML109W	34.8084
YGL066W	YLR055C	34.8038
YLR055C	YOL148C	34.8023
YGL130W	YPL228W	34.7934
YBR193C	YOL051W	34.7683
YAR003W	YHR119W	34.7679
YDL126C	YGR048W	34.7557
YFR037C	YGR056W	34.7129
YIL093C	YNL306W	34.6614
YJR007W	YLR291C	34.5365
YGR091W	YOR308C	34.5285
YNL151C	YNR003C	34.5189
YBR154C	YGR186W	34.5074
YKL144C	YKR025W	34.5014
YIL035C	YOL145C	34.5008
YDL185W	YPR036W	34.4849
YDL140C	YOL005C	34.4612
YDR318W	YPL018W	34.4527
YBR251W	YNL137C	34.4271
YGR104C	YPR070W	34.4247
YNL097C	YOL004W	34.4085
YBL093C	YGL025C	34.4037
YLR398C	YPR189W	34.3977
YNL113W	YNR003C	34.3761
YHR165C	YLR147C	34.3722
YAR019C	YBR281C	34.3565
YGR158C	YHR069C	34.353
YLR071C	YPR070W	34.3415
YDL108W	YPR056W	34.3329
YBR146W	YHL004W	34.328
YER092W	YGL150C	34.3215
YDR473C	YGR091W	34.3212
YGR165W	YNL137C	34.3008
YDL140C	YGR005C	34.2234
YGL100W	YJR042W	34.2069
YGL044C	YMR061W	34.149
YHL002W	YNR006W	34.1399
YGR274C	YMR005W	34.131
YKL144C	YPR190C	34.0793
YGL129C	YGR084C	34.0724
YBR146W	YIL093C	34.0556
YBR245C	YLR095C	34.0536
YBR146W	YNL137C	34.0516
YGR156W	YNL222W	34.0353
YDR359C	YEL018W	34.0185
YGR017W	YGR159C	33.9765
YDR383C	YLR315W	33.9663
YGL025C	YPR070W	33.9503
YHR118C	YML065W	33.9275
YDR322W	YNL252C	33.9239
YDR175C	YGR084C	33.9239
YGL066W	YOL148C	33.9089
YBL093C	YOR174W	33.8976
YBR234C	YLR370C	33.879
YJL148W	YNL113W	33.8682
YDR176W	YMR223W	33.8643
YGL244W	YOR039W	33.848
YBL093C	YNR010W	33.8314
YDL111C	YHR081W	33.8309
YDL007W	YLR421C	33.7991
YGR232W	YOR259C	33.7905
YBR253W	YOR174W	33.7833
YBR123C	YGR047C	33.7804
YKL122C	YPR088C	33.7785
YLL008W	YOR272W	33.7739
YAL029C	YGL106W	33.7725
YLR396C	YMR231W	33.7556
YLR148W	YLR396C	33.7556
YGL252C	YJL115W	33.7498
YGR084C	YJR113C	33.7487
YOL145C	YOR061W	33.7471
YDL175C	YIL079C	33.7303
YDR347W	YPL118W	33.7277
YDL116W	YGL100W	33.6864
YJR113C	YMR188C	33.6607
YGL129C	YPL118W	33.6269
YDR337W	YNL306W	33.6161
YBL093C	YOL051W	33.6116
YDR295C	YNL021W	33.6016
YBR231C	YDR190C	33.5959
YAL034W-A	YIR010W	33.5728
YER133W	YJR093C	33.5551
YBL093C	YDL005C	33.45
YFL049W	YHL025W	33.419
YBR198C	YDR392W	33.3948
YJR112W	YPL233W	33.3868
YDR347W	YKL155C	33.3622
YER025W	YPL237W	33.3588
YML095C	YPL022W	33.3525
YGL005C	YNL041C	33.3399
YHL004W	YPL118W	33.3352
YBR105C	YMR135C	33.313
YMR231W	YPL045W	33.3061
YLR148W	YPL045W	33.3061
YDR473C	YHR165C	33.263
YJR113C	YPL118W	33.2132
YKL190W	YML057W	33.1798
YBR198C	YDR448W	33.1379
YDR190C	YLR052W	33.1334
YBR193C	YLR071C	33.1274
YGR056W	YML127W	33.1046
YDR448W	YLR055C	33.085
YCL011C	YML062C	33.0567
YER172C	YLR147C	33.0552
YNL137C	YPL118W	33.0538
YDR145W	YMR227C	33.0439
YLR270W	YOR173W	33.0404
YDR404C	YOR151C	33.0322
YDR195W	YJR093C	33.0182
YDR041W	YPL118W	33.0157
YJR113C	YKL155C	33.013
YDL126C	YML069W	33.0092
YDL040C	YHR013C	33.0057
YER029C	YML046W	33.0009
YJR063W	YOR341W	32.9981
YBL026W	YPR178W	32.9838
YDL140C	YGR186W	32.9659
YDL115C	YJL140W	32.9575
YGL223C	YNL041C	32.9487
YFL013C	YJL081C	32.9331
YBR135W	YBR160W	32.9054
YJL081C	YLR085C	32.882
YAL011W	YDR485C	32.8789
YLR335W	YLR347C	32.8702
YDR175C	YDR347W	32.8685
YDR175C	YPL118W	32.853
YDR359C	YNL107W	32.8415
YMR075W	YOL004W	32.8216
YDL002C	YER092W	32.8174
YMR263W	YPL181W	32.8171
YKR027W	YMR237W	32.8155
YHR058C	YOL051W	32.8081
YFL013C	YPL235W	32.8019
YGR244C	YOR142W	32.7983
YLL045C	YNL132W	32.771
YGL151W	YOR174W	32.7592
YDR347W	YJR113C	32.7194
YEL003W	YGR078C	32.6929
YGR128C	YJL109C	32.6732
YJR135C	YLR381W	32.6712
YGR179C	YJR135C	32.6335
YGL190C	YMR273C	32.6234
YGL129C	YJR113C	32.6193
YLR117C	YPR101W	32.6139
YBR289W	YOR290C	32.5672
YER157W	YGR120C	32.5646
YDL126C	YNL030W	32.5299
YGL019W	YGR090W	32.494
YBR253W	YHR058C	32.4862
YCL008C	YLR119W	32.4633
YML071C	YNL051W	32.4625
YLR052W	YPL235W	32.4425
YFL039C	YOR141C	32.4344
YBR198C	YGL066W	32.4254
YLR044C	YOL086C	32.4136
YMR290C	YPL198W	32.4124
YDL092W	YKL122C	32.4124
YKR048C	YLR133W	32.394
YKR025W	YNL113W	32.3905
YBR231C	YPL235W	32.3841
YOR069W	YOR132W	32.3804
YER031C	YER136W	32.3584
YIL161W	YNL023C	32.3527
YDR422C	YGL115W	32.3501
YNL097C	YPL181W	32.3264
YMR146C	YOR361C	32.3048
YGL049C	YGR162W	32.2834
YGR179C	YLR315W	32.2771
YBR154C	YDL150W	32.2549
YDL140C	YOR151C	32.2258
YER018C	YIL144W	32.1886
YHR187W	YLR384C	32.187
YKL173W	YLR147C	32.161
YOR210W	YPR110C	32.1076
YFR010W	YGR232W	32.107
YAL013W	YPL139C	32.0317
YMR263W	YNL097C	32.0264
YGL025C	YPR168W	32.0195
YDR318W	YGR179C	32.0025
YBR211C	YDR318W	31.9771
YJL138C	YKR059W	31.975
YDR041W	YJR113C	31.9492
YDR337W	YKL155C	31.9401
YML071C	YPR105C	31.9312
YAL043C	YOR179C	31.918
YGR056W	YMR091C	31.9174
YGR274C	YMR227C	31.9159
YDR041W	YHL004W	31.9075
YAL013W	YNL330C	31.8746
YGR233C	YOL001W	31.8711
YBL035C	YDL132W	31.8548
YDR394W	YLR421C	31.8547
YBR079C	YJR007W	31.8499
YKR027W	YLR330W	31.8293
YNR023W	YPL016W	31.8025
YDL115C	YOL005C	31.7933
YDR245W	YEL036C	31.7773
YLR113W	YLR248W	31.7757
YGR056W	YLR321C	31.7693
YKL018W	YPR107C	31.7646
YDR145W	YDR392W	31.7063
YHR165C	YOR308C	31.696
YGL112C	YMR227C	31.691
YBR221C	YER178W	31.6732
YDR337W	YDR347W	31.6507
YLR421C	YOR117W	31.6407
YIR010W	YPL233W	31.6291
YCR081W	YDR443C	31.6062
YNR023W	YOR290C	31.5879
YNL132W	YPL198W	31.555
YDR322W	YLR439W	31.542
YDR156W	YOR340C	31.5236
YBR279W	YOR039W	31.5118
YOR210W	YOR224C	31.4877
YHR041C	YPR070W	31.4872
YGR165W	YPL118W	31.4752
YER157W	YPR105C	31.4593
YBR253W	YPR070W	31.4474
YLR439W	YMR024W	31.3782
YDR394W	YGR232W	31.3558
YER029C	YIL061C	31.3536
YDR145W	YHL047C	31.3426
YDR308C	YGL127C	31.3309
YDL150W	YKL144C	31.319
YER090W	YKL211C	31.3165
YCL039W	YGL227W	31.3158
YDL115C	YIL021W	31.285
YJL183W	YPL050C	31.2827
YER018C	YMR117C	31.2689
YBR245C	YFR013W	31.2656
YBL093C	YHR058C	31.2606
YDR337W	YHL004W	31.2493
YDR245W	YPL050C	31.2403
YGL025C	YOR174W	31.2253
YGL092W	YLR208W	31.222
YNL151C	YOR116C	31.221
YHL047C	YML015C	31.2074
YCL010C	YGL066W	31.2039
YDL140C	YGL070C	31.178
YDL005C	YGL025C	31.1755
YDR337W	YIL093C	31.1558
YGR074W	YHR165C	31.1497
YAL035W	YMR146C	31.1318
YHR167W	YNL139C	31.1284
YDL084W	YNL139C	31.0631
YKL155C	YNL306W	31.0556
YKL018W	YLR015W	31.0519
YOL004W	YPR023C	31.0283
YBR160W	YDL155W	31.0255
YBR211C	YPL018W	30.9949
YGR056W	YIL126W	30.9887
YNL113W	YPR190C	30.96
YAL043C	YNL222W	30.954
YMR188C	YNL306W	30.9469
YDR071C	YER089C	30.9404
YHR165C	YML049C	30.9284
YCR020C-A	YPR051W	30.9012
YER172C	YLR275W	30.894
YML069W	YOR061W	30.8933
YJL011C	YNL113W	30.8927
YGL120C	YLR424W	30.8613
YER029C	YOR308C	30.851
YGL246C	YOR151C	30.8365
YOR174W	YPR168W	30.818
YHR058C	YMR112C	30.8125
YGL127C	YOL051W	30.8071
YGL070C	YGR186W	30.7983
YGR056W	YLR357W	30.7802
YGL129C	YNL306W	30.7603
YDL102W	YJR006W	30.7454
YDR175C	YNL306W	30.7256
YJL081C	YOR244W	30.7239
YBR154C	YOL005C	30.7068
YDR347W	YNL306W	30.6953
YBR251W	YDR347W	30.6942
YLR396C	YPL045W	30.6766
YGR274C	YPL011C	30.6619
YDL005C	YNR010W	30.6619
YMR227C	YPL011C	30.6392
YDR201W	YGR113W	30.6293
YGR095C	YGR158C	30.6192
YAL035W	YMR309C	30.612
YHR058C	YPR168W	30.6118
YGR091W	YHR165C	30.6038
YNR010W	YPR168W	30.5988
YOL005C	YOR151C	30.5889
YDR167W	YDR176W	30.5728
YIL093C	YJR101W	30.5609
YAL016W	YMR273C	30.5452
YBR253W	YGL151W	30.5315
YBR095C	YOL004W	30.5276
YGL129C	YGR165W	30.5234
YDR074W	YMR261C	30.5214
YGR084C	YPL118W	30.5016
YGR056W	YPR034W	30.4999
YCR020C-A	YEL053C	30.4957
YGR090W	YOR061W	30.4761
YBR109C	YOR326W	30.453
YEL051W	YPR036W	30.4455
YMR106C	YMR284W	30.4046
YBR146W	YGL129C	30.4013
YNL085W	YOR038C	30.3748
YLR439W	YNL005C	30.365
YGL128C	YKR022C	30.3614
YDR190C	YFL013C	30.3594
YJR042W	YLR208W	30.3565
YBR146W	YDR347W	30.3355
YJL125C	YNL062C	30.3178
YDR254W	YGR179C	30.2931
YBR253W	YHR041C	30.2558
YOL001W	YPL031C	30.222
YJR042W	YKL057C	30.2007
YEL003W	YML094W	30.1998
YBR251W	YPL118W	30.1997
YDR378C	YPR178W	30.1783
YDL156W	YGL207W	30.1503
YAL003W	YKL081W	30.1251
YBR146W	YDR175C	30.121
YBR146W	YJR113C	30.1105
YER071C	YIR003W	30.1046
YGL019W	YOL145C	30.1
YER172C	YOR308C	30.0937
YAL035W	YPR041W	30.0798
YBR154C	YDL140C	30.0529
YIL068C	YLR166C	30.0363
YBR119W	YHR086W	30.0237
YDR121W	YPR175W	30.017
YJL203W	YMR288W	30.0115
YKL135C	YLR170C	30.0061
YIL021W	YOR224C	29.9575
YKL155C	YPL118W	29.9538
YBR193C	YGL025C	29.951
YAL011W	YDR190C	29.9353
YDR145W	YOL148C	29.916
YHR027C	YLR421C	29.9133
YMR314W	YOR157C	29.9125
YBR102C	YER008C	29.9113
YBR102C	YDR166C	29.9113
YGL100W	YLR208W	29.9069
YDR392W	YMR223W	29.8951
YAL013W	YMR263W	29.8842
YDL014W	YNL132W	29.8726
YGR113W	YKR037C	29.8625
YGL066W	YMR236W	29.8336
YDR383C	YGR179C	29.8125
YDL140C	YDR404C	29.8123
YDR347W	YGR084C	29.8059
YKL142W	YPL004C	29.7966
YDR489W	YJL072C	29.7905
YDR013W	YJL072C	29.7905
YDR013W	YDR489W	29.7905
YDL195W	YLR208W	29.7719
YNL262W	YPR175W	29.7567
YGR056W	YMR033W	29.7558
YER025W	YLR291C	29.7497
YMR263W	YOL004W	29.6918
YGL100W	YKL057C	29.6615
YDR145W	YPL254W	29.6528
YGR047C	YPL007C	29.6457
YGR232W	YHR200W	29.6429
YGL048C	YLR421C	29.6056
YDR416W	YMR213W	29.6022
YJL011C	YKL144C	29.5996
YLR071C	YOR174W	29.5926
YJL124C	YKL188C	29.5923
YNL290W	YOR217W	29.5841
YBR154C	YDL115C	29.5804
YDL147W	YDL216C	29.5658
YER178W	YNL071W	29.5473
YBL026W	YDR378C	29.5316
YGR275W	YPR034W	29.526
YEL051W	YGR020C	29.5232
YGR084C	YIL093C	29.4994
YBR193C	YBR253W	29.4753
YBL093C	YPR070W	29.4741
YGR165W	YIL093C	29.4739
YIL035C	YOR123C	29.4675
YDR448W	YMR223W	29.4472
YOR224C	YPR187W	29.4244
YDL160C	YER112W	29.4124
YKL173W	YLL036C	29.4081
YGL252C	YLL022C	29.379
YDR195W	YNL222W	29.3765
YGL097W	YLR293C	29.3686
YER022W	YOL135C	29.3546
YJL124C	YMR268C	29.3191
YHL004W	YKL155C	29.293
YDR347W	YGL129C	29.2488
YDL030W	YML049C	29.2404
YPR070W	YPR168W	29.232
YBR105C	YDR255C	29.1968
YER157W	YNL041C	29.1853
YER022W	YGL025C	29.1557
YGL223C	YNL051W	29.0917
YBR231C	YNL107W	29.0901
YBR251W	YJR113C	29.0827
YBR257W	YNL221C	29.0787
YDR156W	YPR110C	29.0644
YDR385W	YKL191W	29.0244
YGL094C	YKL025C	29.02
YBR198C	YHL047C	29.0169
YBR055C	YDL098C	29.0156
YMR112C	YNR010W	29.0086
YAR002W	YLR335W	29.0039
YGL125W	YPL023C	29.0007
YDR385W	YOR133W	28.9615
YGL044C	YOR250C	28.9394
YBR055C	YER029C	28.9363
YDR347W	YHL004W	28.9307
YDR190C	YGL150C	28.9273
YEL056W	YNL031C	28.9213
YML069W	YOR039W	28.892
YLR147C	YML046W	28.8915
YBR105C	YIL017C	28.8884
YJR093C	YNL222W	28.8784
YKL173W	YLR117C	28.8768
YBL017C	YHR114W	28.8577
YBL007C	YBL017C	28.8577
YBR146W	YDR337W	28.8506
YGR104C	YHR058C	28.8415
YEL036C	YPL050C	28.8403
YNL049C	YPR181C	28.8402
YJL011C	YOR116C	28.8268
YNL031C	YPL001W	28.8266
YNL005C	YNL252C	28.8232
YDR369C	YMR224C	28.802
YAL013W	YOL004W	28.8019
YBR009C	YNL206C	28.7828
YKL144C	YPR110C	28.7774
YJL176C	YPL016W	28.775
YGR275W	YIL126W	28.7687
YIL093C	YKL155C	28.7654
YBR154C	YKR025W	28.7619
YLR373C	YPL128C	28.7596
YKL155C	YNL137C	28.7454
YJL208C	YKR079C	28.7432
YLR399C	YNL107W	28.7404
YLL002W	YNL246W	28.733
YBR081C	YMR236W	28.7286
YFR021W	YNL242W	28.7096
YBR154C	YJL011C	28.7017
YJL155C	YLR345W	28.7008
YLL010C	YOR043W	28.6948
YKL144C	YNL151C	28.6742
YGR074W	YKL173W	28.6736
YGR113W	YKL052C	28.6656
YMR288W	YPL213W	28.6414
YLR447C	YOR270C	28.6156
YML049C	YMR240C	28.6017
YGR162W	YOL139C	28.5909
YCR052W	YGR275W	28.5872
YKL059C	YNL222W	28.5816
YGL197W	YLR310C	28.5481
YNL132W	YOR063W	28.5471
YJR101W	YNL306W	28.5335
YBR289W	YHL025W	28.5307
YMR284W	YOL054W	28.5229
YJL154C	YOR069W	28.5058
YDR334W	YPL235W	28.493
YBR251W	YOR158W	28.4825
YDR022C	YLR423C	28.4798
YAL007C	YAR002C-A	28.4765
YMR112C	YOR174W	28.4549
YDR167W	YMR236W	28.4403
YBR031W	YOR063W	28.4387
YBR081C	YDR167W	28.4337
YBR260C	YGR196C	28.4133
YDR407C	YDR472W	28.4011
YER029C	YER172C	28.3743
YER133W	YMR311C	28.3714
YER027C	YGL208W	28.3572
YDR347W	YOR158W	28.3539
YGL207W	YOR039W	28.3163
YOR063W	YPL198W	28.3078
YDR407C	YMR218C	28.3006
YDR041W	YNL137C	28.2622
YKR001C	YPL082C	28.2376
YDR347W	YNL137C	28.224
YDR473C	YER029C	28.215
YGR013W	YGR074W	28.2143
YOL135C	YOR174W	28.2114
YMR213W	YPR101W	28.2071
YBR154C	YOR341W	28.1931
YBR119W	YDR240C	28.1879
YLR215C	YPL237W	28.1829
YCR093W	YNR052C	28.1297
YDR337W	YPL118W	28.1087
YJR113C	YNL137C	28.0852
YBR253W	YDR308C	28.0664
YJL124C	YNL118C	28.0643
YJL203W	YML049C	28.0607
YBL093C	YPR168W	28.059
YMR213W	YPL151C	28.0553
YDR407C	YKR068C	28.0503
YJR135C	YLR315W	28.0397
YGR020C	YHR039C-A	28.0385
YMR240C	YPL213W	28.0229
YGL223C	YPR105C	28.021
YDR407C	YOR115C	27.9957
YIL093C	YNL137C	27.9856
YDR041W	YIL093C	27.9848
YJL154C	YOR132W	27.9827
YDR460W	YPR056W	27.9685
YNL222W	YPR107C	27.9569
YEL036C	YJL183W	27.9522
YHR058C	YNL236W	27.9307
YGL155W	YKL019W	27.9257
YDR392W	YGL066W	27.8998
YKL122C	YML105C	27.8523
YNL107W	YOR244W	27.8498
YFL039C	YLR085C	27.8347
YDR212W	YIL142W	27.8086
YNR046W	YOL124C	27.7431
YGR120C	YNL041C	27.7361
YFL007W	YOR362C	27.7298
YAL007C	YML012W	27.7288
YAL016W	YOR014W	27.6978
YGR013W	YLR147C	27.6975
YGR215W	YJR113C	27.629
YER083C	YGL020C	27.6253
YBR160W	YLR079W	27.623
YBR095C	YPL181W	27.6217
YDR175C	YDR337W	27.6031
YHR165C	YLR117C	27.5839
YER157W	YML071C	27.5515
YGL213C	YPR189W	27.5502
YGR283C	YMR310C	27.5456
YDR041W	YDR175C	27.5335
YDR337W	YGL129C	27.5325
YBR253W	YNR010W	27.5307
YBR055C	YLR147C	27.52
YGR090W	YNL132W	27.5129
YIL075C	YLR421C	27.5115
YDR190C	YNL107W	27.5036
YHL004W	YOR158W	27.502
YGR013W	YLR275W	27.4818
YJL184W	YKR038C	27.4756
YLL045C	YPL198W	27.4693
YDR489W	YOL146W	27.4622
YBR154C	YNL151C	27.4556
YDR041W	YDR337W	27.4536
YKL018W	YPL138C	27.4521
YER022W	YMR112C	27.4386
YNL041C	YNL051W	27.4369
YGR215W	YHL004W	27.4284
YJR101W	YPL118W	27.4281
YKL122C	YPL243W	27.4108
YNL248C	YPR190C	27.4098
YIL084C	YMR075W	27.4023
YDR328C	YMR258C	27.3951
YNR023W	YPR034W	27.3499
YBR095C	YMR263W	27.3401
YDR293C	YNL201C	27.3274
YOR063W	YOR312C	27.3232
YMR024W	YNL005C	27.3131
YPL031C	YPL219W	27.3111
YDL137W	YDL192W	27.2961
YGL112C	YHL047C	27.2946
YNL306W	YPL118W	27.2844
YJL179W	YNL153C	27.2709
YBR289W	YMR033W	27.2657
YJL184W	YML036W	27.2525
YIR008C	YNL102W	27.2371
YJR101W	YKL155C	27.2346
YDL150W	YNL113W	27.2201
YHL047C	YPL011C	27.2186
YGL025C	YGL127C	27.2068
YNL041C	YPR105C	27.2063
YDL005C	YOR174W	27.2063
YDL147W	YOL117W	27.1856
YDR318W	YJR135C	27.1848
YCR033W	YGL194C	27.1805
YAL034W-A	YJR112W	27.1751
YDR280W	YHR081W	27.1619
YGR200C	YHR187W	27.1357
YLR127C	YNL172W	27.1131
YLR197W	YOR310C	27.0663
YGR013W	YMR125W	27.0608
YLL045C	YOR063W	27.0404
YBR146W	YGR084C	27.0404
YER133W	YPR107C	27.0394
YMR049C	YMR290C	27.0222
YEL056W	YMR076C	27.0189
YDR175C	YOR158W	27.0173
YGL019W	YGL207W	27.0079
YKL206C	YLR199C	26.9894
YHR154W	YJL047C	26.9872
YJL203W	YMR240C	26.9843
YGL151W	YHR058C	26.982
YDR190C	YER092W	26.9583
YHR012W	YJL053W	26.955
YGL129C	YJR101W	26.9394
YMR076C	YPL001W	26.931
YHL030W	YHR200W	26.9158
YGR274C	YML015C	26.9041
YGL111W	YMR290C	26.8986
YBR198C	YCL010C	26.8907
YHR090C	YNL107W	26.8877
YAL029C	YBR130C	26.8769
YHR102W	YOR353C	26.8722
YKL173W	YLR275W	26.8705
YDR347W	YGR165W	26.8681
YHR166C	YNL172W	26.8658
YBR114W	YPR135W	26.8628
YDR347W	YJR101W	26.8625
YHL004W	YNL306W	26.8455
YDR337W	YNL137C	26.8341
YFR010W	YLR421C	26.8331
YLR085C	YML041C	26.8247
YKL144C	YOR116C	26.7982
YPR187W	YPR190C	26.7717
YHR041C	YHR058C	26.7652
YDR237W	YNL005C	26.7242
YGR084C	YKL155C	26.7092
YGL070C	YOR151C	26.7081
YBR146W	YKL155C	26.6771
YNL113W	YOR341W	26.6534
YBR193C	YOL135C	26.649
YDL147W	YDR179C	26.6463
YDR328C	YJR090C	26.6443
YDR201W	YKL052C	26.6405
YJR101W	YJR113C	26.6356
YJR109C	YOR303W	26.6331
YLL004W	YPL001W	26.63
YDR041W	YNL306W	26.6216
YNL113W	YOR207C	26.6122
YCL032W	YLR362W	26.6087
YDR167W	YMR005W	26.6059
YDR325W	YFR031C	26.6044
YJL140W	YPR187W	26.5935
YJL176C	YPR034W	26.5874
YJL124C	YKL173W	26.5852
YMR263W	YPL139C	26.5799
YDR378C	YOR308C	26.5793
YKL060C	YLR044C	26.5218
YHR187W	YPL086C	26.4918
YCL039W	YIL017C	26.4807
YHR086W	YLR275W	26.4786
YDR429C	YOR361C	26.4567
YGL143C	YJL047C	26.4475
YBR055C	YDL030W	26.4465
YMR167W	YNL082W	26.4438
YBR221C	YNL071W	26.4344
YDR167W	YML015C	26.3862
YGR179C	YLR381W	26.3833
YHR084W	YPL049C	26.3581
YBR225W	YLR310C	26.3393
YDL005C	YPR070W	26.3176
YBL090W	YIL093C	26.3092
YMR290C	YOR312C	26.2998
YNL236W	YNR010W	26.287
YGR120C	YML071C	26.2775
YBR146W	YDR041W	26.2646
YBR253W	YMR112C	26.258
YDR383C	YJR135C	26.2526
YDL156W	YEL056W	26.2414
YAL024C	YHR158C	26.2315
YER022W	YOL051W	26.2226
YDL098C	YOR308C	26.1938
YER092W	YJL081C	26.1769
YGL061C	YKL052C	26.1704
YDL156W	YPL001W	26.1422
YGL252C	YIL046W	26.1314
YDL164C	YGL252C	26.1314
YGR165W	YJR113C	26.1087
YJL053W	YJL154C	26.0996
YFL013C	YFL039C	26.088
YBL058W	YBR170C	26.0762
YDR295C	YPR179C	26.0645
YMR308C	YPL001W	26.0459
YDL043C	YJL203W	26.022
YDL056W	YLR182W	26.014
YKL052C	YKR037C	26.005
YDR190C	YML041C	25.9967
YIL061C	YMR125W	25.9507
YKL144C	YNR003C	25.9347
YBR231C	YDR334W	25.9115
YDR422C	YDR477W	25.9087
YGL043W	YGR186W	25.8952
YDR167W	YPL011C	25.8776
YDR041W	YGR084C	25.8759
YDR190C	YDR485C	25.8632
YDR404C	YOL005C	25.855
YMR284W	YNL030W	25.8496
YJR074W	YOR185C	25.8411
YLL036C	YPR101W	25.8313
YGR156W	YOR179C	25.8236
YBR095C	YMR075W	25.8149
YGR179C	YPL018W	25.7872
YNL288W	YNR052C	25.7547
YER029C	YGR091W	25.7512
YDR108W	YDR472W	25.741
YGR275W	YMR033W	25.7329
YBR055C	YPR082C	25.7217
YGR206W	YLR119W	25.711
YGR056W	YHR056C	25.708
YBR279W	YML069W	25.6962
YDR337W	YJR101W	25.6831
YOL135C	YPR070W	25.6822
YDR237W	YNL252C	25.67
YDL030W	YPL213W	25.6694
YMR112C	YPR070W	25.6682
YFR052W	YHL030W	25.6637
YDL030W	YKL173W	25.6521
YJR063W	YPR010C	25.6278
YAR002W	YLR347C	25.6196
YGL207W	YNL031C	25.6139
YAL029C	YOR326W	25.6124
YER022W	YPR168W	25.601
YBL026W	YJR022W	25.6002
YDL115C	YGL070C	25.5937
YER092W	YPL235W	25.5921
YJL115W	YNL031C	25.5505
YER094C	YFL007W	25.5454
YDR416W	YPL151C	25.5339
YBL093C	YGL127C	25.5287
YBR253W	YGR104C	25.525
YER172C	YLR117C	25.5237
YBL058W	YGR048W	25.5229
YER029C	YGR013W	25.507
YBL007C	YHR114W	25.4832
YGR090W	YOR039W	25.4627
YJL081C	YPR023C	25.4575
YFL005W	YFL038C	25.3998
YGR063C	YML010W	25.3956
YMR188C	YPL118W	25.3877
YIL084C	YPR023C	25.3803
YGR275W	YKR008W	25.3569
YDR108W	YOR115C	25.3361
YDR041W	YKL155C	25.3319
YHR174W	YOL086C	25.3316
YGR240C	YMR205C	25.3198
YIL021W	YML010W	25.3021
YDR289C	YGL246C	25.2997
YGL151W	YNR010W	25.2924
YMR223W	YPL254W	25.2812
YGR274C	YMR236W	25.2579
YDL076C	YIL084C	25.2569
YAL002W	YMR231W	25.252
YBR175W	YPL138C	25.2381
YKL018W	YNL222W	25.2346
YAL011W	YPL235W	25.2206
YBR175W	YLR015W	25.2095
YDL140C	YPR187W	25.2009
YDR156W	YJL148W	25.1987
YDR322W	YDR462W	25.1473
YGR215W	YNR037C	25.1438
YDR116C	YDR322W	25.1266
YBR081C	YHR099W	25.1263
YGL207W	YLR418C	25.0945
YBR193C	YPR168W	25.0916
YDL002C	YFL039C	25.0734
YBR154C	YJL148W	25.0728
YDR448W	YMR236W	25.0584
YDR041W	YGL129C	25.0406
YDL116W	YLR208W	25.0386
YMR049C	YOL077C	25.0348
YHR114W	YOR181W	25.0257
YHL047C	YMR236W	25.0193
YMR186W	YPL240C	25.015
YBL084C	YLR127C	25.0111
YFL007W	YFR050C	25.0101
YGL122C	YOL123W	25.0098
YKL210W	YPL126W	25.007
YDL115C	YDL140C	24.9916
YGR078C	YJL179W	24.9829
YOR340C	YPR187W	24.9779
YMR033W	YOR290C	24.965
YDL042C	YLR442C	24.9474
YHL004W	YMR188C	24.9459
YBR102C	YLR166C	24.9386
YAL021C	YNL288W	24.9338
YNL238W	YOL031C	24.9324
YBR193C	YDL005C	24.9305
YNL107W	YPL235W	24.924
YIL021W	YPR187W	24.917
YHR119W	YKL018W	24.9111
YBR125C	YDR071C	24.9103
YHL029C	YNL099C	24.9004
YCR095C	YNL099C	24.9004
YAL029C	YKL130C	24.8844
YDL188C	YMR273C	24.8728
YCR066W	YGL058W	24.8724
YBR154C	YGL070C	24.8382
YGR084C	YOR158W	24.8045
YDR235W	YMR125W	24.7992
YER092W	YFL013C	24.7922
YDR347W	YMR188C	24.7754
YIL050W	YPL031C	24.7676
YDL156W	YML069W	24.756
YML069W	YOL145C	24.7539
YGR005C	YPR187W	24.7287
YGR165W	YNL306W	24.7208
YBR289W	YPL016W	24.715
YLR347C	YMR049C	24.7048
YGR104C	YNR010W	24.694
YOL145C	YOR039W	24.6926
YDR473C	YER172C	24.6884
YER172C	YML049C	24.6826
YDR060W	YOR206W	24.6752
YIL093C	YMR188C	24.6634
YBR094W	YEL015W	24.6441
YOR361C	YPR041W	24.6164
YDR404C	YGL070C	24.608
YFL049W	YOR290C	24.6042
YDL007W	YGR232W	24.6011
YOL051W	YPR168W	24.5682
YGR165W	YOR158W	24.5539
YJR063W	YPR110C	24.5487
YGR145W	YNL132W	24.5466
YLR337C	YOR181W	24.5386
YBR245C	YOR304W	24.5379
YHR056C	YML127W	24.5137
YBR102C	YIL068C	24.4983
YLR192C	YOR361C	24.4863
YBL093C	YER022W	24.4827
YDR240C	YMR125W	24.4801
YGL087C	YHR137W	24.4766
YGR275W	YLR321C	24.4569
YDL115C	YDR404C	24.4344
YDL087C	YMR125W	24.4185
YPR082C	YPR178W	24.4117
YDR337W	YGR084C	24.3758
YMR290C	YOR272W	24.3729
YKL173W	YPL213W	24.3659
YGL244W	YML069W	24.3516
YBR030W	YLR244C	24.3509
YDL160C	YNL118C	24.3367
YEL056W	YMR308C	24.3329
YDL030W	YLL036C	24.3267
YLR071C	YNR010W	24.3195
YDR473C	YJL124C	24.2945
YDL134C	YMR273C	24.2923
YAL011W	YLR085C	24.29
YGL061C	YGR113W	24.2833
YGR103W	YPL093W	24.2771
YER025W	YPR041W	24.2749
YER025W	YMR309C	24.2706
YOR158W	YPL118W	24.2629
YDR347W	YGR215W	24.2584
YKL028W	YKR062W	24.2573
YKL155C	YOR158W	24.2555
YDL147W	YIL071C	24.2481
YBL026W	YMR268C	24.2458
YNL290W	YOR144C	24.243
YDR156W	YPR010C	24.2371
YNL113W	YPR187W	24.2323
YDR322W	YJL063C	24.2269
YMR172W	YOR039W	24.223
YHL029C	YNL032W	24.2029
YCR095C	YNL032W	24.2029
YNL056W	YNL099C	24.1695
YDL097C	YLR421C	24.1612
YBL047C	YCR030C	24.1606
YDR415C	YKL210W	24.119
YBR198C	YMR223W	24.1188
YJL164C	YPL203W	24.1174
YDL051W	YPR088C	24.116
YCR081W	YGL151W	24.1151
YDR148C	YIL125W	24.1082
YAL032C	YLR117C	24.1079
YER012W	YOR157C	24.0919
YBL007C	YOR181W	24.0712
YAL007C	YGL200C	24.0671
YJL164C	YKL166C	24.0665
YNL151C	YPR187W	24.0649
YDL098C	YKL173W	24.0578
YER006W	YPL093W	24.0478
YBR279W	YGL207W	24.0381
YAL047C	YHR172W	24.0282
YHR076W	YMR079W	24.0162
YDR357C	YKL061W	24.0162
YBL093C	YBR193C	24.0136
YER146W	YJL124C	23.9983
YKL145W	YLR421C	23.9934
YFL007W	YMR314W	23.986
YDR101C	YJL122W	23.9743
YDL077C	YPL045W	23.9648
YER172C	YMR288W	23.9519
YGL207W	YMR076C	23.9469
YCL010C	YDR145W	23.9429
YKR025W	YPR187W	23.927
YKL057C	YLR208W	23.9262
YGR161C	YJL098W	23.9261
YBR146W	YJR101W	23.907
YBR154C	YOR207C	23.9022
YCR079W	YOR323C	23.8993
YDR012W	YML063W	23.8938
YBR146W	YNL306W	23.891
YAL047C	YNL126W	23.8885
YJR042W	YPL169C	23.8703
YLL026W	YLR249W	23.8654
YOR151C	YOR224C	23.8612
YGR005C	YPL129W	23.852
YDL185W	YHR039C-A	23.8424
YOR141C	YPL129W	23.8393
YDR190C	YDR334W	23.8385
YMR049C	YNL189W	23.8368
YAR002W	YNL189W	23.7969
YMR033W	YPL016W	23.7869
YLR147C	YOR159C	23.7425
YBR253W	YGL025C	23.742
YCR071C	YLR439W	23.7373
YGR017W	YLR410W	23.7366
YDR308C	YNL236W	23.7331
YBR055C	YDR378C	23.7247
YOR210W	YOR340C	23.7159
YBR193C	YMR112C	23.7063
YIL144W	YMR117C	23.7024
YER017C	YMR089C	23.6921
YHR012W	YOR069W	23.6907
YDL014W	YLL045C	23.6819
YDR385W	YIL103W	23.6766
YER172C	YGR074W	23.6731
YLR071C	YPL042C	23.6491
YKL166C	YPL203W	23.6487
YBR160W	YPR119W	23.648
YDL042C	YDR227W	23.6451
YAL021C	YGR134W	23.641
YDR245W	YGR231C	23.6325
YDL005C	YHR058C	23.6285
YNL137C	YOR158W	23.6234
YBR081C	YMR223W	23.6177
YGR091W	YLR147C	23.6166
YHR165C	YMR288W	23.6039
YER102W	YMR146C	23.6003
YDR337W	YMR188C	23.5907
YCR052W	YHR056C	23.5813
YDR484W	YJL029C	23.5696
YBR011C	YLR371W	23.5682
YDL150W	YPR187W	23.5679
YMR061W	YOR250C	23.5675
YAL047C	YLR212C	23.5584
YDL087C	YLR275W	23.5313
YHR056C	YKR008W	23.5299
YDR045C	YOR116C	23.5103
YMR117C	YOL069W	23.5084
YCL010C	YMR223W	23.49
YJR113C	YOR158W	23.4873
YAR003W	YKL018W	23.4872
YIL061C	YLR298C	23.4711
YDL098C	YPR178W	23.4687
YBR119W	YLR275W	23.4484
YGR090W	YKL078W	23.4471
YBR146W	YBR251W	23.4464
YGR084C	YGR165W	23.4333
YDR246W	YOR115C	23.432
YGR013W	YLR298C	23.426
YDR308C	YGL151W	23.3836
YDL115C	YGR005C	23.3811
YGR084C	YNL306W	23.3732
YDR116C	YLR439W	23.3576
YCR057C	YDR324C	23.3555
YGL043W	YJL140W	23.3315
YLR147C	YPR178W	23.3235
YDR361C	YPL208W	23.3197
YER172C	YLL036C	23.3164
YKL058W	YOR194C	23.3154
YGR124W	YPR145W	23.3154
YDL147W	YMR025W	23.3072
YER029C	YPL213W	23.2974
YGR104C	YOL135C	23.2822
YDR075W	YDR293C	23.2749
YBL026W	YOR308C	23.2749
YML049C	YPL213W	23.2623
YMR236W	YPL254W	23.2602
YDR337W	YGR215W	23.2573
YHL030W	YOR261C	23.2522
YML100W	YMR261C	23.2208
YBR130C	YKL130C	23.2206
YDL051W	YDL092W	23.2143
YOL005C	YOR224C	23.2126
YJL115W	YMR076C	23.1884
YHR039C-A	YPR036W	23.1791
YDR308C	YHR041C	23.1739
YDR041W	YOR158W	23.1634
YLR055C	YMR236W	23.1528
YDR121W	YNL262W	23.1473
YDR045C	YJL011C	23.1277
YGR104C	YMR112C	23.1097
YIL093C	YOR158W	23.1084
YFL038C	YPR017C	23.1014
YHR013C	YJR145C	23.0996
YOR310C	YPR137W	23.0976
YDR045C	YNL151C	23.0837
YLR127C	YOR249C	23.0807
YDR324C	YDR449C	23.0786
YHR196W	YJL109C	23.0638
YBL026W	YBR055C	23.0547
YFL034W	YKL135C	23.0545
YLL022C	YNL031C	23.0455
YGR215W	YIL093C	23.0435
YFR037C	YGR275W	23.04
YIL021W	YPL225W	23.003
YDR359C	YHR099W	22.9952
YFL037W	YML085C	22.9943
YHL004W	YJR101W	22.976
YLR275W	YOR159C	22.9735
YDR116C	YMR024W	22.9672
YGL252C	YOL054W	22.9513
YDR001C	YDR099W	22.9457
YGR278W	YLL036C	22.9264
YBR289W	YFL049W	22.9219
YAL027W	YPL022W	22.9195
YGR074W	YOR159C	22.9158
YDR156W	YNL113W	22.8967
YMR263W	YNL330C	22.8641
YDR462W	YNL252C	22.8623
YER151C	YNR051C	22.8446
YBL090W	YNL306W	22.8329
YDR404C	YPR187W	22.805
YNL139C	YNL253W	22.8019
YEL018W	YJL081C	22.7953
YDR378C	YGR091W	22.7934
YPL042C	YPR070W	22.7663
YDR328C	YIL046W	22.7633
YJR113C	YNR037C	22.761
YCL039W	YDR255C	22.7521
YDR195W	YOR179C	22.7477
YFR031C	YLR086W	22.7389
YHL004W	YNR037C	22.732
YDR041W	YJR101W	22.7269
YDR175C	YJR101W	22.7029
YGR165W	YKL155C	22.6983
YER127W	YNL132W	22.6731
YGR074W	YML046W	22.6541
YPL001W	YPR162C	22.6432
YAL035W	YLR192C	22.6381
YEL055C	YMR093W	22.6344
YGL127C	YNR010W	22.6285
YDL115C	YOR151C	22.6172
YBR031W	YPL126W	22.6141
YBL056W	YDR071C	22.5882
YGR192C	YHR174W	22.5808
YHR166C	YOR249C	22.5784
YDL007W	YHL030W	22.5704
YMR005W	YMR227C	22.5679
YGR103W	YOL077C	22.5618
YGL098W	YNL258C	22.5533
YGL098W	YLR440C	22.5533
YDL150W	YDR045C	22.5532
YDR027C	YDR484W	22.5386
YKL173W	YML049C	22.521
YNL051W	YPR105C	22.5092
YHR056C	YLR033W	22.5079
YLR146C	YPR069C	22.5045
YKL074C	YLR116W	22.5045
YGR262C	YML036W	22.4999
YJL063C	YLR439W	22.497
YHR058C	YOL135C	22.4958
YDR202C	YJR033C	22.4733
YDR322W	YML025C	22.4697
YHR193C	YPL037C	22.433
YJR007W	YMR257C	22.4268
YIR026C	YJR007W	22.4268
YKL122C	YPL210C	22.4123
YDL030W	YER172C	22.4113
YDR308C	YMR112C	22.4039
YGL005C	YNL051W	22.3981
YJL148W	YOR341W	22.3794
YLR275W	YPR178W	22.3791
YER172C	YPL151C	22.3773
YJR068W	YOR144C	22.3681
YDR405W	YLR439W	22.3557
YDR337W	YGR165W	22.3516
YDL014W	YLR197W	22.3468
YBR094W	YOL149W	22.3462
YDL030W	YER029C	22.346
YBR107C	YJR135C	22.3411
YGR275W	YMR091C	22.3405
YDR195W	YPR107C	22.3242
YDL077C	YMR231W	22.2975
YDL077C	YLR148W	22.2975
YNL258C	YOR075W	22.2909
YLR440C	YOR075W	22.2909
YJR101W	YNL137C	22.2893
YNL248C	YPR187W	22.2798
YBR017C	YOL123W	22.2647
YNL061W	YPL211W	22.2618
YHR041C	YNR010W	22.2603
YGL019W	YOL006C	22.2561
YCR077C	YPR178W	22.2461
YDL098C	YHR165C	22.2404
YBL026W	YGR091W	22.2399
YLR226W	YPR161C	22.239
YDR308C	YGR104C	22.2305
YGL145W	YKL196C	22.2277
YGL049C	YOL139C	22.2254
YBR135W	YMR199W	22.2251
YGL206C	YGR167W	22.2159
YHR062C	YNL282W	22.2109
YGR252W	YMR236W	22.2002
YDL030W	YHR165C	22.1947
YGL120C	YOL115W	22.1946
YDR267C	YHR122W	22.1932
YKR002W	YNL222W	22.1921
YGR186W	YPL129W	22.1912
YBL103C	YOL067C	22.1629
YNL113W	YNL151C	22.1574
YDL098C	YGR075C	22.1442
YBL026W	YKL188C	22.1401
YNL137C	YNL306W	22.1275
YJL072C	YOL146W	22.1244
YDR013W	YOL146W	22.1244
YGR020C	YOR332W	22.1149
YAL033W	YBR257W	22.1138
YJL063C	YMR024W	22.1076
YOL005C	YOR210W	22.1045
YCR077C	YNL147W	22.1005
YDR303C	YHR056C	22.1001
YBR251W	YMR188C	22.0891
YDL030W	YLR147C	22.0783
YDR060W	YMR290C	22.0779
YGR071C	YPL128C	22.0738
YDR324C	YLR222C	22.071
YKR085C	YML025C	22.068
YGL112C	YHR099W	22.0627
YJR101W	YMR188C	22.0602
YGR278W	YLR117C	22.0367
YGL019W	YMR172W	22.0226
YER149C	YLL021W	22.0034
YEL003W	YLR200W	21.9914
YHR086W	YMR125W	21.9898
YGR074W	YPR178W	21.9854
YFL008W	YIL026C	21.9854
YJL024C	YPL195W	21.9655
YLR055C	YMR223W	21.9631
YER029C	YMR240C	21.9612
YNL078W	YOL070C	21.957
YGL086W	YJL030W	21.9302
YGL187C	YNL052W	21.9292
YML046W	YMR125W	21.9085
YBR257W	YHR062C	21.8951
YKL196C	YNL258C	21.8843
YKL196C	YLR440C	21.8843
YOR332W	YPR036W	21.8618
YGL237C	YOR358W	21.8488
YBR253W	YLR071C	21.843
YDL043C	YMR240C	21.8151
YER102W	YMR309C	21.8141
YAL032C	YDR416W	21.7922
YPL003W	YPR066W	21.7667
YGR215W	YOR158W	21.7534
YGR165W	YHL004W	21.7521
YFR009W	YKL104C	21.7321
YBR154C	YKL144C	21.72
YOR207C	YPR187W	21.7126
YDR334W	YLR385C	21.6847
YHR042W	YIL112W	21.6831
YNL221C	YNL282W	21.6509
YER125W	YOR124C	21.6409
YER021W	YHL030W	21.6303
YGL223C	YGR120C	21.6158
YGL005C	YPR105C	21.6158
YGL005C	YGR120C	21.6158
YBR146W	YMR188C	21.6095
YGL207W	YOL145C	21.5905
YDR041W	YDR347W	21.5823
YDL115C	YGR186W	21.5818
YGL151W	YMR112C	21.5538
YBR095C	YPR023C	21.5424
YNL312W	YPL240C	21.5362
YKL139W	YML112W	21.5217
YJL006C	YKL139W	21.5217
YBL090W	YJR113C	21.5151
YLR147C	YML049C	21.4991
YNL189W	YPR023C	21.4914
YDL042C	YFL007W	21.4883
YCL043C	YHR204W	21.4829
YNR010W	YPL042C	21.4808
YDR308C	YOL135C	21.4724
YGL150C	YJL081C	21.458
YDL030W	YPR178W	21.4517
YJL050W	YPL190C	21.4435
YGL043W	YIL021W	21.4377
YDL014W	YPL198W	21.4355
YJR006W	YJR043C	21.4169
YHR099W	YPL254W	21.4166
YKL206C	YMR314W	21.4125
YBL074C	YKL173W	21.4085
YDR167W	YGR274C	21.4073
YJL124C	YNL147W	21.4065
YDL156W	YJL115W	21.4061
YIL128W	YNL240C	21.4035
YDR378C	YDR473C	21.3939
YHR041C	YMR112C	21.3831
YBR135W	YLR079W	21.3806
YLR006C	YNR031C	21.3803
YBR103W	YHR042W	21.3758
YDR473C	YLR275W	21.3698
YIL098C	YJL180C	21.3695
YCR093W	YNL288W	21.3599
YGR091W	YPR082C	21.3419
YHR012W	YOR132W	21.3328
YGL070C	YOR210W	21.3307
YML049C	YOR319W	21.3215
YDR041W	YGR165W	21.3157
YBL026W	YKL173W	21.3096
YKL173W	YMR288W	21.3055
YBR193C	YGL127C	21.3054
YHR013C	YNL132W	21.2974
YFR002W	YGR119C	21.2963
YDR324C	YKL210W	21.2952
YGL128C	YLR117C	21.2816
YJL065C	YOR304W	21.2602
YGL133W	YJL065C	21.2602
YEL051W	YOR332W	21.2463
YER022W	YPL042C	21.2377
YHR122W	YIL128W	21.2365
YBL052C	YPR031W	21.2297
YDR443C	YPR070W	21.2265
YGL252C	YJR090C	21.2244
YLR275W	YML046W	21.2183
YBL007C	YLR337C	21.2169
YGL252C	YML069W	21.2168
YAR007C	YHR164C	21.1906
YGR084C	YMR188C	21.1818
YGR250C	YIR001C	21.1803
YGL207W	YGR017W	21.1762
YDL209C	YLL036C	21.1682
YGR048W	YMR067C	21.1487
YGL151W	YOL135C	21.1373
YAL035W	YDR429C	21.133
YDR116C	YJL063C	21.1296
YKR079C	YMR099C	21.1257
YJL208C	YMR099C	21.1257
YAL033W	YBL018C	21.1173
YBL035C	YGL252C	21.1146
YLR298C	YML046W	21.1109
YER136W	YFL038C	21.0996
YLR147C	YMR240C	21.0932
YCR081W	YNL236W	21.0914
YDR443C	YGL151W	21.0805
YGR005C	YOR210W	21.0798
YGR252W	YHR099W	21.0709
YBL017C	YDR388W	21.0661
YCR081W	YPR070W	21.0633
YBR102C	YGL233W	21.0592
YGL252C	YNL030W	21.0515
YDL087C	YLR147C	21.0394
YKL155C	YMR188C	21.0007
YKR014C	YMR258C	20.9789
YJR063W	YNL248C	20.969
YLR147C	YOR308C	20.9628
YDR176W	YMR236W	20.9582
YIL068C	YPR055W	20.9269
YDR473C	YGR074W	20.9238
YLL022C	YMR076C	20.9133
YGL129C	YMR188C	20.9035
YBR119W	YGR074W	20.902
YBL008W	YBR215W	20.8954
YBR146W	YOR158W	20.8878
YGL070C	YPR187W	20.882
YBR211C	YLR381W	20.8808
YNL236W	YOL135C	20.8579
YOR290C	YPL016W	20.8518
YGL025C	YMR112C	20.8503
YGR275W	YLR357W	20.8468
YDR485C	YPL235W	20.8441
YGR002C	YOR244W	20.841
YJL072C	YPR135W	20.8399
YDR489W	YPR135W	20.8399
YDR013W	YPR135W	20.8399
YKR071C	YPR048W	20.8306
YHL025W	YOR290C	20.8238
YDR427W	YHL030W	20.8236
YKL045W	YNL102W	20.8164
YIR008C	YKL045W	20.8164
YBL035C	YKL045W	20.8164
YBL035C	YIR008C	20.8164
YDR041W	YMR188C	20.8033
YNR010W	YOL135C	20.7816
YCR077C	YMR268C	20.7581
YJL140W	YOR224C	20.7526
YDR101C	YPL093W	20.7247
YOR078W	YPR137W	20.7198
YLR216C	YMR186W	20.695
YBL090W	YDR337W	20.6862
YBL018C	YNL221C	20.677
YPL085W	YPR181C	20.6733
YBR251W	YDR041W	20.6666
YGR192C	YKL060C	20.6587
YMR312W	YPL101W	20.6584
YER008C	YPR055W	20.6571
YDR166C	YPR055W	20.6571
YER070W	YJL026W	20.6396
YKL144C	YPR187W	20.6387
YFL007W	YGR135W	20.6332
YDR359C	YHR090C	20.6302
YGR084C	YGR215W	20.623
YGR002C	YHR090C	20.6221
YBL026W	YER172C	20.6198
YHR090C	YJL081C	20.5997
YNL248C	YNR003C	20.5979
YHL019C	YKL135C	20.5822
YDL098C	YER029C	20.5708
YDR045C	YKR025W	20.5687
YDL077C	YLR396C	20.5673
YBL026W	YDL160C	20.5653
YDR416W	YHR165C	20.5585
YDR045C	YOR207C	20.5567
YBR253W	YNL236W	20.5557
YDL047W	YGR161C	20.543
YGR063C	YIL021W	20.5358
YLR028C	YMR120C	20.5316
YAR003W	YBR175W	20.5186
YDR167W	YMR227C	20.5088
YDL098C	YGR091W	20.4952
YER107C	YJL061W	20.4821
YGR134W	YJR011C	20.4744
YHR165C	YLL036C	20.4728
YDL115C	YOR210W	20.4681
YER172C	YPL213W	20.4653
YDL040C	YOR253W	20.4628
YJR135C	YPL018W	20.4579
YAL032C	YMR213W	20.4554
YBL084C	YDR118W	20.4531
YDR496C	YOR206W	20.4472
YFR003C	YKL193C	20.4382
YIL061C	YPR182W	20.4176
YJL140W	YOR210W	20.4017
YBL084C	YNL172W	20.3768
YDL087C	YGR074W	20.3763
YDL108W	YPR025C	20.3713
YGR261C	YJL024C	20.3296
YDL140C	YML010W	20.3267
YDR237W	YDR462W	20.3247
YDR190C	YGR002C	20.3236
YDR116C	YNL005C	20.3224
YDR118W	YGL240W	20.3151
YMR227C	YMR236W	20.3101
YDL005C	YPR168W	20.3047
YBR245C	YOL017W	20.2916
YHR135C	YNL154C	20.2868
YGR186W	YPR187W	20.2758
YDL175C	YGL120C	20.2708
YER008C	YGL233W	20.2524
YDR166C	YGL233W	20.2524
YDL126C	YDR049W	20.2503
YKL173W	YPL151C	20.2491
YNL284C	YPL173W	20.249
YOR063W	YOR206W	20.2445
YJL063C	YML025C	20.2315
YBL026W	YER029C	20.2112
YBR152W	YDL098C	20.1979
YDR490C	YGR086C	20.1941
YDL051W	YML105C	20.1937
YDR398W	YHR196W	20.1881
YDR449C	YMR093W	20.1859
YNL261W	YPL001W	20.182
YKL012W	YMR125W	20.17
YGR275W	YLR033W	20.1695
YHL019C	YPR029C	20.1691
YJL140W	YML010W	20.164
YDR224C	YGL241W	20.154
YKL173W	YPR082C	20.1496
YMR049C	YNL061W	20.1394
YDR378C	YER172C	20.1383
YDL229W	YER022W	20.1293
YHR056C	YLR321C	20.1242
YKL012W	YPR182W	20.1212
YHR203C	YJR145C	20.1206
YMR188C	YNL137C	20.1185
YJR007W	YMR146C	20.1128
YKL103C	YOL082W	20.1092
YDL030W	YDL043C	20.0877
YBR112C	YCR084C	20.0783
YLL022C	YMR308C	20.0754
YGR215W	YKL155C	20.074
YER029C	YMR288W	20.0641
YGR200C	YMR312W	20.0616
YBL038W	YLR439W	20.0144
YIR010W	YJR112W	20.0118
YER029C	YML049C	20.0026
YBR215W	YOR038C	19.9986
YKL167C	YML025C	19.9692
YGR074W	YOR308C	19.9672
YNL132W	YOR312C	19.9639
YBR251W	YNL306W	19.9458
YGR002C	YPL235W	19.9453
YMR049C	YPL093W	19.9402
YDL155W	YLR079W	19.9331
YMR049C	YOR206W	19.8965
YBR251W	YGR215W	19.8803
YDR156W	YOR341W	19.8631
YDR156W	YOR210W	19.8464
YGL246C	YLR347C	19.8391
YBR146W	YGR165W	19.8305
YFL002C	YLR002C	19.8278
YDL014W	YJR145C	19.8213
YDL150W	YOR224C	19.8028
YBR127C	YGL246C	19.7983
YER025W	YLR215C	19.7965
YJR104C	YMR038C	19.7815
YFL007W	YGL011C	19.7706
YGR233C	YPL219W	19.7562
YJL179W	YLR200W	19.747
YGR010W	YLR328W	19.7319
YGL097W	YOR185C	19.7302
YGL127C	YHR058C	19.7275
YDR363W-A	YOR259C	19.7202
YGL061C	YKR083C	19.7095
YDR167W	YGL066W	19.7042
YNR053C	YPL093W	19.6978
YAL032C	YLL036C	19.6965
YHR058C	YLR071C	19.6926
YKR068C	YMR218C	19.6844
YBR254C	YMR218C	19.6844
YBR254C	YKR068C	19.6844
YBR254C	YDR407C	19.6844
YDR496C	YMR290C	19.6839
YEL051W	YJR033C	19.6684
YEL036C	YGR132C	19.649
YLR436C	YMR304W	19.6475
YGR084C	YJR101W	19.6362
YDL160C	YKL188C	19.6342
YDL060W	YNL207W	19.6186
YDL043C	YMR288W	19.6157
YDR028C	YER133W	19.6146
YEL018W	YHR090C	19.604
YGR119C	YJL041W	19.5997
YDR167W	YPL254W	19.5985
YLR117C	YLR424W	19.5805
YHR069C	YNL189W	19.5764
YDR141C	YNL297C	19.5677
YOR341W	YPR187W	19.5668
YDR240C	YER029C	19.5526
YDL005C	YER022W	19.5409
YDL098C	YER172C	19.539
YMR172W	YOR061W	19.5354
YLR385C	YPL235W	19.5291
YLR115W	YNL222W	19.5182
YAL003W	YPL048W	19.5148
YFL034C-B	YNL161W	19.5107
YFL007W	YNL189W	19.5057
YDL042C	YNL189W	19.5057
YGL207W	YGL244W	19.5048
YGR231C	YJL183W	19.5026
YGR166W	YMR218C	19.4914
YGR166W	YKR068C	19.4914
YDR407C	YGR166W	19.4914
YBR254C	YGR166W	19.4914
YLR222C	YPL126W	19.477
YJL063C	YNL005C	19.4658
YFL024C	YHR099W	19.4623
YOL111C	YOR007C	19.4481
YBL014C	YJL025W	19.4481
YMR218C	YOR115C	19.4291
YKR068C	YOR115C	19.4291
YDR472W	YMR218C	19.4291
YDR472W	YKR068C	19.4291
YBR254C	YOR115C	19.4291
YBR254C	YDR472W	19.4291
YHR099W	YJL081C	19.4189
YBR281C	YML064C	19.4076
YDR118W	YLR102C	19.4017
YHR034C	YPL235W	19.4012
YDL087C	YLR298C	19.3951
YER025W	YMR146C	19.3909
YDR448W	YHR099W	19.354
YDR443C	YER022W	19.3537
YDL098C	YDR473C	19.353
YGL240W	YLR127C	19.3483
YDR041W	YGR215W	19.3444
YKL193C	YMR311C	19.3366
YGR090W	YHR013C	19.2819
YBR055C	YGR074W	19.2728
YLR347C	YMR125W	19.2709
YGR134W	YNR052C	19.2708
YGR134W	YNL288W	19.2708
YML010W	YOR151C	19.2509
YLR439W	YML025C	19.2373
YGR166W	YOR115C	19.2361
YDR472W	YGR166W	19.2361
YDL051W	YPL210C	19.2342
YLR275W	YML049C	19.2256
YBR130C	YGL106W	19.2202
YCR081W	YER022W	19.2085
YBR160W	YPL256C	19.2076
YBL026W	YNL147W	19.2028
YMR048W	YNL273W	19.1928
YAL032C	YPR101W	19.1894
YBR251W	YKL155C	19.1839
YBR082C	YKL010C	19.1695
YIL026C	YJL074C	19.1676
YCR020W-B	YIL126W	19.165
YNL189W	YOR001W	19.1618
YDR337W	YOR158W	19.1581
YBR280C	YNL141W	19.1532
YGL025C	YOL135C	19.1493
YML025C	YMR193W	19.1428
YJR063W	YOR340C	19.1372
YKL012W	YLR298C	19.1352
YHL025W	YPL016W	19.1199
YER094C	YLR263W	19.1146
YAL053W	YER094C	19.1146
YFL007W	YLR442C	19.1139
YDR045C	YPR190C	19.1071
YNL236W	YPL042C	19.1054
YJL203W	YPL213W	19.0736
YDR188W	YDR212W	19.0477
YKL191W	YOR133W	19.0475
YMR193W	YNL252C	19.045
YGR215W	YMR188C	19.0405
YMR284W	YNL206C	19.0395
YDR365C	YNR054C	19.0334
YDR172W	YGL019W	19.0311
YBR160W	YMR199W	19.0148
YIL034C	YIR003W	19.0143
YDR118W	YKL022C	18.9926
YHR039C-A	YJR033C	18.9853
YKR025W	YOR224C	18.9822
YBR245C	YPL082C	18.9691
YDR473C	YPR082C	18.9656
YJL179W	YML094W	18.9624
YNL097C	YPR023C	18.9621
YDR460W	YER171W	18.9618
YDL156W	YLL022C	18.961
YDR175C	YGR215W	18.9527
YIL021W	YOR210W	18.9413
YOR340C	YPR190C	18.9406
YLR071C	YOL135C	18.9355
YEL051W	YHR039C-A	18.9354
YCL017C	YER048W-A	18.9334
YJR121W	YNL315C	18.9294
YBR119W	YLR147C	18.9241
YKL078W	YNL186W	18.9229
YBR109C	YMR109W	18.9177
YKL080W	YOR332W	18.9168
YCL031C	YGR090W	18.9069
YDR195W	YDR301W	18.897
YER125W	YJL084C	18.8957
YOL139C	YOR276W	18.8904
YDL209C	YDR416W	18.872
YGL240W	YHR166C	18.8458
YDR091C	YLR192C	18.8416
YDR022C	YPR049C	18.8399
YMR049C	YNL002C	18.837
YMR236W	YOL148C	18.8367
YDR443C	YGR104C	18.8328
YBR055C	YLR275W	18.8273
YER006W	YGR103W	18.8268
YMR075W	YNL097C	18.826
YLR215C	YOR260W	18.8228
YJR022W	YNL147W	18.8221
YCR057C	YJL109C	18.8209
YCR009C	YPL249C	18.8159
YDL087C	YER029C	18.8053
YGR092W	YIL106W	18.7861
YOR210W	YOR341W	18.7819
YBR193C	YPL042C	18.7817
YMR268C	YPR178W	18.7813
YBR225W	YGL197W	18.7781
YJL005W	YNL138W	18.7716
YER031C	YFL038C	18.748
YDR398W	YGR128C	18.7437
YBR231C	YDR485C	18.7418
YLR347C	YPL178W	18.7403
YDR328C	YGL252C	18.7363
YGR084C	YNR037C	18.7344
YCR071C	YDR116C	18.7128
YER157W	YGL223C	18.7058
YER157W	YGL005C	18.7058
YCL008C	YPL065W	18.6979
YBR081C	YPL011C	18.6979
YPL213W	YPR178W	18.6928
YGL098W	YLR268W	18.6911
YCR081W	YGR104C	18.688
YNL098C	YOR101W	18.6868
YDR517W	YER094C	18.6811
YGR104C	YPL042C	18.6769
YGR128C	YKL210W	18.6742
YDL126C	YKL213C	18.6661
YHR069C	YNR024W	18.6527
YBL038W	YCR071C	18.6361
YFL007W	YOL038W	18.6138
YBR154C	YOR116C	18.6092
YCR035C	YNL189W	18.6002
YDR022C	YPL166W	18.599
YER094C	YJR051W	18.5982
YDR443C	YLR071C	18.5971
YDL090C	YKL019W	18.5971
YNL023C	YNL135C	18.5856
YFL039C	YLR429W	18.5721
YCR042C	YML114C	18.5683
YGL127C	YPR070W	18.5517
YGL105W	YGL245W	18.5477
YDR303C	YGR275W	18.5476
YDR416W	YJR050W	18.5455
YML032C	YNL312W	18.5319
YER029C	YOR159C	18.5287
YBR137W	YOR164C	18.5156
YIL142W	YJR064W	18.5112
YDR363W-A	YFR004W	18.5086
YKR085C	YNL252C	18.506
YDR167W	YDR448W	18.4978
YLR268W	YOR075W	18.4975
YDR416W	YGR278W	18.4911
YGL207W	YGL241W	18.4843
YBR119W	YLR298C	18.4826
YDR308C	YLR071C	18.475
YNR024W	YOR001W	18.4703
YDR449C	YJL109C	18.4649
YLR119W	YPL065W	18.4615
YPR010C	YPR187W	18.4589
YDR190C	YLR385C	18.4576
YBR131W	YGL124C	18.4549
YCR081W	YLR071C	18.4525
YDR429C	YER102W	18.4484
YGR192C	YOL086C	18.4483
YDR212W	YJL014W	18.4381
YER172C	YPR082C	18.4223
YBL021C	YOR358W	18.4185
YGR220C	YKR085C	18.4112
YBL026W	YDR473C	18.4107
YBR137W	YOR007C	18.4103
YLR216C	YPL240C	18.3835
YDR443C	YNL236W	18.373
YAL002W	YPL045W	18.3657
YOL094C	YOR144C	18.3648
YDR322W	YLR312W-A	18.3643
YER029C	YPR182W	18.3528
YBR175W	YHR119W	18.3253
YDL043C	YML049C	18.3226
YBL067C	YOL087C	18.3204
YEL055C	YHR196W	18.3146
YBR031W	YDR324C	18.3124
YKR022C	YLR424W	18.3028
YOR116C	YPR187W	18.2989
YDR212W	YJR064W	18.2822
YAL016W	YGR161C	18.2764
YGR063C	YJL140W	18.2656
YBR268W	YNL185C	18.2542
YGR186W	YML010W	18.2442
YDL030W	YGR091W	18.2389
YGR091W	YJL124C	18.2383
YHR166C	YLR102C	18.2297
YDL140C	YOR224C	18.2218
YNL222W	YOR179C	18.2108
YDL160C	YNL147W	18.2047
YLR347C	YOR098C	18.2002
YJL026W	YOR230W	18.1943
YFL049W	YPR034W	18.1874
YGL098W	YOR075W	18.1815
YHR099W	YOL148C	18.1717
YDR378C	YHR165C	18.1665
YCR073W-A	YLR028C	18.161
YCR057C	YMR093W	18.1602
YDL045W-A	YNL306W	18.1586
YEL056W	YNL261W	18.1573
YML025C	YMR024W	18.149
YAL029C	YBR109C	18.1431
YDR060W	YLR002C	18.1383
YFL023W	YLR200W	18.1345
YCL016C	YHR191C	18.1184
YKL012W	YPR178W	18.1162
YLR086W	YLR272C	18.1161
YMR112C	YOL051W	18.1147
YNL151C	YOR224C	18.1059
YHR216W	YML056C	18.104
YDL031W	YLL008W	18.1038
YOL005C	YPR187W	18.1022
YDR328C	YKR014C	18.1008
YMR290C	YOR204W	18.0938
YDR324C	YLR409C	18.0801
YBR231C	YLR399C	18.0697
YHL025W	YPR034W	18.0696
YGL207W	YOR061W	18.0657
YNL222W	YNL317W	18.0656
YDR240C	YLR298C	18.0628
YBL090W	YJR101W	18.0558
YFL024C	YJR082C	18.0307
YER029C	YJL203W	18.029
YDR028C	YKL193C	18.0138
YDR041W	YMR158W	18.0127
YER177W	YFR017C	18.0098
YOR007C	YOR164C	18.0048
YBL003C	YBR010W	17.9878
YDR485C	YLR085C	17.9787
YBR221C	YFL018C	17.9775
YLR386W	YNL325C	17.9721
YDR080W	YPL045W	17.9649
YGR043C	YLR354C	17.9643
YBR253W	YOL135C	17.9613
YDR473C	YLR147C	17.9578
YGL241W	YML069W	17.9504
YGL011C	YLR263W	17.9438
YAL053W	YGL011C	17.9438
YGR074W	YHR086W	17.9437
YDL229W	YNL236W	17.9348
YDR407C	YJL044C	17.9255
YJL148W	YPR187W	17.9119
YBR251W	YDR337W	17.9119
YER029C	YPR082C	17.8975
YDR228C	YOR250C	17.8974
YDR228C	YGL044C	17.8974
YHR165C	YPR082C	17.8966
YGR165W	YMR188C	17.8884
YGR161C	YOR014W	17.8811
YDL051W	YPL243W	17.8767
YIL093C	YNR037C	17.8671
YGR074W	YJR022W	17.8606
YER029C	YJR022W	17.8582
YCR012W	YLR044C	17.8569
YER029C	YHR086W	17.8538
YDL185W	YEL051W	17.8397
YBR253W	YPR168W	17.8387
YFR004W	YHL030W	17.8378
YFL038C	YKR014C	17.8235
YDR416W	YER172C	17.8234
YNR003C	YPR187W	17.8016
YBL026W	YLR147C	17.7945
YDR138W	YGL253W	17.7862
YLR075W	YNL178W	17.7837
YEL036C	YGR231C	17.7808
YOR224C	YOR341W	17.7639
YLR115W	YMR061W	17.7473
YGR253C	YLR263W	17.7459
YAL053W	YGR253C	17.7459
YJL011C	YPR187W	17.7418
YHL030W	YOR259C	17.7418
YFR037C	YKR001C	17.7395
YHR041C	YOL135C	17.7384
YDL098C	YPR082C	17.7384
YNL059C	YOR141C	17.735
YDR385W	YGR187C	17.7325
YML069W	YNL031C	17.7287
YGL150C	YNL059C	17.7245
YCL008C	YGR206W	17.7172
YHR165C	YPL213W	17.7127
YDR245W	YGR132C	17.6981
YER082C	YPL126W	17.6874
YKR001C	YOR304W	17.6784
YGR186W	YOR224C	17.6779
YBL090W	YBR146W	17.6745
YDR299W	YNL132W	17.6712
YLR052W	YPL129W	17.6693
YIL035C	YJL115W	17.667
YDR028C	YER177W	17.6668
YKR006C	YLR439W	17.6612
YGR103W	YNL002C	17.6603
YDR235W	YLR298C	17.6598
YDR324C	YDR398W	17.6353
YJR033C	YPR036W	17.6322
YDR469W	YKL018W	17.6166
YLR347C	YPL020C	17.6151
YIL061C	YPR057W	17.6127
YDR036C	YPL118W	17.6111
YDR036C	YNL306W	17.6111
YDR036C	YHL004W	17.6111
YBR146W	YDR036C	17.6111
YKL012W	YPR057W	17.6044
YBR245C	YFR037C	17.6017
YJL109C	YLR222C	17.5963
YFL018C	YIL125W	17.5901
YDR301W	YNL222W	17.5811
YLR071C	YOL051W	17.5769
YNL248C	YOR210W	17.574
YMR223W	YOL148C	17.5719
YDR054C	YGL252C	17.5645
YLR263W	YOR362C	17.5546
YAL053W	YOR362C	17.5546
YDL097C	YHL030W	17.5456
YCR002C	YJR092W	17.5425
YBR107C	YPL018W	17.5399
YGL207W	YOR123C	17.5388
YDR398W	YMR093W	17.5244
YBL097W	YLR086W	17.5143
YKR048C	YOL012C	17.514
YDR517W	YGL011C	17.5121
YDR012W	YOR063W	17.5025
YMR290C	YNL002C	17.4936
YHR174W	YLR044C	17.493
YCR077C	YNL118C	17.4805
YLR095C	YMR044W	17.4789
YBL090W	YDR041W	17.4717
YLR035C	YMR167W	17.4678
YLR147C	YPR182W	17.4651
YDR172W	YOR061W	17.4573
YHR122W	YNL240C	17.4549
YAL032C	YPL151C	17.4438
YLR275W	YPR082C	17.4388
YBR009C	YMR284W	17.4349
YGR002C	YNL136W	17.4335
YGL011C	YJR051W	17.4296
YBL090W	YPL118W	17.4274
YOR151C	YOR210W	17.4203
YDR443C	YOL135C	17.4165
YFR031C	YLR272C	17.4158
YJL115W	YMR308C	17.4143
YER102W	YPR041W	17.4121
YGL048C	YHL030W	17.4106
YKL210W	YMR093W	17.4103
YML015C	YMR227C	17.4096
YBR009C	YDL126C	17.3917
YDR292C	YKL154W	17.3848
YCR012W	YGR192C	17.3761
YGR132C	YJL183W	17.3737
YGL111W	YMR049C	17.3671
YOR224C	YOR340C	17.3612
YBL074C	YDR473C	17.3556
YGL151W	YPL042C	17.3314
YDR116C	YDR296W	17.3242
YDR188W	YJR064W	17.3238
YKL144C	YNL113W	17.318
YBR114W	YMR201C	17.3151
YBR114W	YER162C	17.3151
YDR517W	YGR253C	17.3146
YOL077C	YOR272W	17.3114
YJL115W	YPL153C	17.3103
YGL111W	YNL002C	17.3101
YJL047C	YJR090C	17.3086
YDL159W	YPL049C	17.3044
YBR125C	YDR186C	17.2997
YDL030W	YLR117C	17.2925
YCR081W	YOL135C	17.2894
YHR086W	YLR147C	17.2843
YDR167W	YGR252W	17.2837
YDL209C	YPL151C	17.2776
YJR063W	YOR210W	17.265
YDR334W	YLR085C	17.2637
YDL098C	YGR074W	17.2617
YLR002C	YOR206W	17.2609
YDR517W	YPR181C	17.2588
YBR135W	YDL155W	17.2585
YML010W	YOL005C	17.2538
YBR095C	YDL076C	17.2508
YDL031W	YMR049C	17.2433
YKL060C	YKL152C	17.2357
YGL128C	YKL173W	17.2342
YDR167W	YDR392W	17.2323
YGR253C	YJR051W	17.2321
YGR130C	YMR031C	17.2297
YDR036C	YJR113C	17.2264
YKR085C	YMR193W	17.2126
YBL090W	YHL004W	17.2066
YDR118W	YOR249C	17.2052
YHR165C	YPL151C	17.2047
YJR101W	YOR158W	17.2007
YDR324C	YLR129W	17.2003
YDR378C	YJL124C	17.1948
YDR328C	YJR033C	17.1896
YDR148C	YFL018C	17.1873
YAL038W	YLL024C	17.1854
YDL047W	YLR179C	17.1853
YBR107C	YDR254W	17.1813
YJR058C	YOL062C	17.1811
YBR196C	YLR044C	17.1733
YCR020W-B	YLR321C	17.1696
YDL147W	YDR363W-A	17.1686
YDR036C	YKL155C	17.1651
YDR036C	YGR084C	17.1651
YDR036C	YGL129C	17.1651
YGR005C	YOR224C	17.161
YOR308C	YPR082C	17.1593
YBL090W	YKL155C	17.1563
YGR215W	YJR101W	17.1526
YDR188W	YJL014W	17.1456
YDR429C	YJR007W	17.1402
YDL031W	YLR002C	17.1302
YOR039W	YOR123C	17.1274
YAL002W	YLR148W	17.1272
YDR517W	YOR362C	17.1236
YLR277C	YMR061W	17.1221
YOR174W	YPL042C	17.1156
YHR052W	YMR049C	17.1061
YMR309C	YPL237W	17.1052
YLL008W	YMR290C	17.1041
YDR299W	YER127W	17.0964
YHR081W	YOR076C	17.0822
YGL066W	YMR223W	17.0814
YLR221C	YOL144W	17.0578
YNL306W	YOR158W	17.0565
YLR275W	YPR182W	17.0506
YJR051W	YOR362C	17.0412
YKL022C	YNL172W	17.0291
YNL189W	YOL021C	17.0237
YGL076C	YPL198W	17.0212
YDR156W	YJR063W	17.02
YBR245C	YGL133W	17.0165
YGR215W	YNL306W	17.0083
YGL066W	YPL011C	16.9995
YDR388W	YPL249C	16.9973
YHL047C	YMR227C	16.9961
YER025W	YLR192C	16.9865
YFL039C	YLR052W	16.9765
YLL045C	YOR312C	16.9717
YGR220C	YML025C	16.9653
YBL026W	YPR082C	16.9563
YBR142W	YMR049C	16.948
YKL188C	YPR178W	16.9453
YHR146W	YPL154C	16.9441
YIL061C	YOR159C	16.9379
YGR002C	YLR399C	16.9308
YHL030W	YIL075C	16.9143
YDR156W	YPR187W	16.9142
YDL209C	YDR364C	16.9125
YGR162W	YPL178W	16.9109
YBR114W	YKL011C	16.9102
YMR224C	YNL250W	16.9098
YDR363W-A	YLR421C	16.9047
YCR020W-B	YLR357W	16.8982
YBR253W	YOL051W	16.8931
YKR048C	YMR139W	16.8872
YJR102C	YLR417W	16.8864
YLL026W	YPL048W	16.8844
YDL076C	YNL097C	16.8806
YLR287C	YLR340W	16.8798
YGL120C	YKR022C	16.872
YDL131W	YML063W	16.8583
YPL151C	YPR101W	16.8569
YBL090W	YDR347W	16.8527
YML025C	YNL252C	16.8421
YDR116C	YML025C	16.8387
YGR186W	YOR210W	16.8386
YER112W	YNL147W	16.836
YGR278W	YKL173W	16.8358
YBR031W	YLL045C	16.8256
YBL097W	YFR031C	16.8142
YOR224C	YPL225W	16.8078
YBR094W	YNL118C	16.8064
YNL049C	YPL085W	16.8057
YDR416W	YKL173W	16.797
YDL031W	YMR290C	16.7934
YJR052W	YPL046C	16.786
YBL079W	YMR153W	16.7826
YDR369C	YNL250W	16.7771
YLR147C	YMR288W	16.7739
YHL030W	YHR027C	16.7731
YGL129C	YOR158W	16.7728
YBR146W	YGR215W	16.7708
YGR020C	YJR033C	16.7577
YBR221C	YOR362C	16.743
YDR175C	YMR188C	16.7385
YNL110C	YPL093W	16.7285
YDR080W	YMR231W	16.7267
YDR080W	YLR148W	16.7267
YER006W	YMR049C	16.7234
YAL003W	YPR080W	16.7202
YDR318W	YDR383C	16.7159
YBR194W	YPR152C	16.7139
YGL111W	YHR088W	16.7069
YDR308C	YDR443C	16.7043
YDL031W	YGL111W	16.7019
YDR036C	YDR347W	16.701
YLR222C	YMR093W	16.6998
YER136W	YKR014C	16.6986
YBR107C	YBR211C	16.6979
YDR237W	YLR439W	16.6971
YDR156W	YNL248C	16.6958
YGR220C	YNL252C	16.6839
YDR138W	YNL004W	16.68
YBL074C	YBR055C	16.6759
YJL124C	YLR438C-A	16.6725
YKL144C	YOR224C	16.6648
YDL002C	YPL129W	16.6608
YGR063C	YOR151C	16.6585
YLR208W	YPL085W	16.6528
YGL207W	YMR308C	16.6514
YGL025C	YLR071C	16.6451
YGL043W	YOR151C	16.6366
YER112W	YGR091W	16.6351
YGR262C	YKR038C	16.6275
YJL109C	YLR409C	16.6242
YLR312W-A	YMR024W	16.6227
YBR109C	YML057W	16.6214
YDL076C	YPL181W	16.6205
YDL003W	YIL026C	16.6168
YKL204W	YOL139C	16.6143
YCR081W	YDR308C	16.6142
YCR033W	YHR042W	16.613
YJR050W	YLL036C	16.6089
YDR036C	YIL093C	16.6036
YMR146C	YOR204W	16.6005
YGR195W	YNL189W	16.598
YHR052W	YHR088W	16.5971
YFL034W	YPL259C	16.5937
YNL037C	YOR136W	16.5917
YGR135W	YKL206C	16.5905
YNL189W	YPL020C	16.589
YGL172W	YGR119C	16.5823
YML041C	YPL235W	16.5819
YBR193C	YDR443C	16.5816
YIL103W	YOR133W	16.5668
YLR268W	YNL258C	16.5638
YLR268W	YLR440C	16.5638
YHR052W	YMR290C	16.5628
YAL035W	YBR079C	16.5498
YDL076C	YMR263W	16.5492
YBR119W	YMR125W	16.5486
YER164W	YOR039W	16.5484
YBR231C	YFL039C	16.545
YCL030C	YGR052W	16.5392
YJL014W	YJR064W	16.5386
YER112W	YPR178W	16.5367
YBR247C	YKL143W	16.5203
YAR007C	YOL090W	16.5197
YDR228C	YMR061W	16.5119
YHR052W	YNL002C	16.5054
YBR231C	YGR002C	16.4985
YOL051W	YOL135C	16.4916
YOR151C	YPR187W	16.4903
YNL113W	YOR210W	16.4886
YDL159W	YHR084W	16.4866
YBR152W	YGR075C	16.4701
YLR002C	YMR049C	16.4674
YMR169C	YMR170C	16.4626
YBL090W	YNL137C	16.4624
YDR028C	YER027C	16.4612
YDR394W	YHL030W	16.4561
YNL002C	YNL061W	16.4555
YBR193C	YCR081W	16.4551
YDL031W	YHR088W	16.4548
YOL006C	YOR061W	16.4522
YGL098W	YGL145W	16.4512
YDL209C	YLR117C	16.4509
YER122C	YNL287W	16.4484
YAL032C	YJR050W	16.4425
YLR319C	YOR304C-A	16.4317
YGL194C	YHR042W	16.4197
YML025C	YNL005C	16.4187
YIL035C	YOL054W	16.3977
YDR378C	YKL173W	16.3962
YDR334W	YJL081C	16.3925
YFR037C	YPL082C	16.3907
YGR129W	YLR117C	16.3891
YBR221C	YGL011C	16.3889
YCR012W	YOL086C	16.3844
YBL026W	YHR165C	16.3785
YGL127C	YOR174W	16.3773
YMR112C	YNL236W	16.3699
YOR224C	YPR010C	16.3559
YJR002W	YLR197W	16.3549
YPR010C	YPR190C	16.3545
YBR055C	YML046W	16.3396
YPL016W	YPR034W	16.3375
YNL178W	YOR063W	16.3224
YOR224C	YPR190C	16.3203
YHR090C	YOR244W	16.3153
YFL034W	YPR029C	16.3083
YDR254W	YDR383C	16.3037
YDR118W	YNL172W	16.292
YGR074W	YPR182W	16.2915
YCR071C	YDR405W	16.2857
YAR007C	YML032C	16.2854
YGR215W	YPL118W	16.2802
YFL013C	YPL129W	16.2704
YAL038W	YHR174W	16.2664
YDR363W-A	YFR052W	16.2653
YER094C	YGR161C	16.2636
YDL134C	YOR014W	16.2635
YGL145W	YOR075W	16.2578
YHR099W	YLR055C	16.2577
YDR190C	YLR399C	16.2562
YDR036C	YNL137C	16.2522
YDR176W	YML015C	16.2488
YBR079C	YER025W	16.2484
YKL173W	YMR213W	16.2473
YGL133W	YPL082C	16.2439
YDR296W	YLR439W	16.2416
YJR063W	YPR187W	16.2291
YLR071C	YMR112C	16.2287
YDR405W	YML025C	16.2262
YDR334W	YDR485C	16.2246
YDL076C	YOL004W	16.2193
YGL111W	YLL008W	16.2162
YNL189W	YOR272W	16.216
YCL010C	YMR236W	16.2137
YDR404C	YGL043W	16.2055
YHR088W	YOR272W	16.2053
YKR002W	YMR061W	16.2035
YLR355C	YPL225W	16.2004
YML063W	YOR063W	16.1965
YBR247C	YDL060W	16.1926
YJL109C	YLR129W	16.1922
YHR056C	YIL126W	16.192
YIR006C	YNL084C	16.1864
YGR165W	YJR101W	16.1676
YCR020W-B	YPR034W	16.1646
YIR003W	YKL007W	16.1593
YDL134C	YML109W	16.1503
YDR378C	YJR022W	16.1491
YDR238C	YER122C	16.1473
YHR056C	YMR091C	16.1429
YCR046C	YLR439W	16.1242
YCR009C	YMR192W	16.1189
YBR251W	YJR101W	16.1054
YGR120C	YNL051W	16.104
YFR002W	YJL041W	16.0931
YDL148C	YPR144C	16.0818
YJR050W	YMR213W	16.0699
YNL035C	YOL123W	16.0674
YNL002C	YPL093W	16.063
YFL018C	YFR049W	16.0625
YDR404C	YOR210W	16.0608
YHR165C	YJL203W	16.0569
YCR071C	YNL284C	16.0547
YBR221C	YER094C	16.0489
YGR240C	YOR138C	16.0477
YDL132W	YDL216C	16.0446
YGR180C	YOR230W	16.0427
YJR022W	YLR147C	16.0409
YJL140W	YPL129W	16.0288
YBR109C	YOR035C	16.0285
YJR145C	YLL045C	16.0116
YML109W	YMR273C	16.0085
YDL115C	YPR187W	16.0084
YMR076C	YOL054W	16.0046
YLR147C	YPL213W	15.9824
YER021W	YMR314W	15.9819
YDL126C	YNL206C	15.9803
YDR363W-A	YOR261C	15.9712
YFL039C	YGL150C	15.9552
YDR296W	YDR322W	15.9534
YBL026W	YCR077C	15.9448
YGR103W	YPR016C	15.9432
YHR056C	YPR034W	15.9378
YBR055C	YPL213W	15.9353
YDL108W	YML063W	15.9163
YDR280W	YNL189W	15.915
YGL122C	YNL035C	15.9137
YBR017C	YNL035C	15.9137
YDR359C	YNL136W	15.913
YKL173W	YPR101W	15.9083
YLR347C	YOR272W	15.9014
YDR240C	YLR275W	15.9008
YDL216C	YDR328C	15.8884
YER172C	YGL128C	15.8843
YDL209C	YGR278W	15.8841
YDR392W	YHR099W	15.8737
YLR002C	YNL061W	15.8735
YDL031W	YHR052W	15.8628
YNL002C	YOL077C	15.8578
YLR442C	YNL189W	15.8564
YER095W	YML032C	15.8522
YNL059C	YPL235W	15.8478
YCR071C	YKR006C	15.8454
YAL002W	YLR396C	15.8377
YCR046C	YDR322W	15.8362
YOR035C	YOR326W	15.8317
YHL030W	YPR108W	15.8277
YDR121W	YOR304W	15.8216
YBR119W	YER029C	15.8159
YBR245C	YKR001C	15.8093
YBR154C	YJR063W	15.8089
YHR088W	YMR049C	15.8009
YDR172W	YOR039W	15.8005
YDR240C	YLR147C	15.7996
YBR105C	YCL039W	15.7981
YDR449C	YPL126W	15.7894
YBL074C	YHR165C	15.7846
YDL145C	YER122C	15.7841
YNR003C	YOR224C	15.7817
YBL038W	YKR006C	15.773
YHR088W	YLL008W	15.7705
YNR037C	YOR158W	15.7522
YOR290C	YPR034W	15.7501
YLR275W	YLR298C	15.7497
YJR063W	YNL113W	15.7423
YDR378C	YER029C	15.7363
YMR158W	YOR158W	15.7315
YHL047C	YMR005W	15.7309
YDR036C	YDR337W	15.727
YER136W	YOR370C	15.7247
YDR364C	YDR416W	15.7247
YHR088W	YMR290C	15.724
YML049C	YPR178W	15.7206
YDL143W	YDR188W	15.7113
YDR416W	YPR101W	15.7088
YHL027W	YJL173C	15.7024
YGR215W	YNL137C	15.7017
YBR231C	YLR085C	15.6967
YFL024C	YNL136W	15.6966
YDR334W	YNL107W	15.6747
YEL018W	YLR347C	15.6743
YDR235W	YER029C	15.6687
YDR176W	YHR099W	15.6654
YGR158C	YHR081W	15.6619
YDR328C	YJL047C	15.6616
YDR429C	YER025W	15.6533
YFR024C-A	YNL243W	15.6478
YCR020W-B	YFR037C	15.6079
YML069W	YMR076C	15.6042
YDL043C	YER029C	15.597
YCR057C	YPR144C	15.5949
YHR052W	YOL077C	15.5897
YLR360W	YPL120W	15.5849
YLR240W	YPL120W	15.5849
YLR240W	YLR360W	15.5849
YIL143C	YOR352W	15.5849
YER107C	YMR047C	15.5849
YOR001W	YPR190C	15.5743
YHR041C	YOL051W	15.5719
YDL156W	YOL054W	15.5596
YBR198C	YHR099W	15.5506
YCL011C	YGL173C	15.5469
YIL035C	YMR172W	15.5376
YLR315W	YPR046W	15.5328
YBL008W	YPL153C	15.53
YKL018W	YOR179C	15.5288
YMR112C	YOL135C	15.527
YJR002W	YMR093W	15.5236
YDR449C	YJR002W	15.5236
YML115C	YPL050C	15.5177
YBL064C	YDR488C	15.5177
YLL008W	YOL077C	15.5086
YNL002C	YNL110C	15.5074
YMR146C	YNL244C	15.5054
YCR093W	YGR134W	15.504
YML063W	YOR038C	15.498
YGL070C	YML010W	15.4857
YIL070C	YJL115W	15.4749
YHR216W	YLR432W	15.4734
YNL031C	YOL054W	15.4701
YJL148W	YJR063W	15.4694
YDR310C	YOR279C	15.4692
YER174C	YGR262C	15.4686
YAR019C	YML064C	15.4546
YDR443C	YHR058C	15.4527
YBR102C	YPR055W	15.4435
YFR037C	YGL078C	15.4429
YJL146W	YPL154C	15.4412
YDR080W	YLR396C	15.4376
YBL041W	YFL007W	15.4317
YLR199C	YMR314W	15.4298
YEL055C	YGR128C	15.4284
YKL173W	YLR424W	15.4249
YOR207C	YOR224C	15.4239
YGR231C	YPL050C	15.4185
YBL084C	YOR249C	15.4025
YKL023W	YLR398C	15.3947
YDR227W	YLR442C	15.3922
YBL071W-A	YIL103W	15.3775
YHR090C	YPR023C	15.3766
YAL011W	YFL039C	15.3674
YIL106W	YPR111W	15.3628
YBR127C	YEL051W	15.3567
YOL135C	YPL042C	15.3439
YBR154C	YGL043W	15.3363
YBR167C	YHR062C	15.3343
YIL021W	YPL129W	15.3334
YNL113W	YOR224C	15.328
YCR081W	YHR058C	15.3272
YBR060C	YEL056W	15.3131
YHR099W	YOR244W	15.3105
YNL189W	YOR076C	15.3065
YER171W	YIL128W	15.3064
YLR166C	YPR055W	15.3043
YDL115C	YOR224C	15.298
YER117W	YPL208W	15.2965
YDR361C	YER117W	15.2965
YDL195W	YKR082W	15.2866
YDL195W	YHL039W	15.2866
YER006W	YHR197W	15.2851
YJR022W	YMR268C	15.2824
YBR031W	YGR128C	15.2711
YGL025C	YGL151W	15.2672
YHL019C	YLR170C	15.2669
YCR057C	YPL126W	15.258
YDR363W-A	YFR010W	15.2535
YCR071C	YDR322W	15.2531
YLR147C	YNL147W	15.2424
YGL111W	YOL077C	15.2388
YFL007W	YPR103W	15.2327
YLR275W	YOR308C	15.2271
YDR016C	YGL061C	15.2175
YGR074W	YGR091W	15.2144
YDR028C	YDR477W	15.2101
YGR253C	YKL206C	15.2003
YMR288W	YPL151C	15.1999
YKL173W	YPR182W	15.1814
YDR359C	YFL039C	15.1754
YBL017C	YHR027C	15.1699
YLR432W	YML056C	15.1577
YGL111W	YOR206W	15.1522
YGR005C	YML010W	15.1492
YEL018W	YNL136W	15.1446
YBL072C	YER102W	15.1402
YOR159C	YPR182W	15.1391
YDR028C	YGL115W	15.136
YLL036C	YLR424W	15.135
YDR116C	YMR193W	15.1321
YDR138W	YDR381W	15.1309
YDR416W	YML049C	15.1275
YLR347C	YPR023C	15.1152
YBR251W	YNR037C	15.1079
YGL011C	YGR161C	15.1077
YGL025C	YHR041C	15.1041
YLR075W	YOR063W	15.0985
YBL090W	YMR188C	15.0979
YBR211C	YPR046W	15.0873
YDR424C	YDR488C	15.0854
YDL185W	YJR121W	15.0833
YDL111C	YNL189W	15.081
YLR055C	YML015C	15.0805
YGR056W	YGR275W	15.0782
YFR013W	YLR095C	15.0742
YBR258C	YPL138C	15.0686
YBL090W	YDL045W-A	15.0672
YHR056C	YLR357W	15.0643
YMR061W	YNL317W	15.0577
YDR443C	YPL042C	15.0507
YBR221C	YML092C	15.0487
YJR148W	YLL048C	15.0444
YLR399C	YPL235W	15.0413
YLR263W	YML092C	15.0361
YAL053W	YML092C	15.0361
YBR233W-A	YGL061C	15.0243
YHR165C	YMR240C	15.0227
YAL043C	YMR061W	15.0219
YDR363W-A	YER021W	15.0179
YBR258C	YLR015W	15.011
YEL022W	YGR218W	15.007
YGL233W	YLR166C	15.0047
YOL115W	YPL190C	15.0003
YKL190W	YLR433C	14.9983
YGL070C	YOR224C	14.9956
YAL011W	YGR002C	14.9873
YOR254C	YPL094C	14.9872
YLR292C	YPL094C	14.9872
YBR171W	YPL094C	14.9872
YDR311W	YDR460W	14.987
YBR055C	YGR075C	14.9829
YGR103W	YNL061W	14.9795
YPR025C	YPR056W	14.9788
YBL090W	YOR158W	14.9786
YBR055C	YJL124C	14.977
YDR462W	YLR439W	14.9725
YGR220C	YMR193W	14.9677
YDL047W	YKR028W	14.9661
YDR101C	YHR197W	14.9654
YDR246W	YML077W	14.965
YDL188C	YGR161C	14.962
YLL008W	YNL002C	14.9545
YHL019C	YPL259C	14.9504
YBR088C	YGL019W	14.9458
YCR081W	YPL042C	14.942
YER006W	YNL002C	14.9414
YBL090W	YGR215W	14.9386
YHR052W	YOR272W	14.9379
YFL005W	YPR017C	14.9378
YDR364C	YLL036C	14.9293
YDL031W	YER006W	14.9194
YBR288C	YJL024C	14.9163
YGR161C	YGR253C	14.9126
YKR048C	YML069W	14.9058
YDR378C	YER146W	14.9023
YDR364C	YGR278W	14.9016
YCR063W	YMR213W	14.888
YKL012W	YPL213W	14.8855
YDR443C	YHR041C	14.8823
YER029C	YLR298C	14.882
YDR054C	YJL047C	14.8805
YER082C	YHR148W	14.8775
YHR165C	YPR182W	14.8731
YHR165C	YIL061C	14.8714
YDR443C	YOR174W	14.8641
YDR190C	YNL059C	14.863
YGR135W	YLR263W	14.8626
YAL053W	YGR135W	14.8626
YGR017W	YML056C	14.8613
YJR041C	YKL014C	14.8535
YLR263W	YOL038W	14.8526
YAL053W	YOL038W	14.8526
YLR117C	YML049C	14.8518
YFL002C	YOR272W	14.8501
YDL132W	YOL117W	14.8453
YDL014W	YDR496C	14.844
YBL041W	YLR263W	14.8426
YAL053W	YBL041W	14.8426
YLR147C	YPR082C	14.8419
YGR084C	YPL013C	14.8369
YBR031W	YOR312C	14.8358
YCL024W	YDR507C	14.8255
YPL011C	YPL254W	14.8107
YHR099W	YNL107W	14.8087
YNR003C	YOR340C	14.8046
YBR055C	YKL188C	14.802
YER164W	YGL019W	14.7946
YNL136W	YPR023C	14.7881
YBL099W	YJR121W	14.7826
YNL002C	YOR206W	14.7773
YGR135W	YLR199C	14.7693
YKL196C	YMR197C	14.7691
YFL049W	YPL016W	14.7587
YDR427W	YLR278C	14.7574
YCR081W	YHR041C	14.7574
YBL090W	YGR165W	14.7565
YDL140C	YOR210W	14.7557
YDR049W	YKL204W	14.7554
YEL002C	YMR149W	14.755
YML069W	YOR123C	14.7512
YJR101W	YMR158W	14.7508
YGR104C	YOL051W	14.7501
YBL017C	YOR181W	14.7484
YBL017C	YNR065C	14.7484
YBL017C	YLR337C	14.7484
YBL017C	YLR081W	14.7484
YBL017C	YIR012W	14.7484
YDR108W	YML077W	14.7474
YDR108W	YDR246W	14.7474
YDR235W	YLR275W	14.7453
YGL233W	YIL068C	14.7445
YJR041C	YOL144W	14.7444
YBL074C	YER029C	14.7432
YDL076C	YNL330C	14.7409
YCR081W	YOR174W	14.7393
YNL061W	YPL093W	14.7356
YBR258C	YDR469W	14.735
YER082C	YJL109C	14.7346
YAL062W	YOR375C	14.7317
YFL002C	YMR290C	14.7264
YGR161C	YOR362C	14.7241
YIL061C	YPL178W	14.7177
YBR107C	YGR179C	14.7157
YIL046W	YJL047C	14.7095
YDL164C	YJL047C	14.7095
YDR267C	YOL111C	14.7069
YHR052W	YPL211W	14.7023
YGR245C	YHR197W	14.7004
YBR221C	YGR253C	14.6996
YDL076C	YPL139C	14.6966
YML098W	YMR005W	14.6863
YDR517W	YML092C	14.6791
YCR071C	YNL005C	14.6724
YER122C	YGL137W	14.6668
YDR237W	YDR296W	14.6622
YDR116C	YDR237W	14.6622
YOR157C	YPR103W	14.6601
YKL060C	YOL086C	14.6573
YLR424W	YPL151C	14.6541
YDR235W	YLR147C	14.6451
YOR210W	YPR010C	14.6432
YDL031W	YGR103W	14.6387
YOR027W	YPL240C	14.6317
YHR023W	YPR188C	14.6278
YBR273C	YGR048W	14.6207
YDR202C	YEL051W	14.62
YDR036C	YMR188C	14.6199
YER006W	YNR053C	14.6152
YJR051W	YML092C	14.6107
YMR186W	YOL086C	14.6072
YGR103W	YNL110C	14.6041
YDL156W	YNL030W	14.6039
YDR363W-A	YKL145W	14.6017
YNL189W	YNL330C	14.6014
YDR398W	YEL055C	14.5997
YOR204W	YOR206W	14.5964
YKL173W	YMR268C	14.5928
YBR087W	YOR144C	14.5818
YDR207C	YMR263W	14.5796
YKR014C	YOR089C	14.5791
YGR002C	YLR385C	14.5786
YDR028C	YMR311C	14.5779
YMR112C	YPR168W	14.5766
YDR364C	YPL151C	14.5762
YBR079C	YNL244C	14.5746
YMR240C	YOR319W	14.5745
YJL081C	YLR399C	14.5694
YPR010C	YPR191W	14.5655
YMR158W	YNL306W	14.5648
YGL120C	YIL079C	14.5645
YMR188C	YOR158W	14.5618
YGR233C	YNL138W	14.5602
YFL002C	YGL111W	14.5525
YGL111W	YOR272W	14.5469
YDR404C	YOR224C	14.5458
YML077W	YMR218C	14.5445
YKR068C	YML077W	14.5445
YDR407C	YML077W	14.5445
YDR246W	YMR218C	14.5445
YDR246W	YKR068C	14.5445
YDR246W	YDR407C	14.5445
YBR254C	YML077W	14.5445
YBR254C	YDR246W	14.5445
YLR275W	YMR288W	14.5414
YFL013C	YNL059C	14.5351
YJR002W	YPR137W	14.5296
YLR430W	YNL251C	14.5241
YBR081C	YMR227C	14.5222
YBR055C	YML049C	14.5179
YOR304W	YPL082C	14.5167
YCR071C	YML025C	14.5094
YDR517W	YGR135W	14.5058
YJR063W	YOR224C	14.5048
YDR176W	YMR227C	14.5043
YDL175C	YPL190C	14.5034
YDL185W	YJR033C	14.498
YDR517W	YOL038W	14.4958
YBR122C	YDR237W	14.4954
YDR101C	YER006W	14.4923
YMR076C	YNL030W	14.4914
YCR071C	YJL063C	14.4905
YHR154W	YLR320W	14.4883
YDL215C	YHR154W	14.4883
YDL013W	YLR006C	14.4883
YBL041W	YDR517W	14.4859
YDL102W	YJR043C	14.4857
YER082C	YMR093W	14.4837
YPL013C	YPL118W	14.4795
YDL155W	YPR119W	14.4775
YMR240C	YPR178W	14.462
YER012W	YER021W	14.4618
YOL077C	YOR206W	14.4537
YDL031W	YNL061W	14.4511
YDL140C	YGR063C	14.448
YBR146W	YMR158W	14.446
YGR135W	YJR051W	14.4376
YJR051W	YOL038W	14.4276
YNL147W	YOR308C	14.4223
YGL106W	YKL130C	14.4218
YKL206C	YOR362C	14.4206
YDL031W	YPL211W	14.4202
YDR201W	YKR083C	14.4188
YPL093W	YPR016C	14.418
YJL109C	YMR128W	14.418
YBL041W	YJR051W	14.4176
YDR098C	YER174C	14.4144
YNR024W	YOL021C	14.4075
YOL006C	YOR039W	14.4067
YGR166W	YML077W	14.401
YDR246W	YGR166W	14.401
YLL024C	YPL106C	14.4002
YDR378C	YER112W	14.3959
YDR279W	YNL072W	14.395
YDR422C	YER027C	14.3895
YMR308C	YPL020C	14.3864
YDL002C	YNL059C	14.3858
YJR075W	YPL050C	14.3766
YBR231C	YJL081C	14.3715
YHR090C	YJR082C	14.3702
YML077W	YOR115C	14.3546
YDR472W	YML077W	14.3546
YDR246W	YDR472W	14.3546
YGL190C	YOR014W	14.3348
YCL010C	YHR099W	14.3344
YLR025W	YPR173C	14.3343
YDR267C	YNL240C	14.3308
YBR170C	YMR067C	14.3308
YER112W	YKL188C	14.3306
YNL306W	YNR037C	14.3294
YDR108W	YMR218C	14.327
YDR108W	YKR068C	14.327
YDR108W	YDR407C	14.327
YBR254C	YDR108W	14.327
YLR288C	YPL194W	14.3239
YDR416W	YGL128C	14.321
YCL010C	YDR167W	14.3183
YCR077C	YDR473C	14.3133
YKL182W	YPL231W	14.3075
YJL203W	YLR147C	14.3072
YJR002W	YPL126W	14.2881
YNL032W	YNL056W	14.2877
YLR117C	YMR288W	14.2845
YGL172W	YJL041W	14.2802
YDL031W	YFL002C	14.2771
YER027C	YIL095W	14.2644
YDR422C	YIL095W	14.2644
YDR017C	YNL267W	14.2638
YJL063C	YKR085C	14.2607
YDR296W	YNL252C	14.2581
YDR116C	YNL252C	14.2581
YLR298C	YMR125W	14.2438
YNL189W	YNL232W	14.2412
YGL197W	YJL098W	14.2354
YDL148C	YPL217C	14.2162
YNL132W	YOR204W	14.2113
YBL074C	YPR178W	14.2089
YMR158W	YPL118W	14.2001
YGR084C	YMR158W	14.1989
YKL113C	YMR308C	14.1966
YOR312C	YPL198W	14.1885
YDR314C	YDR427W	14.1846
YDR237W	YML025C	14.184
YDR108W	YGR166W	14.1835
YNL004W	YNL139C	14.1791
YKR014C	YOR370C	14.1756
YNL137C	YPL013C	14.1742
YCR046C	YNL252C	14.1601
YPL166W	YPR049C	14.1599
YOR144C	YOR217W	14.1579
YCR020W-B	YMR033W	14.154
YFR040W	YMR028W	14.1504
YMR308C	YOL054W	14.1482
YIL079C	YJL050W	14.1451
YDR028C	YLR028C	14.1413
YDR443C	YNR010W	14.1404
YBR253W	YDR443C	14.1404
YGL022W	YJL002C	14.1393
YAL059W	YDR395W	14.1363
YGR054W	YHR013C	14.1351
YDL031W	YHR013C	14.1351
YDR416W	YMR288W	14.125
YBR127C	YJR033C	14.1195
YFR015C	YJL137C	14.1117
YER172C	YLR424W	14.1023
YDR392W	YMR236W	14.0915
YBR136W	YNL312W	14.0915
YLR186W	YPR144C	14.0744
YBL018C	YNL282W	14.0637
YAL033W	YNL282W	14.0637
YDR175C	YNR037C	14.0613
YER022W	YGL127C	14.0591
YLR002C	YNL002C	14.0564
YDR324C	YJL069C	14.0552
YDR101C	YGR103W	14.0526
YCR020W-B	YMR091C	14.0459
YKR037C	YKR083C	14.0425
YMR314W	YOR261C	14.0421
YBR135W	YPL256C	14.0396
YHL004W	YPL013C	14.0371
YNL030W	YNL031C	14.0328
YCR081W	YNR010W	14.0323
YBR253W	YCR081W	14.0323
YFL039C	YHR090C	14.0217
YHR013C	YMR290C	14.0216
YKL155C	YMR158W	14.0173
YDL025C	YHR009C	14.0139
YAL049C	YHR009C	14.0139
YCL059C	YGR017W	14.0099
YJL183W	YJR075W	14.0033
YNL059C	YPL129W	14
YJL148W	YOR210W	13.9976
YML025C	YPL173W	13.9935
YGR013W	YPL178W	13.9899
YCR073W-A	YMR120C	13.9897
YDR324C	YJR002W	13.9894
YGL025C	YGR104C	13.989
YDR322W	YMR193W	13.9886
YOR204W	YPR041W	13.9831
YDR496C	YNL061W	13.9831
YHL004W	YMR158W	13.9806
YDR041W	YPL013C	13.9802
YDR364C	YLR117C	13.979
YBR017C	YKL009W	13.9775
YDR298C	YKL016C	13.9758
YNL132W	YPL126W	13.9746
YBR188C	YJR050W	13.9715
YOR145C	YPL266W	13.9546
YAL011W	YBR231C	13.9501
YIL076W	YPL219W	13.9439
YER022W	YPL106C	13.9437
YGL150C	YPL129W	13.9423
YBR142W	YHR052W	13.942
YER157W	YNL051W	13.9384
YLR208W	YPR181C	13.9351
YJL111W	YOR281C	13.9302
YBL018C	YBR257W	13.9302
YDR012W	YLR075W	13.923
YDR167W	YLR055C	13.9227
YCR092C	YOL090W	13.9202
YGL129C	YMR158W	13.9198
YDL065C	YJL124C	13.9182
YLL036C	YML049C	13.9161
YDR427W	YLR359W	13.916
YDR427W	YKL165C	13.916
YDR427W	YJR120W	13.916
YDR427W	YJR062C	13.916
YDR167W	YOL148C	13.9158
YER031C	YKR014C	13.9146
YGL190C	YGR161C	13.9139
YDL044C	YOL031C	13.9104
YDR041W	YNR037C	13.9098
YKL196C	YOR075W	13.9085
YCR073C	YLR006C	13.9045
YAL042W	YML067C	13.9038
YCR012W	YKL152C	13.903
YJL138C	YOL139C	13.9004
YEL018W	YFL039C	13.8976
YBR119W	YPL178W	13.8948
YFR036W	YIR025W	13.8944
YIL093C	YMR158W	13.8851
YBR267W	YHR170W	13.8771
YJR082C	YNL107W	13.8669
YJL098W	YOR267C	13.8586
YKL018W	YMR061W	13.8553
YDL148C	YJR002W	13.8523
YLR197W	YPL126W	13.8495
YBR097W	YPL120W	13.8402
YBR097W	YLR360W	13.8402
YBR097W	YLR240W	13.8402
YDL209C	YKL173W	13.8387
YLR002C	YMR290C	13.8381
YEL056W	YLL004W	13.838
YBR154C	YML010W	13.8376
YDL031W	YPL093W	13.8365
YJR113C	YMR158W	13.8356
YDR296W	YNL005C	13.8347
YIL076W	YOL001W	13.8311
YDR449C	YLR197W	13.8311
YAL036C	YHR013C	13.829
YER133W	YLR028C	13.821
YER102W	YLR192C	13.8142
YER172C	YJL203W	13.8117
YEL056W	YPR162C	13.81
YDR036C	YDR041W	13.8077
YDR347W	YMR158W	13.8058
YFL002C	YHR052W	13.8054
YDR477W	YIL095W	13.8051
YGR275W	YML127W	13.802
YHR197W	YPL093W	13.7871
YKL155C	YNR037C	13.7817
YCR077C	YDL160C	13.7588
YER133W	YNL222W	13.7485
YBL037W	YOL062C	13.7472
YGL173C	YJL124C	13.7413
YNL189W	YPL139C	13.7244
YGR132C	YPL050C	13.7225
YMR290C	YPL093W	13.7218
YDR036C	YJR101W	13.7207
YBR109C	YLR429W	13.7207
YDR254W	YLR315W	13.7198
YCR046C	YNL005C	13.7191
YDR322W	YDR405W	13.717
YDR427W	YJL204C	13.7168
YDL147W	YHL030W	13.7129
YNL061W	YOR206W	13.7108
YBL056W	YER089C	13.7106
YGL133W	YKR001C	13.7018
YHR165C	YLR424W	13.6998
YBL026W	YGR074W	13.6943
YDR002W	YOR185C	13.6942
YNL236W	YPL106C	13.6908
YGL240W	YIR025W	13.6908
YGR274C	YML098W	13.6905
YNL248C	YOR224C	13.6878
YBL018C	YHR062C	13.6873
YDR485C	YLR385C	13.6865
YCL024W	YKR048C	13.6851
YCR071C	YMR024W	13.6838
YER120W	YHL020C	13.6815
YCR035C	YPR190C	13.6788
YHR090C	YLR399C	13.6783
YDL060W	YPL204W	13.6718
YJL109C	YPL217C	13.6616
YNL236W	YPR168W	13.6614
YHR058C	YPL042C	13.6592
YGR083C	YLR215C	13.6571
YBR221C	YOL038W	13.6552
YBR142W	YOR272W	13.6527
YBR245C	YMR044W	13.651
YGL106W	YHR023W	13.6497
YOL146W	YPR135W	13.6463
YDL014W	YGR017W	13.6445
YGR074W	YPR082C	13.6442
YDR378C	YKL188C	13.6433
YCR046C	YDR296W	13.6394
YCR046C	YDR116C	13.6394
YER172C	YPR182W	13.6323
YLR175W	YNL308C	13.6316
YAL017W	YOL045W	13.631
YGL066W	YHR099W	13.6302
YCR072C	YNR053C	13.6297
YJR022W	YPR178W	13.6236
YER164W	YIL035C	13.6208
YHR148W	YMR093W	13.6195
YGR091W	YLR275W	13.6185
YLR002C	YPL093W	13.6066
YOR272W	YPL093W	13.6056
YBR011C	YPL066W	13.6008
YBR031W	YMR093W	13.5978
YHR114W	YLR337C	13.5909
YDR378C	YLR275W	13.5858
YJL098W	YKR028W	13.5824
YBL058W	YBR223C	13.5821
YER102W	YHR013C	13.5803
YDR045C	YKL144C	13.5742
YDR012W	YNL178W	13.5716
YDR347W	YNR037C	13.5712
YDL209C	YPR101W	13.5684
YNL132W	YOR206W	13.568
YNL061W	YOL077C	13.5674
YGR278W	YPL151C	13.5583
YKR085C	YLR439W	13.5551
YBL090W	YDR175C	13.5515
YDR405W	YKR006C	13.5504
YDR233C	YPL154C	13.5502
YLR277C	YNL222W	13.5486
YBL071W-A	YKL191W	13.5417
YKL173W	YML046W	13.5415
YDR240C	YGR074W	13.5377
YMR128W	YOR206W	13.5336
YLR186W	YPL126W	13.5322
YGL151W	YPR168W	13.5321
YDR175C	YPL013C	13.5246
YEL056W	YIL035C	13.5228
YER006W	YGL111W	13.5207
YGR233C	YIL076W	13.5162
YNL113W	YOR116C	13.5158
YKL009W	YLR002C	13.5122
YMR158W	YNL137C	13.5047
YML091C	YPL204W	13.5024
YDR207C	YIL084C	13.5021
YDL007W	YDR363W-A	13.4997
YDL097C	YDR052C	13.4983
YDR334W	YFL039C	13.4979
YBL058W	YFL044C	13.4917
YGR123C	YMR186W	13.487
YMR205C	YOR138C	13.4859
YDR405W	YNL005C	13.4795
YDR299W	YGR145W	13.4764
YDR330W	YGR048W	13.476
YDR264C	YOR212W	13.4709
YDR405W	YNL284C	13.4702
YMR288W	YPR178W	13.4667
YLR044C	YLR249W	13.4622
YFR004W	YLR278C	13.4561
YER012W	YKL206C	13.4553
YFL002C	YMR049C	13.4513
YDL030W	YDR473C	13.4501
YBL093C	YBR253W	13.4466
YCR046C	YJL063C	13.4462
YKL095W	YLL036C	13.443
YLL010C	YLR019W	13.4378
YAR014C	YDR028C	13.4365
YBR055C	YDR240C	13.422
YDR131C	YDR328C	13.4203
YBR109C	YKL129C	13.4191
YDR162C	YJL128C	13.418
YJR045C	YLR203C	13.4131
YBR088C	YOR061W	13.3955
YDR027C	YJL029C	13.3936
YBR160W	YLR210W	13.3931
YLR197W	YMR093W	13.3929
YGL025C	YPL042C	13.3924
YIL035C	YPL001W	13.3883
YGL128C	YLL036C	13.3878
YJL061W	YMR047C	13.3867
YJR074W	YLR293C	13.3848
YJR002W	YMR128W	13.379
YDR398W	YPL126W	13.3764
YBL038W	YDR462W	13.3656
YBR247C	YGL122C	13.3604
YBR017C	YBR247C	13.3604
YLR439W	YNL252C	13.3593
YDL031W	YNL002C	13.3557
YCR057C	YGR090W	13.3555
YHR086W	YLR298C	13.3541
YDR449C	YGR090W	13.35
YFR040W	YJL098W	13.3492
YML069W	YMR308C	13.3429
YOL005C	YPL129W	13.338
YDR296W	YML025C	13.3353
YIR002C	YOL090W	13.3324
YDR378C	YPR082C	13.3257
YJL109C	YLR197W	13.3244
YJL023C	YML091C	13.3201
YGR245C	YNR053C	13.3168
YCR072C	YNL182C	13.3166
YDR322W	YKR085C	13.3155
YBR122C	YDR322W	13.3076
YGR013W	YPR182W	13.3025
YIL093C	YPL013C	13.2943
YLR197W	YOR078W	13.2938
YML063W	YNL178W	13.2921
YJL115W	YOR038C	13.2881
YBL038W	YDR322W	13.2871
YDR099W	YNL267W	13.2866
YDR224C	YKR048C	13.28
YFL039C	YOR244W	13.2739
YMR312W	YPL086C	13.2716
YCR088W	YDR129C	13.2688
YBR055C	YER112W	13.2606
YGL111W	YPL093W	13.258
YGR013W	YPR057W	13.2564
YHR081W	YNR024W	13.2543
YOL135C	YPR168W	13.2485
YGR090W	YPL126W	13.2406
YNL232W	YPR190C	13.24
YCR046C	YML025C	13.2379
YMR197C	YOR036W	13.2308
YJR033C	YKL080W	13.2308
YDR121W	YGL133W	13.221
YMR290C	YNL061W	13.2196
YER071C	YIL034C	13.2192
YLR409C	YMR093W	13.2182
YAL013W	YDL076C	13.2181
YER006W	YNL061W	13.218
YDR036C	YDR175C	13.2172
YKL206C	YML092C	13.2121
YHR164C	YNL312W	13.2121
YFL002C	YOR206W	13.2105
YDR405W	YPL173W	13.2089
YGR091W	YML049C	13.2064
YBR058C	YMR304W	13.2054
YDL108W	YDR460W	13.2041
YGL128C	YHR165C	13.1983
YDL065C	YMR284W	13.1918
YCR057C	YLR197W	13.1849
YCR072C	YGR245C	13.1828
YCR057C	YJR002W	13.1787
YBL017C	YLR447C	13.1748
YHR041C	YPL042C	13.1695
YJL047C	YLR320W	13.1669
YDL215C	YJL047C	13.1669
YDR480W	YPL049C	13.1617
YHR069C	YPR190C	13.1516
YLL011W	YLR186W	13.1486
YGL253W	YML062C	13.1456
YDL014W	YDR060W	13.1446
YJL098W	YLR310C	13.1396
YLR019W	YOR043W	13.1354
YBL090W	YGR084C	13.1291
YBL090W	YGL129C	13.1291
YDR337W	YMR158W	13.1204
YAL011W	YJL081C	13.1187
YKR048C	YNL078W	13.1121
YAL029C	YMR109W	13.1102
YDR480W	YHR084W	13.1093
YPL151C	YPL213W	13.1092
YGR116W	YPR133C	13.1072
YHR066W	YPR143W	13.1044
YGR013W	YPR178W	13.1039
YJL066C	YMR203W	13.0997
YLR175W	YMR310C	13.0996
YML046W	YPR182W	13.0904
YDR045C	YPR110C	13.0874
YNL244C	YPR041W	13.0862
YBR211C	YIR010W	13.0846
YPR178W	YPR182W	13.0825
YIL153W	YNL201C	13.0716
YGR233C	YJL005W	13.069
YGL111W	YNL061W	13.0683
YLR075W	YML063W	13.0645
YLL036C	YMR288W	13.0572
YHR099W	YPR023C	13.0571
YHR056C	YMR033W	13.0508
YDR460W	YPR025C	13.0508
YDL150W	YOR340C	13.0445
YOR116C	YOR224C	13.044
YDL165W	YPR072W	13.0421
YMR257C	YPL237W	13.0385
YIR026C	YPL237W	13.0385
YDL014W	YMR128W	13.0372
YGR234W	YNL085W	13.0336
YLR417W	YPL002C	13.0261
YJR102C	YPL002C	13.0261
YGR200C	YMR093W	13.025
YFL018C	YNL071W	13.022
YNL189W	YOL142W	13.0211
YDR245W	YLR342W	13.0187
YDR363W-A	YDR394W	13.0179
YMR309C	YNL244C	13.0175
YGL111W	YLL034C	13.0123
YJL001W	YKL206C	13.0087
YDL030W	YDR416W	13.0074
YKL012W	YOR159C	13.0031
YDL014W	YOR063W	13.0031
YLR249W	YOL086C	13.0022
YER172C	YMR240C	12.9972
YCR020W-B	YKR008W	12.9882
YDL060W	YKL143W	12.9867
YDR436W	YOR054C	12.9846
YLR222C	YPR137W	12.9831
YBR247C	YNL207W	12.9827
YKR026C	YLR215C	12.9819
YFL039C	YPR023C	12.9798
YBL036C	YNL247W	12.978
YGL128C	YPL151C	12.9749
YGR200C	YPL126W	12.973
YEL036C	YJR075W	12.9729
YDL175C	YLR430W	12.9728
YER082C	YJR002W	12.9714
YJR002W	YNL075W	12.9669
YGL151W	YOL051W	12.9642
YDL098C	YDR378C	12.962
YJL081C	YLR385C	12.9525
YDL098C	YLR275W	12.9513
YEL015W	YJL124C	12.9453
YGR063C	YOL005C	12.9439
YMR290C	YOL077C	12.9438
YCL024W	YOL070C	12.9381
YLR002C	YOR272W	12.9363
YER006W	YPR016C	12.935
YBR088C	YKL113C	12.9329
YKL081W	YPL048W	12.9271
YGL242C	YMR109W	12.9254
YOR063W	YPL126W	12.9246
YDR260C	YHR166C	12.9205
YGR075C	YKL173W	12.9179
YOL001W	YPL219W	12.9164
YGR074W	YLR298C	12.9163
YAL011W	YLR385C	12.9135
YBR175W	YDR469W	12.9105
YDR334W	YLR399C	12.9091
YDL105W	YEL019C	12.9058
YGR162W	YJL138C	12.8995
YJL203W	YKL173W	12.8952
YOR151C	YPL129W	12.8916
YER071C	YKL007W	12.8876
YDR337W	YNR037C	12.8874
YBL038W	YNL284C	12.8874
YDR211W	YLR215C	12.8869
YFL002C	YKL009W	12.8798
YDL084W	YNL004W	12.8731
YHR052W	YLR002C	12.863
YFL008W	YMR001C	12.8602
YDR101C	YNR053C	12.8578
YDR188W	YJL111W	12.8524
YDR324C	YER082C	12.8483
YAL035W	YOR361C	12.845
YDR449C	YML093W	12.8431
YKL167C	YKR006C	12.8321
YBR109C	YBR130C	12.8307
YAL002W	YDR080W	12.8191
YDR449C	YLR186W	12.819
YCR071C	YPL173W	12.8175
YDL045W-A	YGR165W	12.8161
YDR363W-A	YHR200W	12.8154
YGL079W	YNL086W	12.8138
YEL005C	YNL086W	12.8138
YEL005C	YGL079W	12.8138
YCR060W	YHR034C	12.8075
YGR195W	YPR190C	12.7931
YNL236W	YOL051W	12.7918
YLR197W	YPL217C	12.7883
YBR258C	YHR119W	12.7848
YBR247C	YLR186W	12.7769
YIR025W	YKL022C	12.7732
YCR072C	YHR197W	12.7657
YML114C	YPL129W	12.7618
YDL209C	YER172C	12.7609
YDL132W	YOL133W	12.7557
YER172C	YNL147W	12.7503
YER125W	YMR275C	12.7489
YGL120C	YLR117C	12.7479
YJL041W	YMR047C	12.7475
YER107C	YJL041W	12.7475
YDR429C	YNL244C	12.7446
YDR141C	YIL076W	12.7331
YGR103W	YOR206W	12.7318
YDR235W	YPL178W	12.7306
YFR037C	YHR056C	12.73
YEL051W	YKL080W	12.7282
YER112W	YNL118C	12.7259
YER102W	YJR145C	12.7237
YDR167W	YMR223W	12.7235
YHR052W	YLL008W	12.7186
YBR175W	YKL018W	12.7184
YHR088W	YLR002C	12.7136
YGR074W	YPR057W	12.713
YBR127C	YOR151C	12.7026
YGL106W	YOR035C	12.701
YLR028C	YNR034W	12.6971
YHR090C	YNL136W	12.6969
YMR075W	YMR263W	12.6929
YBR061C	YPL183C	12.6916
YFL038C	YGL210W	12.6894
YGR161C	YML092C	12.6869
YDR237W	YMR024W	12.685
YJR002W	YPL217C	12.6806
YLR147C	YMR125W	12.6801
YJR053W	YML064C	12.6747
YHR005C	YOR212W	12.6732
YCR030C	YDR225W	12.6724
YMR288W	YNL286W	12.6692
YEL018W	YHR099W	12.6679
YHR197W	YNL002C	12.6667
YDR235W	YGR074W	12.6606
YDL043C	YPL213W	12.6537
YDL006W	YJL128C	12.6452
YDL006W	YDR162C	12.6451
YHR148W	YJR002W	12.645
YGL213C	YKL023W	12.6387
YGR156W	YMR061W	12.631
YGL111W	YLR002C	12.6218
YGL025C	YNL236W	12.6148
YER012W	YLR263W	12.6112
YAL053W	YER012W	12.6112
YGL129C	YGR215W	12.6082
YLL022C	YNL261W	12.6
YBL003C	YDR224C	12.598
YER133W	YFR003C	12.5975
YDR462W	YNL005C	12.5876
YCR046C	YDR462W	12.584
YGR063C	YPR187W	12.5812
YDR502C	YLR180W	12.5714
YBR088C	YOR039W	12.5629
YDR324C	YMR128W	12.5541
YGR103W	YOR063W	12.5503
YFR050C	YLR263W	12.543
YAL053W	YFR050C	12.543
YMR288W	YOR319W	12.5373
YBR154C	YGR063C	12.5277
YGR209C	YHR199C	12.5235
YCL011C	YNL139C	12.5218
YLR259C	YNL085W	12.5161
YGR135W	YGR161C	12.5159
YDR383C	YPL018W	12.5119
YHL030W	YMR314W	12.5066
YGR161C	YOL038W	12.506
YBL007C	YDR388W	12.4992
YGR254W	YLR044C	12.498
YBL041W	YGR161C	12.4961
YNL002C	YOR272W	12.495
YER006W	YMR290C	12.4945
YKL129C	YOR035C	12.4914
YDL148C	YMR128W	12.4909
YDL031W	YOR206W	12.4899
YDR462W	YJL063C	12.489
YHL027W	YNL312W	12.4867
YGR103W	YLR002C	12.4854
YDR363W-A	YOR117W	12.4828
YDR363W-A	YGL048C	12.4828
YDL131W	YNL178W	12.4817
YDL040C	YJR145C	12.4723
YER177W	YLR028C	12.4697
YDR190C	YHR034C	12.4664
YPL126W	YPR144C	12.4656
YCR020W-B	YCR052W	12.4592
YGR090W	YJR145C	12.4538
YDR382W	YOL127W	12.4533
YGR202C	YLR347C	12.4503
YBR065C	YPR101W	12.4503
YGL127C	YPR168W	12.4498
YDR443C	YGL025C	12.4448
YER082C	YGR090W	12.4396
YDR288W	YOL034W	12.433
YDL160C	YDR378C	12.4304
YBR154C	YPL129W	12.4257
YGR252W	YML015C	12.4254
YEL055C	YPL126W	12.4177
YEL003W	YJL179W	12.412
YDR045C	YNR003C	12.4116
YDL209C	YGL128C	12.411
YBL026W	YER112W	12.4048
YDR176W	YPL011C	12.4044
YDR427W	YOR108W	12.4
YLR384C	YMR312W	12.3986
YLR425W	YPL066W	12.398
YBR143C	YGL019W	12.3973
YER006W	YLL008W	12.3925
YDR381W	YML062C	12.3884
YLR039C	YLR262C	12.3855
YHR148W	YPR137W	12.3818
YBR001C	YDR001C	12.3785
YBR264C	YFL038C	12.3749
YGL006W	YHR104W	12.3732
YDR313C	YLR083C	12.3732
YDR051C	YLR024C	12.3732
YDL069C	YKL164C	12.3732
YDL054C	YOR315W	12.3732
YDL044C	YLR139C	12.3732
YBR281C	YIL173W	12.3732
YGL233W	YJL085W	12.3724
YGR245C	YKR048C	12.3694
YCR081W	YGL025C	12.3544
YCR066W	YHR137W	12.3509
YBR127C	YML085C	12.3504
YJL069C	YJL109C	12.3492
YGR040W	YPL049C	12.3449
YKL009W	YNL002C	12.342
YDR473C	YER112W	12.3418
YMR227C	YPL254W	12.3408
YBR221C	YGR135W	12.3391
YMR109W	YOR326W	12.3312
YJR145C	YMR290C	12.3289
YLR383W	YLR435W	12.3285
YJL031C	YPR176C	12.3285
YER140W	YOR154W	12.3285
YBR034C	YML017W	12.3285
YDR517W	YER012W	12.3269
YBL041W	YBR221C	12.3194
YGR091W	YJL203W	12.3183
YLR117C	YPL213W	12.3119
YKL193C	YML016C	12.309
YBR173C	YLR199C	12.3067
YBL035C	YJL047C	12.3012
YAL011W	YNL107W	12.2963
YLR409C	YPL126W	12.2906
YLR129W	YMR093W	12.2891
YDR496C	YMR049C	12.2861
YDR462W	YML025C	12.2822
YDL014W	YPR137W	12.2819
YBR065C	YDL209C	12.2747
YER012W	YJR051W	12.2725
YDL185W	YOR332W	12.2639
YDR517W	YFR050C	12.2588
YMR273C	YOR014W	12.2583
YDL014W	YGR090W	12.2564
YKL088W	YML016C	12.2556
YGL111W	YGR103W	12.2545
YDR060W	YNL061W	12.2518
YNL147W	YPR178W	12.2501
YGR063C	YGR186W	12.2485
YLR418C	YOL006C	12.247
YDR237W	YKR085C	12.2418
YMR093W	YPR137W	12.2364
YPL126W	YPL217C	12.2319
YDR116C	YLR312W-A	12.2319
YJL047C	YOL063C	12.2312
YER133W	YPL137C	12.2302
YBL004W	YJL109C	12.2287
YER006W	YFL002C	12.2255
YBR103W	YLR347C	12.2251
YBR127C	YDR171W	12.2182
YJR082C	YOR244W	12.2177
YBL038W	YDR237W	12.2136
YBL026W	YGL173C	12.2125
YGL240W	YNL172W	12.2124
YDR060W	YOL120C	12.2109
YDR118W	YIR025W	12.2072
YDR363W-A	YHR027C	12.2063
YLR055C	YMR005W	12.2059
YFR050C	YJR051W	12.2044
YFL039C	YGL106W	12.2039
YHR148W	YPL126W	12.1966
YKL012W	YPL178W	12.1956
YDL140C	YPL129W	12.1933
YHR088W	YPL211W	12.1915
YMR049C	YPR016C	12.1868
YHR034C	YKL013C	12.1855
YIR010W	YMR117C	12.1854
YDR496C	YLL008W	12.1767
YDL111C	YPR190C	12.1747
YBR278W	YPR175W	12.1712
YBR127C	YDR202C	12.1704
YDL155W	YMR199W	12.1702
YDL014W	YGL120C	12.1662
YDR091C	YMR146C	12.1649
YIL075C	YIL107C	12.1638
YLL008W	YNL061W	12.1605
YBR231C	YLR385C	12.1602
YKR006C	YKR085C	12.1559
YKL167C	YKR085C	12.1559
YJL081C	YPL129W	12.1529
YJL109C	YNL132W	12.15
YDL150W	YNL248C	12.1427
YBL026W	YLR275W	12.138
YDL045W-A	YKL155C	12.1337
YGR016W	YHR009C	12.128
YHR074W	YLR216C	12.1272
YBL046W	YDR293C	12.1245
YLR249W	YPL048W	12.1213
YLR044C	YMR186W	12.1213
YDL077C	YDR080W	12.1201
YDL051W	YKL122C	12.1118
YJL053W	YOR069W	12.1112
YLR288C	YPL029W	12.1096
YFR036W	YHR166C	12.0989
YDR363W-A	YIL075C	12.0989
YGR086C	YMR086W	12.0968
YNL270C	YOL062C	12.0962
YDL018C	YHR110W	12.0962
YBL004W	YMR128W	12.0919
YER126C	YNR053C	12.0885
YFR009W	YGL195W	12.0869
YMR093W	YMR128W	12.0857
YJL063C	YLR312W-A	12.0764
YDL030W	YPR082C	12.0762
YDR432W	YGL049C	12.0748
YDR296W	YMR024W	12.0726
YKR025W	YOR210W	12.0692
YJR050W	YLR117C	12.0659
YER104W	YNL312W	12.0588
YJR007W	YLR192C	12.0559
YDL065C	YER112W	12.0559
YKL206C	YPR103W	12.055
YDR279W	YLR154C	12.0451
YBR253W	YPL042C	12.0419
YLR216C	YOR027W	12.0402
YJR145C	YPL198W	12.0355
YBR055C	YMR288W	12.0345
YAL032C	YDR364C	12.0343
YDL030W	YPL151C	12.0327
YDR388W	YHR114W	12.0272
YDR405W	YMR024W	12.0236
YDR490C	YPL004C	12.0227
YBR055C	YKL012W	12.016
YNL061W	YPR016C	12.0135
YDR383C	YLR381W	12.0097
YDR045C	YPR187W	12.0076
YFL002C	YPL211W	12.0049
YKR006C	YNL252C	11.9985
YKL167C	YNL252C	11.9985
YOR207C	YOR210W	11.9967
YDR245W	YJR075W	11.9941
YDR324C	YPL217C	11.986
YJL053W	YPL070W	11.9785
YKL193C	YPL137C	11.9778
YCR046C	YMR024W	11.9761
YCR071C	YNL252C	11.9713
YKL022C	YOR249C	11.9702
YBR107C	YLR381W	11.9685
YBR107C	YDR318W	11.9685
YKL009W	YPL093W	11.9641
YOR206W	YOR272W	11.9635
YJL203W	YLR117C	11.9595
YGR090W	YMR290C	11.958
YDR324C	YHR148W	11.9508
YMR314W	YPR108W	11.9466
YCR077C	YEL015W	11.9429
YJL173C	YML032C	11.9386
YDR324C	YEL055C	11.9358
YDR318W	YGR006W	11.9338
YPL213W	YPR057W	11.9323
YDR190C	YPL129W	11.9309
YFL039C	YKL007W	11.9272
YER006W	YKL009W	11.927
YJR002W	YLR186W	11.9213
YBR170C	YDR049W	11.9206
YHL030W	YLR421C	11.9204
YDR167W	YHL047C	11.9203
YCL054W	YDL031W	11.9199
YKR029C	YLR347C	11.9151
YGR123C	YOR014W	11.9141
YIL126W	YPL082C	11.9127
YOL115W	YPL146C	11.9122
YLR312W-A	YML025C	11.9088
YDR324C	YLR197W	11.908
YGR104C	YPR168W	11.9038
YGR090W	YNL186W	11.8993
YDR101C	YER126C	11.8895
YGL174W	YIR005W	11.887
YCR057C	YGR210C	11.8866
YER050C	YOR158W	11.8819
YER050C	YJR101W	11.8797
YIL076W	YPL031C	11.8763
YDR449C	YER082C	11.8761
YBL041W	YER021W	11.8736
YCR014C	YDR108W	11.8731
YFR040W	YKR028W	11.8725
YBL026W	YER146W	11.8705
YBR122C	YDR462W	11.869
YFL034W	YHL019C	11.8661
YBR196C	YOL086C	11.8658
YDR416W	YPL213W	11.8631
YCR020W-B	YGR275W	11.8591
YEL019C	YLR383W	11.8584
YDL225W	YPL167C	11.855
YER050C	YGR215W	11.8548
YER082C	YLR186W	11.8533
YDR052C	YFR052W	11.8459
YBR031W	YNL132W	11.844
YCR073C	YNR031C	11.8436
YGL242C	YNL075W	11.8397
YGL242C	YKL035W	11.8397
YOL148C	YPL047W	11.837
YDL185W	YKL080W	11.8345
YKR048C	YOL070C	11.8319
YER006W	YLR002C	11.8249
YBR052C	YDR032C	11.8248
YDL188C	YML109W	11.8225
YDR449C	YPR144C	11.8217
YDR452W	YKR062W	11.8168
YBR122C	YNL252C	11.8123
YJL001W	YLR263W	11.8074
YAL053W	YJL001W	11.8074
YAL032C	YCR063W	11.7967
YER104W	YJL173C	11.7924
YKL173W	YKL188C	11.7923
YBL097W	YLR272C	11.791
YAL034W-A	YMR117C	11.791
YML117W	YPL190C	11.7903
YLR439W	YNL284C	11.7852
YLR197W	YLR222C	11.7835
YDR449C	YKR060W	11.7795
YCR042C	YPL129W	11.779
YIR025W	YNL172W	11.7774
YDL045W-A	YDR337W	11.7709
YFL033C	YLR033W	11.7706
YGL087C	YMR140W	11.7677
YDR092W	YMR140W	11.7677
YGR020C	YKL119C	11.767
YGR090W	YLR222C	11.7641
YBR031W	YMR290C	11.7635
YDL132W	YIL071C	11.7621
YHR115C	YNL311C	11.7606
YGL156W	YKL103C	11.7536
YDL030W	YDL098C	11.7396
YHR086W	YPR182W	11.7377
YHR088W	YNL002C	11.7361
YJR022W	YPR182W	11.7293
YDL030W	YOR308C	11.7271
YGL078C	YLR357W	11.7211
YLR335W	YMR125W	11.715
YFL039C	YML041C	11.7053
YDL045W-A	YJR113C	11.7043
YCR088W	YNL094W	11.7008
YGR040W	YHR084W	11.7006
YDR280W	YNR024W	11.6996
YLR249W	YMR186W	11.6976
YGL194C	YLR347C	11.6963
YBL004W	YNL132W	11.6919
YCR033W	YLR347C	11.6913
YGR158C	YNL189W	11.6827
YMR012W	YNL234W	11.68
YER153C	YNL274C	11.68
YLR085C	YLR385C	11.655
YDL148C	YNL132W	11.6493
YER082C	YLL011W	11.6486
YBR146W	YER050C	11.648
YNR003C	YPR010C	11.6459
YBR233W-A	YDR016C	11.6447
YGL115W	YIL095W	11.6434
YKL126W	YMR104C	11.6427
YOR116C	YOR210W	11.641
YMR229C	YOR206W	11.6396
YGR232W	YLR421C	11.6377
YAL036C	YOR091W	11.6359
YEL058W	YNL218W	11.6354
YBR055C	YJL203W	11.6349
YER021W	YPR103W	11.6322
YGR103W	YHR197W	11.6316
YCR066W	YGL087C	11.6286
YDR002W	YLR293C	11.6268
YCR077C	YLR438C-A	11.6247
YAL041W	YER114C	11.6221
YDL087C	YPL178W	11.6206
YGR054W	YJR145C	11.6173
YGR090W	YMR093W	11.6123
YHR165C	YMR213W	11.6112
YKL143W	YNL207W	11.6061
YLL011W	YPR144C	11.6045
YDR448W	YPL011C	11.6037
YMR049C	YOR312C	11.6034
YLR071C	YPR168W	11.6022
YMR290C	YPL211W	11.6007
YML117W	YNL251C	11.5982
YMR158W	YMR188C	11.5954
YLR385C	YNL107W	11.5918
YMR093W	YPL217C	11.5905
YAL038W	YJR121W	11.5902
YIR025W	YLR127C	11.5835
YHR052W	YLR449W	11.5814
YDR296W	YKR006C	11.5813
YDR296W	YKL167C	11.5813
YDR116C	YKR006C	11.5813
YDR116C	YKL167C	11.5813
YAL032C	YHR165C	11.5727
YBR102C	YJL085W	11.5702
YML007W	YNR010W	11.5666
YHR197W	YNR053C	11.5643
YHR148W	YLR186W	11.5622
YDR449C	YHR148W	11.5556
YBL004W	YPL217C	11.5556
YCR071C	YDR296W	11.5542
YKR085C	YNL005C	11.5536
YDR062W	YMR296C	11.5535
YLR002C	YOL077C	11.5531
YDR385W	YKL152C	11.5522
YDR404C	YGR063C	11.5502
YAL011W	YDR334W	11.5485
YJR076C	YPL167C	11.5436
YLR449W	YMR049C	11.542
YMR263W	YPR023C	11.5364
YJL050W	YNL251C	11.5364
YBL038W	YNL005C	11.5255
YDR517W	YJL001W	11.5244
YDR060W	YHR052W	11.5202
YHR064C	YLR249W	11.5198
YGR095C	YNL189W	11.5197
YOL010W	YPR112C	11.5139
YCL040W	YLR197W	11.5137
YDL043C	YLR147C	11.5098
YGL111W	YHR052W	11.5089
YNL189W	YOR098C	11.5086
YLR052W	YNL059C	11.5083
YDR025W	YNR054C	11.5075
YBL038W	YOR150W	11.5055
YCR046C	YKR006C	11.5031
YCR046C	YKL167C	11.5031
YCR066W	YDL074C	11.5029
YML123C	YPL249C	11.5026
YDR060W	YPL198W	11.4972
YHR034C	YNR035C	11.4961
YHR107C	YJR092W	11.4949
YDR175C	YHR059W	11.4929
YMR128W	YPL217C	11.4833
YDL045W-A	YIL093C	11.4825
YER012W	YPR108W	11.4819
YDL108W	YDR311W	11.4794
YCR046C	YCR071C	11.476
YER171W	YPR056W	11.4721
YJL001W	YJR051W	11.4702
YDL209C	YLR424W	11.4696
YKL088W	YOR061W	11.4653
YJL154C	YPL070W	11.4619
YGR005C	YGR063C	11.4602
YBL038W	YKR085C	11.4595
YFL034W	YLR170C	11.4588
YKL080W	YPR036W	11.4572
YFL002C	YHR088W	11.4538
YJL128C	YNR031C	11.4533
YHR059W	YIL093C	11.4505
YDR296W	YMR193W	11.4477
YER133W	YOR179C	11.4473
YCR046C	YDR237W	11.4392
YDR496C	YLR449W	11.4348
YGR090W	YIL035C	11.4328
YKL173W	YNL147W	11.4323
YBL002W	YBL003C	11.4304
YJL063C	YKR006C	11.4265
YJL063C	YKL167C	11.4265
YGL043W	YOL005C	11.4256
YHR107C	YPL167C	11.4238
YDL132W	YDR179C	11.4196
YLR196W	YOL077C	11.4177
YDR443C	YMR112C	11.4149
YBL017C	YMR241W	11.413
YBL050W	YKL196C	11.4115
YGR020C	YKL080W	11.4108
YBR017C	YBR205W	11.4104
YAL036C	YJR145C	11.409
YGR202C	YOR098C	11.4055
YMR188C	YNR037C	11.4035
YBR264C	YER136W	11.3994
YBR122C	YDR296W	11.3956
YBR122C	YDR116C	11.3956
YDL209C	YML049C	11.3955
YJR022W	YOR308C	11.3947
YHR148W	YLL011W	11.3889
YBL090W	YMR158W	11.3869
YGR215W	YPL013C	11.3866
YOR158W	YPL013C	11.3842
YGR090W	YJR002W	11.3831
YLR382C	YMR240C	11.382
YDL045W-A	YDR175C	11.3809
YDL075W	YGL103W	11.3723
YCR046C	YMR193W	11.3696
YBR154C	YDR045C	11.3679
YER094C	YNR003C	11.3655
YDL150W	YOR210W	11.3624
YMR308C	YNL030W	11.3621
YBR114W	YPL022W	11.362
YGR202C	YNL189W	11.3603
YGR285C	YJL080C	11.3509
YDR237W	YJL063C	11.3452
YML046W	YPL213W	11.345
YGL120C	YJL050W	11.3423
YHR166C	YIR025W	11.336
YER146W	YOR308C	11.3359
YBR289W	YPR034W	11.3328
YAR007C	YHL027W	11.3327
YJL011C	YOR224C	11.3313
YNL084C	YNL243W	11.3309
YDL047W	YGL197W	11.3304
YIL107C	YKL145W	11.3287
YGL092W	YPL169C	11.328
YHR052W	YPL093W	11.3276
YCR081W	YMR112C	11.3254
YKL009W	YPR016C	11.3207
YBR122C	YCR046C	11.3175
YBR130C	YMR109W	11.3139
YJR145C	YOR253W	11.3119
YCR002C	YPL167C	11.3107
YDR097C	YIR002C	11.3075
YBL038W	YNL252C	11.3027
YCL040W	YPR137W	11.2974
YDR303C	YPL082C	11.2971
YOR089C	YOR370C	11.2961
YEL015W	YML091C	11.2957
YJL063C	YMR193W	11.293
YDL148C	YPL126W	11.2907
YCR033W	YMR155W	11.2882
YCR033W	YML109W	11.2882
YCR033W	YGR296W	11.2882
YCR033W	YEL064C	11.2882
YDR092W	YHR137W	11.2817
YKL193C	YLR028C	11.2809
YBL058W	YDR049W	11.2777
YJL069C	YMR093W	11.2743
YDR052C	YGR232W	11.2729
YDR347W	YER050C	11.2712
YKL059C	YMR061W	11.2702
YBL002W	YMR072W	11.2693
YFL038C	YML001W	11.2685
YGL173C	YKL188C	11.2671
YKR060W	YPL126W	11.2643
YMR146C	YPL105C	11.2609
YKR006C	YML025C	11.2595
YFL039C	YLR399C	11.2488
YCL031C	YGL019W	11.247
YLR129W	YPL126W	11.2419
YCR063W	YLR117C	11.2375
YER164W	YOR061W	11.2359
YBR173C	YKL206C	11.2322
YER050C	YKL155C	11.232
YOR213C	YOR229W	11.2299
YOR061W	YOR123C	11.2293
YHL027W	YML032C	11.229
YKL172W	YPL093W	11.2226
YAR042W	YDL019C	11.2167
YGL043W	YGL128C	11.2117
YIL046W	YNL103W	11.2054
YJR002W	YLR222C	11.2048
YDR224C	YLR133W	11.1979
YDR087C	YFL002C	11.1966
YAL016W	YMR167W	11.1922
YJR076C	YJR092W	11.1917
YGL245W	YGR264C	11.1855
YIL061C	YKL173W	11.1786
YDR404C	YML010W	11.1737
YER008C	YJL085W	11.1722
YDR166C	YJL085W	11.1722
YDR473C	YML049C	11.17
YBL008W	YNL085W	11.1693
YDL105W	YOL034W	11.1624
YBR251W	YMR158W	11.1606
YML062C	YNL004W	11.1598
YGR165W	YIL070C	11.1581
YER172C	YGR278W	11.1571
YER050C	YPL118W	11.1531
YGR215W	YMR158W	11.1515
YLR429W	YMR109W	11.1471
YGL120C	YLR197W	11.1466
YGR231C	YLR342W	11.1354
YHL030W	YKL145W	11.1312
YKL172W	YLL008W	11.13
YLL011W	YMR093W	11.1294
YDR449C	YLL011W	11.1294
YGL019W	YOR123C	11.128
YFL002C	YLL008W	11.1272
YER050C	YGR165W	11.1272
YFL017W-A	YHR165C	11.1268
YDL143W	YIL142W	11.1266
YJR033C	YOR332W	11.1247
YER050C	YNL137C	11.1152
YJL109C	YJR002W	11.1088
YBR267W	YDR101C	11.1071
YDR129C	YFL039C	11.1053
YNL178W	YPL239W	11.1016
YER112W	YPR082C	11.101
YHR013C	YLL045C	11.1008
YHR179W	YPL171C	11.0991
YBL058W	YMR067C	11.0963
YGL070C	YGR063C	11.0943
YAL036C	YGR054W	11.0834
YLR215C	YMR257C	11.0829
YIR026C	YLR215C	11.0829
YDR485C	YML041C	11.0815
YKR001C	YMR091C	11.0795
YGL106W	YLR429W	11.068
YML063W	YPL090C	11.0595
YLR147C	YPR057W	11.0589
YKL215C	YLL026W	11.0578
YBR060C	YLL022C	11.0563
YDL065C	YNL118C	11.0544
YAL003W	YLR249W	11.0487
YDR449C	YNL132W	11.0477
YIR001C	YNL016W	11.0475
YGL049C	YML017W	11.0448
YBR009C	YGL252C	11.0447
YGR103W	YKR081C	11.0396
YPL129W	YPL235W	11.0394
YGR103W	YHR052W	11.0356
YLR385C	YLR399C	11.0309
YDR462W	YMR024W	11.0307
YJR092W	YLR314C	11.0297
YDR378C	YLR147C	11.0288
YDR314C	YOR261C	11.0273
YNL112W	YOR063W	11.0245
YGR091W	YPL213W	11.0225
YER172C	YJL124C	11.022
YHR042W	YKR029C	11.0205
YLR215C	YMR309C	11.0199
YBL084C	YKL022C	11.0197
YML049C	YPL151C	11.0138
YBR087W	YMR078C	11.0129
YAL034C	YER110C	11.0126
YGL078C	YLR033W	11.01
YHR165C	YNL147W	11.0081
YDR477W	YEL023C	11.0081
YER112W	YMR268C	11.0079
YGR254W	YHR174W	11.0067
YER125W	YHR131C	11.0067
YDR145W	YMR223W	11.0051
YAR014C	YER133W	10.9983
YKL057C	YPL169C	10.992
YMR128W	YPL126W	10.9892
YDR087C	YPL211W	10.9885
YJL144W	YMR012W	10.9869
YIL078W	YKR018C	10.9869
YER040W	YNL229C	10.9869
YDR532C	YGL093W	10.9869
YDR189W	YLR026C	10.9869
YDL008W	YER047C	10.9869
YBR045C	YGL057C	10.9869
YAL060W	YAL061W	10.9869
YGR245C	YNL182C	10.9831
YCR063W	YPR101W	10.9825
YDR388W	YLR429W	10.975
YBR009C	YNL031C	10.9736
YDL097C	YDR363W-A	10.9715
YER112W	YER172C	10.9707
YDR427W	YMR314W	10.9688
YBL084C	YGL240W	10.9647
YMR186W	YOR027W	10.9609
YCR057C	YDL148C	10.9594
YJL203W	YPR178W	10.9551
YAR014C	YPL137C	10.9542
YLR275W	YMR125W	10.9541
YDL134C	YGR161C	10.9534
YLL036C	YPL213W	10.953
YBR251W	YER050C	10.9525
YNL132W	YPR144C	10.9508
YDL031W	YPR016C	10.9502
YOR251C	YOR346W	10.9422
YJR104C	YOL081W	10.9422
YGR188C	YOR026W	10.9422
YGR093W	YKL149C	10.9422
YGR019W	YHR124W	10.9422
YFL059W	YMR096W	10.9422
YEL041W	YJR049C	10.9422
YDR231C	YNL192W	10.9422
YDR159W	YOL072W	10.9422
YHR027C	YIL107C	10.9412
YJR082C	YPR023C	10.937
YBR233W-A	YKR083C	10.9356
YDR028C	YDR422C	10.9299
YHR152W	YMR308C	10.9228
YJR145C	YNL132W	10.9178
YIL126W	YOR304W	10.9144
YLR074C	YNR053C	10.9134
YLR275W	YPR057W	10.9122
YDR296W	YKR085C	10.9103
YDR116C	YKR085C	10.9103
YDL185W	YIL128W	10.9057
YGR278W	YHR165C	10.9049
YKR060W	YLR409C	10.9037
YBR081C	YGR274C	10.9037
YMR199W	YPL256C	10.9024
YER172C	YGL120C	10.9024
YBR247C	YOL123W	10.9014
YNL207W	YPL204W	10.9
YBR079C	YER102W	10.8965
YCR077C	YGL173C	10.8921
YBL038W	YDR296W	10.8875
YBL038W	YDR116C	10.8875
YIL142W	YOR281C	10.8848
YDL030W	YGR074W	10.8847
YDL030W	YML046W	10.8782
YBR143C	YOR061W	10.8757
YER029C	YMR125W	10.8741
YDR227W	YFL007W	10.8716
YBL090W	YNR037C	10.871
YGR002C	YLR347C	10.8695
YHR066W	YIL076W	10.8689
YDR337W	YER050C	10.8684
YKL167C	YLR439W	10.8635
YER082C	YLR197W	10.8614
YLR102C	YOR249C	10.8571
YDL005C	YMR112C	10.8564
YER172C	YKL188C	10.855
YOL021C	YPR190C	10.8469
YLR186W	YMR093W	10.846
YLR055C	YPL011C	10.8437
YER112W	YOR308C	10.8434
YJL124C	YPR082C	10.8414
YER082C	YHR196W	10.8392
YCR046C	YKR085C	10.8324
YDR289C	YOR048C	10.8311
YJR101W	YPL013C	10.8304
YDR225W	YGL207W	10.8289
YER146W	YKL188C	10.8285
YFL033C	YMR033W	10.8238
YDR427W	YNL004W	10.8191
YCR072C	YDR101C	10.8187
YGL120C	YGR017W	10.8156
YER165W	YPL178W	10.8155
YGR103W	YGR245C	10.8123
YBL038W	YCR046C	10.8097
YER050C	YJR113C	10.8053
YBL090W	YBR251W	10.8053
YDR028C	YIL095W	10.805
YDL175C	YPL146C	10.7984
YIL126W	YKR001C	10.793
YIL076W	YPR143W	10.7888
YJR002W	YPR144C	10.788
YGR054W	YNL132W	10.7813
YDR378C	YNL147W	10.7808
YBL004W	YPR137W	10.7764
YIL070C	YNL137C	10.7729
YMR303C	YOL086C	10.7722
YNL151C	YOR210W	10.7707
YNL106C	YPR171W	10.7658
YNL061W	YNL110C	10.7658
YDL031W	YDR087C	10.7649
YIL035C	YOL006C	10.7643
YML106W	YMR271C	10.7638
YDR473C	YOR159C	10.7625
YBL003C	YBR245C	10.762
YGR091W	YMR240C	10.7593
YDR496C	YGR103W	10.7579
YER172C	YPR101W	10.7571
YDR028C	YNR034W	10.751
YJL026W	YOL086C	10.7507
YDR240C	YKL173W	10.7506
YBL004W	YJR002W	10.7479
YDL148C	YLR186W	10.7476
YOR048C	YOR151C	10.747
YOL051W	YPL042C	10.7464
YCL059C	YDL014W	10.7395
YBR011C	YLR425W	10.7393
YBR011C	YDL203C	10.7393
YCR033W	YDL112W	10.7391
YDR394W	YMR314W	10.7384
YER012W	YGR161C	10.7367
YHR165C	YJL124C	10.7355
YBL038W	YJL063C	10.7335
YLR439W	YMR193W	10.7306
YKL188C	YPR082C	10.717
YLR407W	YOR061W	10.7141
YER050C	YHL004W	10.7118
YGR274C	YPL254W	10.7111
YIL076W	YPL088W	10.7108
YCR071C	YDR462W	10.708
YDL031W	YLL034C	10.7061
YBR065C	YLL036C	10.7032
YHR041C	YPR168W	10.6991
YOR014W	YPL152W	10.69
YAL016W	YOR386W	10.6897
YPL018W	YPR046W	10.687
YOR210W	YPR190C	10.6803
YBR122C	YLR439W	10.6787
YPL042C	YPR168W	10.675
YDR322W	YKR006C	10.6723
YDR322W	YKL167C	10.6723
YFR050C	YGR161C	10.6694
YBL003C	YMR072W	10.6635
YDR252W	YHR193C	10.6612
YDL148C	YMR093W	10.6606
YDL148C	YDR449C	10.6606
YDR252W	YJR011C	10.66
YDR252W	YGR134W	10.66
YGR238C	YHR158C	10.6592
YGL043W	YGL070C	10.6564
YDL084W	YDR381W	10.655
YER012W	YFL007W	10.6478
YJR002W	YNL132W	10.6417
YKR062W	YNL112W	10.6416
YDL098C	YLR147C	10.6362
YER006W	YGR245C	10.6345
YGR165W	YPL013C	10.6291
YDL031W	YOR272W	10.6289
YGR128C	YLR222C	10.6287
YDR448W	YNL236W	10.6285
YJL140W	YPL225W	10.6264
YGR165W	YMR158W	10.6262
YGL145W	YLR268W	10.623
YDR293C	YML010W	10.6182
YLR438C-A	YNL118C	10.6176
YKR059W	YNL201C	10.6175
YLR222C	YPL217C	10.6147
YLR147C	YLR298C	10.6123
YJL050W	YMR239C	10.6104
YHR098C	YPR181C	10.6079
YBL084C	YLR102C	10.6072
YGR200C	YHR196W	10.603
YBR146W	YPL013C	10.6021
YDL117W	YER177W	10.6019
YJL063C	YNL252C	10.6002
YGR128C	YJR002W	10.5975
YKL052C	YKR083C	10.5974
YDL060W	YPL012W	10.5963
YBR221C	YER012W	10.5951
YLL008W	YPL093W	10.5948
YDR378C	YMR268C	10.5929
YFL002C	YNL061W	10.5907
YDL043C	YOR319W	10.5867
YER050C	YIL093C	10.5859
YJL124C	YOR308C	10.5846
YMR074C	YNL256W	10.5814
YLR403W	YOR370C	10.5814
YHL039W	YKR071C	10.5814
YGL232W	YNL177C	10.5814
YDR501W	YKR078W	10.5814
YBL026W	YDL098C	10.5814
YDL099W	YDR517W	10.5799
YER006W	YER126C	10.5762
YNL061W	YOR272W	10.5745
YGR220C	YNL177C	10.5706
YHR165C	YKL188C	10.5694
YPL213W	YPR101W	10.5693
YBL038W	YML025C	10.5674
YGL252C	YNL206C	10.5659
YBR048W	YNR054C	10.5655
YBR055C	YIL061C	10.5595
YDR041W	YER050C	10.5587
YBL002W	YBR010W	10.5577
YAR007C	YER104W	10.5558
YGR090W	YLR129W	10.5545
YBR245C	YLR357W	10.5532
YDL156W	YKR048C	10.5462
YKL056C	YPL048W	10.5454
YML123C	YMR275C	10.5399
YGR145W	YMR290C	10.5382
YDR324C	YGR090W	10.5371
YER043C	YFL028C	10.5368
YPL126W	YPR137W	10.5317
YBL084C	YIR025W	10.5308
YDR289C	YLR347C	10.5292
YBR221C	YFR050C	10.5279
YBR143C	YOR039W	10.5277
YER172C	YJR022W	10.5235
YDL045W-A	YHR059W	10.5232
YHR197W	YNL110C	10.5227
YMR049C	YPL211W	10.5175
YGR075C	YOR308C	10.5172
YGR091W	YMR288W	10.5147
YBL093C	YMR112C	10.5145
YBR215W	YPL153C	10.5077
YDR485C	YNL107W	10.5069
YER006W	YHR052W	10.5032
YDR101C	YPR016C	10.5029
YER006W	YPL211W	10.5017
YAL011W	YML041C	10.5014
YGL111W	YPL211W	10.4996
YOL077C	YPL093W	10.4922
YDL014W	YJR002W	10.4886
YNL207W	YPL266W	10.4884
YDR175C	YER050C	10.4872
YDR129C	YLR429W	10.4865
YLR449W	YPL211W	10.4855
YGR162W	YMR125W	10.4854
YDL190C	YEL037C	10.4785
YFR040W	YHR096C	10.476
YER082C	YPR144C	10.4719
YPL049C	YPL115C	10.4699
YOL077C	YPL211W	10.462
YKL188C	YOR308C	10.4606
YML063W	YPR056W	10.4596
YGL066W	YML015C	10.4591
YJL099W	YOR299W	10.4589
YJL099W	YMR237W	10.4589
YJL099W	YLR330W	10.4589
YGL143C	YIL046W	10.4561
YDL164C	YGL143C	10.4561
YER126C	YPL093W	10.4507
YBR251W	YPL013C	10.4495
YBR233W-A	YGR113W	10.4494
YLL008W	YOR206W	10.4479
YER091C	YGL241W	10.4477
YBR160W	YPR120C	10.4426
YER082C	YNL132W	10.438
YFL002C	YPL093W	10.434
YFL024C	YLR399C	10.4284
YLR430W	YPL190C	10.4266
YIL142W	YJL111W	10.4263
YBR065C	YPL151C	10.4239
YAR007C	YCR092C	10.4209
YNL132W	YOR253W	10.4187
YLR033W	YPL082C	10.4098
YPL082C	YPR073C	10.4079
YOR386W	YPL104W	10.4079
YMR055C	YPL040C	10.4079
YJR110W	YLR071C	10.4079
YJR061W	YKL168C	10.4079
YJL165C	YOL103W	10.4079
YGR258C	YOL044W	10.4079
YDR335W	YOL051W	10.4079
YDR283C	YNL213C	10.4079
YCR091W	YNR047W	10.4079
YCL017C	YPL135W	10.4079
YJR101W	YNR037C	10.4074
YCR057C	YKR060W	10.4062
YDR224C	YIL126W	10.4038
YDL042C	YJL076W	10.4032
YDR058C	YKR048C	10.3991
YBR267W	YKR048C	10.3991
YBR245C	YIL126W	10.3949
YER127W	YMR290C	10.3945
YCR009C	YGL060W	10.3901
YNL172W	YOR249C	10.3853
YLR102C	YNL172W	10.3853
YMR314W	YOR259C	10.3849
YNL251C	YOL115W	10.3788
YBR055C	YGR013W	10.3761
YDR050C	YOL086C	10.3738
YKR081C	YPL093W	10.3706
YMR078C	YNL290W	10.3699
YNL002C	YPR016C	10.369
YGR054W	YGR090W	10.3686
YDL126C	YMR284W	10.3651
YGR103W	YMR290C	10.3634
YHR086W	YPL178W	10.3624
YDR353W	YKL085W	10.3603
YDL209C	YPL213W	10.3573
YDL156W	YNL312W	10.352
YDR324C	YOR063W	10.3479
YBR031W	YPL198W	10.3464
YDR098C	YLL029W	10.339
YDR324C	YPR137W	10.3372
YGR006W	YLR315W	10.3298
YGL106W	YPR188C	10.3265
YDL007W	YMR314W	10.3246
YDR416W	YLR424W	10.3236
YGR020C	YLR447C	10.317
YDR484W	YJL034W	10.3124
YDR027C	YJL034W	10.3124
YDR449C	YMR128W	10.3098
YLR002C	YNL110C	10.3073
YER082C	YGR128C	10.3021
YLR270W	YPL247C	10.3019
YKR001C	YPL247C	10.3019
YKR001C	YLR270W	10.3019
YAR014C	YKL193C	10.3003
YDR364C	YKL173W	10.2967
YDR429C	YOR204W	10.2963
YOL133W	YPR164W	10.295
YDL234C	YIL078W	10.2938
YNL201C	YOR133W	10.2933
YLL008W	YLR002C	10.2894
YGL127C	YOL135C	10.2891
YNL025C	YPL042C	10.2887
YBR247C	YPL266W	10.2869
YDL043C	YHR165C	10.2838
YKR001C	YLR033W	10.2834
YHR013C	YPL198W	10.2819
YDR179C	YDR328C	10.2815
YGR275W	YJL176C	10.277
YJL069C	YPR137W	10.2768
YDR473C	YNL147W	10.2759
YKL014C	YPL012W	10.2755
YLR196W	YOR206W	10.274
YDL132W	YMR025W	10.2716
YBR060C	YPL001W	10.2698
YER165W	YGL049C	10.2682
YNL132W	YPL217C	10.2679
YKL155C	YPL013C	10.2632
YLR102C	YLR127C	10.2631
YGL011C	YNR003C	10.2612
YDR098C	YGR262C	10.261
YGL242C	YPL061W	10.2541
YIL035C	YIL084C	10.2524
YLR129W	YOR145C	10.2515
YGL125W	YML082W	10.2491
YIL075C	YMR314W	10.2483
YMR311C	YOR227W	10.2477
YLR439W	YOR150W	10.2417
YCR057C	YLR186W	10.2413
YDR363W-A	YDR427W	10.241
YGR006W	YJR135C	10.2342
YJR002W	YLR409C	10.2333
YGR193C	YNL071W	10.2324
YEL018W	YJR082C	10.2299
YGR123C	YLR216C	10.2298
YDR227W	YJL076W	10.2295
YIR002C	YNL312W	10.2292
YDR097C	YNL312W	10.2292
YKL028W	YPR056W	10.2255
YLR197W	YLR409C	10.223
YIL070C	YPL001W	10.2211
YHR148W	YPR144C	10.2173
YNL110C	YOR272W	10.217
YBR282W	YMR193W	10.2123
YFL017W-A	YOR159C	10.2092
YDR404C	YPL129W	10.2074
YKR059W	YOL139C	10.2031
YDL140C	YGL043W	10.202
YHR052W	YOR206W	10.1991
YDR101C	YGR245C	10.1963
YLL050C	YNL138W	10.1953
YMR128W	YMR290C	10.1947
YDL031W	YOL077C	10.1919
YMR288W	YPR101W	10.1907
YDL209C	YHR165C	10.1895
YDL098C	YJL203W	10.1887
YBR231C	YER178W	10.1886
YDR296W	YJL063C	10.1874
YJR050W	YPR101W	10.1846
YNR037C	YPL118W	10.1807
YBR146W	YNR037C	10.1807
YBR247C	YDL148C	10.1774
YHR020W	YJR077C	10.1767
YBL004W	YPL126W	10.1764
YMR043W	YNL042W	10.176
YML031W	YPL177C	10.176
YJR067C	YNL260C	10.176
YJL107C	YOR265W	10.176
YGR248W	YOR018W	10.176
YBR249C	YKL056C	10.176
YHR052W	YNL061W	10.1755
YML049C	YPR082C	10.1737
YBR055C	YMR240C	10.173
YNL039W	YOR207C	10.1711
YLR197W	YNL132W	10.1706
YGR090W	YPL217C	10.1702
YML007W	YOL051W	10.1682
YKR081C	YOR206W	10.1661
YGL129C	YPL013C	10.1659
YDR485C	YFL039C	10.1658
YCR073W-A	YDR028C	10.1656
YJL081C	YPL254W	10.1632
YGR252W	YPL047W	10.1595
YER082C	YPR137W	10.1585
YDR448W	YGL151W	10.1584
YBR278W	YDR121W	10.1576
YBR055C	YJR022W	10.1576
YNL110C	YOL077C	10.1524
YBL090W	YDR036C	10.1484
YDR347W	YPL013C	10.1448
YGR233C	YIL050W	10.1404
YFR021W	YFR040W	10.1404
YLR196W	YOL041C	10.1397
YLR052W	YOR189W	10.136
YGR002C	YNL330C	10.134
YDL030W	YLR275W	10.132
YDR240C	YPL178W	10.1318
YGR206W	YPL065W	10.1313
YDR088C	YGR006W	10.1313
YBL080C	YMR293C	10.1313
YGR220C	YKR006C	10.1284
YGR220C	YKL167C	10.1284
YKL172W	YNL061W	10.1277
YDL225W	YJR092W	10.1277
YDR092W	YER125W	10.1266
YNL189W	YPR137W	10.1252
YJL176C	YPL129W	10.1251
YDL175C	YNL251C	10.1217
YPL217C	YPR112C	10.1216
YMR024W	YMR193W	10.1147
YDR462W	YOR150W	10.1139
YDL014W	YDR449C	10.1062
YHR064C	YJL080C	10.0999
YBL004W	YGR090W	10.0952
YCR063W	YML049C	10.0923
YDL076C	YPR023C	10.0866
YPL237W	YPR041W	10.0849
YGR103W	YKL172W	10.0823
YGR253C	YNR003C	10.0759
YGR128C	YPL217C	10.0759
YGR278W	YMR213W	10.0754
YKL088W	YKR072C	10.0706
YGR170W	YNL264C	10.0706
YJR113C	YPL013C	10.0696
YDR462W	YKR085C	10.0684
YGL078C	YMR033W	10.0661
YFL013C	YOR189W	10.0619
YDR045C	YOR224C	10.0566
YLR275W	YPL213W	10.0558
YDR322W	YOR150W	10.0516
YDR175C	YMR158W	10.0506
YGR128C	YHR148W	10.0483
YIL035C	YKL088W	10.0421
YOR159C	YPR178W	10.0417
YBR031W	YDR012W	10.0402
YDR328C	YOL133W	10.0397
YER006W	YHR088W	10.0396
YBR211C	YGR006W	10.0346
YGR005C	YPR110C	10.0278
YKL007W	YLR429W	10.0265
YDR449C	YPR137W	10.0243
YBR198C	YML114C	10.0083
YGL105W	YLL024C	10.0079
YIL035C	YLL022C	10.0077
YNL093W	YOR089C	10.0058
YER136W	YFL005W	10.0058
YGL078C	YMR091C	10.0007
YKL127W	YMR105C	10.0005
YAL035W	YER025W	10.0003
YDR064W	YGR192C	9.99999999999999e-05
YGL112C	YML114C	9.9978
YDR496C	YKL014C	9.9969
YJL080C	YLL026W	9.9927
YLR186W	YNL132W	9.9924
YGR075C	YGR091W	9.9923
YDR028C	YPL137C	9.9906
YHR039C-A	YOR332W	9.9902
YDR443C	YOL051W	9.9901
YGR252W	YMR005W	9.9896
YFL002C	YNL002C	9.9888
YLL004W	YLL022C	9.9887
YDL060W	YPL266W	9.9881
YHR084W	YPL115C	9.9868
YLR382C	YPL213W	9.9857
YHR052W	YKL172W	9.9823
YMR078C	YOL094C	9.9813
YDL209C	YMR288W	9.9798
YMR075W	YNL189W	9.9791
YHR115C	YNL116W	9.9786
YDL013W	YNR031C	9.9781
YBL002W	YBR009C	9.9774
YER082C	YLR409C	9.9771
YDL060W	YGR054W	9.9743
YLR074C	YPL093W	9.9727
YOL010W	YPL217C	9.9705
YLL022C	YPR162C	9.9685
YKL196C	YOR036W	9.9679
YHR058C	YML007W	9.9652
YLR222C	YNL132W	9.9648
YGR253C	YLR199C	9.9645
YDL209C	YGL120C	9.9643
YGR081C	YMR308C	9.9609
YGL127C	YHR041C	9.9587
YGR161C	YPL152W	9.9564
YAL029C	YBL017C	9.9529
YBR055C	YPR182W	9.9517
YHR165C	YMR125W	9.9507
YIL112W	YLR347C	9.9499
YER112W	YHR165C	9.9499
YLR357W	YPL082C	9.9451
YGR161C	YJL001W	9.9436
YKR001C	YMR304W	9.9406
YLL008W	YPL211W	9.9402
YFL010C	YOR124C	9.9398
YBR136W	YIR002C	9.9392
YBR136W	YDR097C	9.9392
YDR473C	YHR156C	9.9342
YBR253W	YGL127C	9.9329
YMR125W	YOR098C	9.9316
YGR090W	YMR128W	9.9302
YAL034C	YAR002W	9.929
YKL206C	YOL038W	9.9257
YGR255C	YPL139C	9.9225
YBL041W	YKL206C	9.9218
YBL072C	YMR146C	9.9211
YDR443C	YPR168W	9.9192
YCR081W	YOL051W	9.918
YBR245C	YDR303C	9.9161
YGL129C	YNR037C	9.9155
YDL188C	YPL152W	9.9152
YKL188C	YMR268C	9.9147
YDR227W	YNL189W	9.9099
YKL028W	YLR005W	9.9089
YDR311W	YKL028W	9.9089
YLR418C	YMR125W	9.9076
YGL120C	YKL173W	9.9021
YHR148W	YJL109C	9.9017
YDR049W	YOL139C	9.8975
YNR003C	YOR362C	9.8972
YAL032C	YDL209C	9.8909
YAL003W	YHR064C	9.8908
YDL014W	YOR310C	9.8903
YEL037C	YPL096W	9.8891
YMR260C	YNR050C	9.8883
YMR105C	YOR090C	9.8883
YDR486C	YLR181C	9.8883
YDR023W	YHL039W	9.8883
YDL241W	YKL157W	9.8883
YDL036C	YDL063C	9.8883
YBR268W	YDL202W	9.8883
YML046W	YPR178W	9.888
YDL030W	YLR382C	9.8865
YDR176W	YMR005W	9.8826
YEL056W	YHR118C	9.8787
YLL026W	YMR186W	9.8784
YGL207W	YLR133W	9.8731
YER092W	YPL129W	9.8726
YJL203W	YOR319W	9.8715
YDR060W	YOL077C	9.8691
YGR113W	YKR083C	9.8679
YBL004W	YDR324C	9.8654
YDL060W	YOR056C	9.8643
YGR090W	YJL109C	9.8606
YHR165C	YJR050W	9.856
YCR063W	YMR240C	9.8551
YGR006W	YGR179C	9.8532
YDR224C	YPL082C	9.8526
YHR148W	YHR196W	9.8486
YDR432W	YPL190C	9.8484
YCR081W	YPR168W	9.8472
YGL120C	YLL036C	9.8465
YDR346C	YPR118W	9.8436
YDR331W	YHR188C	9.8436
YJR053W	YMR153W	9.8368
YBL093C	YOL135C	9.8362
YER112W	YLR147C	9.8359
YML049C	YOR308C	9.8354
YMR093W	YPR144C	9.8303
YBR009C	YKR001C	9.8265
YGR123C	YPL240C	9.8262
YML007W	YOR174W	9.8232
YBR253W	YDL005C	9.8209
YGR091W	YKL188C	9.8207
YBR025C	YJR121W	9.8187
YDL002C	YKL013C	9.8179
YLR275W	YPL178W	9.8176
YDR308C	YPL042C	9.8159
YDR237W	YKR006C	9.8153
YDR237W	YKL167C	9.8153
YIL142W	YNL212W	9.8144
YER006W	YOL077C	9.8141
YKL012W	YKL173W	9.8107
YMR201C	YPR135W	9.8103
YER162C	YPR135W	9.8103
YDL014W	YGR145W	9.81
YDR507C	YOL070C	9.8091
YDR334W	YGR002C	9.8067
YIL144W	YKL173W	9.8065
YCR063W	YER029C	9.8033
YBR221C	YJL001W	9.8031
YGL049C	YMR125W	9.8013
YER112W	YLR275W	9.8009
YFR024C-A	YOR181W	9.8
YER177W	YGL252C	9.7964
YGR192C	YKL152C	9.7936
YER062C	YIL053W	9.7901
YCR071C	YDR237W	9.7886
YDR016C	YDR201W	9.7855
YMR290C	YPL043W	9.7852
YDR052C	YIL075C	9.7849
YDR517W	YIL109C	9.7826
YFL018C	YGR193C	9.7798
YLR033W	YOL087C	9.7789
YKR059W	YOR133W	9.7743
YDL216C	YOL088C	9.774
YMR240C	YOR308C	9.7734
YDR392W	YML015C	9.7704
YDR060W	YKR024C	9.7692
YML015C	YML098W	9.7689
YBR109C	YKL190W	9.7673
YER147C	YJL074C	9.7672
YER070W	YHR169W	9.7672
YLR430W	YMR239C	9.767
YMR125W	YNL251C	9.7647
YKL173W	YOR159C	9.759
YGL120C	YHR165C	9.7587
YDR473C	YPR182W	9.7586
YBL007C	YFR024C-A	9.7567
YHR052W	YKL009W	9.7522
YLR359W	YPR108W	9.7484
YKL165C	YPR108W	9.7484
YJR120W	YPR108W	9.7484
YJR062C	YPR108W	9.7484
YBR107C	YLR315W	9.7455
YOR116C	YOR304W	9.7439
YDL185W	YFR009W	9.7422
YKL173W	YMR240C	9.7365
YDR507C	YKR048C	9.7363
YMR128W	YPR144C	9.7361
YCR063W	YLR147C	9.7342
YBR039W	YKL016C	9.7339
YBR039W	YDR298C	9.7339
YKR001C	YLR357W	9.7321
YDL131W	YDL182W	9.7311
YGR275W	YNR023W	9.7307
YDR087C	YLL008W	9.7302
YLL036C	YLR132C	9.7291
YGR013W	YML049C	9.7272
YBR055C	YBR133C	9.7263
YER112W	YER146W	9.7259
YHR148W	YLR409C	9.725
YCR088W	YLR429W	9.7247
YIR012W	YLR075W	9.7218
YML007W	YPR070W	9.7208
YFL039C	YNL059C	9.7195
YNR039C	YOR174W	9.7148
YMR102C	YOR174W	9.7148
YCR014C	YNL180C	9.7148
YJR135C	YPR046W	9.7143
YGR275W	YPL016W	9.712
YGR097W	YNR047W	9.7096
YER029C	YLR117C	9.7082
YDR363W-A	YPR108W	9.7023
YER127W	YGR145W	9.6951
YFR013W	YPL082C	9.6925
YDR092W	YOL081W	9.6912
YGR245C	YPL093W	9.6909
YLR409C	YPR137W	9.6879
YBR282W	YNL252C	9.6875
YGL244W	YOL006C	9.6864
YGL011C	YKL206C	9.6845
YDR237W	YMR193W	9.6837
YER006W	YNL110C	9.6822
YFL002C	YOL077C	9.6811
YLR382C	YMR288W	9.6774
YHR110W	YML012W	9.6748
YDL018C	YML012W	9.6748
YNL118C	YNL147W	9.6719
YLR359W	YOR261C	9.6695
YKL165C	YOR261C	9.6695
YJR120W	YOR261C	9.6695
YJR062C	YOR261C	9.6695
YIL061C	YPR178W	9.6688
YOL111C	YOR164C	9.6652
YBR137W	YOL111C	9.6652
YER007W	YOR349W	9.6651
YER146W	YGL173C	9.665
YER018C	YKL173W	9.6639
YEL013W	YOR270C	9.6599
YDL175C	YMR239C	9.6599
YER136W	YLR039C	9.6596
YBR233W-A	YDR201W	9.6589
YAL003W	YGL245W	9.6568
YDR337W	YPL013C	9.6553
YKL172W	YOR206W	9.6499
YFL010C	YOR138C	9.6499
YDR214W	YEL060C	9.6498
YGR103W	YLL045C	9.6488
YAL013W	YMR075W	9.6446
YGL151W	YML007W	9.6423
YBR169C	YHR064C	9.6413
YBR264C	YGL210W	9.6404
YDR480W	YGR040W	9.639
YHR165C	YOR159C	9.6355
YHR075C	YOR014W	9.6352
YGR161C	YHR075C	9.6352
YPR057W	YPR178W	9.6338
YNL116W	YNL311C	9.6304
YDR449C	YPL217C	9.6283
YBR048W	YDL060W	9.6268
YDR017C	YER177W	9.6263
YBR009C	YPL082C	9.6196
YML060W	YNL189W	9.6139
YJL204C	YPR108W	9.6089
YGL252C	YLR085C	9.6077
YDR364C	YER172C	9.6072
YDR296W	YDR405W	9.6043
YDR116C	YDR405W	9.6043
YMR229C	YMR290C	9.6037
YMR239C	YPL212C	9.6006
YJL217W	YPR118W	9.6006
YHR156C	YOR238W	9.6006
YHR011W	YNL292W	9.6006
YDR079C-A	YPL122C	9.6006
YCL047C	YIL007C	9.6006
YER133W	YOR227W	9.5992
YLL008W	YLL034C	9.5947
YGR086C	YGR130C	9.5938
YGR074W	YNL147W	9.5928
YGR091W	YNL147W	9.5914
YNL132W	YPL012W	9.5889
YDR328C	YMR094W	9.5889
YBR203W	YDR328C	9.5889
YER110C	YHR020W	9.5886
YCR072C	YPR016C	9.5864
YKR085C	YMR024W	9.5834
YGL049C	YJL138C	9.5784
YBR185C	YCL032W	9.5757
YDL098C	YFL017W-A	9.5748
YDR176W	YPL047W	9.5729
YBR079C	YPL237W	9.5712
YGL200C	YHR110W	9.5699
YDL018C	YGL200C	9.5699
YJR093C	YOR179C	9.5698
YLL011W	YPL126W	9.5689
YGR283C	YLR175W	9.5675
YKL157W	YNL085W	9.5656
YHR041C	YML007W	9.5652
YBR087W	YCL042W	9.5644
YBL091C	YBR087W	9.5644
YMR049C	YNL110C	9.5634
YAL003W	YEL034W	9.5634
YDR050C	YHR174W	9.5617
YJL109C	YPL012W	9.561
YBL038W	YMR024W	9.561
YKL011C	YPR135W	9.5599
YDR190C	YFR037C	9.5595
YHR052W	YPR016C	9.5579
YLR197W	YMR128W	9.5573
YCR020W-B	YML127W	9.5568
YLR207W	YOL013C	9.556
YLL032C	YML119W	9.556
YHR146W	YMR196W	9.556
YHR051W	YNL052W	9.556
YGL187C	YHR051W	9.556
YFL023W	YJL179W	9.556
YEL043W	YGR089W	9.556
YDR057W	YOL013C	9.556
YDR057W	YLR207W	9.556
YDL089W	YML034W	9.556
YBR281C	YNL191W	9.556
YDR359C	YPL139C	9.5531
YGR091W	YOR159C	9.551
YLR362W	YPL049C	9.5472
YBR065C	YMR288W	9.5422
YCR030C	YOR178C	9.5406
YCR030C	YOL054W	9.5406
YKL092C	YLR182W	9.54
YMR273C	YPL152W	9.5373
YDR016C	YKR037C	9.536
YMR239C	YOL115W	9.5338
YBR017C	YOR160W	9.5338
YJL204C	YOR261C	9.5302
YJL033W	YNR054C	9.5278
YGL049C	YIR001C	9.5275
YBL090W	YHR059W	9.5253
YDR060W	YMR049C	9.521
YHR174W	YKL152C	9.5182
YDR359C	YNL330C	9.5172
YKR006C	YLR312W-A	9.5125
YKL167C	YLR312W-A	9.5125
YDL159W	YGR040W	9.5081
YMR093W	YNL132W	9.5075
YNL099C	YNR054C	9.5061
YNL032W	YNR054C	9.5061
YDR296W	YDR462W	9.5035
YDR116C	YDR462W	9.5035
YGL171W	YMR093W	9.5011
YGR128C	YOR063W	9.5003
YKR081C	YPR016C	9.4991
YCR071C	YLR312W-A	9.4921
YDR496C	YPL043W	9.4898
YDR101C	YHR170W	9.4898
YDR473C	YKL012W	9.4887
YIL079C	YPL146C	9.4886
YGR128C	YLR186W	9.4854
YJL145W	YLR403W	9.4828
YIL094C	YPR061C	9.4828
YDR023W	YKR071C	9.4828
YIL035C	YNL030W	9.4819
YDL147W	YLR359W	9.4815
YDL147W	YKL165C	9.4815
YDL147W	YJR120W	9.4815
YDL147W	YJR062C	9.4815
YAR007C	YBR136W	9.4812
YFR037C	YMR125W	9.4797
YFR028C	YOR340C	9.4724
YBR247C	YNL132W	9.4716
YAR007C	YGL133W	9.4705
YIL035C	YPL181W	9.4704
YGR074W	YJL203W	9.4703
YDL156W	YJL173C	9.4699
YGL100W	YPL169C	9.4672
YER086W	YGL245W	9.4662
YKR006C	YNL177C	9.4646
YKL167C	YNL177C	9.4646
YGL025C	YML007W	9.4638
YBL038W	YDR405W	9.4631
YGL128C	YPR101W	9.461
YDL185W	YKL048C	9.4589
YLR079W	YPL256C	9.4571
YDR318W	YLR315W	9.4519
YDL160C	YER146W	9.4465
YMR268C	YNL147W	9.4391
YBL050W	YDR189W	9.4382
YOL142W	YPR190C	9.435
YER126C	YHR197W	9.432
YBR079C	YDR091C	9.4311
YDR225W	YML069W	9.4299
YGR091W	YMR268C	9.4298
YBR006W	YDR175C	9.4298
YMR024W	YNL252C	9.4293
YJR022W	YOR159C	9.4276
YBR245C	YDR190C	9.4238
YGR090W	YOR253W	9.4195
YHL030W	YOR117W	9.419
YJL047C	YOL133W	9.4148
YDL148C	YOL010W	9.4142
YLR312W-A	YMR193W	9.412
YDL030W	YOR319W	9.4107
YBR233W-A	YKR037C	9.4095
YLR441C	YNL178W	9.4079
YKL173W	YOL069W	9.4062
YKR060W	YLR186W	9.4061
YLR196W	YMR290C	9.4044
YIL035C	YMR263W	9.4017
YEL037C	YER162C	9.4015
YDL108W	YPL090C	9.4
YGL120C	YPL151C	9.3991
YER006W	YOR206W	9.3945
YIR002C	YML032C	9.3922
YDR097C	YML032C	9.3922
YML074C	YMR311C	9.39
YGR090W	YLR186W	9.3883
YCL054W	YNL061W	9.3857
YFR004W	YMR314W	9.3848
YDL063C	YOL123W	9.3828
YMR288W	YPR082C	9.3813
YBR109C	YDR171W	9.3801
YMR200W	YOR021C	9.3775
YIL007C	YML106W	9.3775
YHR183W	YJR099W	9.3775
YFL022C	YGR094W	9.3775
YDR478W	YNL282W	9.3775
YCL047C	YML106W	9.3775
YGL112C	YPL047W	9.3769
YCR057C	YOR145C	9.3759
YBR282W	YDR296W	9.3741
YBR282W	YDR116C	9.3741
YDL190C	YPL096W	9.3739
YER094C	YKL206C	9.3735
YNL137C	YNR037C	9.3725
YER172C	YKL012W	9.3692
YDR190C	YOR304W	9.3687
YBL004W	YMR093W	9.3686
YBL004W	YDR449C	9.3686
YMR237W	YOR299W	9.3659
YLR330W	YOR299W	9.3659
YBR001C	YER177W	9.3651
YCR077C	YKL173W	9.3642
YGR245C	YPR016C	9.3639
YER133W	YLR182W	9.3635
YIL109C	YPL085W	9.3616
YHR200W	YMR314W	9.3585
YDR036C	YPL013C	9.3583
YKR048C	YLR410W	9.3502
YGR103W	YKL009W	9.3477
YKL081W	YLR249W	9.3455
YDR288W	YEL019C	9.345
YMR049C	YOR063W	9.3436
YFR004W	YLR359W	9.3434
YFR004W	YKL165C	9.3434
YFR004W	YJR120W	9.3434
YFR004W	YJR062C	9.3434
YDL147W	YJL204C	9.3426
YDL074C	YGL058W	9.3421
YDL160C	YMR268C	9.3414
YBR031W	YML063W	9.3393
YLR449W	YML074C	9.3349
YBR247C	YPL204W	9.3342
YGR179C	YPR046W	9.3336
YML014W	YOL124C	9.3328
YML014W	YNR046W	9.3328
YLR337C	YML001W	9.3328
YDR140W	YML014W	9.3328
YKR060W	YLR129W	9.3296
YAL038W	YBR127C	9.3288
YDL202W	YGR218W	9.3287
YMR235C	YOR185C	9.3278
YLR147C	YMR213W	9.3258
YDL014W	YER127W	9.3232
YKL059C	YOR179C	9.319
YDR324C	YLR186W	9.3187
YDL043C	YLR275W	9.3185
YMR086W	YPL004C	9.316
YBR282W	YCR046C	9.3153
YML025C	YNL284C	9.3143
YLR133W	YML069W	9.3117
YER095W	YMR167W	9.3093
YBR274W	YLR152C	9.3093
YBR006W	YBR251W	9.3087
YML098W	YMR236W	9.3064
YAL029C	YHR023W	9.3038
YDR207C	YPL181W	9.3026
YMR072W	YPR052C	9.3001
YKL193C	YOR227W	9.2999
YFL013C	YNL215W	9.2938
YFR013W	YGL133W	9.2928
YJL081C	YML041C	9.2926
YDL063C	YGL122C	9.2922
YBR017C	YDL063C	9.2922
YDL160C	YOR308C	9.2863
YGL070C	YPL129W	9.2847
YLR278C	YPR108W	9.2784
YHR196W	YPR137W	9.2771
YDR473C	YPL213W	9.2737
YDL126C	YFL044C	9.27
YLR243W	YPL249C	9.2677
YJR139C	YPL249C	9.2677
YIL156W	YPL249C	9.2677
YKR006C	YNL005C	9.2658
YKL167C	YNL005C	9.2658
YDR334W	YHR034C	9.2585
YLR197W	YNL189W	9.2575
YNL107W	YNL136W	9.2562
YJL140W	YPR110C	9.2516
YLR199C	YOR362C	9.2477
YML010W	YOR224C	9.2459
YMR112C	YPL042C	9.2451
YBR009C	YDR225W	9.245
YML010W	YPR187W	9.2424
YLR424W	YML049C	9.2401
YLR347C	YPL066W	9.2374
YBR025C	YKL081W	9.2311
YOR206W	YPL198W	9.2296
YDL176W	YDR255C	9.2187
YGL120C	YJR145C	9.2178
YJL033W	YNL132W	9.2172
YNL178W	YOR312C	9.2162
YDR176W	YGR274C	9.216
YBR009C	YDR224C	9.2152
YJR007W	YOR335C	9.2128
YDR045C	YNL113W	9.2117
YNR023W	YPL129W	9.2102
YER126C	YGR245C	9.2102
YHL011C	YKL181W	9.2096
YDL051W	YNL262W	9.2076
YBL049W	YDR255C	9.2054
YFR004W	YJL204C	9.2048
YML130C	YPR103W	9.2022
YGR284C	YPR103W	9.2022
YDR324C	YGR200C	9.2001
YDR237W	YOR150W	9.2
YFR051C	YPL219W	9.1983
YMR240C	YPR082C	9.1954
YBR257W	YDR478W	9.1952
YFR034C	YKR027W	9.1951
YDR346C	YPL111W	9.1951
YBR142W	YDR496C	9.1949
YCR088W	YFL039C	9.1947
YDL153C	YJR002W	9.1945
YBR105C	YDL176W	9.1918
YDR296W	YGR220C	9.1904
YDR116C	YGR220C	9.1904
YDR473C	YJL203W	9.1896
YCR035C	YNR024W	9.189
YLR278C	YOR261C	9.1872
YJL109C	YPR137W	9.186
YDL047W	YLR310C	9.1848
YDL098C	YER146W	9.1803
YKL173W	YPR057W	9.1789
YGR075C	YPR178W	9.1775
YGL043W	YPL129W	9.1771
YLR449W	YNL061W	9.1767
YDR364C	YPR101W	9.1766
YBL071W-A	YDR385W	9.1755
YBR245C	YLR033W	9.174
YLR002C	YPL211W	9.173
YDR496C	YPL093W	9.1691
YBL002W	YOL012C	9.1686
YOR141C	YOR189W	9.1662
YER021W	YER094C	9.1661
YLR186W	YLR409C	9.1659
YGL253W	YNL253W	9.1652
YCR063W	YDL209C	9.1644
YBR009C	YMR076C	9.1626
YOR159C	YOR308C	9.1585
YKL023W	YPR189W	9.1585
YDL148C	YDR324C	9.1556
YDR226W	YER078C	9.1544
YDR213W	YML111W	9.1544
YDR123C	YOL108C	9.1544
YBR223C	YGL082W	9.1544
YBL021C	YGL237C	9.1544
YCR053W	YLL034C	9.1543
YBR162C	YOR323C	9.1543
YDR238C	YPL219W	9.152
YMR312W	YOR358W	9.1505
YMR149W	YOR085W	9.1505
YAL012W	YMR300C	9.1505
YER178W	YGR193C	9.1445
YBR109C	YKL130C	9.1408
YGR020C	YOR270C	9.1396
YDR289C	YOR151C	9.1396
YBR189W	YDL060W	9.1395
YDL176W	YGL227W	9.139
YJR002W	YLR129W	9.1367
YDL002C	YNR035C	9.136
YMR193W	YNL005C	9.1351
YKL145W	YMR314W	9.1351
YDR127W	YKL211C	9.1337
YLR129W	YLR197W	9.1328
YCR046C	YGR220C	9.1316
YGR090W	YJL115W	9.13
YLR399C	YNL136W	9.1289
YBL074C	YHR156C	9.1255
YFR051C	YOL001W	9.1245
YER177W	YNR047W	9.1245
YML091C	YNL118C	9.1238
YDR394W	YIL107C	9.1192
YGL103W	YOL127W	9.118
YER048W-A	YKL192C	9.1151
YDR120C	YER049W	9.1151
YDL147W	YMR314W	9.1134
YDL160C	YGL173C	9.1118
YBR081C	YJL081C	9.1108
YCR063W	YDR482C	9.1104
YMR153W	YOR032C	9.1097
YDR458C	YNL280C	9.1097
YDR140W	YOL124C	9.1097
YDR140W	YNR046W	9.1097
YBL079W	YOR032C	9.1097
YER102W	YOR361C	9.1091
YGR103W	YNL182C	9.1065
YCR057C	YER082C	9.1037
YEL003W	YJL050W	9.0949
YLR208W	YPL169C	9.0934
YDR148C	YFR049W	9.0932
YBL004W	YLR186W	9.0932
YOR308C	YPL213W	9.0878
YIL035C	YJL087C	9.0867
YDR060W	YLL045C	9.0863
YER172C	YOR159C	9.0861
YBR122C	YNL005C	9.0841
YHR193C	YJR011C	9.0833
YGR134W	YHR193C	9.0833
YCR063W	YPL151C	9.0818
YBR152W	YDR473C	9.0802
YKL009W	YOR272W	9.0801
YDR238C	YOL001W	9.0783
YBR130C	YOR326W	9.0779
YKL216W	YLR113W	9.0774
YBR034C	YEL016C	9.0774
YJR022W	YKL173W	9.0754
YNL258C	YOL086C	9.0752
YLR440C	YOL086C	9.0752
YKR081C	YNR053C	9.0744
YKL210W	YOR184W	9.0741
YGR220C	YJL063C	9.0741
YGL128C	YMR288W	9.0738
YHR088W	YNL061W	9.073
YDR087C	YLL034C	9.0728
YML085C	YML124C	9.0727
YJL050W	YLR430W	9.0714
YHR029C	YMR096W	9.0705
YFL059W	YHR029C	9.0705
YGL115W	YOR267C	9.0699
YPL093W	YPL211W	9.0694
YKL196C	YLR268W	9.0671
YHR084W	YLR362W	9.0668
YLR392C	YMR275C	9.0652
YKR018C	YMR275C	9.0652
YGR136W	YMR275C	9.0652
YGR074W	YPL213W	9.0645
YDR303C	YKR001C	9.056
YLR288C	YOL094C	9.054
YDR485C	YJL081C	9.0536
YDR091C	YPR041W	9.0532
YBR205W	YOL123W	9.0522
YML049C	YPR101W	9.0488
YHR165C	YKR022C	9.0483
YER133W	YLR449W	9.0456
YKL172W	YOL077C	9.0449
YEL056W	YIL070C	9.0448
YDL098C	YMR268C	9.0418
YPL016W	YPL129W	9.0411
YJL180C	YNL315C	9.041
YJL082W	YMR132C	9.041
YGR125W	YIL034C	9.041
YBR268W	YGR218W	9.041
YKL014C	YOL144W	9.04
YBR196C	YMR186W	9.0377
YBR189W	YPL081W	9.0355
YNL059C	YNL215W	9.0335
YHR188C	YLR088W	9.0327
YFR049W	YIL125W	9.0305
YBR109C	YLL040C	9.0286
YIL022W	YJR045C	9.0274
YMR091C	YPL082C	9.0269
YBR289W	YGR275W	9.0267
YML049C	YMR125W	9.026
YBR198C	YCR042C	9.0231
YNL008C	YNL260C	9.0216
YLR396C	YMR291W	9.0216
YHR149C	YPL055C	9.0216
YDR339C	YGL006W	9.0216
YDR219C	YHR122W	9.0216
YDL074C	YHR149C	9.0216
YBR082C	YDR529C	9.0216
YCR042C	YGL112C	9.0175
YBR087W	YER173W	9.0167
YDL101C	YJL092W	9.0154
YGR074W	YMR125W	9.0153
YFL010C	YLL029W	9.0144
YKR028W	YNL106C	9.0131
YKL009W	YNR053C	9.0087
YER029C	YPL151C	9.0084
YKR085C	YLR312W-A	9.0076
YCR071C	YKL167C	9.0051
YFR037C	YGL133W	9.0037
YDR507C	YMR139W	9.0026
YKR062W	YMR075W	9.0003
YML007W	YPL042C	8.9975
YBR053C	YDR032C	8.9963
YLR106C	YNL182C	8.9926
YBL038W	YLR312W-A	8.9905
YFL002C	YLL034C	8.9899
YGR285C	YPR189W	8.9889
YDR060W	YPR131C	8.9881
YDR378C	YGR074W	8.9849
YOR206W	YPL093W	8.984
YDR449C	YOR145C	8.9827
YHL030W	YPL249C	8.9799
YHR165C	YKL012W	8.979
YER052C	YNL135C	8.9743
YJL011C	YOR340C	8.9729
YJL084C	YJR005W	8.972
YDL013W	YER116C	8.972
YBR257W	YIR015W	8.972
YBR049C	YGL108C	8.972
YBR017C	YLR426W	8.972
YBR160W	YGL003C	8.9706
YBR127C	YHR039C-A	8.9704
YDL147W	YLR278C	8.9701
YDL148C	YJL109C	8.9686
YAL036C	YGR090W	8.9678
YBR205W	YGL122C	8.9619
YKR085C	YNL177C	8.96
YLR172C	YOR133W	8.9588
YDL159W	YER167W	8.9564
YLR192C	YNL244C	8.9527
YKL193C	YLR258W	8.9524
YCR030C	YLL019C	8.9502
YER029C	YPR057W	8.9472
YBR247C	YNL035C	8.9467
YIL061C	YPL213W	8.9462
YEL031W	YOR157C	8.9444
YER017C	YGR231C	8.9419
YDR005C	YNL151C	8.9417
YHR052W	YPL043W	8.9406
YOR290C	YPL129W	8.9334
YHR085W	YNR053C	8.93
YFL038C	YOR089C	8.93
YBR083W	YHR084W	8.9271
YCR057C	YNL132W	8.9264
YKR006C	YMR193W	8.9253
YKL167C	YMR193W	8.9253
YDR092W	YOR220W	8.9228
YCR057C	YPR137W	8.9217
YMR075W	YPL181W	8.9209
YDL156W	YNL206C	8.9202
YFR051C	YGR233C	8.9185
YGL210W	YML001W	8.9152
YDR060W	YLL008W	8.9152
YDR071C	YDR247W	8.9141
YAL038W	YPR080W	8.9119
YBR143C	YOL086C	8.9115
YER012W	YOR261C	8.9083
YJL081C	YMR019W	8.9079
YER015W	YJL081C	8.9079
YDR146C	YJL081C	8.9079
YGR240C	YJL157C	8.9075
YCL057W	YPR002W	8.9075
YGL143C	YJR090C	8.9062
YCR071C	YMR193W	8.9051
YDR427W	YIL107C	8.9042
YKL013C	YOR141C	8.8995
YNR035C	YPL235W	8.8994
YLR347C	YOR244W	8.8974
YCR057C	YGR128C	8.8968
YDL203C	YLR371W	8.896
YGR285C	YLL026W	8.8953
YAR015W	YKR048C	8.8932
YLR312W-A	YNL252C	8.89
YBL004W	YDL148C	8.887
YLR186W	YMR128W	8.8863
YBR122C	YKR006C	8.8863
YBR122C	YKL167C	8.8863
YCR057C	YPL217C	8.8833
YDR280W	YHR139C	8.8784
YDL202W	YNL185C	8.8767
YDL195W	YMR300C	8.8767
YDL098C	YMR288W	8.8742
YKL152C	YLR044C	8.8736
YBR267W	YGL099W	8.8726
YLR002C	YLR449W	8.8723
YDR238C	YGR233C	8.8722
YBR196C	YLR249W	8.8708
YFL017W-A	YGR091W	8.8707
YBL035C	YGL143C	8.8707
YGR165W	YGR215W	8.8661
YBR122C	YCR071C	8.866
YDL115C	YPL129W	8.8646
YBR087W	YLR413W	8.8634
YBR155W	YGR187C	8.8628
YBR117C	YPR074C	8.8628
YGL098W	YKL196C	8.8611
YAL016W	YPL152W	8.8586
YNL182C	YNR053C	8.8581
YCR057C	YHR148W	8.8564
YDL097C	YOL018C	8.8512
YIL107C	YOR117W	8.8506
YJL034W	YMR214W	8.8502
YML046W	YPL178W	8.8497
YBL049W	YIL097W	8.8489
YBR188C	YMR213W	8.8464
YHR147C	YMR193W	8.8451
YDL185W	YKL104C	8.8425
YNL177C	YNL252C	8.8424
YMR033W	YOL087C	8.8418
YDR328C	YOL117W	8.8378
YDL047W	YER155C	8.8372
YDR225W	YGL241W	8.8371
YCR057C	YHR196W	8.8364
YBR282W	YLR439W	8.8341
YER050C	YPL013C	8.8327
YER172C	YMR213W	8.8319
YHR165C	YJR022W	8.8285
YHR039C-A	YLR447C	8.8276
YER021W	YFR050C	8.8275
YCL011C	YNL004W	8.8232
YLL024C	YNL236W	8.8185
YGR094W	YLR060W	8.8179
YLR075W	YNL132W	8.8178
YCR060W	YPL235W	8.8135
YBL052C	YFL024C	8.8075
YBL090W	YER050C	8.8069
YBR122C	YHR147C	8.8061
YDL014W	YHR013C	8.8058
YBL047C	YOL016C	8.8055
YNL215W	YPL129W	8.8041
YLL048C	YML070W	8.8022
YGR252W	YPL011C	8.8015
YER030W	YLR186W	8.7985
YDR080W	YLR248W	8.7985
YER165W	YOL139C	8.7983
YGR234W	YKL157W	8.7941
YBR169C	YGR285C	8.7929
YNL225C	YOR373W	8.7897
YNL181W	YPR072W	8.7897
YDR140W	YIL050W	8.7897
YCL044C	YGR140W	8.7897
YAL044C	YLR239C	8.7897
YDL014W	YLR196W	8.7879
YBR122C	YMR193W	8.7863
YGL210W	YKR014C	8.7847
YBL049W	YDL225W	8.7847
YBL049W	YCL039W	8.7847
YKR026C	YOR335C	8.7819
YDR188W	YPL151C	8.7769
YGR270W	YHR034C	8.7761
YDR496C	YHR066W	8.7748
YGR262C	YJL184W	8.7732
YCL059C	YNL132W	8.7726
YNR053C	YPR016C	8.7699
YER050C	YMR158W	8.7604
YBR055C	YNL147W	8.7588
YLR449W	YMR311C	8.7578
YGR080W	YJR065C	8.7575
YBR154C	YPL225W	8.7537
YNL244C	YOR361C	8.7529
YBR160W	YKR048C	8.7498
YBL003C	YPL082C	8.7474
YIL053W	YLR044C	8.7469
YHR109W	YJR139C	8.7451
YDR331W	YLR088W	8.7451
YLR382C	YML049C	8.7441
YGL066W	YMR227C	8.7429
YHR088W	YKL009W	8.7404
YCR012W	YDR050C	8.7403
YDL176W	YIL017C	8.7398
YNL215W	YOR141C	8.7393
YLL034C	YMR049C	8.7387
YIL053W	YKL060C	8.7353
YDL185W	YER171W	8.7349
YLR315W	YPL018W	8.7284
YGL206C	YNL084C	8.7279
YCL010C	YML015C	8.7253
YKL182W	YLR044C	8.7252
YLL036C	YPR191W	8.7234
YFL005W	YGL210W	8.7225
YGR090W	YPR144C	8.7195
YHR089C	YLR175W	8.7173
YDR012W	YMR290C	8.7171
YLR424W	YPR101W	8.7141
YDR087C	YMR290C	8.7132
YDL075W	YDR382W	8.7123
YBR264C	YML001W	8.7109
YKR078W	YLL005C	8.7097
YKL091C	YMR079W	8.7097
YHR076W	YKL091C	8.7097
YDL140C	YJR017C	8.7097
YER029C	YGR075C	8.7084
YDR138W	YNL189W	8.7064
YPL059W	YPL139C	8.7043
YBR109C	YLR433C	8.7035
YDL132W	YLR352W	8.7034
YDR073W	YHL025W	8.7033
YER146W	YPR178W	8.7022
YLR347C	YNL136W	8.7016
YJL050W	YNL153C	8.7007
YMR125W	YMR239C	8.6976
YAL032C	YKL173W	8.6972
YAR002W	YOR098C	8.6956
YBR109C	YOL016C	8.6921
YHR195W	YLR183C	8.691
YPR057W	YPR182W	8.6902
YBR282W	YDR322W	8.6901
YOR108W	YPR108W	8.6859
YDR028C	YDR099W	8.6852
YBR167C	YDR478W	8.6844
YAL012W	YLR328W	8.6844
YAL012W	YGR010W	8.6844
YJL154C	YKL195W	8.6843
YDR148C	YPL235W	8.6799
YCR073W-A	YER177W	8.6764
YGR283C	YHR089C	8.6724
YBL004W	YER082C	8.6713
YGR274C	YLR055C	8.6702
YLR291C	YOR335C	8.6679
YDR143C	YOR217W	8.6654
YLR105C	YOR187W	8.665
YOR078W	YOR310C	8.6647
YCR072C	YLR074C	8.6647
YDR181C	YMR127C	8.6596
YML092C	YNR003C	8.6594
YNL308C	YPL012W	8.6593
YML091C	YOL149W	8.6575
YIL070C	YKL108W	8.6565
YDR416W	YGL120C	8.6552
YNL005C	YOR150W	8.6548
YGR220C	YLR439W	8.6512
YBR245C	YMR072W	8.6494
YML007W	YMR112C	8.6474
YAL005C	YBR143C	8.6415
YBR057C	YGL036W	8.6397
YLL011W	YPR137W	8.6388
YFL024C	YPR031W	8.637
YER082C	YLR222C	8.6367
YER082C	YLR129W	8.6367
YBL074C	YPR182W	8.6364
YDL176W	YMR135C	8.6362
YNL220W	YOR187W	8.6356
YIL020C	YLR179C	8.6356
YHR015W	YPL091W	8.6356
YGL050W	YIL050W	8.6356
YDR140W	YGL050W	8.6356
YGR013W	YPL213W	8.6314
YAL013W	YPR023C	8.6295
YLL024C	YPR080W	8.628
YFR052W	YLR278C	8.6263
YER173W	YNL290W	8.6261
YBL072C	YJR145C	8.6254
YLR208W	YNL049C	8.6227
YCL059C	YDL213C	8.6211
YBL004W	YPR144C	8.6167
YPL016W	YPL195W	8.6162
YMR323W	YOR351C	8.6162
YMR167W	YOR155C	8.6162
YLR229C	YPL161C	8.6162
YJL044C	YMR218C	8.6162
YHR120W	YLR163C	8.6162
YHR024C	YHR120W	8.6162
YFR014C	YKL119C	8.6162
YDR516C	YNL032W	8.6162
YBR288C	YPL016W	8.6162
YML016C	YOR308C	8.6161
YBR276C	YLR359W	8.6161
YBR195C	YKR029C	8.6161
YJL050W	YLR424W	8.6133
YCR092C	YJL173C	8.611
YNL110C	YOR206W	8.6089
YOR108W	YOR261C	8.6085
YFL017W-A	YOR308C	8.6069
YBR031W	YNL112W	8.6068
YDR028C	YOR227W	8.6022
YLR222C	YPR144C	8.6
YLR129W	YPR144C	8.6
YDL030W	YJR050W	8.6
YCR063W	YGL128C	8.5973
YLR029C	YOR206W	8.5948
YHR193C	YNL247W	8.5936
YAL026C	YAL053W	8.5909
YDR365C	YJL033W	8.5897
YHR034C	YJR065C	8.5895
YGR017W	YLR197W	8.5865
YDR388W	YGL060W	8.5843
YBR221C	YMR314W	8.582
YBR264C	YKR014C	8.5805
YBR061C	YBR127C	8.5804
YDR050C	YLR044C	8.5797
YDR496C	YLR196W	8.5786
YDR296W	YLR312W-A	8.5785
YCR057C	YDL014W	8.5772
YOL088C	YOL117W	8.5708
YDL097C	YMR314W	8.5674
YHR051W	YIL111W	8.5666
YCR065W	YLR417W	8.5666
YCR065W	YJR102C	8.5666
YHR196W	YLL011W	8.5659
YDL209C	YMR213W	8.5602
YDL043C	YKL173W	8.5598
YER172C	YFL017W-A	8.5582
YIL069C	YNR054C	8.5577
YJL164C	YNL227C	8.5575
YCR012W	YGR254W	8.5568
YDR496C	YOR063W	8.5567
YBR009C	YDL156W	8.555
YJL115W	YPL128C	8.5469
YER111C	YJL115W	8.5469
YER089C	YJL115W	8.5469
YML049C	YMR213W	8.5456
YER006W	YOR272W	8.5454
YFR028C	YOR341W	8.5452
YDR494W	YER050C	8.545
YBR115C	YGL154C	8.5444
YER022W	YLL024C	8.5443
YHR148W	YJL069C	8.5427
YDR473C	YKL188C	8.5424
YLR424W	YPL213W	8.5397
YCR071C	YOR150W	8.5367
YGL242C	YJR002W	8.5357
YER165W	YOL115W	8.5351
YDR432W	YNL251C	8.5322
YDL043C	YLR117C	8.5311
YDR296W	YNL177C	8.531
YDR116C	YNL177C	8.531
YLR424W	YPL208W	8.523
YCR009C	YNL208W	8.5221
YGR090W	YGR128C	8.5211
YDR091C	YMR309C	8.5211
YCR046C	YLR312W-A	8.5201
YNL039W	YPR110C	8.5198
YJL176C	YNL027W	8.5197
YBR264C	YFL005W	8.5183
YBL003C	YPL128C	8.5181
YOR204W	YOR312C	8.5175
YNL004W	YNL253W	8.5171
YKL003C	YPL013C	8.5147
YLR175W	YLR197W	8.5146
YDL003W	YMR001C	8.5127
YDR480W	YER093C	8.5099
YDR001C	YDR028C	8.5098
YDR381W	YHR167W	8.5089
YEL030W	YOR378W	8.5076
YEL030W	YLR276C	8.5076
YDL164C	YEL030W	8.5076
YDR322W	YGR220C	8.5074
YBL038W	YKL167C	8.5055
YCR071C	YKR085C	8.5023
YIL149C	YLR132C	8.5021
YGL232W	YPL212C	8.5021
YHR148W	YLR197W	8.5013
YBL105C	YGR254W	8.4999
YDL176W	YIL097W	8.4998
YGR135W	YNR003C	8.4968
YCR030C	YOR056C	8.496
YBR084W	YGR204W	8.4954
YBL087C	YGL111W	8.4941
YGR215W	YHR059W	8.4919
YLR259C	YPL247C	8.4881
YNR003C	YOL038W	8.4874
YDR364C	YGL128C	8.4789
YGR231C	YJR075W	8.4788
YJR022W	YLR275W	8.4781
YBL041W	YNR003C	8.4781
YKL009W	YMR049C	8.4766
YBR247C	YPL217C	8.4765
YLR449W	YMR128W	8.4751
YKR048C	YMR049C	8.4736
YLR413W	YNL290W	8.473
YCR046C	YNL177C	8.4726
YLR263W	YMR314W	8.4715
YAL053W	YMR314W	8.4715
YDR188W	YOR281C	8.4711
YBR081C	YML015C	8.4705
YJL203W	YPR082C	8.4634
YGR063C	YOR224C	8.4634
YLR310C	YOR267C	8.4624
YGL197W	YOR267C	8.4624
YLR347C	YMR308C	8.4623
YBR059C	YPR033C	8.462
YJR144W	YNL312W	8.4619
YBR152W	YOR308C	8.4616
YLL062C	YOR064C	8.4612
YBL032W	YGL105W	8.4612
YCR063W	YDR416W	8.4604
YDR496C	YLR175W	8.4587
YLR403W	YOR347C	8.4574
YDR346C	YHR112C	8.4574
YDR051C	YER048W-A	8.4574
YGR128C	YLR129W	8.456
YDR194C	YKR081C	8.4523
YLL034C	YMR290C	8.4498
YDR378C	YOR159C	8.4483
YBR189W	YDR064W	8.4471
YML007W	YOL135C	8.4466
YEL055C	YGL242C	8.4459
YDR381W	YNL139C	8.4442
YLL026W	YPL240C	8.4401
YGL095C	YOL018C	8.4368
YDR468C	YOL018C	8.4368
YDR468C	YGL095C	8.4368
YLR002C	YOR243C	8.4364
YJL050W	YPL146C	8.4361
YER177W	YLR258W	8.4249
YDL147W	YOR108W	8.424
YKL041W	YLR025W	8.422
YIL098C	YKL192C	8.422
YAR015W	YBR267W	8.422
YDL030W	YLR424W	8.4218
YHR148W	YLR222C	8.4207
YHR148W	YLR129W	8.4207
YBR122C	YOR150W	8.4183
YDR198C	YDR359C	8.4171
YIL128W	YOR204W	8.4166
YJR050W	YMR288W	8.4154
YJL063C	YNL177C	8.4154
YMR308C	YNL206C	8.4128
YNL312W	YOL090W	8.4106
YBL052C	YPR023C	8.41
YFL039C	YLR385C	8.4097
YBL038W	YMR193W	8.4059
YDL087C	YML049C	8.4057
YDR141C	YFR051C	8.4044
YDL047W	YOR267C	8.4003
YDR050C	YMR186W	8.3997
YCR033W	YLR409C	8.3955
YHR023W	YOR326W	8.3923
YDR328C	YLR352W	8.3912
YLR197W	YMR229C	8.3904
YDR496C	YKL172W	8.3902
YJL076W	YJR063W	8.3897
YER177W	YGR097W	8.3897
YHR052W	YLL034C	8.3891
YGR278W	YML049C	8.3881
YGR255C	YPR023C	8.3854
YLR079W	YPR119W	8.3843
YHR102W	YNL267W	8.3843
YAL044W-A	YPL059W	8.3843
YBR122C	YKR085C	8.384
YDR012W	YOR206W	8.3781
YFL023W	YNL153C	8.3774
YER125W	YKR021W	8.3773
YDL143W	YOR281C	8.3743
YIL026C	YMR001C	8.3706
YBR282W	YMR024W	8.3696
YHR197W	YMR290C	8.3689
YGR090W	YLR197W	8.3689
YER167W	YPL049C	8.3682
YEL022W	YHR020W	8.3676
YBL038W	YBR122C	8.367
YDR207C	YNL330C	8.3666
YAL033W	YIR015W	8.3659
YGR086C	YMR031C	8.3623
YDR141C	YDR238C	8.3582
YKR024C	YLR196W	8.356
YBR221C	YGR193C	8.3553
YLL034C	YLR002C	8.3548
YJL180C	YJR121W	8.3479
YGL110C	YKR023W	8.3479
YDR164C	YLR208W	8.3479
YCL024W	YMR132C	8.3479
YGL043W	YLR095C	8.347
YDR207C	YPL139C	8.3445
YBR247C	YMR128W	8.3413
YFL037W	YGR218W	8.3408
YGL167C	YNL169C	8.3396
YDR006C	YJL146W	8.3396
YDL033C	YGL039W	8.3396
YDR052C	YGL048C	8.3393
YLL034C	YOL077C	8.3364
YEL018W	YMR075W	8.3357
YHR066W	YKL014C	8.334
YCR020W-B	YLR033W	8.3313
YDL111C	YHR139C	8.3309
YDL030W	YDR482C	8.3309
YBL052C	YLR455W	8.3307
YGR052W	YLR127C	8.3285
YGL192W	YOR125C	8.3285
YDR310C	YHR152W	8.3285
YCL030C	YLR127C	8.3285
YBL041W	YPR108W	8.3276
YAL032C	YBR188C	8.3262
YHR147C	YNL252C	8.3256
YDR432W	YIR001C	8.3248
YJL203W	YLR275W	8.3231
YNL002C	YPL211W	8.3221
YOR145C	YPL126W	8.3216
YAL015C	YEL060C	8.3216
YHR165C	YPR101W	8.3198
YLL029W	YOR124C	8.3176
YHR059W	YJR101W	8.3164
YDR073W	YNR023W	8.3157
YCR052W	YDR225W	8.3137
YHR156C	YLR147C	8.3135
YAL035W	YGL049C	8.3125
YHR139C	YOL142W	8.3099
YDR385W	YKL060C	8.3071
YGR145W	YPR144C	8.3035
YGL190C	YPL152W	8.303
YBR188C	YLR117C	8.3022
YOR224C	YPL129W	8.3021
YHR197W	YLR106C	8.2988
YOL041C	YOL077C	8.2932
YCR072C	YER126C	8.2928
YBR048W	YNL207W	8.2924
YML025C	YNL177C	8.2907
YFR004W	YOR108W	8.2885
YMR029C	YMR052W	8.2856
YHR118C	YPL001W	8.2844
YDR324C	YNL132W	8.2832
YBR031W	YNL178W	8.2827
YHR059W	YNL306W	8.2825
YDL014W	YPR131C	8.282
YER146W	YOR159C	8.2816
YGL111W	YNL110C	8.2796
YBL074C	YER172C	8.2794
YGR072W	YHR077C	8.2789
YDL004W	YPL160W	8.2789
YBR167C	YBR257W	8.2789
YBL038W	YGR093W	8.2789
YBL032W	YLR180W	8.2789
YBL004W	YCR057C	8.2734
YLL008W	YPR016C	8.273
YAL038W	YOL086C	8.2719
YDR237W	YDR405W	8.2717
YDR343C	YOL100W	8.2715
YDR343C	YGR033C	8.2715
YIL109C	YPR181C	8.2712
YDR030C	YIL142W	8.2695
YGR002C	YPL139C	8.2686
YFR030W	YHR027C	8.2682
YGR145W	YJL033W	8.2651
YDR314C	YPR108W	8.2651
YGL128C	YJL050W	8.2618
YDR517W	YMR314W	8.2617
YOR335C	YPL237W	8.2591
YCL014W	YLR189C	8.2584
YHR020W	YKL104C	8.2572
YDR443C	YML007W	8.2565
YDL150W	YHR069C	8.2556
YDR324C	YPR144C	8.2543
YDR153C	YPR189W	8.2536
YJR032W	YKL173W	8.2502
YGR270W	YJL115W	8.2482
YER050C	YMR188C	8.2478
YAL038W	YDL047W	8.2478
YER029C	YLR424W	8.2473
YHL047C	YML114C	8.246
YCR042C	YHL047C	8.246
YCR057C	YMR128W	8.2433
YPR023C	YPR031W	8.2402
YBL026W	YGR075C	8.2358
YNL315C	YPR072W	8.2343
YFL023W	YGR078C	8.2343
YCR077C	YER146W	8.231
YGR068C	YIL094C	8.2302
YER107C	YJL122W	8.2302
YNL317W	YOR179C	8.2287
YKL182W	YMR186W	8.2277
YBL074C	YLR147C	8.2258
YLR222C	YMR128W	8.2253
YDR188W	YGR187C	8.2253
YBR114W	YHL027W	8.2227
YNR035C	YOR141C	8.2225
YJR051W	YMR314W	8.2216
YDR359C	YMR075W	8.2208
YLR455W	YPR031W	8.2161
YJL203W	YOR308C	8.2161
YKL172W	YMR049C	8.2152
YNL093W	YOR370C	8.2147
YNL019C	YOR168W	8.2144
YNL015W	YOR168W	8.2144
YNL015W	YNL019C	8.2144
YEL054C	YOR293W	8.2144
YDR225W	YMR091C	8.2141
YDR167W	YML098W	8.2128
YNL110C	YPR016C	8.2119
YDR381W	YPL129W	8.211
YBL003C	YCR030C	8.2109
YNL307C	YOR266W	8.2107
YML029W	YNL329C	8.2107
YLR450W	YOL113W	8.2107
YKR017C	YLR138W	8.2107
YIL105C	YOR266W	8.2107
YIL105C	YNL307C	8.2107
YHR171W	YOR191W	8.2107
YHL010C	YLR138W	8.2107
YHL010C	YKR017C	8.2107
YGL220W	YLL029W	8.2107
YER164W	YPR188C	8.2107
YDR169C	YML112W	8.2107
YDL190C	YPL236C	8.2107
YIL107C	YPR108W	8.2102
YFL007W	YJL001W	8.2074
YDL097C	YPL002C	8.2074
YLR275W	YMR240C	8.2071
YKL172W	YNL175C	8.2063
YLL045C	YOR206W	8.2045
YDL131W	YLR075W	8.2042
YOL077C	YPL043W	8.2031
YKL172W	YNL002C	8.2023
YDR073W	YPL016W	8.2018
YCL017C	YKL192C	8.1989
YHR088W	YOL077C	8.197
YDR087C	YGL111W	8.1967
YJL090C	YNL189W	8.1965
YHR167W	YNL004W	8.1964
YER133W	YJL042W	8.1955
YNL247W	YPL037C	8.195
YDL051W	YDR395W	8.1949
YML007W	YNL236W	8.1921
YDL030W	YGR013W	8.1913
YGR220C	YMR024W	8.1875
YBR245C	YFL039C	8.1869
YCR081W	YML007W	8.186
YBL050W	YOL018C	8.1855
YBL050W	YGL095C	8.1855
YBL050W	YDR468C	8.1855
YDR473C	YML046W	8.1842
YCR066W	YMR100W	8.1836
YDR054C	YJR090C	8.1828
YPR103W	YPR108W	8.1819
YMR172W	YOR361C	8.1801
YMR246W	YOL126C	8.1788
YGL111W	YHR197W	8.1784
YDR496C	YMR229C	8.1765
YMR128W	YOL010W	8.1743
YBR055C	YDL160C	8.1734
YDL014W	YKR081C	8.1714
YLR356W	YPL055C	8.1698
YJL115W	YJR140C	8.1698
YDL074C	YLR356W	8.1698
YER082C	YPL217C	8.1692
YGR002C	YHR099W	8.1671
YBR218C	YGL062W	8.165
YER029C	YHR156C	8.1642
YBR146W	YHR059W	8.163
YGR162W	YIR001C	8.1626
YGR275W	YOR290C	8.1622
YDL014W	YLR175W	8.1599
YDL220C	YJL115W	8.1579
YLL011W	YNL132W	8.1558
YCR072C	YER006W	8.1558
YDR320C	YPR049C	8.1542
YDR035W	YDR375C	8.1542
YDL215C	YOL034W	8.1538
YOL041C	YPL043W	8.1525
YDR050C	YKL152C	8.1453
YGR128C	YPR144C	8.1447
YAL032C	YER172C	8.1424
YDR448W	YHR058C	8.1402
YDR473C	YGR013W	8.137
YDR101C	YLR074C	8.1356
YFL033C	YML127W	8.1344
YPL217C	YPR144C	8.133
YBL072C	YOR293W	8.1311
YCR072C	YLR325C	8.128
YOR204W	YPL249C	8.1271
YDR378C	YFL017W-A	8.1271
YDL098C	YMR240C	8.1264
YGL113W	YJR070C	8.1248
YER111C	YOR083W	8.1248
YDR101C	YER107C	8.1248
YDL226C	YDR083W	8.1248
YAL012W	YMR099C	8.1248
YHR013C	YNL178W	8.1223
YML109W	YOR014W	8.122
YBR136W	YOL090W	8.122
YDL159W	YLR154C	8.1211
YFR028C	YOR224C	8.1177
YDL150W	YOR001W	8.1171
YLR398C	YOR076C	8.1102
YBL104C	YGL100W	8.1098
YJL002C	YOR085W	8.1091
YHR186C	YJR066W	8.1091
YGL022W	YOR085W	8.1091
YBR245C	YCR052W	8.1077
YDR087C	YER006W	8.1062
YGR263C	YLR433C	8.1054
YDL013W	YOR208W	8.1054
YBL064C	YDR424C	8.1054
YAL017W	YAL019W	8.1054
YKL216W	YKR055W	8.1053
YGR231C	YMR089C	8.1039
YKL172W	YNL110C	8.1025
YCR057C	YLL011W	8.1009
YDR017C	YDR099W	8.1008
YLR085C	YNL107W	8.0999
YFR021W	YHR070W	8.0966
YDR322C-A	YOR335C	8.0966
YBR248C	YLR401C	8.0966
YGL241W	YMR284W	8.0955
YER017C	YGR132C	8.0954
YDR328C	YGL143C	8.0947
YHR143W-A	YPR187W	8.0944
YDL043C	YPR178W	8.0911
YDL126C	YGL252C	8.0898
YFR048W	YMR186W	8.0891
YMR158W	YPL013C	8.0874
YFL013C	YGR002C	8.0873
YAL032C	YMR288W	8.0858
YLR449W	YPL093W	8.085
YBR160W	YIL142W	8.0849
YDR101C	YGL099W	8.0848
YFR052W	YMR314W	8.0832
YDR190C	YKL013C	8.0832
YBR088C	YDL101C	8.0827
YOR038C	YPL001W	8.0817
YAL021C	YDR252W	8.0813
YAR003W	YBR258C	8.0809
YBR057C	YKR031C	8.0801
YBR142W	YNL061W	8.0786
YDR005C	YKR025W	8.078
YDL045W-A	YGR215W	8.0764
YCR063W	YDL030W	8.0749
YDR457W	YER149C	8.074
YER172C	YIL061C	8.0697
YMR290C	YOL041C	8.0695
YBR152W	YGR091W	8.0661
YDR153C	YGL213C	8.0656
YKR085C	YOR150W	8.0562
YKL167C	YPL173W	8.0558
YCR053W	YML126C	8.0558
YKL172W	YKR081C	8.0542
YPL018W	YPL139C	8.0537
YLR203C	YOR232W	8.052
YIL034C	YMR109W	8.0519
YHR064C	YKL081W	8.0472
YER167W	YHR084W	8.0461
YHR085W	YLR106C	8.0459
YBR065C	YGL128C	8.0459
YER029C	YLR382C	8.0443
YBR282W	YDR237W	8.0441
YDL031W	YDR060W	8.0433
YPL117C	YPL226W	8.0426
YLR312W-A	YLR439W	8.0423
YGL043W	YLR189C	8.0381
YCL011C	YDR432W	8.0327
YGR090W	YLR409C	8.0297
YJR041C	YKL214C	8.0295
YGR218W	YNL185C	8.0295
YFR028C	YOR210W	8.0292
YLL029W	YOR138C	8.0291
YBR136W	YML032C	8.0291
YJL080C	YKL040C	8.0289
YLR335W	YOR098C	8.0268
YMR240C	YOR159C	8.0233
YDR240C	YDR473C	8.0232
YBR055C	YLL036C	8.0231
YOL148C	YPL011C	8.0203
YLR194C	YNL050C	8.0166
YJR105W	YKL004W	8.0166
YDL014W	YLR409C	8.0164
YDR296W	YHR147C	8.0157
YDR116C	YHR147C	8.0157
YGR013W	YKL173W	8.0127
YDR224C	YDR225W	8.0124
YJR009C	YML032C	8.0108
YLR288C	YOR368W	8.01
YDL147W	YDR314C	8.0054
YDL126C	YMR067C	8.0054
YDL126C	YDR330W	8.0054
YDR432W	YNL016W	8.0047
YGL049C	YML117W	8.0022
YFL037W	YNL064C	8.0014
YLR430W	YOL115W	7.9987
YAR042W	YHR195W	7.9979
YBR085W	YER062C	7.9966
YGL128C	YMR213W	7.9963
YCL059C	YLR175W	7.9952
YLR439W	YNL177C	7.9951
YDR385W	YOL086C	7.9945
YHR164C	YJL173C	7.9941
YGR086C	YLR305C	7.9939
YDR099W	YGL252C	7.9922
YBR087W	YDR394W	7.9921
YJL065C	YNL230C	7.9913
YCL017C	YKL130C	7.9913
YBR218C	YJL092W	7.9913
YKL009W	YMR290C	7.9912
YHR196W	YLR222C	7.9905
YJR050W	YML049C	7.9903
YNL132W	YNL308C	7.9898
YCR063W	YLL036C	7.9894
YEL055C	YLR196W	7.9889
YHR069C	YNL151C	7.9864
YHR064C	YPR189W	7.9851
YBR136W	YJL173C	7.9843
YBR193C	YML007W	7.9826
YER006W	YLL034C	7.9819
YFR037C	YMR072W	7.9807
YDL065C	YDL160C	7.9807
YDR432W	YPL178W	7.979
YOL063C	YOL133W	7.9789
YNL272C	YPR117W	7.9789
YLR190W	YPL219W	7.9789
YGR062C	YPR067W	7.9789
YDR059C	YER068W	7.9789
YCR084C	YKL213C	7.9789
YAR075W	YPR067W	7.9789
YAR075W	YGR062C	7.9789
YHR090C	YHR099W	7.9788
YLR259C	YOR230W	7.9779
YGR002C	YLR085C	7.9759
YGL133W	YIL126W	7.9752
YHR088W	YPL093W	7.9745
YGR245C	YHR085W	7.9728
YGR104C	YNL025C	7.9727
YMR138W	YMR192W	7.972
YBR170C	YML013W	7.9719
YJL109C	YKR060W	7.9718
YDR060W	YER165W	7.9661
YMR239C	YPL190C	7.9638
YKR024C	YPR131C	7.9634
YLR293C	YMR235C	7.9632
YBR130C	YIL034C	7.9623
YMR268C	YPR082C	7.9604
YAL005C	YDR517W	7.9596
YNL002C	YNL182C	7.9578
YCR046C	YHR147C	7.9577
YLR002C	YPR016C	7.9562
YHR156C	YKL173W	7.9554
YER029C	YPR101W	7.9544
YGL128C	YML049C	7.9539
YER006W	YLR074C	7.9522
YER021W	YLR278C	7.9515
YBR245C	YMR091C	7.9505
YPL012W	YPL043W	7.9503
YEL056W	YML065W	7.9476
YJL111W	YMR028W	7.9466
YDR484W	YKR020W	7.9466
YDR027C	YKR020W	7.9466
YDR462W	YLR312W-A	7.9463
YHR099W	YMR223W	7.9456
YLR208W	YMR300C	7.9425
YBR273C	YMR067C	7.9425
YBR273C	YDR330W	7.9425
YAL029C	YFL039C	7.942
YLR186W	YLR222C	7.9418
YLR129W	YLR186W	7.9418
YLR002C	YOL120C	7.9417
YER092W	YFL039C	7.9407
YNL252C	YOR150W	7.9397
YDL007W	YIL107C	7.9387
YNL075W	YPR137W	7.9375
YAR002W	YER110C	7.9346
YPL183C	YPR006C	7.9342
YLR144C	YPL014W	7.9342
YKR091W	YPL014W	7.9342
YKR091W	YLR144C	7.9342
YJR070C	YLR168C	7.9342
YFL046W	YGR284C	7.9342
YJR002W	YOR078W	7.9341
YDL055C	YJL039C	7.9338
YDR328C	YMR025W	7.9327
YNL069C	YNL301C	7.932
YER148W	YHL047C	7.9309
YDL043C	YGL174W	7.9309
YMR005W	YPL254W	7.9308
YHR062C	YIR015W	7.9307
YGL066W	YMR005W	7.9288
YHR088W	YLR449W	7.9284
YGR282C	YMR145C	7.9283
YNL128W	YPL164C	7.9231
YJL068C	YOR125C	7.9231
YDL150W	YPR010C	7.9224
YEL022W	YJR077C	7.9212
YDL115C	YML010W	7.9202
YBR127C	YGR020C	7.9196
YER165W	YMR125W	7.9191
YCL059C	YDL060W	7.9185
YDL143W	YJL111W	7.9184
YLR342W	YML072C	7.916
YHR137W	YLR059C	7.9155
YML009C	YMR225C	7.9154
YHR059W	YPL118W	7.9153
YDR473C	YGR075C	7.9124
YHR052W	YNL110C	7.9112
YHR059W	YKL155C	7.9111
YCL032W	YDR121W	7.9104
YDL229W	YGR071C	7.9103
YDL126C	YGR192C	7.91
YBR065C	YDR416W	7.9098
YGR103W	YNR053C	7.9071
YOR063W	YOR272W	7.9046
YDR448W	YPR070W	7.9038
YOR272W	YOR312C	7.903
YDL045W-A	YJR101W	7.9003
YGR162W	YGR250C	7.8999
YPL181W	YPR023C	7.8996
YGL111W	YKL172W	7.8992
YDR214W	YJR068W	7.8989
YOR168W	YOR352W	7.8979
YIL143C	YOR168W	7.8979
YDR473C	YMR288W	7.8979
YDR434W	YHR188C	7.8978
YEL047C	YLR058C	7.8959
YGR145W	YPL198W	7.8958
YGL106W	YMR109W	7.8937
YLR275W	YNL147W	7.8924
YLR347C	YPL190C	7.8922
YDL098C	YJR022W	7.8919
YCR063W	YMR288W	7.8918
YFR052W	YOL018C	7.891
YDR388W	YMR192W	7.89
YPL178W	YPL190C	7.8893
YFL033C	YLR321C	7.8891
YJL141C	YPL247C	7.8868
YJL141C	YLR270W	7.8868
YJL141C	YKR001C	7.8868
YDR320C	YLR423C	7.886
YDR347W	YHR059W	7.885
YPL129W	YPR034W	7.8772
YIL034C	YKL130C	7.8765
YBR169C	YDR171W	7.8761
YBR001C	YDR171W	7.8761
YGL128C	YGR278W	7.8758
YMR128W	YPR137W	7.8749
YIR015W	YNL221C	7.8735
YDR453C	YGR185C	7.8735
YDL035C	YGR262C	7.8735
YML015C	YPL254W	7.8728
YDR314C	YFR004W	7.8712
YGL120C	YPR101W	7.8687
YER029C	YER146W	7.8671
YAR007C	YDR300C	7.867
YMR239C	YNL251C	7.8668
YIL035C	YPL090C	7.8658
YGR272C	YMR014W	7.8652
YGL004C	YLR421C	7.8641
YDR449C	YHR196W	7.863
YML057W	YNL047C	7.8627
YDR237W	YGR220C	7.8627
YLR039C	YNL044W	7.8625
YJL180C	YKL192C	7.8625
YJL034W	YJR045C	7.8601
YKR014C	YML001W	7.8577
YBR001C	YDR099W	7.8568
YJR002W	YLL011W	7.8565
YPL198W	YPL220W	7.855
YLL008W	YLR347C	7.8531
YJL203W	YLL036C	7.8531
YDR322W	YNL177C	7.8524
YLR074C	YLR325C	7.8523
YFL033C	YKR008W	7.8522
YBR247C	YNR050C	7.8515
YCL024W	YDR381W	7.8506
YEL032W	YLR274W	7.8493
YNL151C	YOR001W	7.8486
YBL049W	YMR135C	7.8471
YNR037C	YPL013C	7.847
YKL003C	YNR037C	7.847
YGR215W	YKL003C	7.847
YJL076W	YOR210W	7.8469
YDR137W	YLL024C	7.8447
YKL119C	YLR447C	7.8446
YDR494W	YJR101W	7.8444
YDL006W	YNR019W	7.843
YBR122C	YJL063C	7.8426
YLL008W	YPL043W	7.8416
YKR060W	YMR128W	7.8415
YBR127C	YPR036W	7.841
YGR233C	YLR190W	7.8407
YDR005C	YPR187W	7.8406
YFR051C	YPL031C	7.8399
YDR482C	YMR240C	7.8395
YGL137W	YMR224C	7.8344
YAL038W	YDL055C	7.8344
YDR224C	YGL207W	7.8332
YPL151C	YPR134W	7.83
YOR206W	YPL211W	7.83
YDL155W	YLR210W	7.8289
YBR143C	YJL026W	7.8253
YMR128W	YNL132W	7.8251
YHR088W	YLL034C	7.8249
YDL029W	YHR034C	7.8249
YDR190C	YGL133W	7.8244
YFL017W-A	YPR182W	7.8228
YHR143W-A	YPL129W	7.8227
YIL156W	YJL020C	7.8195
YGR019W	YGR211W	7.8195
YGR245C	YNL002C	7.8193
YBR125C	YDR247W	7.8169
YGR232W	YOL018C	7.8096
YCR052W	YKR001C	7.8096
YBR001C	YBR127C	7.8087
YER031C	YGL210W	7.8082
YCR088W	YPL004C	7.8075
YMR235C	YMR300C	7.8069
YBR211C	YJR112W	7.8069
YKR023W	YPL139C	7.8067
YMR268C	YOR308C	7.8035
YBR198C	YML098W	7.8024
YHR027C	YHR114W	7.8013
YBL007C	YHR027C	7.8013
YBR188C	YLL036C	7.8011
YOR272W	YPL211W	7.8005
YGR232W	YLR278C	7.8005
YDR496C	YOR272W	7.7996
YLR448W	YML073C	7.7982
YHR139C	YNL232W	7.7981
YLR153C	YPL028W	7.798
YKL172W	YLR002C	7.7977
YPR137W	YPR144C	7.7968
YDR449C	YGR128C	7.7966
YLR105C	YPL083C	7.7961
YFL005W	YML001W	7.7957
YER174C	YKR038C	7.7957
YDR238C	YPL031C	7.7938
YDL031W	YKL172W	7.793
YDL002C	YDL141W	7.7928
YDR301W	YMR061W	7.7926
YCL059C	YOL010W	7.7895
YCL024W	YJL082W	7.7884
YDL229W	YHR041C	7.7877
YNL178W	YPL126W	7.7855
YDL043C	YER172C	7.7844
YBL004W	YLR197W	7.7843
YBR017C	YJR077C	7.7839
YMR028W	YOR014W	7.7823
YHR075C	YMR028W	7.7823
YGR161C	YMR028W	7.7823
YGR275W	YPL129W	7.7808
YFL033C	YLR357W	7.7805
YGL116W	YHR009C	7.7782
YDL035C	YER174C	7.7782
YLR298C	YPL178W	7.7781
YHR143W-A	YOR210W	7.7771
YGL207W	YMR172W	7.7769
YDL014W	YPL217C	7.7731
YHR069C	YHR139C	7.7715
YLR199C	YML092C	7.7712
YNL025C	YNR010W	7.7688
YGL131C	YLR413W	7.7688
YDR533C	YMR322C	7.7682
YDL130W-A	YDL181W	7.7682
YCR005C	YOR323C	7.7682
YAL012W	YGL113W	7.7682
YDL074C	YOR365C	7.7681
YKR060W	YPR144C	7.768
YBR017C	YEL022W	7.7665
YER103W	YIL053W	7.7657
YER022W	YML007W	7.7655
YJL014W	YOR281C	7.7643
YLR039C	YMR146C	7.7642
YHR059W	YNL137C	7.7628
YGR002C	YOR374W	7.76
YBR055C	YBR152W	7.759
YDL098C	YML049C	7.7589
YDR012W	YNL132W	7.7558
YBL002W	YGL241W	7.754
YDR448W	YHR041C	7.7534
YLR071C	YNL025C	7.7518
YBL017C	YDR343C	7.7518
YDL097C	YER012W	7.7505
YIL070C	YLL022C	7.7499
YCL028W	YMR275C	7.7499
YHR179W	YMR201C	7.7484
YGL174W	YJL203W	7.7475
YGR091W	YLR424W	7.7472
YBR146W	YDL045W-A	7.7472
YNL251C	YPL178W	7.7471
YGR218W	YJR077C	7.7468
YKR023W	YNL330C	7.7437
YGL066W	YGR274C	7.7434
YCR063W	YHR165C	7.7434
YIL070C	YPL013C	7.7432
YNL207W	YOR145C	7.7417
YER133W	YLR258W	7.7415
YDR101C	YLR276C	7.7412
YGR145W	YLR186W	7.74
YFR037C	YOR304W	7.7397
YDR060W	YOL041C	7.7396
YGL019W	YKL088W	7.7385
YAR014C	YER155C	7.7385
YDL030W	YPR101W	7.7366
YLR199C	YOL038W	7.7352
YFL018C	YMR211W	7.7334
YJL109C	YMR229C	7.7318
YKL009W	YNL035C	7.731
YER021W	YGL011C	7.7283
YGR204W	YKL210W	7.7273
YLR147C	YPL151C	7.7272
YLR448W	YNL069C	7.7267
YHR196W	YPR144C	7.7252
YDR436W	YMR311C	7.7237
YNL014W	YOR262W	7.7235
YMR125W	YPL190C	7.7235
YGL212W	YHR084W	7.7235
YDR480W	YGL212W	7.7235
YBR001C	YNL267W	7.7235
YKL007W	YMR109W	7.7231
YDR211W	YMR309C	7.721
YIL152W	YJR092W	7.7194
YDR515W	YPR188C	7.7194
YDR300C	YHR164C	7.7194
YDL097C	YNL049C	7.7194
YDL004W	YDL160C	7.7194
YDL182W	YHR079C	7.7192
YBR122C	YML025C	7.7187
YKL172W	YPL211W	7.7168
YGR128C	YMR128W	7.7163
YAL035W	YDR091C	7.7155
YER162C	YML011C	7.7136
YDR194C	YNL230C	7.7123
YGL121C	YJL124C	7.7098
YDR054C	YGL143C	7.7086
YGR013W	YHR165C	7.7066
YMR072W	YPL082C	7.7059
YER070W	YJR053W	7.7048
YLR449W	YNL002C	7.7039
YJL002C	YMR149W	7.7037
YGL022W	YMR149W	7.7037
YLL045C	YMR049C	7.7035
YHR059W	YJR113C	7.7032
YDR145W	YML098W	7.7013
YER173W	YJR068W	7.7001
YDR488C	YOR172W	7.7
YDR233C	YLL022C	7.7
YBL064C	YOR172W	7.7
YKL206C	YPL051W	7.6999
YEL018W	YNL330C	7.6992
YBL072C	YNL132W	7.6989
YHR139C	YOR001W	7.6987
YBL006C	YDR303C	7.6944
YOR116C	YPL082C	7.6943
YHL004W	YHR059W	7.6939
YLR384C	YPL204W	7.6932
YML046W	YMR288W	7.6927
YDL160C	YPR178W	7.6916
YGL009C	YPL028W	7.6912
YGL009C	YGR171C	7.6912
YHR059W	YPL013C	7.6901
YJR007W	YOR361C	7.6896
YBR146W	YDR494W	7.6882
YCR077C	YJR022W	7.6877
YKL143W	YLR186W	7.6874
YDL126C	YKL204W	7.6872
YBR009C	YMR308C	7.6858
YHR029C	YNL333W	7.6843
YDL189W	YPR191W	7.6842
YBL045C	YDL189W	7.6842
YDR485C	YGR002C	7.6797
YBR087W	YLR058C	7.6793
YDR448W	YMR227C	7.6787
YDL195W	YPL085W	7.6783
YAL038W	YKL104C	7.6773
YHR148W	YMR128W	7.6772
YEL018W	YPL139C	7.6772
YBR055C	YOR159C	7.6764
YGL082W	YMR246W	7.6747
YAL033W	YBR167C	7.6729
YDR312W	YPR143W	7.6721
YKL088W	YOR039W	7.6717
YHL034C	YNL255C	7.6688
YGL206C	YNL243W	7.6679
YGR090W	YLL045C	7.6663
YFL005W	YKR014C	7.6657
YPL217C	YPR137W	7.6652
YDR041W	YHR059W	7.665
YCR063W	YHR156C	7.6639
YGL115W	YOR142W	7.6638
YGL115W	YGR244C	7.6638
YJL069C	YPL126W	7.6632
YMR223W	YMR236W	7.6627
YAL032C	YML049C	7.6618
YGR090W	YPR112C	7.6617
YIL021W	YJR017C	7.6589
YDR050C	YDR385W	7.6559
YCR072C	YGR103W	7.6551
YGR286C	YKR064W	7.6549
YGL026C	YLR006C	7.6549
YKR081C	YMR229C	7.6547
YKR001C	YKR008W	7.6541
YDR073W	YPL129W	7.6533
YDL075W	YOL127W	7.6528
YBL074C	YGL128C	7.6495
YDL047W	YPR040W	7.6491
YDL148C	YGR090W	7.6469
YCL043C	YIR001C	7.646
YGL078C	YML127W	7.6362
YNL078W	YPL086C	7.6355
YLR384C	YNL078W	7.6355
YLR247C	YOR028C	7.6355
YJR127C	YPL086C	7.6355
YJR127C	YNL078W	7.6355
YJR127C	YLR384C	7.6355
YJL085W	YOR028C	7.6355
YJL085W	YLR247C	7.6355
YHL039W	YKR082W	7.6355
YGR200C	YNL078W	7.6355
YGR200C	YJR127C	7.6355
YGL150C	YNL068C	7.6355
YFR016C	YKL007W	7.6355
YFR016C	YIL034C	7.6355
YBR139W	YNL141W	7.6355
YGR132C	YJR075W	7.6338
YBR130C	YKL007W	7.6336
YDR296W	YOR150W	7.6312
YDR116C	YOR150W	7.6312
YCR077C	YGR091W	7.6312
YBR282W	YNL005C	7.6292
YBL074C	YGR074W	7.6288
YAL016W	YHR075C	7.6284
Q0055	YMR237W	7.6284
YDL083C	YGL103W	7.6273
YER164W	YGL207W	7.6223
YDR299W	YMR290C	7.621
YGL174W	YML049C	7.6203
YLR287C	YML029W	7.6198
YLR178C	YMR297W	7.6192
YDR432W	YNL004W	7.6184
YJL109C	YLR186W	7.6177
YGL011C	YLR199C	7.6141
YGL245W	YHR064C	7.6134
YGR218W	YNL064C	7.6125
YNL155W	YOR201C	7.6112
YBR197C	YLR423C	7.6112
YGL036W	YKR031C	7.6102
YDR430C	YOR232W	7.6102
YDR331W	YDR434W	7.6102
YGR252W	YNL236W	7.6091
YBR264C	YER031C	7.6048
YDR303C	YJL137C	7.6034
YJR045C	YOL070C	7.6031
YJR045C	YNL078W	7.6031
YDL031W	YKL009W	7.6028
YOR206W	YPL043W	7.6026
YJL076W	YNL113W	7.6017
YLR314C	YPL167C	7.5993
YDR517W	YPL085W	7.5991
YFR028C	YPR187W	7.5969
YGR090W	YMR229C	7.5957
YLR182W	YOR083W	7.5943
YDR137W	YNL044W	7.5943
YGR103W	YHR088W	7.5928
YER112W	YKL173W	7.5928
YBL006C	YIL126W	7.592
YAL003W	YGR285C	7.5904
YDR337W	YHR059W	7.5875
YCR052W	YFL033C	7.5863
YDL155W	YEL023C	7.5859
YDL004W	YKR022C	7.5859
YDL007W	YMR191W	7.5855
YHR052W	YOL041C	7.5847
YIL109C	YLR208W	7.5817
YDR224C	YOR304W	7.581
YDL087C	YKL173W	7.5802
YHR114W	YNR065C	7.5796
YHR114W	YLR081W	7.5796
YHR114W	YIR012W	7.5796
YBL007C	YNR065C	7.5796
YBL007C	YLR081W	7.5796
YBL007C	YIR012W	7.5796
YBR173C	YMR314W	7.5792
YJR145C	YOL115W	7.5785
YML032C	YOL090W	7.5781
YLR064W	YMR123W	7.5776
YDR280W	YLR398C	7.5776
YDL055C	YLR243W	7.5776
YCR086W	YDR439W	7.5776
YAL005C	YNR050C	7.5774
YGR285C	YLR249W	7.5772
YNL004W	YPR108W	7.5758
YHR156C	YHR165C	7.5751
YDR170C	YLR263W	7.5747
YDR099W	YPR030W	7.5747
YDR050C	YKL060C	7.5747
YCL054W	YPL211W	7.5744
YCR046C	YOR150W	7.5734
YDR153C	YLR398C	7.5726
YER025W	YER102W	7.5724
YDR228C	YLR115W	7.5724
YDL030W	YDL209C	7.5698
YER050C	YNL306W	7.5689
YCR072C	YLR106C	7.5678
YJL060W	YLR314C	7.5665
YDR006C	YPL154C	7.5665
YDR028C	YLR258W	7.5646
YOR076C	YPR189W	7.5635
YPL213W	YPR182W	7.5606
YJR084W	YMR240C	7.5593
YLR449W	YOL077C	7.5586
YLR424W	YMR240C	7.5552
YDR129C	YGL106W	7.5541
YKR001C	YMR033W	7.5528
YBR247C	YJR002W	7.552
YBL004W	YLR222C	7.5519
YDR050C	YMR303C	7.5518
YBR181C	YOL127W	7.5508
YLL048C	YNL238W	7.5496
YPL213W	YPR082C	7.5495
YDR293C	YJR053W	7.5484
YKL007W	YKL130C	7.548
YJR068W	YLR413W	7.5476
YKR014C	YNL093W	7.5475
YNL230C	YOR304W	7.5451
YGL133W	YNL230C	7.5451
YER179W	YMR224C	7.5451
YCR053W	YHR183W	7.5451
YGR095C	YPR190C	7.545
YER172C	YNR011C	7.5446
YGL019W	YOL054W	7.5427
YKL110C	YMR312W	7.5413
YGR030C	YNL282W	7.5413
YOR101W	YOR335C	7.5412
YJL014W	YJL111W	7.5412
YCR063W	YJR050W	7.5411
YDL131W	YOR063W	7.5409
YMR093W	YOR063W	7.5408
YGL174W	YMR288W	7.5408
YEL031W	YJL001W	7.5373
YGR074W	YML049C	7.5365
YDR087C	YOR206W	7.5357
YMR024W	YNL177C	7.5348
YLR154C	YPL049C	7.5348
YJL173C	YOL090W	7.5334
YMR209C	YMR243C	7.533
YDL215C	YKR066C	7.533
YKR002W	YOR179C	7.5274
YMR290C	YNL110C	7.5242
YMR158W	YNR037C	7.523
YER127W	YPL198W	7.522
YLR299W	YOR168W	7.5214
YLR299W	YNL019C	7.5214
YLR299W	YNL015W	7.5214
YMR049C	YPL043W	7.518
YOR100C	YPR018W	7.5177
YOR100C	YOR308C	7.5177
YBR195C	YOR100C	7.5177
YJL063C	YOR150W	7.5168
YGR104C	YML007W	7.5147
YEL054C	YER102W	7.5144
YNL182C	YPR016C	7.5129
YGL103W	YGR034W	7.5096
YML014W	YNR050C	7.5084
YBL076C	YHR019C	7.5082
YAL029C	YLR429W	7.5064
YBR267W	YNL227C	7.5059
YOR093C	YOR194C	7.5058
YKL058W	YOR093C	7.5058
YDL145C	YGL021W	7.5057
YMR261C	YNL064C	7.5056
YOL086C	YPL126W	7.5053
YKL009W	YPL211W	7.5038
YER012W	YNR003C	7.5012
YIL126W	YJL137C	7.501
YJL109C	YOR145C	7.5004
YDL045W-A	YPL118W	7.5003
YNL004W	YOR261C	7.5002
YJL076W	YOR341W	7.4966
YGR162W	YKR059W	7.4934
YDR328C	YOL088C	7.4934
YKR031C	YPR006C	7.4925
YKR031C	YPL183C	7.4925
YDR430C	YLR203C	7.4925
YDR347W	YDR494W	7.4919
YOR207C	YOR341W	7.491
YGL207W	YOL006C	7.4883
YFL033C	YPR034W	7.4869
YER146W	YHR165C	7.4857
YDL188C	YHR075C	7.4856
YER092W	YNL059C	7.4844
YHR147C	YLR439W	7.483
YLR186W	YPL217C	7.4828
YGR202C	YMR125W	7.4812
YKR022C	YLR117C	7.4797
YAL029C	YIL034C	7.4792
YFR010W	YML057W	7.4785
YBR257W	YGR030C	7.4767
YDR473C	YFL017W-A	7.4743
YBR193C	YNL025C	7.4724
YDR359C	YJR082C	7.4707
YIL035C	YNL097C	7.4699
YDL045W-A	YDR347W	7.4695
YLR175W	YMR229C	7.4679
YDL131W	YDR012W	7.4678
YIL035C	YOL004W	7.4676
YDR462W	YKR006C	7.4666
YDR462W	YKL167C	7.4666
YDR496C	YMR128W	7.4641
YBR142W	YOR206W	7.4615
YDL031W	YMR229C	7.4614
YGL212W	YPL049C	7.4612
YGL112C	YNL236W	7.4611
YEL022W	YER110C	7.4601
YDR494W	YOR158W	7.4582
YCL011C	YDR381W	7.4566
YHR069C	YJL011C	7.4552
YGR145W	YNR054C	7.4542
YLR029C	YMR290C	7.4525
YBR152W	YPR178W	7.4514
YHR088W	YOR206W	7.4504
YHR143W-A	YOR341W	7.4493
YGR220C	YNL005C	7.4487
YMR076C	YNL206C	7.4472
YBR152W	YMR004W	7.4462
YGL253W	YOL097C	7.4461
YKR090W	YLR143W	7.446
YEL052W	YIL074C	7.446
YDL165W	YNL315C	7.4459
YMR309C	YOR260W	7.4457
YER006W	YNL182C	7.4457
YDR507C	YPL022W	7.4447
YER125W	YGR240C	7.4442
YGL173C	YKL023W	7.4429
YDR494W	YGR215W	7.4416
YKL012W	YMR240C	7.44
YHR041C	YPL106C	7.4392
YIL129C	YML081W	7.4382
YBL017C	YML123C	7.4378
YFR050C	YNR003C	7.4367
YDR381W	YFR001W	7.436
YDL014W	YLR129W	7.4337
YNL025C	YPR070W	7.4336
YDL132W	YML088W	7.4333
YBR280C	YDL132W	7.4333
YCL017C	YKR064W	7.4318
YBL072C	YBR189W	7.4273
YKL143W	YPL204W	7.427
YDR494W	YNL137C	7.4251
YJR059W	YOR257W	7.4244
YDL155W	YPL014W	7.4235
YDL155W	YLR144C	7.4235
YDL155W	YKR091W	7.4235
YGL120C	YGR278W	7.4227
YBL030C	YKR046C	7.4218
YBR142W	YMR290C	7.4195
YDR432W	YMR125W	7.4186
YKL166C	YNL227C	7.4179
YHR165C	YLL002W	7.4172
YER074W	YFR031C-A	7.4168
YLL036C	YPR178W	7.4162
YEL002C	YPL050C	7.4124
YEL002C	YML115C	7.4124
YGR280C	YMR302C	7.4123
YGL244W	YHR009C	7.4123
YDR243C	YGL192W	7.4123
YGR275W	YHR056C	7.4121
YDR145W	YML114C	7.411
YKL192C	YNL185C	7.4106
YJL039C	YLR243W	7.4106
YGR031W	YJR089W	7.4106
YGL112C	YML098W	7.4077
YDR188W	YNL212W	7.4074
YDR052C	YFR004W	7.4042
YDR324C	YLL011W	7.4039
YGR136W	YLR144C	7.4036
YDR330W	YMR067C	7.4036
YAL012W	YMR235C	7.4015
YLR071C	YML007W	7.4013
YDR190C	YPL082C	7.401
YDL040C	YGR090W	7.4005
YBL090W	YPL013C	7.399
YBL090W	YKL003C	7.399
YIL021W	YPR133C	7.398
YDL045W-A	YGL129C	7.3979
YEL055C	YMR121C	7.3972
YIL135C	YOR043W	7.3967
YDR369C	YER179W	7.3967
Q0055	YLR330W	7.3967
YKL009W	YOL123W	7.3962
YDL098C	YNL147W	7.3959
YKL143W	YPL266W	7.3943
YML025C	YOR150W	7.3935
YDR021W	YGR162W	7.3922
YDL047W	YGR090W	7.3912
YER012W	YLR199C	7.3896
YIL094C	YPL249C	7.3875
YDR264C	YHR005C	7.3871
YBR107C	YDR383C	7.3871
YMR203W	YPR024W	7.387
YDR436W	YKL193C	7.3866
YGR091W	YPR182W	7.3847
YHR089C	YMR310C	7.3844
YGR074W	YMR240C	7.3812
YER050C	YGR084C	7.3809
YCR076C	YER094C	7.3794
YBR011C	YLR043C	7.3782
YBR247C	YGR145W	7.3758
YBR009C	YOL012C	7.3753
YDR054C	YDR328C	7.3737
YGR103W	YPL211W	7.3725
YDR328C	YFL009W	7.3711
YDL148C	YGR128C	7.3707
YLL011W	YLR222C	7.3693
YLL011W	YLR129W	7.3693
YOR210W	YOR335C	7.3685
YDR462W	YMR193W	7.3682
YDR129C	YMR237W	7.3682
YCL054W	YER006W	7.3681
YDR101C	YLR397C	7.3675
YEL018W	YLR399C	7.3664
YGL049C	YPL190C	7.3663
YHR074W	YNL278W	7.3636
YDL195W	YLR429W	7.3636
YBR059C	YPR189W	7.3636
YBR059C	YIL095W	7.3636
YNL215W	YPL235W	7.3629
YDR127W	YGR009C	7.3628
YDL074C	YMR100W	7.3628
YGL210W	YOR089C	7.3624
YJL069C	YLR197W	7.3623
YDR494W	YKL155C	7.3606
YDR129C	YGR080W	7.3593
YOL041C	YPL012W	7.3592
YOR298W	YOR386W	7.359
YLR080W	YLR250W	7.359
YGR030C	YHR062C	7.359
YGR028W	YJL123C	7.359
YFL048C	YLR250W	7.359
YFL048C	YLR080W	7.359
YBR177C	YGL242C	7.359
YDR198C	YJR009C	7.3589
YMR304W	YPL247C	7.3575
YLR270W	YMR304W	7.3575
YDR224C	YLR357W	7.3569
YKL130C	YMR109W	7.3561
YDL014W	YJL109C	7.355
YGL252C	YNL076W	7.3538
YCL059C	YML056C	7.3534
YFR053C	YLR249W	7.3533
YDR388W	YLR337C	7.3531
YBR055C	YDR416W	7.3498
YCR063W	YLR275W	7.348
YGL003C	YKR048C	7.3474
YDL045W-A	YNL137C	7.3474
YAR007C	YDR499W	7.3448
YJL001W	YLR199C	7.3445
YDR322W	YHR147C	7.3412
YDR299W	YJL033W	7.341
YNR003C	YOR210W	7.3384
YIL070C	YOR035C	7.3377
YER125W	YOR061W	7.3376
YDR473C	YLL036C	7.337
YDR228C	YLR277C	7.3368
YMR167W	YOR386W	7.3365
YGR017W	YLR449W	7.3362
YJL001W	YML130C	7.3342
YGR284C	YJL001W	7.3342
YGR002C	YOL052C	7.334
YDR198C	YFL024C	7.3323
YDR280W	YPR189W	7.3313
YGR159C	YLR410W	7.3308
YDR324C	YOR145C	7.3301
YDR404C	YPR110C	7.33
YBL037W	YJR005W	7.33
YDR473C	YMR240C	7.3299
YDR060W	YPL043W	7.3286
YCR014C	YLR421C	7.3255
YGR232W	YPL002C	7.3226
YMR033W	YMR072W	7.3224
YDL147W	YNL004W	7.3204
YGR156W	YPL137C	7.3202
YDL060W	YML091C	7.3202
YJL011C	YOR001W	7.3188
YBR065C	YJR050W	7.3188
YIR003W	YJL020C	7.3182
YER083C	YKL062W	7.317
YDL100C	YKL062W	7.317
YGR126W	YLL029W	7.3148
YJL122W	YLR397C	7.311
YGR278W	YLR424W	7.3107
YGL245W	YKL104C	7.3098
YOL115W	YOR204W	7.3077
YGL122C	YKL009W	7.3075
YLR109W	YNL250W	7.3074
YLR190W	YPL031C	7.3045
YIL070C	YOL054W	7.3027
YKL172W	YOR272W	7.3013
YHR197W	YMR049C	7.3008
YGR005C	YJL176C	7.2984
YMR304W	YPR163C	7.2983
YIL007C	YKR022C	7.2983
YCL047C	YKR022C	7.2983
YDR166C	YGR037C	7.2982
YBL004W	YOL010W	7.2968
YER172C	YGR075C	7.2953
YER126C	YGR103W	7.2951
YMR125W	YOR361C	7.2942
YGR078C	YJL050W	7.2933
YER112W	YGL173C	7.2931
YBR170C	YBR273C	7.2921
YDR517W	YOR089C	7.2917
YDL031W	YGR090W	7.2893
YFL007W	YML092C	7.2873
YGR283C	YNL009W	7.2859
YDL216C	YDR023W	7.2859
YBR234C	YOR269W	7.2858
YDR175C	YKL003C	7.2855
YNR050C	YOL124C	7.2854
YNR046W	YNR050C	7.2854
YDR140W	YNR050C	7.2854
YCR047C	YMR128W	7.2846
YDR337W	YDR494W	7.2828
YDR091C	YOR361C	7.2823
YDR129C	YKR027W	7.2822
YAR073W	YLR432W	7.2811
YAR007C	YDL156W	7.2811
YLR275W	YPL064C	7.2809
YDL045W-A	YHL004W	7.2798
YMR196W	YPL154C	7.2789
YDL113C	YJL036W	7.2789
YDL113C	YDR425W	7.2789
YGL173C	YOL139C	7.2786
YDR060W	YOR063W	7.278
YBR118W	YGR254W	7.2774
YMR093W	YNL075W	7.2754
YDR449C	YNL075W	7.2754
YLR052W	YNL215W	7.2738
YDL215C	YDR288W	7.2736
YLR347C	YPL153C	7.2733
YBR055C	YLR424W	7.2727
YJL080C	YLR249W	7.2715
YGR091W	YML046W	7.2714
YML063W	YPR025C	7.2712
YHR086W	YHR165C	7.27
YBR281C	YKL010C	7.2686
YDR388W	YOR181W	7.2681
YCL059C	YGR145W	7.2675
YBL056W	YBR125C	7.2675
YDL185W	YLL019C	7.2663
YDR381W	YNL004W	7.2662
YFR031C	YKL173W	7.2655
YPL090C	YPR056W	7.2646
YDL111C	YPR189W	7.2636
YML063W	YOR312C	7.2629
YGR145W	YKR060W	7.2606
YGR132C	YMR089C	7.2604
YDR237W	YLR312W-A	7.2594
YFR052W	YPL002C	7.258
YHR156C	YMR213W	7.2578
YJL052W	YJR009C	7.2538
YDL014W	YPL126W	7.2536
YBL018C	YGR030C	7.2536
YAL033W	YGR030C	7.2536
YLR357W	YMR125W	7.2524
YDL045W-A	YDR041W	7.2515
YLR175W	YNL009W	7.2508
YDR060W	YHR088W	7.2507
YGL207W	YIL070C	7.2499
YDR325W	YLR272C	7.2495
YDR254W	YGR111W	7.2495
YKR081C	YNL182C	7.2489
YLR362W	YPL115C	7.2459
YMR070W	YNL027W	7.2436
YDR372C	YOL002C	7.2436
YCR073W-A	YNR034W	7.2436
YAL020C	YHR103W	7.2436
YER136W	YGL210W	7.2435
YJL011C	YOR210W	7.2432
YEL031W	YGR135W	7.2414
YDR359C	YOR046C	7.2404
YLR186W	YPR137W	7.2402
YMR240C	YPL151C	7.2393
YHR197W	YLL008W	7.2386
YKL009W	YNL061W	7.2378
YHR179W	YLL060C	7.2377
YEL031W	YOL038W	7.2373
YGR002C	YNL189W	7.2364
YJL008C	YKL145W	7.236
YDR364C	YPL213W	7.2347
YLR075W	YOR312C	7.2336
YBL041W	YEL031W	7.2333
YDL073W	YJL179W	7.2318
YBL036C	YGR185C	7.2318
YDL182W	YNL178W	7.2313
YBL045C	YLL036C	7.2304
YHR046C	YPR015C	7.2301
YFL034W	YOL082W	7.2301
YDR395W	YPR015C	7.2301
YDR233C	YEL056W	7.2301
YAL019W	YOL045W	7.23
YDR496C	YHR052W	7.227
YNL308C	YOL041C	7.226
YDL136W	YJL177W	7.2252
YDR448W	YPL047W	7.2251
YMR181C	YOR144C	7.2229
YHL029C	YNL056W	7.2229
YCR095C	YNL056W	7.2229
YAL041W	YNL007C	7.2229
YDR099W	YNR047W	7.2225
YNL031C	YNL206C	7.2208
YDR240C	YPR178W	7.2207
YLR312W-A	YMR098C	7.2196
YGL145W	YOL086C	7.2193
YGR090W	YML063W	7.2165
YMR125W	YOL139C	7.2161
YBR009C	YMR072W	7.2159
YJL042W	YMR311C	7.2157
YCR057C	YPL266W	7.2154
YMR128W	YNL075W	7.2148
YBR247C	YPR144C	7.2145
YGL174W	YMR240C	7.2143
YKL143W	YML091C	7.2142
YOR179C	YPR107C	7.2141
YHR084W	YLR154C	7.214
YDR237W	YNL177C	7.2127
YAL032C	YBR065C	7.2113
YER104W	YHR164C	7.2087
YER088C	YNL091W	7.2087
YDR314C	YER021W	7.2069
YCR009C	YNL106C	7.2057
YLL045C	YOR272W	7.2053
YDR012W	YPL198W	7.2049
YLL002W	YMR020W	7.203
YKL007W	YOL065C	7.2027
YGL087C	YLR059C	7.2003
YDR171W	YML085C	7.1995
YLR424W	YMR125W	7.1993
YDR108W	YLR421C	7.1991
YGR158C	YPR189W	7.1975
YCR035C	YHR139C	7.1975
YMR308C	YOL108C	7.1972
YDR025W	YPL081W	7.1969
YJR084W	YLR424W	7.1961
YKR026C	YMR309C	7.1953
YLL034C	YNL061W	7.194
YCR088W	YNL208W	7.1938
YDR494W	YPL118W	7.1933
YKL186C	YPL169C	7.193
YGR090W	YGR270W	7.1912
YCR031C	YDR450W	7.1908
YLR276C	YOR378W	7.1893
YJL156C	YLR313C	7.1893
YFL053W	YGR041W	7.1893
YDR316W	YGR041W	7.1893
YDR316W	YFL053W	7.1893
YDL215C	YLR320W	7.1893
YDL164C	YOR378W	7.1893
YDL164C	YLR276C	7.1893
YAL049C	YDL025C	7.1893
YFR004W	YNL004W	7.1885
YBR081C	YMR005W	7.1883
YDR224C	YOR116C	7.1854
YGL049C	YNL251C	7.1846
YAL038W	YIL128W	7.1838
YHR165C	YML046W	7.1837
YKR014C	YLR362W	7.1821
YGR255C	YPL059W	7.1806
YFR021W	YLR163C	7.1806
YFR021W	YHR024C	7.1806
YDL122W	YNL085W	7.1806
YBR167C	YNL221C	7.1806
YBR049C	YCR047C	7.1806
YJL137C	YLR258W	7.1805
YGR155W	YML085C	7.1789
YDR359C	YLR347C	7.1781
YML091C	YNL207W	7.1763
YDR341C	YGR204W	7.1762
YLR355C	YOL086C	7.1753
YLR430W	YOL059W	7.1752
YLR368W	YLR430W	7.1752
YHL007C	YLR430W	7.1752
YDL022W	YLR430W	7.1752
YBL052C	YOR244W	7.175
YFR051C	YHR066W	7.1744
YPL059W	YPR023C	7.1737
YJL076W	YPR110C	7.1736
YCR035C	YDL150W	7.1713
YLR276C	YMR290C	7.1708
YHR196W	YLR186W	7.1696
YFR043C	YLR170C	7.1695
YER049W	YOR051C	7.1695
YER027C	YER129W	7.1695
YDR033W	YJL098W	7.1695
YAR002W	YGR067C	7.1695
YKL210W	YNR016C	7.1694
YLR129W	YNL132W	7.169
YKR060W	YNL132W	7.1667
YNL232W	YPR189W	7.165
YBR081C	YHL047C	7.1649
YKR081C	YLL008W	7.1646
YMR197C	YOL018C	7.1641
YGL095C	YMR197C	7.1641
YDR468C	YMR197C	7.1641
YHR193C	YNL112W	7.1635
YER110C	YGR218W	7.1634
YML046W	YMR240C	7.162
YDR188W	YFR009W	7.1613
YBR264C	YOR089C	7.1595
YAR007C	YIR002C	7.1595
YAR007C	YDR097C	7.1595
YMR237W	YNL045W	7.1586
YGR075C	YMR004W	7.1586
YDR381W	YLR044C	7.1586
YHR024C	YLR172C	7.1584
YDR422C	YGL208W	7.1584
YBR039W	YDR168W	7.1584
YGL151W	YNL025C	7.1583
YJR002W	YOR310C	7.1575
YIL133C	YNL069C	7.1565
YHR013C	YHR203C	7.1564
YBR143C	YPL237W	7.1534
YOR159C	YPR057W	7.1531
YOL040C	YOL127W	7.1531
YDR060W	YNL132W	7.1521
YBL071W-A	YOL109W	7.1514
YAL029C	YKL007W	7.1514
YDL006W	YLR148W	7.15
YIL034C	YLR429W	7.1491
YGR128C	YNL132W	7.1488
YBL037W	YJL084C	7.1478
YBR119W	YPR182W	7.1474
YIR002C	YJR144W	7.1468
YER126C	YPR016C	7.1464
YDR207C	YOL004W	7.1462
YDL140C	YMR275C	7.1462
YBR289W	YPL129W	7.146
YDR127W	YGR240C	7.1459
YCR088W	YGL106W	7.1452
YLL034C	YPL093W	7.1451
YDL014W	YDR087C	7.1442
YDR174W	YDR224C	7.1438
YBR173C	YGR135W	7.1374
YBL093C	YNL236W	7.1371
YKL014C	YLR175W	7.1369
YJL020C	YJL123C	7.1359
YIL079C	YJR076C	7.1359
YGR028W	YJL020C	7.1359
YDR122W	YHR158C	7.1359
YAR019C	YKL010C	7.135
YDR041W	YKL003C	7.1342
YDL060W	YGR081C	7.1337
YGL111W	YPL043W	7.133
YDR299W	YPR144C	7.1329
YDR427W	YER012W	7.1321
YGL245W	YKL081W	7.1317
YFL002C	YKL172W	7.1302
YKR060W	YLR197W	7.1294
YDR238C	YHR066W	7.1285
YGR075C	YHR165C	7.1284
YHL015W	YOR173W	7.1272
YHL015W	YLR270W	7.1272
YDL148C	YFL016C	7.1265
YBL036C	YDR453C	7.1265
YFR051C	YKR067W	7.1238
YER029C	YGL128C	7.1233
YDR328C	YML088W	7.1229
YKR008W	YPL082C	7.1228
YFR051C	YPR143W	7.1214
YDR359C	YOL004W	7.1194
YDR036C	YHR059W	7.1182
YBL004W	YPL012W	7.1164
YLR360W	YNL223W	7.116
YGL058W	YMR100W	7.116
YGL058W	YML101C	7.116
YDR523C	YGL062W	7.116
YDR017C	YHR102W	7.116
YER122C	YPL010W	7.1159
YKL012W	YOR308C	7.1152
YER094C	YLR199C	7.114
YGR030C	YNL221C	7.1139
YKL078W	YLR055C	7.1136
YDR233C	YPL001W	7.1123
YBL016W	YNL053W	7.1123
YGL133W	YLR357W	7.1119
YAR014C	YHR158C	7.111
YHR148W	YNL132W	7.1105
YML092C	YML130C	7.1085
YGR284C	YML092C	7.1085
YCR002C	YIL079C	7.1085
YFL017W-A	YPR178W	7.1083
YBR247C	YPL012W	7.1081
YGR054W	YOR253W	7.1075
YDL031W	YOR253W	7.1075
YJR063W	YOR207C	7.1067
YBR055C	YMR268C	7.1066
YDL148C	YKR060W	7.1058
YGR161C	YMR273C	7.1057
YKR006C	YMR024W	7.1044
YKL167C	YMR024W	7.1044
YDL014W	YLR186W	7.1035
YGR245C	YLR106C	7.1032
YGL142C	YML012W	7.103
YFL007W	YKL168C	7.103
YER050C	YGL129C	7.1029
YDL031W	YDR496C	7.1029
YAL005C	YLR354C	7.1023
YBR282W	YGR220C	7.1006
YER003C	YGR250C	7.1005
YDR128W	YOR093C	7.1005
YCL008C	YGL045W	7.1005
YBR247C	YNL178W	7.0992
YGL208W	YIL095W	7.0978
YGL245W	YPR010C	7.0974
YBR152W	YER029C	7.0971
YKR081C	YOL041C	7.0965
YAL032C	YGR278W	7.0961
YKL009W	YNL182C	7.0959
YGL173C	YNL147W	7.0959
YPL204W	YPR116W	7.0954
YLR256W	YOR220W	7.0954
YLR091W	YPR116W	7.0954
YLR091W	YPL204W	7.0954
YLL033W	YPR116W	7.0954
YLL033W	YPL204W	7.0954
YLL033W	YLR091W	7.0954
YGL181W	YOR220W	7.0954
YGL181W	YLR256W	7.0954
YFR008W	YKL144C	7.0954
YDR179W-A	YGL201C	7.0954
YDR065W	YPR116W	7.0954
YDR065W	YPL204W	7.0954
YDR065W	YLR091W	7.0954
YDR065W	YLL033W	7.0954
YDR011W	YGL201C	7.0954
YDR011W	YDR179W-A	7.0954
YBR059C	YOR220W	7.0954
YBR059C	YLR256W	7.0954
YBR059C	YGL181W	7.0954
YJR084W	YKL012W	7.094
YKL007W	YKR062W	7.0934
YGR090W	YOR063W	7.0919
YJR101W	YKL003C	7.0909
YPL012W	YPL266W	7.0908
YML074C	YPL198W	7.0902
YCR052W	YGL078C	7.0896
YDR005C	YPR190C	7.0895
YGR161C	YMR314W	7.0893
YDR075W	YIL153W	7.0871
YBL058W	YKL213C	7.0871
YDL076C	YMR075W	7.0825
YBR072W	YJL066C	7.0819
YJL177W	YNL096C	7.0813
YER171W	YIL053W	7.0789
YER012W	YOR259C	7.077
YLL034C	YNL002C	7.0762
YHR074W	YOR154W	7.076
YBR059C	YLR398C	7.076
YBR059C	YGL213C	7.076
YDL137W	YHR188C	7.0759
YDR238C	YPR143W	7.0755
YLR433C	YNL104C	7.0752
YKR022C	YML106W	7.0752
YER111C	YPL160W	7.0752
YDL031W	YLR449W	7.075
YFR050C	YHL030W	7.0737
YBR010W	YDR224C	7.0731
YDR171W	YNL085W	7.0706
YBR154C	YHR143W-A	7.0702
YFR051C	YPL088W	7.0698
YGL013C	YJL176C	7.0671
YLR347C	YOR151C	7.0639
YGL112C	YML007W	7.0632
YJL081C	YNL330C	7.0616
YER021W	YIL107C	7.0612
YOR244W	YPR031W	7.0611
YGR218W	YMR196W	7.0603
YGR159C	YMR237W	7.0593
YAL027W	YLR430W	7.059
YBR065C	YKL173W	7.0581
YIL079C	YLR314C	7.0559
YDR202C	YDR328C	7.0559
YLR383W	YOL034W	7.0554
YML069W	YOL006C	7.0545
YDR496C	YNL002C	7.0539
YDR190C	YKR001C	7.0535
YER126C	YLR074C	7.0533
YBR142W	YKL172W	7.0514
YJL139C	YKR061W	7.0508
YGL180W	YPR185W	7.0508
YDR498C	YLR268W	7.0508
YBR207W	YFL041W	7.0508
YDL115C	YGR063C	7.0485
YIL018W	YOR312C	7.0467
YBR221C	YHR121W	7.0444
YKR060W	YOR145C	7.0443
YDR036C	YMR158W	7.0428
YNL307C	YPL048W	7.0414
YBR170C	YNL155W	7.0408
YOR151C	YPL090C	7.0406
YDR067C	YNL099C	7.0406
YDR067C	YHL029C	7.0406
YCR095C	YDR067C	7.0406
YDL007W	YER012W	7.0405
YBL064C	YDR069C	7.0405
YCL054W	YPL093W	7.0367
YPL195W	YPL212C	7.036
YMR120C	YNR034W	7.036
YGR258C	YHR103W	7.036
YDR237W	YLR360W	7.036
YAL020C	YGR258C	7.036
YER110C	YNL122C	7.0359
YNL025C	YOR174W	7.0337
YER092W	YOR189W	7.0335
YDR485C	YLR399C	7.0332
YDL014W	YMR229C	7.0316
YBR127C	YOR048C	7.0312
YAL032C	YLR275W	7.0307
YML016C	YOR054C	7.0306
YBL046W	YDR379W	7.0306
YBL041W	YML130C	7.0306
YBL041W	YGR284C	7.0306
YIR022W	YML055W	7.0276
YIL076W	YKR067W	7.0274
YER007W	YMR145C	7.0265
YDR363W	YGL158W	7.0265
YGR048W	YHR205W	7.0264
YKR081C	YNL110C	7.0247
YCL040W	YNL189W	7.0245
YNL085W	YPL153C	7.0239
YDR238C	YPL088W	7.0239
YML093W	YOR145C	7.0231
YDL159W	YPL115C	7.0212
YDL160C	YPR082C	7.0208
YFR010W	YNL047C	7.0192
YDR392W	YPL047W	7.0178
YDL132W	YNL103W	7.0148
YER165W	YGR162W	7.0143
YDL134C	YNL154C	7.0136
YBR214W	YDL047W	7.0122
YJR002W	YPL266W	7.0094
YDR087C	YDR496C	7.0077
YGR187C	YNL317W	7.007
YER098W	YOL111C	7.007
YDR267C	YER098W	7.007
YBR112C	YLR313C	7.007
YBR112C	YJL156C	7.007
YFR040W	YGL056C	7.0059
YDR364C	YMR288W	7.0049
YDR145W	YHR099W	7.0044
YFR015C	YMR311C	7.0039
YDR130C	YMR311C	7.0039
YBR187W	YPL217C	7.0029
YIL035C	YOR151C	7.002
YGL003C	YIL142W	7.002
YDR190C	YNR035C	6.9998
YDR394W	YER012W	6.9983
YLL036C	YPR134W	6.9981
YAL038W	YGR071C	6.9968
YNL249C	YNR033W	6.9962
YAL034W-A	YBR211C	6.9962
YLR398C	YML056C	6.9946
YDR025W	YJR145C	6.9946
YGR091W	YJR022W	6.9945
YGR067C	YLR335W	6.9937
YDR156W	YJL076W	6.9935
YCL010C	YNL236W	6.9932
YER133W	YGL242C	6.993
YGR245C	YKL009W	6.9926
YBR162C	YMR214W	6.9914
YDR228C	YKR002W	6.9901
YER012W	YKL145W	6.9895
YBR118W	YPR080W	6.9893
YBL084C	YOR242C	6.9889
YGR128C	YLL011W	6.9888
YDR363W-A	YMR191W	6.9884
YDR224C	YDR303C	6.9883
YEL031W	YGL011C	6.988
YDL047W	YPL106C	6.987
YER112W	YML049C	6.9857
YEL030W	YLR043C	6.9851
YDR198C	YGR002C	6.9845
YGL150C	YOR189W	6.9843
YKL110C	YPL101W	6.9818
YHR187W	YKL110C	6.9818
YGR200C	YKL110C	6.9818
YGL172W	YJL039C	6.9818
YEL018W	YPL066W	6.9809
YFR036W	YGL240W	6.98
YLL011W	YPL217C	6.979
YDR448W	YOL135C	6.9768
YJL138C	YOR133W	6.9761
YFR050C	YIL075C	6.9727
YML007W	YPR168W	6.9721
YOR189W	YPL235W	6.9719
YNL330C	YOR244W	6.9715
YDR190C	YER178W	6.9709
YFL034C-A	YMR068W	6.9705
YDR214W	YER090W	6.9696
YBR109C	YIL034C	6.9696
YBR122C	YMR024W	6.9684
YER127W	YKR060W	6.9657
YFL017W-A	YKL173W	6.9642
YNR043W	YOR057W	6.962
YDR069C	YDR207C	6.962
YCL057W	YNR031C	6.962
YCL057W	YCR073C	6.962
YFL034W	YFR043C	6.9619
YDR475C	YOR227W	6.9575
YBR125C	YER089C	6.9575
YBR082C	YER068W	6.9575
YBR082C	YDR059C	6.9575
YBR245C	YOR207C	6.9566
YFR031C-A	YJL177W	6.9564
YAL036C	YOR253W	6.9557
YMR199W	YPL014W	6.9537
YLR144C	YMR199W	6.9537
YKR091W	YMR199W	6.9537
YJL123C	YJL146W	6.9537
YGR028W	YJL146W	6.9537
YBR229C	YML067C	6.9537
YAL042W	YBR229C	6.9537
YMR288W	YOR308C	6.9521
YBR189W	YNL207W	6.9521
YBL004W	YGR128C	6.9521
YGR232W	YNL049C	6.9518
YBR009C	YFR037C	6.9511
YJL109C	YPR144C	6.9508
YDL043C	YLR298C	6.9496
YKL019W	YKL152C	6.949
YDR498C	YGL098W	6.949
YGL128C	YPL213W	6.9482
YER125W	YFL010C	6.948
YLR133W	YPL106C	6.9473
YMR105C	YMR120C	6.9459
YGL252C	YMR284W	6.9443
YGR002C	YMR075W	6.9435
YBR063C	YDR369C	6.9422
YNL306W	YPL013C	6.9402
YKL003C	YPL118W	6.9402
YKL003C	YNL306W	6.9402
YHL004W	YKL003C	6.9402
YBR146W	YKL003C	6.9402
YIL094C	YNL037C	6.9397
YPL235W	YPR183W	6.9389
YDL014W	YDL148C	6.938
YLR438C-A	YMR268C	6.9355
YJR084W	YPR045C	6.9355
YLR389C	YPL184C	6.9353
YDR448W	YML007W	6.9333
YBR253W	YDR448W	6.9333
YIL070C	YKR048C	6.9328
YCL029C	YPL032C	6.9312
YCL029C	YMR134W	6.9312
YDR473C	YER146W	6.9311
YJL002C	YML019W	6.9308
YGL022W	YML019W	6.9308
YLR330W	YNL045W	6.9269
YHR023W	YJR112W	6.9269
YLL008W	YNL110C	6.924
YDR494W	YJR113C	6.9217
YBR049C	YLR357W	6.9195
YKL012W	YML049C	6.9187
YGL157W	YML091C	6.9183
YER171W	YHR042W	6.9183
YDR325W	YLR086W	6.9183
YDR320C-A	YKL052C	6.9183
YHR081W	YPR190C	6.9182
YGR061C	YPR185W	6.9173
YGL180W	YGR061C	6.9173
YOR243C	YOR356W	6.9171
YLR355C	YMR303C	6.9159
YBR283C	YEL002C	6.9159
YGR090W	YHR148W	6.9152
YDR448W	YMR005W	6.9151
YBR289W	YDR073W	6.9148
YJL008C	YOR259C	6.9146
YIL093C	YKL003C	6.914
YNR034W	YOL063C	6.9129
YJL047C	YNR034W	6.9129
YGR185C	YNL230C	6.9129
YGR163W	YGR203W	6.9129
YGR098C	YGR203W	6.9129
YGR098C	YGR163W	6.9129
YCR015C	YGR203W	6.9129
YCR015C	YGR163W	6.9129
YCR015C	YGR098C	6.9129
YBL050W	YMR197C	6.9129
YLR238W	YMR029C	6.9117
YLR175W	YPL012W	6.9113
YCR035C	YNL151C	6.908
YGR252W	YGR274C	6.9077
YNL002C	YPL043W	6.9076
YHR085W	YLR406C	6.9066
YBL004W	YHR148W	6.9063
YKL009W	YLR406C	6.9042
YBL004W	YLL011W	6.9039
YNL022C	YOR209C	6.9014
YJR066W	YNL062C	6.9014
YJR066W	YLR290C	6.9014
YJL125C	YJR066W	6.9014
YCR072C	YPL093W	6.9014
YBR269C	YDL103C	6.9014
YFR017C	YPR184W	6.9013
YDR364C	YHR165C	6.9007
YDL087C	YHR165C	6.9007
YLR222C	YNL075W	6.8993
YJL074C	YJL115W	6.8993
YLL024C	YOR043W	6.8986
YPL204W	YPL266W	6.8975
YDL185W	YKL081W	6.8954
YGR013W	YLL036C	6.8952
YKR006C	YMR098C	6.8938
YKL167C	YMR098C	6.8938
YDR475C	YMR311C	6.893
YAL036C	YNL132W	6.8912
YDR001C	YNL267W	6.8909
YML001W	YOR181W	6.8908
YLR312W-A	YPL183W-A	6.8901
YGL133W	YGR047C	6.8891
YKL152C	YML028W	6.889
YCR035C	YPR189W	6.889
YHR197W	YOR206W	6.8889
YOL052C	YPL235W	6.8886
YDR171W	YGR178C	6.8881
YDR498C	YOR075W	6.8865
YDR517W	YGR136W	6.8864
YER031C	YML001W	6.8856
YKR006C	YNL284C	6.8847
YFR031C-A	YMR142C	6.8844
YBR065C	YLR117C	6.8841
YKL088W	YOR054C	6.8822
YDR170C	YLR148W	6.8818
YAL005C	YLR438W	6.8817
YER073W	YML032C	6.8815
YGR145W	YLL045C	6.8812
YDR162C	YHL007C	6.8806
YDR370C	YJR031C	6.8805
YCR071C	YMR098C	6.8802
YFR052W	YJL008C	6.8771
YGR013W	YMR288W	6.8754
YDL148C	YPR169W	6.8744
YEL013W	YLR447C	6.8738
YHR029C	YJL146W	6.8737
YAL014C	YOR036W	6.8737
YDR067C	YNL032W	6.8736
YER107C	YLR276C	6.8724
YDR168W	YJR076C	6.8723
YIL035C	YNL330C	6.8711
YJL124C	YLR275W	6.8698
YER161C	YIR001C	6.8696
YBL007C	YML001W	6.8693
YJL008C	YJR064W	6.869
YIL034C	YOR326W	6.8669
YBL071W-A	YEL037C	6.8653
YHR064C	YKR048C	6.865
YLL005C	YMR255W	6.8643
YGL045W	YLR119W	6.8643
YDL075W	YDR471W	6.8643
YGR162W	YNL262W	6.8641
YDR060W	YNL301C	6.8614
YBR142W	YLL008W	6.8602
YGR178C	YHR121W	6.8582
YEL031W	YOR362C	6.8577
YER021W	YOL038W	6.8575
YCR079W	YOL005C	6.8573
YOL058W	YPL235W	6.8568
YEL013W	YMR307W	6.8567
YBR202W	YGL001C	6.8567
YGR145W	YPL126W	6.8561
YNR037C	YOR243C	6.8552
YGR215W	YOR243C	6.8552
YLL034C	YLR449W	6.8538
YDL030W	YGL128C	6.8538
YER133W	YHR119W	6.8529
YGL105W	YHL029C	6.8522
YCR095C	YGL105W	6.8522
YDR473C	YEL015W	6.8506
YDL014W	YGR054W	6.8493
YLR312W-A	YNL005C	6.8491
YBR257W	YNL282W	6.8484
YKR048C	YML056C	6.8483
YFR019W	YKL142W	6.8478
YGL241W	YOL012C	6.8476
YML091C	YPR116W	6.8442
YLR154C	YNL072W	6.8442
YLR091W	YML091C	6.8442
YLR079W	YPR120C	6.8442
YLL033W	YML091C	6.8442
YJL061W	YJL122W	6.8442
YGR185C	YNL247W	6.8442
YER016W	YOR220W	6.8442
YER016W	YLR256W	6.8442
YER016W	YGL181W	6.8442
YDR415C	YOR184W	6.8442
YDR065W	YML091C	6.8442
YBR059C	YER016W	6.8442
YDL040C	YGR054W	6.8405
YHR009C	YHR174W	6.8394
YDL040C	YNL132W	6.8388
YCL054W	YGL111W	6.8379
YJL148W	YNR003C	6.8377
YBR175W	YGL039W	6.8359
YNL110C	YNR053C	6.8357
YDR240C	YPR057W	6.8351
YDR416W	YPR178W	6.8329
YER102W	YOR253W	6.8325
YHR019C	YHR064C	6.8323
YGL066W	YPL047W	6.8305
YLR033W	YMR125W	6.8299
YLR021W	YPL144W	6.8285
YGR033C	YLR218C	6.8285
YEL039C	YPL144W	6.8285
YEL039C	YLR021W	6.8285
YCL057W	YLR006C	6.8285
YMR098C	YMR193W	6.8268
YGL171W	YJL109C	6.8258
YBR025C	YFL045C	6.8258
YGL234W	YJR068W	6.8255
YOR308C	YPR018W	6.8248
YJL187C	YOR023C	6.8248
YHR016C	YOR367W	6.8248
YGR033C	YOL100W	6.8248
YGL070C	YPR093C	6.8248
YDR200C	YFR008W	6.8248
YBR195C	YOR308C	6.8248
YBR145W	YDR499W	6.8248
YKR082W	YLR208W	6.8247
YIL149C	YNL128W	6.8247
YIL066C	YLR163C	6.8247
YHR024C	YIL066C	6.8247
YHL039W	YLR208W	6.8247
YFL034W	YGL156W	6.8247
YBR139W	YBR280C	6.8247
YBR014C	YGL020C	6.8247
YER017C	YFL018C	6.8246
YDR529C	YKL010C	6.8246
YBR267W	YJL122W	6.8244
YDR494W	YGR165W	6.8229
YLR133W	YMR284W	6.8224
YHR114W	YLR447C	6.8212
YBL007C	YLR447C	6.8212
YMR193W	YNR022C	6.8207
YML123C	YMR029C	6.8207
YKR085C	YNR022C	6.8207
YJL124C	YLR147C	6.8207
YGR220C	YNR022C	6.8207
YIL075C	YLR278C	6.82
YHL020C	YLR305C	6.8196
YER120W	YLR305C	6.8196
YGR155W	YPL061W	6.8168
YHR085W	YKL009W	6.8158
YCR073W-A	YER133W	6.8158
YHR107C	YNL233W	6.8156
YMR290C	YOR253W	6.8146
YBR084W	YLR340W	6.8144
YMR165C	YNL050C	6.813
YDR424C	YKR082W	6.813
YDR266C	YMR165C	6.813
YDR168W	YHR107C	6.8129
YER125W	YOR322C	6.8125
YBR278W	YCL032W	6.81
YER068W	YKL010C	6.8091
YDR059C	YKL010C	6.8091
YER112W	YGL121C	6.8087
YBR065C	YPL213W	6.8087
YJL081C	YOR189W	6.8085
YNL236W	YOL148C	6.8068
YDR005C	YNR003C	6.8044
YHR064C	YPL048W	6.8036
YNL005C	YNL177C	6.8026
YLR424W	YPR178W	6.8021
YBL006C	YLR321C	6.8006
YPL084W	YPR173C	6.7996
YLR025W	YPL084W	6.7996
YKR018C	YNR029C	6.7996
YHR187W	YMR312W	6.7996
YDR434W	YLR088W	6.7996
YCR004C	YDR032C	6.7996
YDR189W	YKL196C	6.7994
YER036C	YER070W	6.7991
YHR023W	YPL233W	6.7977
YGL127C	YMR112C	6.7977
YBR156C	YJR089W	6.7977
YHR088W	YPR016C	6.7971
YIL033C	YNL227C	6.7966
YER178W	YPL235W	6.7959
YDR443C	YDR448W	6.7922
YLL008W	YLR276C	6.7915
YKL022C	YNL250W	6.7913
YLR275W	YNR011C	6.7905
YDL098C	YPL213W	6.7902
Q0140	YGR211W	6.7902
YLR117C	YMR240C	6.7899
YIL075C	YPR103W	6.7889
YOR001W	YPR189W	6.7888
YJR113C	YKL003C	6.7881
YDL175C	YJR145C	6.7861
YDR068W	YDR483W	6.7838
YAL021C	YER068W	6.7838
YBL003C	YOR304W	6.7836
YDL005C	YHR041C	6.7835
YBR160W	YFR030W	6.7828
YHR023W	YIL034C	6.7813
YML062C	YNL189W	6.7803
YDR028C	YJL042W	6.7795
YFR052W	YNL049C	6.7794
YBR055C	YER146W	6.7791
YHR020W	YML085C	6.7789
YDR240C	YPR182W	6.777
YHR111W	YIL008W	6.7753
YKL188C	YLR275W	6.7741
YJL074C	YLR347C	6.7716
YMR291W	YPL262W	6.7706
YLR396C	YPL262W	6.7706
YBR081C	YGL244W	6.7706
YBL017C	YPL049C	6.7684
YAL014C	YOR212W	6.7684
YAL014C	YDR264C	6.7684
YAL034W-A	YHR023W	6.7679
YOR206W	YOR243C	6.7677
YFR001W	YNL061W	6.7669
YDR448W	YHL047C	6.7647
YMR125W	YPL151C	6.7643
YJR156C	YLR423C	6.7642
YJR070C	YPL153C	6.7642
YER171W	YLR005W	6.7642
YEL013W	YNL168C	6.7642
YBL006C	YKR008W	6.764
YKL003C	YKL155C	6.7639
YGR084C	YKL003C	6.7639
YGL129C	YKL003C	6.7639
YDR471W	YGL103W	6.762
YER172C	YGR013W	6.7616
YER110C	YJR077C	6.761
YDR060W	YLL034C	6.7607
YHR052W	YPL131W	6.7605
YLL011W	YLR409C	6.7604
YBL017C	YFR052W	6.7592
YDL014W	YOR312C	6.7581
YGL016W	YIL128W	6.7579
YGR285C	YKL081W	6.7573
YKR006C	YPL173W	6.7569
YJL023C	YNL185C	6.7569
YGR132C	YLR342W	6.7569
YCR002C	YDR168W	6.7569
YLR117C	YLR147C	6.7567
YER021W	YFL007W	6.7567
YLL034C	YPL211W	6.7564
YDR052C	YDR427W	6.7555
YGR275W	YHL025W	6.7549
YBR173C	YOR362C	6.754
YGR123C	YMR313C	6.7533
YBR170C	YDR330W	6.7533
YIR006C	YNL243W	6.7531
YHR191C	YMR078C	6.7531
YER006W	YKR081C	6.7522
YDR494W	YHL004W	6.752
YDL087C	YJR084W	6.752
YGL048C	YIL107C	6.7519
YFL017W-A	YGR074W	6.7519
YDR385W	YLR259C	6.7508
YBL099W	YKL016C	6.7493
YBL099W	YDR298C	6.7493
YIL068C	YLR430W	6.7471
YJR148W	YLR259C	6.7467
YLR086W	YLR163C	6.7455
YAR042W	YLR397C	6.7455
YJL001W	YNR003C	6.7454
YDL060W	YJR145C	6.7454
YDR141C	YIL055C	6.7447
YDL112W	YOR266W	6.7447
YDL112W	YNL307C	6.7447
YDL112W	YIL105C	6.7447
YDL006W	YOL113W	6.7447
YDL006W	YLR450W	6.7447
YBR088C	YER041W	6.7447
YDR342C	YDR343C	6.7446
YER122C	YFR051C	6.7426
YOL063C	YPR164W	6.7425
YGL004C	YOR259C	6.7403
YGL173C	YGR086C	6.7402
YMR173W	YMR277W	6.7389
YGR188C	YMR304W	6.7389
YFL014W	YMR322C	6.7389
YDR533C	YFL014W	6.7389
YDR515W	YHR023W	6.7389
YDR453C	YNL247W	6.7389
YDR101C	YJL061W	6.7389
YBR173C	YML119W	6.7389
YFR019W	YKL117W	6.738
YBR065C	YDL030W	6.7353
YFR001W	YPL129W	6.7337
YDL111C	YNR024W	6.7326
YDR480W	YGL245W	6.732
YJL020C	YJL146W	6.7306
YDR075W	YDR379W	6.7306
YDR037W	YLR438C-A	6.7306
YGL195W	YJR132W	6.7293
YBR081C	YPL047W	6.7282
YBR065C	YCR063W	6.7276
YDL115C	YGL043W	6.727
YGR002C	YOR141C	6.7267
YKL188C	YLR147C	6.7251
YDR188W	YMR028W	6.725
YCR081W	YDR448W	6.7238
YDR036C	YOR158W	6.7233
YGL049C	YNL016W	6.7229
YOR150W	YPR100W	6.7223
YGL066W	YHL047C	6.7221
YNL308C	YPL043W	6.722
YAL034C	YOL004W	6.7213
YGR253C	YML130C	6.7196
YGR253C	YGR284C	6.7196
YLR006C	YMR022W	6.7194
YDR243C	YOR125C	6.7194
YLR117C	YLR275W	6.7189
YGR145W	YLL011W	6.7188
YIL115C	YKR082W	6.7177
YCR003W	YNL284C	6.7177
YER012W	YHL030W	6.7176
YIL126W	YNR003C	6.7175
YGL120C	YGR159C	6.7171
YFL024C	YLR455W	6.7168
YDR060W	YOR272W	6.7158
YDL182W	YML063W	6.7137
YLR355C	YOR151C	6.7129
YKL013C	YPL235W	6.7121
YHR174W	YMR186W	6.7111
YDR462W	YLR360W	6.7108
YJL137C	YLR321C	6.7101
YML029W	YOL013C	6.7086
YLR207W	YML029W	6.7086
YDR057W	YML029W	6.7086
YBL075C	YJL155C	6.7086
YKL035W	YNL075W	6.7073
YIR029W	YKL003C	6.7073
YHR074W	YJL130C	6.7073
YER132C	YJL130C	6.7073
YER132C	YHR074W	6.7073
YHR020W	YOR217W	6.7066
YDR237W	YHR147C	6.7063
YMR309C	YOR204W	6.7057
YDR328C	YNL103W	6.7054
YMR196W	YMR275C	6.7038
YML029W	YMR277W	6.7038
YIL156W	YOR124C	6.7038
YGR128C	YLR197W	6.703
YBL039C	YPL249C	6.703
YDR036C	YNR037C	6.7024
YDR036C	YGR215W	6.7024
YPL211W	YPR016C	6.7015
YKR060W	YLR222C	6.6997
YKR085C	YPR100W	6.6993
YNR050C	YOR164C	6.6978
YCR071C	YMR225C	6.6964
YCR071C	YML009C	6.6964
YLR276C	YNL002C	6.6951
YER133W	YMR061W	6.695
YER031C	YFL005W	6.6948
YGL049C	YGR250C	6.6947
YDR023W	YOL117W	6.6901
YAL036C	YDL040C	6.6889
YKL009W	YNL110C	6.6883
YBR109C	YFL039C	6.6882
YBL038W	YPR100W	6.6878
YCR065W	YPL002C	6.6867
YNL075W	YPR144C	6.6847
YLL008W	YOR063W	6.683
YBR049C	YDR303C	6.6827
YBR009C	YBR245C	6.6812
YCL059C	YNL308C	6.681
YDR071C	YJL115W	6.6802
YOL023W	YOL070C	6.68
YDL035C	YDR098C	6.68
YCL054W	YDR101C	6.6788
YGR090W	YLL011W	6.678
YDR299W	YLR186W	6.6776
YBR158W	YGR203W	6.6766
YBR158W	YGR163W	6.6766
YBR158W	YGR098C	6.6766
YBR158W	YCR015C	6.6766
YOR136W	YPL061W	6.6756
YOL144W	YOR310C	6.6748
YGR156W	YKL193C	6.6747
YJL137C	YKR008W	6.6735
YGL164C	YOR185C	6.6733
YBR170C	YOR201C	6.6733
YDL098C	YER112W	6.6732
YHR165C	YNR011C	6.6716
YGR165W	YOR204W	6.6715
YBR188C	YHR165C	6.6712
YBR018C	YOR027W	6.6708
YGL092W	YLR359W	6.6707
YDL148C	YER179W	6.6707
YLR289W	YPL170W	6.6705
YOL013C	YPL160W	6.67
YKL190W	YNL104C	6.67
YDR454C	YEL023C	6.67
YML057W	YMR109W	6.6691
YDR073W	YPR034W	6.6688
YOR362C	YPL249C	6.6685
YFR015C	YKL193C	6.6685
YDR130C	YKL193C	6.6685
YJR076C	YNL233W	6.6661
YBR052C	YCR004C	6.6661
YAL024C	YGR238C	6.6661
YGR054W	YMR290C	6.6658
YDR496C	YNL175C	6.6638
YJL207C	YKR017C	6.6627
YHR034C	YPL053C	6.6627
YGL251C	YNL127W	6.6627
YGL131C	YNL127W	6.6627
YGL131C	YGL251C	6.6627
YDR495C	YKR017C	6.6627
YDR495C	YJL207C	6.6627
YDR334W	YPL053C	6.6627
YDR227W	YGR282C	6.6627
YDL042C	YGR282C	6.6627
YLR398C	YOR001W	6.6621
YGR145W	YLR222C	6.6603
YDL175C	YER165W	6.6594
YAL017W	YNL267W	6.6592
YAL017W	YBR001C	6.6592
YBR282W	YNL177C	6.6591
YFL039C	YNL138W	6.6581
YJL203W	YPL151C	6.6573
YMR125W	YMR146C	6.6568
YJL155C	YOR219C	6.6567
YFL037W	YIL094C	6.6565
YHR147C	YMR225C	6.6562
YHR147C	YML009C	6.6562
YML130C	YOR362C	6.6556
YGR284C	YOR362C	6.6556
YCL011C	YKL139W	6.6541
YBR025C	YDL185W	6.6537
YDL097C	YMR191W	6.6518
YMR310C	YNL009W	6.6502
YHR132W-A	YPL152W	6.6502
YGL137W	YGL245W	6.6501
YIL094C	YOR136W	6.6499
YDL108W	YDR012W	6.6498
YER172C	YLR382C	6.6494
YDL229W	YOL090W	6.6494
YDL043C	YGR091W	6.6485
YBR065C	YDR364C	6.6485
YDL148C	YLR197W	6.6484
YDL030W	YKL012W	6.6483
YMR049C	YOL041C	6.6472
YMR024W	YOR150W	6.6469
YGR086C	YPL074W	6.6469
YDR378C	YPR182W	6.6468
YLR079W	YLR210W	6.6455
YFR002W	YGL172W	6.6455
YDL188C	YPR040W	6.6454
YBR245C	YGR056W	6.6442
YOL006C	YPR190C	6.6438
YGL097W	YNL247W	6.6437
YFL014W	YPL075W	6.6437
YFR043C	YKL135C	6.6436
YMR193W	YMR225C	6.6431
YML009C	YMR193W	6.6431
YBR109C	YKL007W	6.6431
YGR159C	YLR197W	6.6427
YOR116C	YOR341W	6.6418
YDL060W	YIL069C	6.6407
YDR176W	YHL047C	6.6404
YDR494W	YIL093C	6.6392
YBR245C	YFL013C	6.6392
YCR002C	YNL233W	6.6387
YKL009W	YLL008W	6.638
YGR086C	YNL208W	6.6379
YGR218W	YHR027C	6.6377
YGR040W	YPL115C	6.6375
YDR005C	YOR207C	6.6372
YDR334W	YML041C	6.6367
YGR202C	YOL021C	6.6363
YDR012W	YOR312C	6.6356
YGR063C	YPL129W	6.6323
YPL047W	YPL254W	6.6311
YGL151W	YGR252W	6.6309
YKL061W	YNL086W	6.6308
YGL079W	YKL061W	6.6308
YEL005C	YKL061W	6.6308
YDR357C	YNL086W	6.6308
YDR357C	YGL079W	6.6308
YDR357C	YEL005C	6.6308
YDR283C	YLL005C	6.6308
YBR156C	YPL209C	6.6308
YBR156C	YGR031W	6.6308
YBL086C	YLR045C	6.6308
YAL019W	YPL224C	6.6308
YGL246C	YNL189W	6.6303
YBR278W	YNL262W	6.6288
YDR324C	YPL266W	6.6276
YKL014C	YNL175C	6.627
YGR074W	YHR156C	6.6265
YML056C	YOL115W	6.6261
YIL079C	YML056C	6.6261
YDR225W	YOR178C	6.6261
YDR225W	YOL054W	6.6261
YJR042W	YKL186C	6.6253
YGL092W	YKL186C	6.6253
YDL116W	YKL186C	6.6253
YDL019C	YGL242C	6.6253
YBR177C	YDL019C	6.6253
YDR432W	YPR191W	6.6243
YPL029W	YPL194W	6.6242
YGL208W	YMR086W	6.6242
YGL090W	YLR288C	6.6242
YBR014C	YER083C	6.6242
YBR014C	YDL100C	6.6242
YDL014W	YLR222C	6.624
YBR245C	YLR321C	6.6232
YDR188W	YHR104W	6.6214
YJL122W	YLR276C	6.6212
YBR169C	YGR171C	6.6212
YBR122C	YMR225C	6.6169
YBR122C	YML009C	6.6169
YPL129W	YPR187W	6.6166
YDR190C	YOL052C	6.6151
YIL061C	YJR022W	6.6134
YCR063W	YGR278W	6.6128
YBL085W	YBR238C	6.6121
YDR140W	YPL031C	6.6117
YCL054W	YOR272W	6.6107
YGL241W	YOR207C	6.6098
YML046W	YML049C	6.6091
YDL225W	YIL079C	6.6087
YBR087W	YMR181C	6.6087
YCR088W	YIL095W	6.6073
YKL009W	YPL081W	6.6055
YJR132W	YMR110C	6.6055
YFR036W	YKL022C	6.6055
YMR027W	YPR036W	6.6054
YLR090W	YPR036W	6.6054
YDL112W	YPL220W	6.6054
YBR227C	YBR281C	6.6051
YMR191W	YOR117W	6.605
YGL048C	YMR191W	6.605
YER171W	YNL064C	6.605
YOR310C	YPL217C	6.6049
YAL038W	YGL246C	6.6045
YDR087C	YHR052W	6.6028
YNL088W	YPL074W	6.6017
YMR209C	YPL215W	6.6017
YML057W	YPR159W	6.6013
YGL049C	YNL262W	6.5992
YBR215W	YJR070C	6.5992
YBL017C	YDR342C	6.5992
YHR024C	YLR086W	6.599
YDR517W	YPL046C	6.5989
YDR517W	YJR052W	6.5989
YGR195W	YPR189W	6.5988
YGL241W	YOR116C	6.5977
YMR116C	YNL244C	6.5965
YDL195W	YPR181C	6.5964
YBL007C	YJL020C	6.5961
YDL014W	YDR299W	6.5947
YNL207W	YOR065W	6.5931
YLR107W	YOR065W	6.5931
YLR107W	YNL207W	6.5931
YGL189C	YOR065W	6.5931
YGL189C	YNL207W	6.5931
YGL189C	YLR107W	6.5931
YDL028C	YJR149W	6.5931
YCR047C	YNR046W	6.5931
YDR449C	YGR198W	6.593
YHR084W	YKR028W	6.5926
YGL212W	YKR028W	6.5926
YDR480W	YKR028W	6.5926
YDR101C	YNL182C	6.5921
YDL148C	YDR299W	6.5906
YGR202C	YHR069C	6.5905
YEL018W	YNL189W	6.5902
YJL041W	YKR082W	6.59
YJL023C	YKL192C	6.59
YGL157W	YJL023C	6.59
YCR003W	YPL173W	6.59
YLR314C	YNL233W	6.5861
YLR447C	YMR054W	6.5855
YJR002W	YKR060W	6.5847
YGL099W	YHR170W	6.5846
YJL203W	YML046W	6.5811
YDR347W	YKL003C	6.5803
YBR127C	YKL007W	6.5802
YDR381W	YLR249W	6.5799
YCL014W	YOR150W	6.5798
YKR063C	YOR370C	6.5796
YDR143C	YPL161C	6.5796
YBL021C	YML048W	6.5796
YGR054W	YNL207W	6.5791
YCR072C	YHR085W	6.5785
YMR304W	YPR119W	6.5778
YPL106C	YPR080W	6.5765
YOR227W	YPL137C	6.5747
YLR163C	YNL242W	6.5747
YHR024C	YNL242W	6.5747
YDR475C	YPL137C	6.5747
YIL070C	YML069W	6.5735
YJR084W	YPR082C	6.5732
YGR252W	YHR041C	6.5709
YBR143C	YDR091C	6.5707
YGR220C	YLR312W-A	6.5686
YER070W	YLR153C	6.5686
YDR028C	YDR130C	6.5685
YDR005C	YOR116C	6.5672
YAR014C	YDL047W	6.5669
YKR024C	YKR081C	6.5663
YIL103W	YOL109W	6.5662
YDL040C	YER102W	6.566
YDL073W	YEL003W	6.5657
YKR006C	YPL183W-A	6.5652
YKL167C	YPL183W-A	6.5652
YNL175C	YPL012W	6.565
YDL051W	YGR162W	6.5649
YMR187C	YPL048W	6.564
YJL157C	YLR430W	6.5639
YHR148W	YNL075W	6.5623
YGR145W	YNL308C	6.5621
YGR252W	YOR174W	6.561
YKL110C	YPL086C	6.5608
YGR027C	YPR004C	6.5608
YKR081C	YNL230C	6.5605
YAL016W	YNL154C	6.5603
YJL115W	YMR135C	6.5598
YDR240C	YPL213W	6.5596
YOL127W	YPL093W	6.5579
YDL097C	YNL115C	6.5577
YKR085C	YMR098C	6.5568
YCL014W	YKR085C	6.5568
YLR408C	YNL086W	6.5567
YGL079W	YLR408C	6.5567
YEL005C	YLR408C	6.5567
YDR320C-A	YGR113W	6.5567
YBR123C	YDR397C	6.5566
YBR109C	YGR130C	6.5557
YDL002C	YNL215W	6.5547
YDL098C	YOR159C	6.5531
YLR189C	YOR150W	6.5525
YMR033W	YPL129W	6.5523
YCR071C	YPL183W-A	6.5516
YMR052W	YNL127W	6.5515
YLL048C	YOL031C	6.5515
YGL251C	YMR052W	6.5515
YGL131C	YMR052W	6.5515
YDR243C	YLR183C	6.5515
YGR090W	YJL069C	6.5499
YAR007C	YFR037C	6.5499
YGR194C	YNL265C	6.5484
YDR350C	YMR282C	6.5484
YDR260C	YIR025W	6.5484
YDR260C	YGL240W	6.5484
YDR023W	YMR282C	6.5484
YDR023W	YDR350C	6.5484
YDL003W	YER147C	6.5484
YHL047C	YLR055C	6.5457
YER012W	YIL075C	6.5454
YOR374W	YPL243W	6.5453
YHR039C-A	YKL080W	6.5453
YFL024C	YPL074W	6.5453
YDR202C	YKL080W	6.5453
YDL019C	YLR305C	6.5453
YBL038W	YMR098C	6.5453
YBL038W	YCL014W	6.5453
YAL014C	YMR197C	6.5453
YDR496C	YKR081C	6.5451
YLR182W	YPL160W	6.5449
YHR196W	YJR002W	6.5434
YFL002C	YLR449W	6.5432
YDL060W	YNL308C	6.5424
YBR130C	YLR429W	6.5423
YDR419W	YDR477W	6.5419
YKL007W	YOR326W	6.5406
YCR079W	YGR186W	6.5397
YDR143C	YJR068W	6.5393
YBL058W	YPL133C	6.5389
YDR167W	YPL047W	6.5386
YKR024C	YNL061W	6.5384
YHR197W	YLR074C	6.5378
YIL149C	YPL164C	6.5372
YIL108W	YOL126C	6.5372
YFL034C-B	YIL129C	6.5372
YBR198C	YOR308C	6.5372
YBR133C	YOR023C	6.5372
YBR133C	YJL187C	6.5372
YLR306W	YPL003W	6.537
YCR009C	YLR429W	6.537
YJR140C	YPL153C	6.5361
YIL127C	YMR099C	6.5361
YDR301W	YKL193C	6.5356
YBL003C	YFR037C	6.5355
YHR131C	YIL035C	6.535
YER029C	YFL017W-A	6.5347
YBR109C	YGL242C	6.5338
YMR213W	YMR288W	6.532
YMR124W	YOL031C	6.5302
YMR124W	YNL238W	6.5302
YBR187W	YPR112C	6.5302
YDL153C	YGL242C	6.5299
YLR430W	YPL178W	6.5295
YKR085C	YLR189C	6.5295
YMR284W	YMR308C	6.5287
YJR084W	YPL213W	6.5284
YBR193C	YDR448W	6.5268
YDR507C	YLR314C	6.526
YNL189W	YPL153C	6.5252
YIL126W	YJL176C	6.525
YPL122C	YPR056W	6.5215
YDR079C-A	YPR056W	6.5215
YBR065C	YER172C	6.5213
YEL034W	YLR249W	6.5203
YJL109C	YLL011W	6.5195
YDR405W	YKL167C	6.5192
YAL021C	YHR193C	6.5192
YDL087C	YPR182W	6.5187
YDL098C	YJL124C	6.5186
YBL038W	YLR189C	6.5181
YHR069C	YKR025W	6.5171
YPR111W	YPR122W	6.5166
YOR210W	YPR122W	6.5166
YOR210W	YPR111W	6.5166
YLR326W	YNL323W	6.5166
YLR267W	YOR057W	6.5166
YGL116W	YJL013C	6.5166
YEL023C	YMR104C	6.5166
YDR139C	YPL038W	6.5166
YBR175W	YMR190C	6.5166
YBL080C	YMR104C	6.5166
YBL080C	YEL023C	6.5166
YNL044W	YNL307C	6.5159
YJL023C	YPR116W	6.5159
YJL023C	YPL204W	6.5159
YJL023C	YLR091W	6.5159
YJL023C	YLL033W	6.5159
YDR065W	YJL023C	6.5159
YCR053W	YNL022C	6.5159
YDL047W	YLL024C	6.5156
YBR136W	YER104W	6.515
YER112W	YOR159C	6.5138
YGL150C	YNL215W	6.5126
YGR094W	YMR234W	6.512
YBL036C	YBL039C	6.5119
YHL021C	YJL140W	6.5118
YGR145W	YOR310C	6.511
YMR311C	YPL137C	6.5102
YAR014C	YIL106W	6.5102
YOR252W	YPR047W	6.5099
YDR312W	YKL014C	6.5098
YCR020W-B	YDR303C	6.5093
YLR409C	YNL132W	6.5058
YMR029C	YMR319C	6.5047
YML056C	YOR360C	6.5044
YDR473C	YMR268C	6.5032
YEL022W	YOR153W	6.503
YDR449C	YGR145W	6.5024
YCR035C	YLR398C	6.5021
YDR041W	YDR494W	6.5018
YMR181C	YOR217W	6.5002
YBL006C	YCR052W	6.5
YCR077C	YMR304W	6.4991
YBL076C	YLR086W	6.4986
YJR145C	YNR054C	6.4984
YNL182C	YPL093W	6.4983
YDR314C	YMR187C	6.4973
YBL027W	YJL177W	6.497
YBR231C	YNL071W	6.4968
YMR275C	YPR187W	6.4914
YGR013W	YMR240C	6.4907
YHR196W	YLR197W	6.4905
YKL143W	YOR056C	6.4901
YFL024C	YLR347C	6.4898
YJR002W	YOL010W	6.4894
YBR173C	YFL007W	6.4878
YDR381W	YNL253W	6.4867
YEL015W	YPR178W	6.4863
YMR290C	YPR016C	6.4856
YGL246C	YOR361C	6.4854
YIL159W	YOR195W	6.485
YHR165C	YLR382C	6.485
YCR072C	YKL009W	6.4839
YEL018W	YOL004W	6.4837
YIL018W	YLL045C	6.4829
YHR072W-A	YPL240C	6.4819
YER117W	YML056C	6.4797
YHR064C	YLL026W	6.4787
YMR098C	YNL252C	6.4782
YKL214C	YNL189W	6.4782
YBR088C	YEL030W	6.4773
YIL115C	YMR047C	6.477
YER107C	YIL115C	6.477
YDR382W	YGL103W	6.477
YDL080C	YGR196C	6.477
YJL200C	YLR355C	6.4769
YJL061W	YKR082W	6.4767
YDR422C	YER129W	6.4767
YOR043W	YPR030W	6.4766
YDR177W	YDR226W	6.4761
YIL075C	YML057W	6.4743
YDL055C	YIL094C	6.4731
YBR122C	YPL183W-A	6.4723
YAR019C	YBR227C	6.4722
YDL007W	YLR278C	6.4716
YPL111W	YPL160W	6.4713
YDR430C	YGR193C	6.4713
YDL083C	YGR085C	6.4712
YGR240C	YIL125W	6.4708
YCR079W	YGL070C	6.4702
YER012W	YFR004W	6.4697
YNL032W	YNR033W	6.4684
YFL002C	YPR016C	6.4684
YAL035W	YLR432W	6.4672
YMR004W	YPL144W	6.4658
YLR021W	YMR004W	6.4658
YEL039C	YMR004W	6.4658
YKL164C	YPL086C	6.4656
YDR405W	YHR147C	6.4656
YDR050C	YJL049W	6.4655
YGL173C	YGR162W	6.465
YJL069C	YKR060W	6.4635
YML006C	YMR205C	6.4592
YDL148C	YLL011W	6.4585
YGL050W	YPL031C	6.4577
YLR429W	YOR326W	6.4574
YKL130C	YLR429W	6.4574
YGL106W	YLR092W	6.4572
YDL030W	YJR084W	6.4554
YHR023W	YKL007W	6.4552
YCL059C	YLR197W	6.4548
YAL027W	YCR087C-A	6.4538
YDR101C	YKL009W	6.4531
YDR405W	YMR193W	6.4524
YGR186W	YPR110C	6.4518
YFR010W	YOL022C	6.4506
YFR010W	YLR341W	6.4506
YFR010W	YGL027C	6.4506
YER041W	YFR010W	6.4506
YGR128C	YOR145C	6.4499
YKL003C	YOR019W	6.4496
YIR029W	YOR019W	6.4496
YDR496C	YOL041C	6.4495
YDL014W	YMR093W	6.448
YDL066W	YJL034W	6.447
YBL038W	YPL173W	6.447
YJR022W	YKL012W	6.4468
YDL161W	YDR229W	6.4468
YFR028C	YPR010C	6.4467
YDR060W	YNL002C	6.4467
YKR025W	YNL248C	6.4461
YBR119W	YDL043C	6.4456
YDL098C	YKL188C	6.4445
YOR036W	YOR212W	6.4431
YIR031C	YMR199W	6.4431
YGR119C	YJL039C	6.4431
YDR264C	YOR036W	6.4431
YBR069C	YPL233W	6.4431
YAL053W	YML072C	6.4431
YJL173C	YMR234W	6.443
YML001W	YOR089C	6.4427
YGL171W	YGR145W	6.4419
YBR152W	YKL173W	6.4411
YHR082C	YOR184W	6.439
YFR043C	YPR029C	6.439
YDL035C	YNL254C	6.439
YCR042C	YOL078W	6.439
YCL024W	YOL023W	6.439
YBR172C	YNL288W	6.439
YLR129W	YMR128W	6.4385
YJL020C	YLR337C	6.4337
YOL006C	YOR304W	6.4306
YDR012W	YGR090W	6.4284
YGR192C	YIL053W	6.4282
YDR198C	YJL081C	6.4282
YDR171W	YJR148W	6.4282
YLR129W	YPL217C	6.4271
YCR042C	YDR145W	6.427
YHR197W	YKL009W	6.4269
YBR122C	YDR405W	6.4264
YHR148W	YOR145C	6.4258
YDL111C	YDL150W	6.4258
YCL037C	YLR175W	6.4257
YGR267C	YNL055C	6.425
YBR152W	YDR378C	6.425
YDL175C	YPL178W	6.4241
YBL075C	YOR054C	6.4211
YKL009W	YOR206W	6.4207
YDL014W	YGR103W	6.4199
YLR208W	YLR359W	6.4195
YGL156W	YOL082W	6.4195
YCL029C	YGL156W	6.4195
YLR389C	YNL238W	6.4194
YDR137W	YLR262C	6.4194
YDL014W	YKR060W	6.4192
YHR105W	YLL040C	6.4189
YFR039C	YNL006W	6.4189
YAL016W	YDR482C	6.4189
YDL188C	YNL154C	6.4186
YLR043C	YML028W	6.4185
YGR090W	YOL041C	6.4183
YDR194C	YKL172W	6.4162
YDL159W	YLR362W	6.4151
YCL059C	YLL008W	6.4151
YDR087C	YEL026W	6.415
YNL207W	YOR056C	6.4146
YGL086W	YOL086C	6.4144
YGR278W	YMR288W	6.4143
YGR091W	YLR382C	6.4132
YDL031W	YHR197W	6.413
YMR242C	YNL069C	6.4122
YDL105W	YGR237C	6.4114
YCR016W	YPL009C	6.4109
YIL053W	YLR250W	6.4104
YIL053W	YLR080W	6.4104
YFL048C	YIL053W	6.4104
YBL090W	YOR243C	6.4103
YDR449C	YPL266W	6.4102
YBL041W	YOR261C	6.4099
YCR052W	YJL137C	6.4098
YLR448W	YMR061W	6.4096
YBR083W	YDR480W	6.409
YGL241W	YPL082C	6.4086
YGL113W	YPL153C	6.4078
YBR017C	YOR374W	6.4078
YOL012C	YOL070C	6.405
YNL078W	YOL012C	6.405
YGL103W	YHL033C	6.405
YDR060W	YGL111W	6.4037
YBL076C	YER086W	6.4033
YOR078W	YOR287C	6.4027
YDR422C	YLR408C	6.4027
YDR001C	YOR220W	6.4027
YDR001C	YLR256W	6.4027
YDR001C	YGL181W	6.4027
YBR059C	YDR001C	6.4027
YKL003C	YNL137C	6.4026
YFR043C	YPL259C	6.4026
YBR139W	YOR267C	6.4026
YBL006C	YPR034W	6.4014
YOR018W	YOR267C	6.4012
YGL097W	YJR074W	6.4012
YFR011C	YOL006C	6.4012
YER020W	YMR029C	6.4012
YBR280C	YEL060C	6.4012
YGR240C	YPL240C	6.4009
YBR286W	YGL100W	6.4006
YDR155C	YLL050C	6.3977
YDR035W	YOR230W	6.397
YMR225C	YOR150W	6.3965
YML009C	YOR150W	6.3965
YDL136W	YGL103W	6.396
YKL028W	YKR018C	6.3944
YDL073W	YML094W	6.3925
YBL024W	YOR243C	6.3925
YER178W	YFL018C	6.3905
YCR035C	YJL011C	6.3896
YDR060W	YFL002C	6.3894
YDR516C	YHR060W	6.3891
YOR206W	YOR312C	6.3885
YDL241W	YLR277C	6.3872
YEL019C	YGR237C	6.3859
YFR050C	YOR261C	6.3858
YFR037C	YNR003C	6.3858
YDR482C	YLR016C	6.3849
YBL076C	YLR060W	6.3849
YAL036C	YMR290C	6.3844
YER082C	YJL069C	6.3843
YKR025W	YOR001W	6.3838
YLR263W	YOR157C	6.3834
YAL053W	YOR157C	6.3834
YHR119W	YMR190C	6.3832
YFL009W	YOR057W	6.3832
YFL009W	YLR267W	6.3832
YDL137W	YGR052W	6.3831
YCL030C	YDL137W	6.3831
YBR247C	YDR299W	6.3828
YDR180W	YER147C	6.3825
YER078C	YHR087W	6.3824
YDR226W	YHR087W	6.3824
YDR432W	YOR204W	6.3806
YER155C	YLR310C	6.3793
YBR281C	YPL218W	6.3782
YBR281C	YJR053W	6.3782
YCL054W	YMR049C	6.3776
YDR036C	YOR243C	6.3774
YDR129C	YDR517W	6.376
YBR154C	YMR275C	6.3758
YDR477W	YER129W	6.375
YBR048W	YDR025W	6.3737
YER093C	YOL064C	6.3722
YKL095W	YPR191W	6.3721
YJL109C	YKL014C	6.3711
YOR294W	YPL093W	6.3708
YDR447C	YKL180W	6.3706
YGR013W	YJR084W	6.3704
YBR018C	YNL037C	6.3702
YIR015W	YJR031C	6.37
YGR094W	YJR031C	6.37
YDL073W	YNL153C	6.37
YDR416W	YDR473C	6.3699
YDR267C	YOR204W	6.3675
YGR074W	YMR288W	6.3672
YDR450W	YKL180W	6.3668
YBR282W	YKR006C	6.3665
YBR282W	YKL167C	6.3665
YML049C	YPR188C	6.3654
YER164W	YML049C	6.3654
YBL049W	YLR138W	6.3654
YBL049W	YKR017C	6.3654
YBL049W	YHL010C	6.3654
YDR419W	YGL115W	6.3651
YDR324C	YNL178W	6.3644
YML097C	YPR055W	6.3631
YGR081C	YPL012W	6.3631
YGL087C	YOR335C	6.3627
YDR092W	YOR335C	6.3627
YDR328C	YNL311C	6.3624
YBL038W	YMR225C	6.3621
YBL038W	YML009C	6.3621
YDR037W	YGL026C	6.3619
YGL076C	YGL242C	6.3616
YER021W	YJL008C	6.3609
YJR145C	YMR014W	6.3597
YGR192C	YLR044C	6.3597
YER022W	YNL025C	6.3597
YIL061C	YML049C	6.359
YHR174W	YPL207W	6.3581
YDR434W	YKR061W	6.358
YDR434W	YJL139C	6.358
YBL004W	YMR229C	6.3568
YDR314C	YFR052W	6.3562
YNL136W	YNL189W	6.3558
YDR175C	YDR494W	6.3545
YBR282W	YCR071C	6.353
YLR429W	YNL208W	6.3501
YDR064W	YPL081W	6.3501
YCL054W	YNL002C	6.3495
YFR001W	YNL308C	6.3488
YLR029C	YML073C	6.3485
YCR092C	YNR058W	6.3479
YDL117W	YDR099W	6.3478
YKL193C	YPL179W	6.3475
YDR122W	YNL035C	6.3475
YLR449W	YOR227W	6.3472
YNL284C	YOR150W	6.3471
YJL076W	YOR173W	6.3456
YJL076W	YLR270W	6.3456
YHR088W	YPL043W	6.3454
YGL197W	YKR028W	6.3453
YDR334W	YFL013C	6.3438
YDL215C	YER110C	6.3432
YDR175C	YJR096W	6.3429
YDL014W	YGL019W	6.3419
YFR031C-A	YHL033C	6.3414
YER006W	YLR449W	6.3414
YHR060W	YKL119C	6.3404
YGR179C	YIR010W	6.3404
YKL075C	YNL164C	6.3385
YJL020C	YOR181W	6.3385
YIL115C	YMR255W	6.3385
YDR171W	YNL261W	6.3382
YBR031W	YKR062W	6.3381
YOL058W	YOR108W	6.3379
YNL104C	YOL058W	6.3379
YML052W	YPL058C	6.3379
YLR411W	YMR251W	6.3379
YKL065C	YPL019C	6.3379
YIR032C	YMR251W	6.3379
YIR032C	YLR411W	6.3379
YIL177C	YMR251W	6.3379
YIL177C	YLR411W	6.3379
YIL177C	YIR032C	6.3379
YFR048W	YPL019C	6.3379
YFR048W	YKL065C	6.3379
YFL052W	YGR148C	6.3379
YMR268C	YNL256W	6.3378
YKL018W	YKL193C	6.3378
YDR394W	YOL022C	6.3366
YDR394W	YLR341W	6.3366
YDR394W	YGL027C	6.3366
YDR394W	YER041W	6.3366
YBR245C	YOL006C	6.3346
YJL063C	YPR100W	6.3345
YOR132W	YPL070W	6.3337
YOL068C	YOR279C	6.3337
YLR027C	YMR323W	6.3337
YIL144W	YJR032W	6.3337
YIL108W	YNL254C	6.3337
YGR262C	YNL254C	6.3337
YCR066W	YKL210W	6.3335
YHR197W	YLR002C	6.333
YBR135W	YLR210W	6.3328
YLR432W	YNL112W	6.332
YER025W	YOR361C	6.3311
YCR012W	YMR021C	6.331
YLR455W	YPR023C	6.3306
YHR088W	YKL172W	6.3305
YOR187W	YPL083C	6.3304
YKR028W	YPL049C	6.3304
YBR025C	YFR010W	6.33
YDL160C	YGR091W	6.3286
YHR143W-A	YOR224C	6.3281
YOR151C	YPR093C	6.3279
YLR147C	YPR101W	6.3278
YBR205W	YDL063C	6.3267
YMR240C	YPR101W	6.3261
YLR392C	YML123C	6.3261
YKR018C	YML123C	6.3261
YGR136W	YML123C	6.3261
YBL026W	YDL030W	6.3261
YDR416W	YML046W	6.3255
YDL132W	YDL147W	6.3255
YER136W	YML001W	6.3246
YBR065C	YMR213W	6.3242
YFL039C	YNR031C	6.3236
YCR053W	YOR209C	6.3219
YDR293C	YMR304W	6.3212
YDL213C	YLR175W	6.32
YDL074C	YPR080W	6.3181
YDL040C	YMR290C	6.3179
YDR448W	YER022W	6.3169
YHR216W	YOR250C	6.3165
YGL044C	YHR216W	6.3165
YGR054W	YPL204W	6.3162
YBR025C	YGL048C	6.3161
YGL206C	YPL106C	6.3153
YGR074W	YPL178W	6.3146
YLR016C	YLR086W	6.3142
YJR042W	YPL215W	6.3142
YIL079C	YJR145C	6.3142
YGL174W	YLR016C	6.3142
YER020W	YHR005C	6.3142
YEL071W	YOL082W	6.3142
YDL025C	YGR016W	6.3142
YBR195C	YDR324C	6.3142
YAL049C	YGR016W	6.3142
YBR274W	YPR124W	6.3141
YBR282W	YHR147C	6.313
YDL197C	YLR442C	6.3125
YGL246C	YHR174W	6.312
YJL137C	YPR034W	6.3112
YBL074C	YOR308C	6.3112
YLR429W	YNL225C	6.3105
YLR429W	YNL108C	6.3105
YGL076C	YOR063W	6.31
YDR364C	YML049C	6.3097
YJL109C	YOL010W	6.3094
YDL140C	YDR457W	6.3088
YNL112W	YPR163C	6.3086
YER146W	YGR074W	6.3084
YBL024W	YDR283C	6.3056
YER036C	YHR169W	6.3054
YKL018W	YLR028C	6.3053
YBL003C	YJL115W	6.305
YER122C	YIL076W	6.3029
YJR084W	YMR288W	6.3015
YHR197W	YPR016C	6.3015
YHR147C	YNL005C	6.3001
YGR090W	YLR223C	6.299
YPL055C	YPR191W	6.2986
YLR356W	YPR191W	6.2986
YLR132C	YPR191W	6.2986
YDL074C	YPR191W	6.2986
YBL045C	YPL055C	6.2986
YBL045C	YLR356W	6.2986
YBL045C	YLR132C	6.2986
YBL045C	YDL074C	6.2986
YML010W	YOR210W	6.2981
YBR142W	YDL148C	6.2959
YMR225C	YNL252C	6.295
YML009C	YNL252C	6.295
YBR247C	YOR056C	6.295
YML073C	YNL069C	6.294
YLR258W	YPL137C	6.2939
YKL166C	YNL093W	6.2939
YAR042W	YEL052W	6.2939
YLR424W	YPR082C	6.293
YBR088C	YDR097C	6.2917
YLR055C	YMR227C	6.2915
YLR074C	YNL110C	6.291
YKR060W	YLL011W	6.291
YGR054W	YLR264W	6.2905
YBR080C	YKL145W	6.2904
YDL002C	YJR065C	6.29
YMR258C	YOR370C	6.2891
YKL057C	YKL186C	6.2891
YDR516C	YKL119C	6.2891
YDR383C	YIR010W	6.2891
YBR265W	YMR251W	6.2891
YBR265W	YLR411W	6.2891
YBR265W	YIR032C	6.2891
YBR265W	YIL177C	6.2891
YBR200W	YER114C	6.2891
YBL061C	YNL230C	6.2891
YBL061C	YGR185C	6.2891
YBL023C	YMR251W	6.2891
YBL023C	YLR411W	6.2891
YBL023C	YIR032C	6.2891
YBL023C	YIL177C	6.2891
YAL026C	YML072C	6.2891
YMR181C	YNL290W	6.289
YBR214W	YJL098W	6.289
YKR081C	YLR406C	6.2884
YDR424C	YIL115C	6.2873
YAR014C	YGR092W	6.2872
YHR161C	YLR106C	6.285
YGR284C	YML130C	6.285
YCR063W	YLR016C	6.2849
YDR381W	YDR507C	6.2834
YBR055C	YDL087C	6.2832
YDR359C	YPL169C	6.2828
YBR251W	YJR096W	6.2823
YEL037C	YIL103W	6.2816
YDR087C	YOR272W	6.2802
YOR204W	YPL198W	6.2799
YER103W	YGL207W	6.2785
YFL034C-A	YPR061C	6.2778
YCR036W	YFL034C-A	6.2778
YAR007C	YOR304W	6.2778
YGL221C	YGR178C	6.2773
YGR090W	YOR312C	6.2758
YDL005C	YOL135C	6.2757
YER146W	YNL147W	6.2756
YGL019W	YLR197W	6.2747
YBR122C	YBR282W	6.2738
YER062C	YER171W	6.2727
YBL074C	YLR275W	6.2725
YBR048W	YIL069C	6.2723
YKL172W	YLR276C	6.2722
YDR296W	YMR098C	6.2699
YDR116C	YMR098C	6.2699
YLR345W	YOR219C	6.2693
YBR107C	YKR054C	6.2693
YIL070C	YOR326W	6.2689
YOR261C	YPR103W	6.2684
YKR048C	YMR308C	6.2683
YBR084W	YGR285C	6.2683
YBR170C	YHR092C	6.2679
YER102W	YPL081W	6.2674
YLR249W	YPL240C	6.2664
YML088W	YOR057W	6.2655
YLR267W	YML088W	6.2655
YBL016W	YGL158W	6.2655
YHR023W	YMR109W	6.2649
YBR055C	YDL043C	6.2649
YER018C	YJR032W	6.2648
YLR275W	YPR101W	6.2647
YFR052W	YML057W	6.2642
YGR145W	YLR129W	6.2616
YNL212W	YOR281C	6.261
YHR121W	YLL013C	6.2609
YIL061C	YPR191W	6.2567
YDR498C	YNL258C	6.2563
YDR498C	YLR440C	6.2563
YDR060W	YDR087C	6.2542
YBR072W	YPR184W	6.254
YDR375C	YLR355C	6.2539
YNL024C	YOR093C	6.2537
YBL008W	YJR070C	6.2537
YDR432W	YML117W	6.253
YFL005W	YOR089C	6.2527
YDL065C	YNL147W	6.2522
YOR150W	YPL183W-A	6.2521
YDR334W	YGL150C	6.252
YER069W	YGR006W	6.2516
YDR283C	YMR255W	6.2516
YML025C	YPR100W	6.2509
YHR199C	YMR308C	6.2504
YFR028C	YPR110C	6.2504
YML072C	YOR303W	6.2498
YLR422W	YOR042W	6.2498
YJR016C	YPR165W	6.2498
YJL045W	YOR042W	6.2498
YJL045W	YLR422W	6.2498
YIL137C	YJL154C	6.2498
YHR088W	YJL154C	6.2498
YHR088W	YIL137C	6.2498
YFR044C	YPR165W	6.2498
YFR044C	YJR016C	6.2498
YFR024C-A	YOR042W	6.2498
YFR024C-A	YLR422W	6.2498
YFR024C-A	YJL045W	6.2498
YER162C	YMR201C	6.2498
YER057C	YMR276W	6.2498
YDR168W	YKL139W	6.2498
YDR049W	YMR276W	6.2498
YDR049W	YER057C	6.2498
YDL235C	YJL154C	6.2498
YDL235C	YIL137C	6.2498
YDL235C	YHR088W	6.2498
YBR242W	YJL154C	6.2498
YBR242W	YIL137C	6.2498
YBR242W	YHR088W	6.2498
YBR242W	YDL235C	6.2498
YBR170C	YMR276W	6.2498
YBR170C	YER057C	6.2498
YFL034W	YKL103C	6.2497
YER027C	YOR276W	6.2497
YAR073W	YML056C	6.2465
YLR075W	YLR441C	6.2462
YJL074C	YMR001C	6.2458
YAR019C	YPL218W	6.2456
YAR019C	YJR053W	6.2456
YOL010W	YPL266W	6.2446
YKR001C	YOL006C	6.2443
YDR517W	YOR157C	6.2434
YDR118W	YFR036W	6.2429
YBR215W	YGL113W	6.2428
YLR005W	YNL135C	6.2426
YDR311W	YNL135C	6.2426
YAL059W	YBR115C	6.2426
YDR142C	YDR188W	6.2422
YCR079W	YDL140C	6.2409
YNL101W	YNL187W	6.2404
YGR159C	YHR132C	6.2404
YDR452W	YHR132C	6.2404
YDR452W	YGR159C	6.2404
YBL023C	YBR265W	6.2404
YAR073W	YHR132C	6.2404
YAR073W	YGR159C	6.2404
YAR073W	YDR452W	6.2404
YLR079W	YPL014W	6.2403
YLR079W	YLR144C	6.2403
YKR091W	YLR079W	6.2403
YGR202C	YPR023C	6.2391
YFL023W	YLR284C	6.2385
YER174C	YNL254C	6.2385
YGR167W	YNL084C	6.2381
YML063W	YOR123C	6.2378
YCR009C	YER125W	6.2376
YBR251W	YDR494W	6.2355
YGR027C	YOL127W	6.2353
YDL030W	YDR235W	6.2353
YFR019W	YGL173C	6.2347
YNL090W	YNL210W	6.2342
YGL097W	YMR235C	6.2342
YDL006W	YLR352W	6.2342
YBR088C	YOR378W	6.2342
YBR088C	YLR276C	6.2342
YBR088C	YDL164C	6.2342
YJR084W	YLR382C	6.234
YLR215C	YPR041W	6.2338
YKR024C	YLR175W	6.2337
YJL053W	YOR132W	6.232
YDL097C	YOL022C	6.2318
YDL097C	YLR341W	6.2318
YDL097C	YGL027C	6.2318
YDL097C	YER041W	6.2318
YCL014W	YCL054W	6.2317
YDR473C	YIL061C	6.2314
YJL076W	YJL148W	6.2311
YCR071C	YGR220C	6.2311
YDR087C	YNL061W	6.231
YCR046C	YMR098C	6.2308
YLL024C	YPR179C	6.2301
YKR085C	YPL183W-A	6.2291
YER074W	YIL069C	6.2285
YNL025C	YNL236W	6.2283
YMR313C	YOR014W	6.2256
YDR447C	YJL177W	6.2246
YDL111C	YNL151C	6.2237
YBL006C	YLR033W	6.2235
YDR087C	YGR103W	6.2231
YJR145C	YLR424W	6.2217
YDR432W	YGR162W	6.2217
YBR245C	YKR008W	6.2217
YHR085W	YKR081C	6.2204
YFR011C	YJR063W	6.219
YFR008W	YMR029C	6.219
YDR200C	YMR029C	6.219
YAR007C	YMR116C	6.219
YGR270W	YIL055C	6.2189
YBL088C	YPL110C	6.2182
YCL009C	YFL045C	6.218
YBL038W	YPL183W-A	6.2178
YLR186W	YLR197W	6.2166
YJR051W	YOR157C	6.2165
YBR025C	YDR394W	6.2164
YGL048C	YMR314W	6.2163
YIL035C	YMR049C	6.2128
YER148W	YKR001C	6.2122
YNL265C	YPR173C	6.2108
YNL091W	YNL164C	6.2108
YBL072C	YMR309C	6.2095
YDR398W	YPL160W	6.2091
YOL017W	YOR046C	6.2087
YDL051W	YOR287C	6.2087
YDR324C	YKR060W	6.2082
YAR042W	YIL074C	6.2069
YHL027W	YMR234W	6.2068
YDR087C	YMR049C	6.2067
YGR185C	YMR158W	6.2066
YKL081W	YMR318C	6.206
YER127W	YLL045C	6.2059
YER146W	YFL017W-A	6.205
YHL015W	YMR230W	6.2035
YDR060W	YPL211W	6.2028
YML117W	YMR125W	6.2014
YNR036C	YNR037C	6.2003
YGR240C	YOL013C	6.2003
YHR087W	YIL162W	6.2002
YDR166C	YPR114W	6.2002
YIL142W	YJR072C	6.1993
YKL110C	YLR384C	6.1982
YER129W	YGL115W	6.1982
YDR311W	YER171W	6.1982
YDL031W	YNL110C	6.1971
YDR052C	YKL145W	6.1958
YCL054W	YDL105W	6.1957
YGL048C	YOL022C	6.1956
YGL048C	YLR341W	6.1956
YGL027C	YGL048C	6.1956
YER041W	YGL048C	6.1956
YBR118W	YER156C	6.1955
YDR337W	YKL003C	6.1946
YKL142W	YKR066C	6.1939
YDL215C	YKL142W	6.1939
YAL041W	YBR200W	6.1939
YIL126W	YOR116C	6.1928
YJL063C	YMR098C	6.1925
YDL209C	YJR050W	6.1925
YCL014W	YJL063C	6.1925
YBR189W	YBR247C	6.1918
YGR192C	YNL271C	6.1916
YFR037C	YNL030W	6.1904
YDR186C	YGL115W	6.1895
YBR125C	YGL115W	6.1895
YDR224C	YGR056W	6.1891
YDR170C	YMR086W	6.1891
YLR258W	YOR026W	6.1889
YER059W	YPL031C	6.1884
YCR008W	YPL031C	6.1884
YNL055C	YNL085W	6.188
YDR412W	YLR449W	6.188
YDR415C	YHR082C	6.1879
YDR499W	YHR164C	6.1874
YLR129W	YPL266W	6.1852
YGR020C	YMR027W	6.1838
YGR020C	YLR090W	6.1838
YDR405W	YKR085C	6.1833
YGR240C	YJL080C	6.1827
YBL072C	YPR041W	6.1817
YER082C	YMR128W	6.1807
YOR252W	YPR163C	6.1806
YJR121W	YOL086C	6.1804
YBL006C	YFR037C	6.1802
YAL012W	YIL127C	6.1797
YBR127C	YJL026W	6.1785
YKL130C	YOR326W	6.1769
YBR130C	YHR023W	6.1764
YMR229C	YNL132W	6.1763
YDR388W	YML123C	6.1758
YDR324C	YOL010W	6.1756
YDR288W	YLR383W	6.1756
YJR123W	YNL178W	6.1752
YBL052C	YEL018W	6.1746
YOR319W	YPR188C	6.1745
YER164W	YOR319W	6.1745
YDR143C	YLR229C	6.1745
YBR140C	YNL098C	6.1745
YGR090W	YKR060W	6.174
YBR212W	YNL135C	6.1736
YAL054C	YLR153C	6.1736
YDR025W	YIL069C	6.1734
YDR507C	YJR076C	6.1729
YGR103W	YHR085W	6.172
YLR025W	YNL265C	6.1716
YMR191W	YPR108W	6.1703
YCL054W	YEL019C	6.1703
YHR027C	YJL008C	6.1699
YKL122C	YLR432W	6.1688
YIL122W	YLR044C	6.1684
YNL071W	YOR132W	6.1682
YER118C	YNL071W	6.1682
YGR198W	YLR222C	6.1674
YDL019C	YHL020C	6.1662
YDL019C	YER120W	6.1662
YBR044C	YGR193C	6.1662
YBR263W	YNL112W	6.1659
YJL063C	YLR189C	6.1654
YJL026W	YOR229W	6.1654
YDL209C	YHR156C	6.1654
YDR207C	YER088C	6.1641
YBL061C	YDL044C	6.1641
YFL024C	YOR046C	6.1639
YDR201W	YDR320C-A	6.1635
YDR299W	YJR002W	6.1622
YDL132W	YPR164W	6.1618
YDL030W	YDR240C	6.1615
YGL158W	YLR113W	6.1602
YGL141W	YKL021C	6.1602
YDR324C	YOL108C	6.1602
YDR300C	YPR137W	6.1602
YDL116W	YPL215W	6.1602
YDR099W	YGR097W	6.1598
YDR424C	YJL041W	6.1595
YPL026C	YPL028W	6.1594
YGR223C	YPL028W	6.1594
YDR224C	YGL133W	6.1586
YIL107C	YOR261C	6.1574
YDR170C	YGL099W	6.1573
YDL066W	YNR051C	6.1557
YBL074C	YPL213W	6.1546
YEL002C	YML019W	6.1536
YLR117C	YMR125W	6.1522
YBR122C	YGR220C	6.152
YKR064W	YOR249C	6.1516
YFR028C	YMR173W	6.1516
YDR346C	YLR186W	6.1516
YDR346C	YKL143W	6.1516
YDR001C	YER016W	6.1516
YJR090C	YOL133W	6.1515
YJR090C	YOL063C	6.1515
YDR023W	YDR328C	6.1515
YBR245C	YNL030W	6.1513
YNL252C	YPL183W-A	6.1508
YER112W	YJL203W	6.1494
YDR041W	YOR243C	6.1476
YDR194C	YDR496C	6.1475
YBL037W	YJR058C	6.1474
YDR449C	YER127W	6.1469
YLR430W	YMR125W	6.146
YOR304W	YPL235W	6.1451
YDL126C	YMR076C	6.145
YBR245C	YMR033W	6.1447
YGR130C	YKR018C	6.1433
YDL014W	YOL144W	6.1421
YGL120C	YLL045C	6.1403
YJR032W	YOL069W	6.1398
YBL090W	YJL063C	6.1398
YDR416W	YGR013W	6.1395
YDL225W	YNL233W	6.1391
YCR052W	YJL176C	6.1381
YJL177W	YKL180W	6.1377
YDL160C	YKL173W	6.1375
YAL003W	YFR053C	6.1375
YDR073W	YJL176C	6.1361
YDR171W	YNL007C	6.1356
YGR005C	YHR077C	6.1354
YDR091C	YNL096C	6.1352
YDL047W	YDL219W	6.135
YMR106C	YMR287C	6.1349
YJL137C	YLR033W	6.1335
YBR198C	YPL047W	6.1329
YDR234W	YLL026W	6.1326
YKL011C	YMR201C	6.1321
YJR042W	YLR359W	6.1321
YFL009W	YML088W	6.1321
YER162C	YKL011C	6.1321
YEL037C	YMR276W	6.1321
YEL037C	YER057C	6.1321
YDR049W	YEL037C	6.1321
YDL190C	YMR276W	6.1321
YDL190C	YER057C	6.1321
YDL190C	YDR049W	6.1321
YBR170C	YEL037C	6.1321
YBR170C	YDL190C	6.1321
YDR131C	YIL063C	6.132
YCR073C	YLR233C	6.132
YLR354C	YNL053W	6.1319
YJL068C	YLR354C	6.1319
YHR186C	YKL119C	6.1319
YDR324C	YOL086C	6.1319
YKL184W	YOR259C	6.1314
YBR009C	YGL133W	6.1308
YLR312W-A	YNL177C	6.1292
YHR146W	YMR297W	6.1278
YAR042W	YLR305C	6.127
YBR127C	YGR092W	6.1269
YJL008C	YJR065C	6.1264
YBR279W	YOL006C	6.1259
YLR199C	YML119W	6.1242
YKR054C	YLR381W	6.1242
YDR490C	YPR140W	6.1242
YKL099C	YKR060W	6.1237
YGL234W	YOR217W	6.1216
YOR335C	YPR010C	6.1214
YJL066C	YML085C	6.1213
YHR103W	YLR384C	6.1204
YAL020C	YLR384C	6.1204
YKR024C	YLR002C	6.1203
YKL014C	YPL043W	6.12
YBL004W	YLR129W	6.1194
YNL248C	YOR207C	6.1183
YDR214W	YMR282C	6.1165
YBR034C	YDR383C	6.1165
YER029C	YKR022C	6.1158
YBR231C	YML041C	6.1154
YCL054W	YPR169W	6.1151
YER029C	YJL124C	6.115
YMR197C	YOR212W	6.1149
YDR264C	YMR197C	6.1149
YLR197W	YOR145C	6.1146
YBR055C	YLR382C	6.1143
YML085C	YNL064C	6.1123
YBR025C	YDL097C	6.1119
YDR475C	YKL193C	6.1108
YMR191W	YOR261C	6.1098
YFL037W	YJR121W	6.1093
YML025C	YMR098C	6.1091
YCL014W	YML025C	6.1091
YDL148C	YNL308C	6.1086
YDR032C	YNL010W	6.107
YBL056W	YBR097W	6.107
YOR347C	YOR370C	6.1069
YJR014W	YKL028W	6.1069
YGL242C	YKL057C	6.1069
YER007C-A	YKL028W	6.1069
YBR177C	YKL057C	6.1069
YDR496C	YPL211W	6.1065
YLR069C	YMR313C	6.1051
YJR041C	YPL178W	6.1051
YJL197W	YOR019W	6.1051
YER098W	YOL087C	6.1051
YDR405W	YNL252C	6.1051
YDR283C	YOR243C	6.1051
YJR101W	YOR243C	6.1048
YMR128W	YMR229C	6.1043
YDR300C	YPL001W	6.1037
YJR050W	YKL173W	6.1035
YDR021W	YLR023C	6.1032
YDR453C	YMR158W	6.1013
YLR153C	YPL226W	6.1011
YOR312C	YPL220W	6.0997
YNL061W	YOL041C	6.0995
YDR073W	YOR290C	6.0981
YKR081C	YNL061W	6.0979
YDL185W	YMR205C	6.0964
YCR063W	YOR159C	6.0961
YKR067W	YNL287W	6.0958
YHR164C	YIR002C	6.0951
YDR097C	YHR164C	6.0951
YDL116W	YPL169C	6.0951
YMR181C	YOL094C	6.095
YPL106C	YPR035W	6.0947
YHR023W	YKL130C	6.0919
YFR001W	YKL009W	6.0919
YGL174W	YGL216W	6.0913
YFR037C	YJL137C	6.0902
YCR073W-A	YPL137C	6.0893
YER146W	YER172C	6.0889
YHR099W	YJR082C	6.0873
YDR296W	YMR225C	6.0871
YDR296W	YML009C	6.0871
YDR116C	YMR225C	6.0871
YDR116C	YML009C	6.0871
YGL133W	YOR116C	6.0846
YGR202C	YLR392C	6.0827
YLR189C	YML025C	6.0819
YNL096C	YNL302C	6.0812
YJR077C	YLR342W	6.0806
YER127W	YLR409C	6.0797
YDL175C	YIL075C	6.0797
YBL072C	YDL148C	6.0787
YDR037W	YDR129C	6.0783
YGL090W	YOR005C	6.078
YBR143C	YLR092W	6.078
YBR109C	YGR263C	6.0777
YBR109C	YFR014C	6.0777
YLR075W	YPR080W	6.077
YGR130C	YMR086W	6.0768
YGR184C	YLR109W	6.0762
YMR251W	YNL076W	6.0757
YLR411W	YNL076W	6.0757
YIR032C	YNL076W	6.0757
YIL177C	YNL076W	6.0757
YGR148C	YKL085W	6.0757
YFL052W	YKL085W	6.0757
YDR049W	YGR048W	6.0757
YCR030C	YOL016C	6.0756
YER082C	YGR145W	6.0755
YMR310C	YOR219C	6.0753
YBR085W	YIL053W	6.0752
YDR448W	YGR104C	6.0749
YDR427W	YJL008C	6.072
YGR270W	YKL088W	6.0719
YJL098W	YLR179C	6.0716
YCR082W	YKL052C	6.0716
YCR003W	YNL254C	6.0716
YDR320C-A	YGL061C	6.0715
YHR197W	YOL077C	6.0704
YGL008C	YNL055C	6.0692
YDR385W	YGL137W	6.0689
YGL004C	YOR117W	6.068
YMR213W	YOR159C	6.0651
YNL103W	YPL038W	6.065
YMR190C	YPL029W	6.065
YGL158W	YOR018W	6.065
YDR139C	YNL103W	6.065
YBR175W	YPL029W	6.065
YKL075C	YNL091W	6.0643
YER090W	YOR064C	6.0643
YDL126C	YML013W	6.0638
YER165W	YGR250C	6.0634
YPL064C	YPR101W	6.0625
YHR114W	YJL020C	6.0625
YEL018W	YPR031W	6.0618
YAL038W	YDL185W	6.0613
YBR247C	YDR346C	6.0607
YDR409W	YLL029W	6.0606
YBL072C	YDR429C	6.0602
YKL060C	YMR113W	6.06
YJL117W	YMR134W	6.0586
YHR117W	YLR072W	6.0586
YDR171W	YGL106W	6.058
YBL092W	YDL083C	6.0574
YIL069C	YOR312C	6.0571
YGL173C	YLR147C	6.0568
YDR507C	YHR107C	6.0555
YLR243W	YML123C	6.0554
YJR139C	YML123C	6.0554
YIL156W	YML123C	6.0554
YHR039C-A	YPL201C	6.0553
YPL014W	YPL256C	6.0548
YLR144C	YPL256C	6.0548
YKR091W	YPL256C	6.0548
YDR341C	YHR064C	6.0547
YGR086C	YHL020C	6.053
YER120W	YGR086C	6.053
YLR439W	YPR100W	6.0525
YLR039C	YMR235C	6.0521
YDR142C	YLR105C	6.0521
YGR087C	YLR134W	6.0516
YFR048W	YHR174W	6.0504
YBR079C	YMR172W	6.0504
YDL073W	YLR200W	6.0499
YDL007W	YEL037C	6.049
YML025C	YNR022C	6.0481
YCR046C	YMR225C	6.0481
YCR046C	YML009C	6.0481
YGR264C	YMR125W	6.0479
YOL041C	YOR206W	6.0466
YBL003C	YFR013W	6.0466
YMR323W	YNL172W	6.0463
YKL089W	YMR117C	6.0463
YIR010W	YKL089W	6.0463
YGL124C	YML007W	6.0463
YFR036W	YNL172W	6.0463
YER133W	YML074C	6.0463
YDR424C	YJL061W	6.0463
YDR422C	YEL023C	6.0463
YAL002W	YOR196C	6.0463
YDL007W	YDR314C	6.0458
YAL038W	YFL049W	6.0457
YDR225W	YLL019C	6.0449
YML056C	YPL208W	6.0441
YDR361C	YML056C	6.0441
YNL112W	YOR312C	6.0436
YBL087C	YPL093W	6.0433
YBR095C	YIL035C	6.0402
YDR471W	YJL177W	6.0401
YMR307W	YOR270C	6.0398
YDR320C-A	YKR037C	6.0391
YNL096C	YPR041W	6.0382
YJL111W	YNL212W	6.0381
YGR091W	YJR084W	6.0365
YGR013W	YLR424W	6.0362
YBR048W	YBR189W	6.0361
YDR025W	YER102W	6.0352
YOR159C	YPL213W	6.0345
YBL026W	YOR159C	6.0345
YJR070C	YOR038C	6.0338
YGL050W	YJR031C	6.0338
YGR040W	YLR362W	6.0335
YJL203W	YJR050W	6.0332
YDR145W	YPL047W	6.0331
YIL035C	YPL139C	6.0317
YBR282W	YKR085C	6.0312
YCR012W	YOR133W	6.0308
YER178W	YLR085C	6.0304
YHR148W	YPL217C	6.0296
YER086W	YLR259C	6.0296
YDL045W-A	YJL063C	6.0287
YDR195W	YMR061W	6.0286
YGL004C	YGR232W	6.0281
YKL009W	YKR081C	6.0279
YDR060W	YKR081C	6.0279
YCR030C	YML069W	6.0278
YLR074C	YPL013C	6.0273
YDR036C	YLR074C	6.0273
YDR238C	YKR067W	6.027
YDR150W	YLR305C	6.027
YBR265W	YNL076W	6.027
YBL023C	YNL076W	6.027
YMR302C	YOR042W	6.0268
YLR422W	YMR302C	6.0268
YJL045W	YMR302C	6.0268
YIL075C	YNL047C	6.0268
YGL179C	YMR092C	6.0268
YFR024C-A	YMR302C	6.0268
YEL071W	YPR165W	6.0268
YEL071W	YJR016C	6.0268
YEL071W	YFR044C	6.0268
YDR260C	YOR303W	6.0268
YDR260C	YML072C	6.0268
YDR243C	YOR303W	6.0268
YDR243C	YML072C	6.0268
YBR152W	YER172C	6.0266
YIL113W	YNL138W	6.0265
YBL056W	YDR076W	6.0265
YBR109C	YCR088W	6.025
YJL138C	YNL201C	6.0239
YLR129W	YML093W	6.0235
YLR429W	YMR262W	6.0232
YMR125W	YPL213W	6.022
YDR190C	YGL252C	6.022
YFL044C	YJL194W	6.0215
YOL040C	YOR096W	6.0214
YER029C	YKL188C	6.0213
YDL014W	YOR204W	6.0213
YOR176W	YOR259C	6.0207
YKR022C	YLR275W	6.02
YBL038W	YBR282W	6.0198
YGR208W	YJL076W	6.0197
YAL003W	YMR187C	6.0197
YHL025W	YPL129W	6.0189
YEL044W	YNL059C	6.0182
YDR538W	YLR436C	6.0182
YDL066W	YLR436C	6.0182
YDL066W	YDR538W	6.0182
YDR023W	YOL088C	6.0181
YOL018C	YOR117W	6.0178
YGL048C	YOL018C	6.0178
YGR240C	YPL193W	6.0168
YGR240C	YOR286W	6.0168
YBR211C	YPL233W	6.0161
YKL060C	YKR059W	6.0146
YLR226W	YPR120C	6.0144
YKL045W	YNL192W	6.0144
YHR082C	YNL192W	6.0144
YHR082C	YKL045W	6.0144
YHR001W	YLR206W	6.0144
YGR002C	YMR019W	6.0144
YER067W	YHR087W	6.0144
YER015W	YMR019W	6.0144
YER015W	YGR002C	6.0144
YDR356W	YOR035C	6.0144
YDR356W	YKL129C	6.0144
YDR150W	YDR416W	6.0144
YDR146C	YMR019W	6.0144
YDR146C	YGR002C	6.0144
YDR146C	YER015W	6.0144
YDR074W	YHR196W	6.0144
YDL155W	YPR120C	6.0144
YDL155W	YLR226W	6.0144
YBR179C	YPL226W	6.0144
YBL066C	YDR416W	6.0144
YBL066C	YDR150W	6.0144
YBL061C	YDR416W	6.0144
YBL061C	YDR150W	6.0144
YBL061C	YBL066C	6.0144
YBL029W	YLR206W	6.0144
YBL029W	YHR001W	6.0144
YAL047C	YDR229W	6.0144
YAL021C	YDL165W	6.0144
YDR496C	YER006W	6.0135
YDR311W	YML063W	6.013
YBR017C	YPL081W	6.0115
YJR112W	YKL089W	6.0113
YIL161W	YNL135C	6.0113
YGR251W	YNL186W	6.0113
YGR251W	YKL078W	6.0113
YBL061C	YGL073W	6.0112
YMR020W	YNL246W	6.0111
YBR061C	YML069W	6.0105
YJL076W	YPL110C	6.0095
YKL145W	YLR180W	6.0081
YGL240W	YKL022C	6.0053
YDR260C	YKL022C	6.0053
YCR079W	YGR005C	6.0052
YCR079W	YDL115C	6.0052
YBL003C	YOL012C	6.0052
YCL054W	YPL217C	6.0039
YDR202C	YPR036W	6.0035
YJL033W	YOR310C	6.0033
YIL075C	YNL115C	6.0032
YDR152W	YKL052C	6.0027
YCR003W	YNL005C	6.0027
YKL007W	YPL106C	6.0023
YIL128W	YMR178W	6.0017
YAL010C	YMR203W	6.0017
YDL075W	YNL302C	6.0006
YDL136W	YPL143W	6.0004
YFL002C	YGR103W	5.9992
YJR045C	YKR048C	5.9989
YDR101C	YPL211W	5.9977
YLR027C	YNL172W	5.9976
YMR093W	YOL010W	5.9965
YER025W	YNL244C	5.9965
YDR449C	YOL010W	5.9965
YOR145C	YPL012W	5.9964
YNL251C	YOR195W	5.9959
YIL159W	YNL251C	5.9959
YER069W	YPR046W	5.9959
YLR175W	YLR196W	5.9955
YBR169C	YOR007C	5.9945
YBR137W	YBR169C	5.9945
YKR060W	YMR093W	5.9941
YDL175C	YMR125W	5.994
YER146W	YNL118C	5.9928
YLR059C	YMR224C	5.9925
YPL022W	YPR135W	5.9923
YDR224C	YMR284W	5.9918
YFR024C-A	YOR039W	5.9916
YHR167W	YLR432W	5.9915
YKL081W	YPR080W	5.9913
YBR017C	YDR502C	5.9908
YGR103W	YLR074C	5.9905
YDL043C	YDL098C	5.9889
YDR462W	YPR100W	5.988
YLL034C	YPL043W	5.9875
YGR180C	YJR140C	5.9875
YGR180C	YJL115W	5.9875
YER030W	YGR116W	5.9875
YCR093W	YER068W	5.9873
YDR087C	YKL009W	5.987
YGR071C	YLL024C	5.9865
YBR181C	YJL177W	5.9865
YDR473C	YPR057W	5.9859
YBR142W	YPL043W	5.9857
YCL054W	YLL008W	5.9851
YLR129W	YPR112C	5.9834
YBR084W	YPR189W	5.9825
YDL136W	YDR447C	5.982
YJR064W	YOR187W	5.9805
YDR202C	YGR020C	5.9805
YGL120C	YNL224C	5.9798
YMR259C	YOR259C	5.9795
YNL161W	YOR007C	5.9781
YMR190C	YOL045W	5.9781
YIL129C	YNL161W	5.9781
YGR016W	YMR104C	5.9781
YEL023C	YGR016W	5.9781
YDL116W	YLR359W	5.9781
YBR175W	YOL045W	5.9781
YBL080C	YGR016W	5.9781
YJR053W	YNL238W	5.9779
YDR098C	YGL220W	5.9779
YFL024C	YML007W	5.9776
YLR439W	YPL173W	5.9774
YGR145W	YOR063W	5.9772
YCR033W	YDR155C	5.9772
YFR031C-A	YNL301C	5.9766
YBR049C	YPL082C	5.976
YBR127C	YDR289C	5.9759
YJL177W	YMR242C	5.9757
YLR264W	YNL096C	5.9753
YDR295C	YOR323C	5.9753
YPL266W	YPR144C	5.975
YBL072C	YBR048W	5.975
YBR189W	YPL266W	5.9746
YKL173W	YKR022C	5.9745
YNR047W	YOR220W	5.9741
YLR256W	YNR047W	5.9741
YGL181W	YNR047W	5.9741
YBR059C	YNR047W	5.9741
YKL198C	YLR082C	5.9736
YIL147C	YLR082C	5.9736
YIL147C	YKL198C	5.9736
YHR015W	YLR344W	5.9736
YER164W	YGR270W	5.9736
YBR052C	YNL010W	5.9736
YCR052W	YDR224C	5.9718
YGL133W	YOR110W	5.9713
YER087W	YMR239C	5.9701
YLR342W	YML067C	5.9698
YAL042W	YLR342W	5.9698
YML062C	YNL230C	5.969
YMR257C	YMR309C	5.9686
YIR026C	YMR309C	5.9686
YJR093C	YMR061W	5.9685
YDR073W	YMR033W	5.9685
YLR430W	YNL116W	5.9678
YBR247C	YML091C	5.9676
YER089C	YOR230W	5.9667
YER054C	YPR184W	5.9662
YCR084C	YLR313C	5.9662
YCR084C	YJL156C	5.9662
YDR448W	YLR071C	5.9658
YLL034C	YOR206W	5.9653
YBR154C	YDR156W	5.965
YHR027C	YMR314W	5.9648
YCR072C	YKR081C	5.9644
YJL076W	YPR010C	5.9627
YMR314W	YOR117W	5.9625
YGR155W	YJR045C	5.9623
YJL034W	YPL048W	5.962
YDR299W	YPL126W	5.9616
YLR409C	YPR144C	5.9611
YHR114W	YMR241W	5.9604
YBL007C	YMR241W	5.9604
YKL172W	YLR449W	5.9587
YKL040C	YLL027W	5.9576
YJL033W	YMR290C	5.9567
YDR322W	YPR100W	5.9566
YOR243C	YPL118W	5.9555
YNL306W	YOR243C	5.9555
YHL004W	YOR243C	5.9555
YBR146W	YOR243C	5.9555
YIR010W	YJR135C	5.953
YER095W	YLR450W	5.953
YOL090W	YPR065W	5.9529
YDL063C	YNL035C	5.9527
YCR031C	YGR034W	5.9524
YFR009W	YJR121W	5.9521
YHR060W	YNR050C	5.9513
YLR424W	YMR288W	5.9492
YLR192C	YNL079C	5.9475
YJL069C	YNL075W	5.9474
YMR125W	YMR240C	5.9467
YDR153C	YKL023W	5.9458
YFL039C	YHR015W	5.9454
YCR002C	YDR507C	5.945
YIR007W	YMR323W	5.9447
YDR101C	YGL103W	5.9445
YDR450W	YPL143W	5.9436
YDR296W	YPL183W-A	5.9433
YDR116C	YPL183W-A	5.9433
YCR073W-A	YLR258W	5.9429
YNR058W	YOR172W	5.9428
YBR120C	YGR282C	5.9427
YBL030C	YBR120C	5.9427
YER021W	YKL184W	5.9424
YHR059W	YHR197W	5.9423
YBR264C	YDL097C	5.9416
YBR083W	YGR040W	5.9416
YFL037W	YGR155W	5.9413
YBL045C	YOR299W	5.9408
YKR035W-A	YOR144C	5.9383
YLR275W	YNL286W	5.9377
YHR052W	YLR276C	5.937
YFR004W	YNL115C	5.9368
YFL017W-A	YLR275W	5.9363
YMR267W	YPL066W	5.9362
YLR425W	YMR267W	5.9362
YDL203C	YMR267W	5.9362
YGR254W	YMR014W	5.9361
YER091C	YKR048C	5.9357
YBR055C	YGL128C	5.9343
YBR143C	YKL060C	5.9341
YDR496C	YLL034C	5.9336
YGL251C	YMR083W	5.9334
YBR062C	YMR083W	5.9334
YMR251W	YMR261C	5.9328
YLR411W	YMR261C	5.9328
YIR032C	YMR261C	5.9328
YIL177C	YMR261C	5.9328
YER013W	YNR011C	5.9328
YDR379W	YNL201C	5.9328
YHR119W	YPL029W	5.9316
YGR145W	YHR148W	5.9308
YNL075W	YPL061W	5.9303
YKL035W	YPL061W	5.9303
YDR490C	YKL003C	5.9303
YDR490C	YIR029W	5.9303
YCL040W	YPL061W	5.9303
YIL093C	YOR243C	5.9296
YDR369C	YLR059C	5.9295
YGL053W	YJL026W	5.9294
YBL074C	YGR091W	5.929
YJR032W	YPL179W	5.9287
YDR214W	YPR089W	5.9287
YDL108W	YLR005W	5.9287
YAL002W	YOL067C	5.9287
YAL002W	YBL103C	5.9287
YDR172W	YGR090W	5.9286
YBR160W	YPL014W	5.9278
YBR160W	YLR144C	5.9278
YBR160W	YKR091W	5.9278
YBR135W	YPL014W	5.9278
YBR135W	YLR144C	5.9278
YBR135W	YKR091W	5.9278
YKL092C	YKR066C	5.9245
YDL215C	YKL092C	5.9245
YDL126C	YNL031C	5.9241
YGR234W	YLR304C	5.9222
YFR001W	YOR272W	5.9221
YNL175C	YPL043W	5.9216
YFL049W	YPL129W	5.9213
YDR087C	YLR002C	5.9199
YJR050W	YMR240C	5.9196
YCR009C	YOL031C	5.9196
YML117W	YPL184C	5.9194
YAL051W	YDR412W	5.9192
YDL063C	YHR089C	5.9191
YIL127C	YJR105W	5.9176
YDL148C	YLR129W	5.9165
YBR160W	YER090W	5.9161
YER029C	YGL174W	5.916
YBL072C	YHR013C	5.916
YGR081C	YKL143W	5.9147
YLR355C	YPL249C	5.9137
YGR258C	YLR384C	5.913
YGR240C	YPL160W	5.913
YFR036W	YLR127C	5.913
YML046W	YPR082C	5.9127
YLR439W	YMR098C	5.911
YCL014W	YLR439W	5.911
YDR037W	YER003C	5.9101
YCR031C	YER074W	5.9098
YGL135W	YOL127W	5.9095
YKR036C	YLR206W	5.9092
YHR001W	YKR036C	5.9092
YBL029W	YKR036C	5.9092
YBL016W	YLR113W	5.9092
YKL010C	YKL189W	5.9091
YDR359C	YLR248W	5.9091
YGL190C	YNL242W	5.909
YAL059W	YDL051W	5.9088
YCR079W	YJL140W	5.9083
YGL128C	YJR145C	5.9073
YLR345W	YML114C	5.9067
YBR215W	YJR140C	5.9067
YJR058C	YNL270C	5.9065
YHR110W	YKL192C	5.9065
YHR099W	YPL082C	5.9065
YDL018C	YKL192C	5.9065
YBL037W	YNL270C	5.9065
YAL010C	YJL066C	5.9065
YCR046C	YPL183W-A	5.9044
YMR255W	YOR046C	5.9036
YJL155C	YMR310C	5.9036
YDR198C	YOR046C	5.9036
YER029C	YMR213W	5.903
YDR496C	YLR002C	5.903
YJL066C	YLR045C	5.9006
YDR516C	YNR050C	5.9001
YDR314C	YER162C	5.9001
YDR314C	YEL037C	5.9001
YLR418C	YLR427W	5.9
YBR152W	YHR165C	5.8998
YCL043C	YGR250C	5.8987
YCR073W-A	YKL193C	5.8985
YBL038W	YGR220C	5.8985
YPL066W	YPR023C	5.8984
YDR127W	YJR109C	5.8979
YLR039C	YNL307C	5.8975
YKL211C	YOR064C	5.8975
YJL008C	YJL159W	5.8974
YBL008W	YGL113W	5.8974
YLL011W	YLR197W	5.897
YIR007W	YLR027C	5.896
YGL194C	YOR028C	5.896
YIL126W	YMR072W	5.8941
YBL087C	YOL077C	5.894
YJL085W	YPR055W	5.8936
YDR310C	YLR082C	5.8936
YDR310C	YKL198C	5.8936
YDR310C	YIL147C	5.8936
YFL045C	YNL169C	5.8933
YLR234W	YMR190C	5.892
YJR144W	YMR190C	5.892
YJR144W	YLR234W	5.892
YIL151C	YKR096W	5.892
YER002W	YOR294W	5.892
YBR058C	YOR204W	5.892
YIL053W	YLR061W	5.8915
YBR065C	YML049C	5.8914
YCR057C	YNL075W	5.8913
YGR090W	YPL012W	5.8906
YDR496C	YHR088W	5.8906
YDR224C	YFR037C	5.8906
YAL048C	YOR284W	5.8889
YDR121W	YOL006C	5.8887
YGR090W	YPR137W	5.8881
YDR272W	YDR397C	5.8881
YLR347C	YOR048C	5.8873
YIL063C	YOL061W	5.8871
YDL171C	YMR117C	5.8871
YDL171C	YER018C	5.8871
YDR224C	YKR008W	5.887
YDL113C	YFR040W	5.8868
YCR057C	YML093W	5.8866
YDR012W	YGR195W	5.8864
YEL002C	YJL002C	5.8857
YEL002C	YGL022W	5.8857
YCR009C	YGR086C	5.8854
YDR385W	YLR044C	5.8848
YLR028C	YOL063C	5.8841
YJL047C	YLR028C	5.8841
YDR328C	YOR370C	5.8841
YBR265W	YMR261C	5.8841
YBL023C	YMR261C	5.8841
YMR203W	YMR241W	5.884
YJL148W	YPR190C	5.884
YLR189C	YLR439W	5.8839
YJL008C	YOR261C	5.8835
YFL039C	YJR082C	5.8829
YKL089W	YPL233W	5.8822
YDR363W	YLL002W	5.8822
YDR152W	YKR083C	5.8822
YAR014C	YOR227W	5.8822
YAR014C	YDR475C	5.8822
YMR229C	YNL061W	5.8816
YDR338C	YNL071W	5.8808
YGR086C	YLR429W	5.8797
YAL005C	YLR257W	5.8796
YHR132W-A	YOR014W	5.8777
YBL086C	YDR289C	5.8777
YDL043C	YGR074W	5.8769
YIL126W	YNL116W	5.8762
YIL055C	YPL115C	5.8762
YER177W	YOR140W	5.8761
YBL087C	YMR290C	5.8752
YDL102W	YIL152W	5.8747
YFL018C	YLR259C	5.8744
YBL022C	YBR083W	5.8737
YGL242C	YJR145C	5.8735
YFL007W	YLR199C	5.8732
YBL026W	YNL118C	5.8728
YIL070C	YKL151C	5.8719
YGR250C	YNL138W	5.8715
YAL017W	YDR044W	5.8715
YBR084W	YNL112W	5.8712
YML063W	YPR080W	5.8704
YMR251W	YPR035W	5.8684
YLR411W	YPR035W	5.8684
YIR032C	YPR035W	5.8684
YIL177C	YPR035W	5.8684
YBR102C	YML097C	5.8683
YER021W	YOR362C	5.8679
YJL063C	YPL183W-A	5.8662
YGL120C	YPR137W	5.8655
YLR131C	YNL254C	5.8642
YDR422C	YNL086W	5.8642
YDR422C	YGL079W	5.8642
YDR422C	YEL005C	5.8642
YDL225W	YLR218C	5.8642
YDL225W	YGR033C	5.8642
YCL054W	YPR016C	5.864
YLR129W	YPR137W	5.8624
YOR323C	YPR179C	5.8621
YNL021W	YOR323C	5.8621
YDR427W	YHR119W	5.8615
YDL087C	YOR159C	5.861
YML007W	YOR244W	5.8602
YDR060W	YGR103W	5.8602
YPL131W	YPR016C	5.8601
YLL048C	YLR259C	5.8601
YFR004W	YMR191W	5.8601
YOL013C	YOR375C	5.8598
YDR507C	YOL012C	5.859
YCL024W	YOL012C	5.859
YCR046C	YDR405W	5.8588
YIR026C	YMR257C	5.8583
YDL164C	YIL046W	5.8583
YGL128C	YLR275W	5.858
YOL022C	YPR108W	5.8577
YLR341W	YPR108W	5.8577
YGL027C	YPR108W	5.8577
YER041W	YPR108W	5.8577
YML063W	YNL112W	5.8565
YBR084W	YDL058W	5.8563
YDL015C	YLR060W	5.856
YBR031W	YDL108W	5.856
YLR452C	YPR088C	5.8559
YLR452C	YPL210C	5.8559
YLR452C	YML105C	5.8559
YDL092W	YLR452C	5.8559
YIL021W	YMR275C	5.8553
YBR120C	YMR145C	5.8553
YLR115W	YOR230W	5.855
YIL070C	YNL189W	5.8548
YDR388W	YHR027C	5.8541
YPL043W	YPL093W	5.8532
YDR202C	YOR332W	5.8529
YJL035C	YKR022C	5.8524
YAL034W-A	YKL089W	5.8524
YBR245C	YGL043W	5.8522
YER112W	YMR304W	5.8515
YKR028W	YNL187W	5.8513
YKR028W	YNL101W	5.8513
YKL119C	YNR050C	5.8513
YNL147W	YPR082C	5.8489
YDR462W	YMR098C	5.8466
YCL014W	YDR462W	5.8466
YGR128C	YLR409C	5.8464
YBL058W	YHR205W	5.8464
YDR101C	YLL034C	5.8462
YLR409C	YOR145C	5.8457
YKL103C	YLR105C	5.8447
YHR119W	YOL045W	5.8447
YCL029C	YKL103C	5.8447
YBR130C	YLR092W	5.8446
YIL069C	YLR290C	5.8436
YFR031C	YIL144W	5.8436
YKL145W	YKL184W	5.8434
YGR128C	YNL178W	5.8427
YBR017C	YER110C	5.8423
YLR340W	YPR016C	5.8403
YFR052W	YNL115C	5.8402
YNR003C	YOR304W	5.8396
YNL050C	YPL031C	5.8393
YFR010W	YLR278C	5.8393
YCR009C	YPL004C	5.8379
YHL032C	YLR305C	5.8378
YDL003W	YLL023C	5.8378
YDR348C	YNL243W	5.8376
YDR388W	YIL156W	5.8375
YDR517W	YLR208W	5.8373
YKL014C	YMR049C	5.8346
YBL071W-A	YEL030W	5.8345
YDR224C	YMR033W	5.8317
YGL103W	YJL177W	5.8315
YBL022C	YDR480W	5.8294
YBR175W	YER062C	5.8292
YOR272W	YPL043W	5.8288
YBR067C	YER151C	5.8288
YKL088W	YKL193C	5.8271
YAL017W	YDR001C	5.8271
YJL069C	YKL099C	5.8265
YMR128W	YPL070W	5.8257
YDL111C	YJL011C	5.8246
YGR282C	YOR259C	5.824
YHR135C	YNL172W	5.8237
YIL152W	YJR043C	5.8235
YDL160C	YPL160W	5.8235
YGL026C	YML085C	5.8234
YLR166C	YML097C	5.8226
YKL012W	YMR288W	5.8226
YCL010C	YMR227C	5.8223
YDR405W	YJL063C	5.8206
YGR002C	YPL066W	5.8199
YGR002C	YOR046C	5.8199
YFR052W	YNL047C	5.8197
YIR010W	YPL018W	5.8196
YDR462W	YLR189C	5.8196
YBR265W	YPR035W	5.8196
YBL023C	YPR035W	5.8196
YGR252W	YPL042C	5.8195
YGL232W	YOR243C	5.8195
YKR001C	YNL030W	5.8194
YBR109C	YDR129C	5.8194
YDR318W	YER069W	5.8192
YJR045C	YKL096W	5.819
YGR254W	YKL060C	5.818
YER091C	YLR133W	5.8178
YAR014C	YMR311C	5.8178
YLR058C	YMR027W	5.8177
YNL123W	YPR047W	5.8175
YDR457W	YLR106C	5.8175
YOR207C	YPR010C	5.8169
YLR382C	YPR178W	5.8158
YGR075C	YJL124C	5.8157
YGL128C	YJR050W	5.8152
YDR322W	YMR098C	5.8152
YCL014W	YDR322W	5.8152
YHR087W	YLR340W	5.814
YGR175C	YHR115C	5.814
YER067W	YLR340W	5.814
YBL016W	YOR018W	5.814
YER174C	YLL029W	5.8137
YER174C	YGL220W	5.8137
YDL203C	YPL066W	5.8137
YDL203C	YLR425W	5.8137
YCR087C-A	YPL022W	5.8134
YHR073W	YPL043W	5.8128
YDL229W	YOL076W	5.8122
YDR141C	YDR335W	5.812
YOR189W	YPL129W	5.8115
YER074W	YNR054C	5.8111
YGR090W	YHR196W	5.8107
YNL085W	YPR010C	5.8106
YBR247C	YOR145C	5.81
YBR189W	YJR145C	5.8092
YKL157W	YLR304C	5.8088
YNL107W	YPL139C	5.8079
YGR148C	YJR092W	5.8078
YFL052W	YJR092W	5.8078
YDL126C	YML029W	5.8076
YJR134C	YML091C	5.8069
YHR098C	YML091C	5.8069
YHR098C	YJR134C	5.8069
YGL197W	YML091C	5.8069
YGL197W	YJR134C	5.8069
YGL197W	YHR098C	5.8069
YEL015W	YJR134C	5.8069
YEL015W	YHR098C	5.8069
YEL015W	YGL197W	5.8069
YHR196W	YLR129W	5.806
YJR121W	YML085C	5.8058
YJR113C	YOR243C	5.805
YAL002W	YDL077C	5.8049
YML109W	YMR155W	5.804
YJL173C	YJR141W	5.804
YGR296W	YMR155W	5.804
YGR296W	YML109W	5.804
YER029C	YGL117W	5.804
YEL064C	YMR155W	5.804
YEL064C	YML109W	5.804
YEL064C	YGR296W	5.804
YDR398W	YLR336C	5.804
YDR243C	YDR260C	5.804
YDR106W	YPL265W	5.804
YDL111C	YGL117W	5.804
YDL111C	YER029C	5.804
YCL050C	YGL117W	5.804
YCL050C	YER029C	5.804
YCL050C	YDL111C	5.804
YBR057C	YPL265W	5.804
YBR057C	YDR106W	5.804
YBL091C	YCL042W	5.804
YGL146C	YPR137W	5.8039
YBR272C	YLR421C	5.8039
YDL075W	YDR447C	5.8038
YKR063C	YLR403W	5.8035
YCL009C	YJL008C	5.8027
YMR282C	YOL123W	5.802
YMR083W	YOL031C	5.802
YIR025W	YOR249C	5.802
YIR025W	YLR102C	5.802
YGL240W	YOR249C	5.802
YGL240W	YLR102C	5.802
YDR350C	YOL123W	5.802
YDR260C	YOR249C	5.802
YDR260C	YLR102C	5.802
YDR118W	YDR260C	5.802
YDR023W	YOL123W	5.802
YGR063C	YOR210W	5.8016
YDR452W	YGL120C	5.8013
YGR278W	YLR147C	5.8009
YJR007W	YOR101W	5.8003
YGL011C	YPR108W	5.7994
YDL047W	YIL153W	5.7989
YCR063W	YJL203W	5.7982
YOL016C	YOR326W	5.7965
YJL026W	YML055W	5.7961
YJL026W	YJR140C	5.7961
YJL026W	YJL115W	5.7961
YAL016W	YMR028W	5.796
YAL032C	YER029C	5.7942
YFR008W	YOR116C	5.7938
YCR071C	YNL177C	5.7934
YBR142W	YGL242C	5.7933
YBR218C	YHR112C	5.7931
YAL053W	YOR153W	5.7931
YGL043W	YPR187W	5.7928
YLR186W	YOR310C	5.7914
YER021W	YMR259C	5.7911
YDL002C	YNL325C	5.7908
YDL002C	YLR386W	5.7908
YGR254W	YKL152C	5.7904
YML103C	YPL206C	5.7889
YML103C	YOR284W	5.7889
YJL066C	YMR241W	5.7888
YDR517W	YNL193W	5.7888
YGL128C	YHR156C	5.7882
YDR322W	YLR189C	5.7882
YNL311C	YOR027W	5.7879
YER172C	YML046W	5.7878
YAL035W	YHR216W	5.7866
YER133W	YER177W	5.7853
YGL157W	YMR109W	5.7845
YAL029C	YPR188C	5.7844
YLR345W	YML098W	5.7843
YGL123W	YLR441C	5.7841
YCR084C	YOR367W	5.7841
YCR084C	YHR016C	5.7841
YBR249C	YPL150W	5.7841
YML025C	YPL183W-A	5.7831
YBR093C	YFR055W	5.7829
YAR071W	YFR055W	5.7829
YAR071W	YBR093C	5.7829
YAL061W	YMR308C	5.7811
YKL155C	YOR243C	5.781
YGR084C	YOR243C	5.781
YGL129C	YOR243C	5.781
YDR312W	YHR066W	5.781
YMR308C	YOL115W	5.7799
YER125W	YMR205C	5.7798
YBR181C	YGL103W	5.7792
YMR246W	YOR051C	5.7789
YER089C	YPL061W	5.7789
YDL019C	YGR086C	5.7789
YFL039C	YOR181W	5.7785
YDL014W	YOL010W	5.7782
YCR012W	YDR216W	5.7782
YGR204W	YMR205C	5.7773
YCR093W	YGR184C	5.7773
YLR362W	YOR089C	5.7751
YER018C	YFR031C	5.7747
YBR151W	YML028W	5.7744
YCL059C	YDL148C	5.7741
YJL081C	YNL215W	5.7736
YER122C	YLR051C	5.7733
YDR026C	YJL148W	5.7731
YFR013W	YFR037C	5.7727
YDL031W	YDR312W	5.7717
YBR034C	YLR315W	5.7706
YMR229C	YOL077C	5.7702
YGR145W	YLR409C	5.77
YDR427W	YGL004C	5.77
YDR392W	YPL011C	5.769
YGL049C	YHR216W	5.7686
YEL023C	YGL115W	5.768
YLR455W	YOR244W	5.7671
YNL107W	YNL330C	5.7668
YGR075C	YKL188C	5.765
YMR235C	YPL111W	5.7648
YKL139W	YNL004W	5.7648
YDR168W	YNL004W	5.7648
YOR133W	YPL240C	5.7647
YBL026W	YBR152W	5.7644
YHR052W	YMR229C	5.7633
YNL071W	YPR006C	5.7632
YNL071W	YPL183C	5.7632
YGR285C	YPL048W	5.7627
YGR006W	YPR046W	5.7626
YBR103W	YOL068C	5.7626
YDL014W	YNL009W	5.7617
YDR005C	YPR110C	5.7612
YLR409C	YMR128W	5.7604
YFR001W	YHR088W	5.7577
YIL115C	YOR046C	5.7573
YNL209W	YPL101W	5.7556
YJL047C	YLL034C	5.7552
YGL105W	YLR180W	5.7546
YGR086C	YNL106C	5.7541
YER155C	YKL122C	5.7539
YFR001W	YKR081C	5.7525
YCL059C	YOR310C	5.7521
YBR229C	YIL076W	5.7507
YGR054W	YPL081W	5.7494
YAR042W	YLR183C	5.748
YAR042W	YHL020C	5.748
YAR042W	YER120W	5.748
YBR142W	YOL041C	5.747
YDR328C	YOL063C	5.7466
YDL056W	YER133W	5.7463
YBR122C	YLR312W-A	5.7462
YBR079C	YMR125W	5.7453
YIL101C	YPL139C	5.7449
YBR083W	YPL049C	5.7448
YBR119W	YPR057W	5.744
YML016C	YPR184W	5.7438
YGR159C	YKR048C	5.7433
YOL021C	YPR189W	5.743
YMR024W	YPR100W	5.743
YMR078C	YOR144C	5.7425
YLR347C	YOR304W	5.7424
YDL098C	YDL160C	5.7424
YHR137W	YKL210W	5.7416
YER091C	YML069W	5.7409
YBR253W	YML007W	5.7409
YDR064W	YPL266W	5.7408
YMR193W	YNL177C	5.7406
YBR055C	YJR084W	5.7402
YKL067W	YPL218W	5.74
YBR025C	YPR108W	5.7389
YBR025C	YFR009W	5.7386
YAL029C	YGR003W	5.7386
YJL012C	YLR087C	5.7383
YFR052W	YMR191W	5.7378
YDR033W	YGR161C	5.7373
YIL068C	YML097C	5.7369
YAL005C	YKL215C	5.7364
YDR502C	YLR153C	5.736
YBR205W	YOR176W	5.7359
YBR205W	YNL035C	5.7359
YBL003C	YDR071C	5.7359
YFR019W	YKL003C	5.7348
YFR019W	YIR029W	5.7348
YOR151C	YPR010C	5.7345
YER148W	YHR023W	5.7345
YBR087W	YHR191C	5.7342
YDL105W	YDL115C	5.7322
YDL098C	YGL128C	5.7313
YMR188C	YNR036C	5.7309
YBR107C	YGR006W	5.7309
YLR439W	YMR225C	5.729
YLR439W	YML009C	5.729
YDR091C	YDR429C	5.7287
YIL152W	YJR006W	5.7283
YER071C	YIL152W	5.7283
YER030W	YKL193C	5.7283
YCR066W	YOL016C	5.7283
YCR077C	YDL065C	5.7281
YBL058W	YCL028W	5.7278
YKL101W	YMR187C	5.7274
YOL005C	YPR093C	5.7271
YMR199W	YPR120C	5.7271
YLR226W	YMR199W	5.7271
YJR140C	YNL312W	5.7271
YJR042W	YMR209C	5.7271
YIL066C	YNL134C	5.7271
YIL066C	YKL011C	5.7271
YDR247W	YDR416W	5.7271
YDR150W	YDR247W	5.7271
YDR131C	YPR093C	5.7271
YDR131C	YGL070C	5.7271
YBL066C	YDR247W	5.7271
YBL061C	YDR247W	5.7271
YER016W	YNR047W	5.7231
YDR299W	YKR060W	5.7197
YGR192C	YIL153W	5.7192
YDL175C	YMR308C	5.7156
YER155C	YGR270W	5.7154
YEL015W	YLR438C-A	5.7154
YBL046W	YKR059W	5.7149
YER110C	YML085C	5.7147
YBR122C	YNL177C	5.7147
YGL031C	YNL301C	5.7142
YDR388W	YNR065C	5.7138
YDR388W	YLR081W	5.7138
YDR388W	YIR012W	5.7138
YFR052W	YIL107C	5.7133
YBR115C	YDR395W	5.7127
YGR167W	YNL243W	5.7126
YIL035C	YKL108W	5.7113
YGL245W	YLR044C	5.711
YCR082W	YGR113W	5.7103
YCR043C	YEL036C	5.7103
YJL039C	YML016C	5.71
YAL032C	YGL128C	5.7093
YLR113W	YOR018W	5.7088
YIL112W	YMR155W	5.7088
YIL112W	YML109W	5.7088
YGR296W	YIL112W	5.7088
YEL064C	YIL112W	5.7088
YFL026W	YMR089C	5.7073
YGR056W	YPL082C	5.7072
YDL115C	YEL019C	5.7069
YBR109C	YGR086C	5.706
YGR162W	YNL189W	5.7058
YMR143W	YMR194W	5.7056
YDL031W	YHR066W	5.705
YDR060W	YLR276C	5.7034
YDR418W	YGR085C	5.7028
YHR042W	YIL053W	5.7026
YBR205W	YHR089C	5.7023
YNL290W	YPR006C	5.702
YNL290W	YPL183C	5.702
YLR450W	YML075C	5.702
YGR270W	YMR172W	5.702
YDL132W	YLR289W	5.702
YBR185C	YNL262W	5.701
YGL121C	YIR012W	5.7002
YKR029C	YOL068C	5.7001
YAL038W	YFR009W	5.7
YCR063W	YGL120C	5.6999
YDR087C	YPR016C	5.6995
YGL206C	YML028W	5.6985
YMR145C	YOR259C	5.698
YDR416W	YHR156C	5.6971
YDL148C	YER082C	5.697
YDR075W	YLL024C	5.6969
YDR432W	YPL055C	5.6967
YDR432W	YLR356W	5.6967
YDL074C	YDR432W	5.6967
YLL008W	YNL189W	5.6955
YJR105W	YMR205C	5.6953
YEL036C	YLR342W	5.6944
YLR432W	YPR175W	5.694
YEL022W	YOR160W	5.6939
YIL079C	YOR204W	5.6938
YDL171C	YLR257W	5.6927
YAR019C	YER070W	5.6927
YKL145W	YMR259C	5.6926
YCR033W	YOR028C	5.6926
YBL058W	YLR377C	5.6925
YBL058W	YBR273C	5.6925
YER172C	YJR050W	5.6923
YER146W	YJR022W	5.6918
YGL122C	YKL186C	5.6914
YMR120C	YOR212W	5.6908
YFL030W	YIL050W	5.6908
YDR449C	YGR210C	5.6906
YHR200W	YJL008C	5.6905
YGL157W	YPL106C	5.6902
YKR022C	YPL160W	5.6901
YDR147W	YHR087W	5.6901
YBR060C	YDR171W	5.6897
YOL006C	YOL145C	5.6896
YER172C	YKR022C	5.6884
YIL107C	YPL096W	5.687
YER131W	YKR027W	5.6863
YER131W	YJL099W	5.6863
YBR205W	YDR346C	5.6863
YJL098W	YPR040W	5.6857
YIL091C	YNL289W	5.685
YDL029W	YJR064W	5.6844
YDR074W	YGR129W	5.6839
YDL190C	YGR048W	5.6839
YLR197W	YNL075W	5.6834
YGR103W	YPL043W	5.6828
YJL081C	YNL136W	5.6813
YDL060W	YPL081W	5.6797
YGR234W	YJL008C	5.6792
YIL033C	YNL093W	5.6791
YBL084C	YDR260C	5.6785
YLR275W	YOR093C	5.6784
YCR031C	YGL008C	5.6784
YCL054W	YDL148C	5.678
YNL005C	YOR249C	5.6777
YGL113W	YOR038C	5.6777
YLR099C	YLR287C	5.6773
YDR499W	YMR190C	5.6773
YDR499W	YLR234W	5.6773
YDR499W	YJR144W	5.6773
YBR103W	YNL189W	5.6756
YAR014C	YGR156W	5.6755
YML029W	YMR173W	5.6753
YJR112W	YMR117C	5.6753
YBR160W	YOL001W	5.6753
YBR061C	YMR259C	5.6753
Q0140	YPL013C	5.6753
YER012W	YFR052W	5.6751
YDR457W	YOL025W	5.6751
YML046W	YPR101W	5.6747
YDR032C	YPL218W	5.674
YCR030C	YGL207W	5.674
YHR027C	YKL184W	5.6734
YBL049W	YBR105C	5.6731
YEL003W	YIL115C	5.6728
YDR490C	YOR019W	5.6728
YBR109C	YKL119C	5.6728
YNL178W	YPL090C	5.6727
YMR261C	YNL076W	5.6708
YLR452C	YPL243W	5.6708
YER008C	YML097C	5.6708
YDR166C	YML097C	5.6708
YDR074W	YMR251W	5.6708
YDR074W	YLR411W	5.6708
YDR074W	YIR032C	5.6708
YDR074W	YIL177C	5.6708
YMR033W	YMR125W	5.6705
YER112W	YNR011C	5.6704
YDL150W	YNL232W	5.6702
YBR282W	YJL063C	5.6692
YGR167W	YOR181W	5.6683
YDL073W	YGR078C	5.6666
YNL048W	YOR239W	5.6657
YGL245W	YLR249W	5.6656
YDR462W	YMR225C	5.6648
YDR462W	YML009C	5.6648
YGL049C	YPL012W	5.6646
YER090W	YKL204W	5.6643
YKL191W	YOL109W	5.6641
YIL101C	YPL216W	5.6605
YCR031C	YNL301C	5.6602
YDR432W	YGR054W	5.6601
YDR101C	YNL002C	5.6599
YBR080C	YDL147W	5.6595
YDR075W	YGR063C	5.6583
YBL071W-A	YLR043C	5.6583
YER136W	YLR362W	5.6579
YEL060C	YNL106C	5.6575
YDR022C	YOR361C	5.6572
YER082C	YKR060W	5.6562
YLR304C	YNR046W	5.6558
YCR047C	YLR304C	5.6558
YGR185C	YLL029W	5.6557
YDR162C	YNR058W	5.6556
YDL188C	YMR028W	5.6556
YBR236C	YML010W	5.6555
YDR310C	YMR308C	5.6552
YDL188C	YPL003W	5.6552
YDR235W	YDR416W	5.6549
YFL045C	YNR016C	5.6532
YLR051C	YNL287W	5.6509
YDR226W	YGR234W	5.6508
YIL069C	YJR066W	5.6498
YFR031C	YOL069W	5.6498
YLR016C	YMR240C	5.6497
YMR146C	YNL096C	5.6487
YDR469W	YER056C-A	5.6485
YDR378C	YGR075C	5.6485
YLR028C	YPL137C	5.6481
YDR507C	YML095C	5.6481
YDR150W	YHL020C	5.6481
YDR150W	YER120W	5.6481
YCL024W	YPL022W	5.6481
YCL024W	YML095C	5.6481
YLR074C	YPR016C	5.6479
YGL091C	YIL129C	5.6479
YCR088W	YJR065C	5.6476
YGL111W	YPR016C	5.6474
YGR175C	YNL311C	5.6472
YGL106W	YKL129C	5.6472
YDR416W	YNL210W	5.6472
YDR356W	YGL106W	5.6472
YDR150W	YNL210W	5.6472
YDR142C	YPL226W	5.6472
YBR179C	YDR142C	5.6472
YBR135W	YPR120C	5.6472
YBR135W	YLR226W	5.6472
YBL066C	YNL210W	5.6472
YBL061C	YNL210W	5.6472
YLL008W	YMR229C	5.6469
YDR448W	YML015C	5.6456
YDL156W	YMR190C	5.6456
YDL156W	YLR234W	5.6456
YDL156W	YJR144W	5.6456
YER070W	YJL066C	5.645
YDL030W	YLR323C	5.6449
YDL156W	YGL241W	5.6448
YBL045C	YDR432W	5.6438
YGR027C	YOL040C	5.6431
YDR427W	YOL145C	5.6423
YBL003C	YOR116C	5.6421
YIL152W	YIR003W	5.6414
YBR159W	YOR233W	5.6403
YAL026C	YOR153W	5.6392
YDR074W	YPL106C	5.6379
YGL133W	YMR033W	5.6368
YLR196W	YPR145W	5.6359
YGR124W	YLR196W	5.6359
YIL128W	YOR136W	5.6353
YEL030W	YOL109W	5.6348
YGR185C	YNL306W	5.6342
YGR128C	YOL086C	5.6341
YDR322W	YMR225C	5.6335
YDR322W	YML009C	5.6335
YBR142W	YDR060W	5.6332
YDL066W	YNL250W	5.6331
YLR002C	YLR196W	5.6321
YLR051C	YPL010W	5.6319
YBR146W	YDR124W	5.6319
YMR125W	YMR288W	5.6318
YBR055C	YPR057W	5.6316
YIL046W	YPL038W	5.6302
YFR001W	YLL008W	5.6302
YER179W	YFL016C	5.6302
YDR139C	YIL046W	5.6302
YDL175C	YGR165W	5.6302
YDR137W	YNL307C	5.6296
YDL098C	YJL035C	5.6296
YCL054W	YHR052W	5.6273
YDR328C	YLL039C	5.6265
YHR118C	YLL022C	5.6264
YDR299W	YMR128W	5.6264
YLR344W	YOR069W	5.6258
YHR015W	YOR069W	5.6258
YER087W	YJL050W	5.6258
YNL071W	YPR140W	5.6249
YAL005C	YGL119W	5.6239
YJL076W	YOR340C	5.6238
YFR019W	YOR195W	5.6238
YFR019W	YIL159W	5.6238
YAL005C	YGR123C	5.6238
YBR125C	YMR140W	5.6237
YBR034C	YBR211C	5.6237
YBR265W	YDR074W	5.6221
YBL023C	YDR074W	5.6221
YGR192C	YNL258C	5.622
YGR192C	YLR440C	5.622
YJR052W	YOL012C	5.6219
YIL149C	YKL216W	5.6219
YDR477W	YMR086W	5.6217
YLR429W	YNL138W	5.6216
YEL024W	YLR109W	5.6208
YDL200C	YLR109W	5.6208
YBR084W	YOL002C	5.6202
YBR084W	YDR372C	5.6202
YDR314C	YHR027C	5.6192
YGL111W	YKL009W	5.6189
YFL039C	YGL026C	5.6179
YJR053W	YMR246W	5.6178
YMR297W	YPL154C	5.6177
YPL082C	YPR110C	5.6175
YJR084W	YML049C	5.6168
YER146W	YKL173W	5.6165
YDR462W	YNL284C	5.616
YDL229W	YPR131C	5.6158
YDR099W	YLR044C	5.6152
YLR389C	YOL063C	5.6148
YLR389C	YNR034W	5.6148
YJL047C	YLR389C	5.6148
YER165W	YNL251C	5.6147
YHR060W	YNL073W	5.6136
YHR043C	YNL073W	5.6136
YHR043C	YHR060W	5.6136
YER182W	YGL205W	5.6136
YEL062W	YGL205W	5.6136
YEL062W	YER182W	5.6136
YGR085C	YMR194W	5.6135
YFL023W	YPR110C	5.6133
YEL012W	YLR410W	5.6133
YNL112W	YPL037C	5.6132
YBR194W	YLL036C	5.6132
YDR343C	YML123C	5.6116
YIL009C-A	YML092C	5.6111
YDR448W	YOR174W	5.6111
YDR225W	YKR008W	5.609
YMR091C	YMR125W	5.6088
YIR007W	YNL172W	5.6087
YGR097W	YOR220W	5.6087
YGR097W	YLR256W	5.6087
YGL181W	YGR097W	5.6087
YBR059C	YGR097W	5.6087
YGR090W	YOR145C	5.6081
YNL076W	YPR035W	5.6064
YLR002C	YPR131C	5.6057
YBR247C	YPL126W	5.6054
YDR028C	YER129W	5.6052
YDL007W	YGL004C	5.605
YKL014C	YOR206W	5.6041
YDL083C	YIL133C	5.6041
YBR245C	YNL059C	5.6039
YDR257C	YLR347C	5.6032
YBL006C	YMR033W	5.6023
YGR020C	YHR060W	5.6016
YDR507C	YNL078W	5.6016
YCL024W	YNL078W	5.6016
YDR515W	YOR359W	5.6007
YFL007W	YIL009C-A	5.6003
YDR225W	YOR056C	5.5999
YDR347W	YOR243C	5.5994
YAL012W	YPL149W	5.5969
YHR084W	YJL098W	5.5968
YGL212W	YJL098W	5.5968
YER162C	YPR032W	5.5968
YEL037C	YPR032W	5.5968
YDR480W	YJL098W	5.5968
YNL081C	YPR166C	5.5957
YER050C	YPR166C	5.5957
YER050C	YNL081C	5.5957
YIL018W	YKL075C	5.595
YEL055C	YPL012W	5.5948
YCL011C	YLR175W	5.5946
YDR224C	YPL128C	5.5941
YDR496C	YKR024C	5.594
YJR002W	YOR145C	5.5936
YKL206C	YML119W	5.5927
YNR054C	YOR310C	5.5908
YCR032W	YIL066C	5.5905
YBR154C	YFL023W	5.5901
YHR163W	YPR162C	5.5897
YDR037W	YPR056W	5.5897
YBR169C	YOR164C	5.5897
YHR030C	YPR054W	5.589
YFR053C	YLR044C	5.5884
YDR190C	YPL049C	5.5879
YBL099W	YBR039W	5.5874
YDR364C	YGL120C	5.5872
YDR091C	YOR096W	5.5869
YBR282W	YML025C	5.5863
YLR045C	YML124C	5.586
YLR439W	YPL183W-A	5.5859
YGL009C	YMR205C	5.5859
YDR310C	YOL068C	5.5859
YMR172W	YMR309C	5.5856
YDL140C	YER139C	5.5854
YIR002C	YMR190C	5.585
YIR002C	YLR234W	5.585
YDR097C	YMR190C	5.585
YDR097C	YLR234W	5.585
YDR097C	YJR144W	5.585
YKR026C	YOR101W	5.5848
YDR322W	YNL284C	5.5847
YDR033W	YDR293C	5.5847
YFL045C	YFR009W	5.5846
YHR216W	YPR067W	5.5825
YGR062C	YHR216W	5.5825
YAR075W	YHR216W	5.5825
YDR099W	YJL052W	5.5819
YGL227W	YPL018W	5.581
YDR196C	YJL194W	5.5803
YDL100C	YJL194W	5.5803
YBL047C	YDR348C	5.5792
YHR143W-A	YOR340C	5.5773
YFR037C	YPL235W	5.5771
YKR064W	YLR127C	5.577
YHR012W	YPL070W	5.577
YER172C	YPR057W	5.5764
YER148W	YML015C	5.5757
YDR141C	YNL271C	5.575
YGR204W	YLL018C	5.5749
YBR160W	YGR233C	5.5737
YGR237C	YKR092C	5.5736
YGL123W	YPR036W	5.5734
YGL141W	YOR035C	5.5732
YGL141W	YKL129C	5.5732
YDR356W	YGL141W	5.5732
YDR131C	YOR057W	5.5732
YDR131C	YLR267W	5.5732
YDL116W	YMR209C	5.5732
YDL003W	YDR229W	5.5732
YAL047C	YDL003W	5.5732
YLR097C	YLR289W	5.5731
YIL068C	YLR105C	5.5731
YOR375C	YPL160W	5.5726
YGR072W	YLR203C	5.5726
YIR010W	YLR381W	5.5722
YFR040W	YNL187W	5.5722
YFR040W	YNL101W	5.5722
YDR318W	YIR010W	5.5722
YDR254W	YIR010W	5.5722
YBR247C	YCL059C	5.5717
YJL011C	YNL248C	5.5699
YGR091W	YLL036C	5.5696
YBR198C	YPL082C	5.5695
YBL017C	YBR085W	5.5695
YDR292C	YNL102W	5.5687
YDR292C	YKL045W	5.5687
YDR292C	YIR008C	5.5687
YDR260C	YNL172W	5.5687
YBL035C	YDR292C	5.5687
YLR264W	YNL207W	5.5682
YDL014W	YDR324C	5.5674
YDR168W	YPL240C	5.5668
YDL097C	YLR278C	5.5661
YDR127W	YGL245W	5.5655
YER148W	YPL011C	5.5649
YLR432W	YMR290C	5.5642
YDR087C	YHR088W	5.5642
YJL016W	YPL083C	5.5641
YJL016W	YLR386W	5.5641
YGR063C	YPR180W	5.5641
YDR293C	YGR063C	5.5639
YCL010C	YGL151W	5.5631
YHR012W	YNL071W	5.5629
YGL127C	YGR104C	5.5629
YJL039C	YPR184W	5.5618
YBL026W	YGL158W	5.5615
YBL008W	YJR140C	5.5615
YGL112C	YGL151W	5.5611
YIL070C	YJR140C	5.5594
YGR128C	YPR137W	5.5594
YPL225W	YPL226W	5.5593
YFL017W-A	YNR011C	5.5593
YBL006C	YMR091C	5.5593
YGL128C	YPR178W	5.5575
YDR162C	YER178W	5.5573
YDR135C	YDR171W	5.5564
YML010W	YOR133W	5.5561
YJL101C	YPL061W	5.5561
YGR020C	YPL201C	5.556
YFL008W	YLL023C	5.556
YLR406C	YNL182C	5.5559
YCL028W	YEL021W	5.5559
YDL063C	YLR293C	5.5557
YDL002C	YDL029W	5.5541
YDR127W	YDR128W	5.5533
YDR054C	YOL133W	5.5527
YDR054C	YOL063C	5.5527
YHR197W	YLL034C	5.5523
YNL132W	YPR137W	5.5522
YBL091C	YDR420W	5.552
YDR453C	YLL029W	5.5506
YDR516C	YGR020C	5.5504
YGL049C	YLR432W	5.5483
YKL196C	YOL018C	5.548
YGL095C	YKL196C	5.548
YDR468C	YKL196C	5.548
YOR117W	YPL002C	5.5471
YGL048C	YPL002C	5.5471
YKR001C	YPL235W	5.5467
YNL254C	YNL284C	5.5463
YMR117C	YPL233W	5.5463
YAL036C	YJL138C	5.5463
YDL105W	YLR383W	5.5462
YJR092W	YKL085W	5.5459
YML124C	YOR005C	5.5452
YEL003W	YJL041W	5.5452
YIR031C	YPL256C	5.5447
YER146W	YLR438C-A	5.5447
YDL155W	YPL256C	5.5447
YDR224C	YLR033W	5.5437
YDR141C	YKL218C	5.542
YDR017C	YMR196W	5.542
YDL112W	YMR155W	5.542
YDL112W	YML109W	5.542
YDL112W	YGR296W	5.542
YDL112W	YEL064C	5.542
YER102W	YOR293W	5.5419
YJL117W	YKL057C	5.5413
YJR063W	YPL255W	5.5398
YDL030W	YNL021W	5.5398
YOR207C	YOR304W	5.5386
YBR143C	YEL052W	5.538
YDL156W	YIL035C	5.5373
YGR128C	YKR060W	5.537
YDL185W	YMR318C	5.536
YBL027W	YFR031C-A	5.5353
YML010W	YPL129W	5.5351
YBR118W	YNL007C	5.5348
YDR342C	YLR447C	5.534
YHR034C	YMR309C	5.5335
YDR394W	YJR068W	5.5335
YNL287W	YPR117W	5.5333
YNL272C	YNL287W	5.5333
YDR124W	YDR347W	5.5333
YBR034C	YGR179C	5.5333
YGR205W	YJL034W	5.5328
YBR139W	YJL034W	5.5328
YHR052W	YNL182C	5.5317
YNL032W	YNL249C	5.5313
YDR028C	YOR220W	5.5313
YDR028C	YLR256W	5.5313
YDR028C	YGL181W	5.5313
YBR059C	YDR028C	5.5313
YDR386W	YPL129W	5.5305
YDL208W	YPL129W	5.5305
YBR098W	YPL129W	5.5305
YBL023C	YER062C	5.5297
YGL106W	YNL106C	5.5294
YDR453C	YNL306W	5.5291
YDR314C	YPL048W	5.5291
YLR312W-A	YOR150W	5.5281
YKR096W	YML091C	5.528
YIL151C	YML091C	5.528
YIL016W	YLR173W	5.528
YLR291C	YOR101W	5.5277
YEL030W	YEL060C	5.5274
YDR416W	YMR240C	5.5273
YCR031C	YFR031C-A	5.5273
YDL148C	YHR148W	5.527
YGR261C	YMR304W	5.5269
YGR261C	YLR436C	5.5269
YFR024C-A	YGR167W	5.5269
YOL045W	YPL029W	5.5268
YDR523C	YOR367W	5.5267
YDR523C	YHR016C	5.5267
YDL148C	YPR183W	5.5267
YGR280C	YPL110C	5.5261
YDR237W	YPR100W	5.5261
YDL148C	YGR145W	5.5258
YKL172W	YMR290C	5.5245
YDR513W	YHR164C	5.5239
YHR027C	YMR259C	5.5232
YCL014W	YDL105W	5.5232
YDR388W	YNL208W	5.5229
YDR462W	YPL183W-A	5.5218
YBL074C	YML046W	5.5216
YAL032C	YDL030W	5.5213
YBL003C	YGL133W	5.521
YGL048C	YLR278C	5.5209
YDR496C	YFL002C	5.5208
YDR496C	YNL112W	5.5206
YBR143C	YGR090W	5.5205
YHL001W	YHL033C	5.5194
YIL106W	YPR122W	5.5192
YIL106W	YOR210W	5.5192
YBL003C	YIL126W	5.5187
YGR175C	YHR020W	5.5176
YER178W	YOR362C	5.5174
YCL010C	YHR041C	5.5174
YBR120C	YKR046C	5.5171
YOR206W	YPL131W	5.517
YKL211C	YKL218C	5.517
YER090W	YKL218C	5.517
YOL006C	YPL082C	5.5169
YBR055C	YBR119W	5.5168
YLR345W	YMR310C	5.5165
YER178W	YPR140W	5.5165
YDL051W	YOR078W	5.5165
YDR124W	YOR158W	5.5164
YLR435W	YOR369C	5.516
YDL160C	YER172C	5.5157
YDL030W	YER112W	5.5149
YAL003W	YLL026W	5.5139
YHR148W	YKR060W	5.5137
YDR224C	YHR056C	5.5137
YGL201C	YLR274W	5.5136
YLR418C	YML063W	5.5134
YJL137C	YMR033W	5.5131
YFR053C	YKL081W	5.5114
YDR228C	YHR216W	5.5111
YKR001C	YMR072W	5.5106
YER125W	YML123C	5.5106
YGR145W	YJL109C	5.5102
YFR009W	YKL029C	5.51
YLL008W	YLL045C	5.5095
YPL012W	YPL217C	5.5092
YGR135W	YML130C	5.5082
YEL013W	YLR087C	5.5082
YEL013W	YJL012C	5.5082
YDR538W	YMR304W	5.5082
YDR260C	YLR127C	5.5082
YDL066W	YMR304W	5.5082
Q0080	YLR347C	5.5072
YBL026W	YHR156C	5.5069
YIL075C	YOL018C	5.506
YKR029C	YNL189W	5.5051
YKL193C	YLR449W	5.5047
YDR229W	YIL026C	5.5043
YCR066W	YDR092W	5.5043
YAL047C	YIL026C	5.5043
YBL046W	YOR133W	5.5032
YDR035W	YGR180C	5.5028
YNL139C	YPL179W	5.5019
YDR285W	YNR024W	5.5019
YLR028C	YLR258W	5.5017
YGL156W	YPR032W	5.5017
YGL156W	YJR063W	5.5017
YDL014W	YLR221C	5.5009
YDR477W	YOR136W	5.5
YGL121C	YIL142W	5.4999
YDR188W	YGL121C	5.4999
YDL143W	YGL121C	5.4999
YDR517W	YGR155W	5.499
YFL017W-A	YJL035C	5.4988
YJL183W	YPR006C	5.4984
YJL183W	YPL183C	5.4984
YCL014W	YEL019C	5.4981
YER164W	YKL088W	5.4974
YDR381W	YOR211C	5.4965
YMR044W	YMR072W	5.4962
YHR196W	YPL217C	5.4959
YER127W	YOR063W	5.4957
YLL036C	YLR323C	5.4956
YDR392W	YMR005W	5.4956
YGR095C	YPL117C	5.4954
YBL076C	YLR355C	5.4953
YDR129C	YDR353W	5.4942
YBL026W	YDR363W	5.4926
YER148W	YPL082C	5.4923
YGL112C	YHR041C	5.492
YBR143C	YGR180C	5.4915
YGL174W	YLR147C	5.4913
YDR322W	YPL183W-A	5.4906
YKR017C	YOR299W	5.4899
YJL207C	YOR299W	5.4899
YGR282C	YJL076W	5.4899
YDR495C	YOR299W	5.4899
YGL043W	YOR210W	5.4888
YBL074C	YGR013W	5.4882
YJL061W	YLR276C	5.4876
YDR478W	YML028W	5.4872
YDL126C	YHR020W	5.4868
YJR145C	YOR293W	5.4864
YFL039C	YLR454W	5.4858
YNR054C	YPL198W	5.484
YDR012W	YOL142W	5.4835
YOR229W	YOR230W	5.4833
YER172C	YHR086W	5.4828
YPL113C	YPR019W	5.4823
YDR143C	YPL113C	5.4823
YDL117W	YIL021W	5.4822
YFR040W	YNL242W	5.482
YLR057W	YPR184W	5.4815
YDL188C	YHR132W-A	5.4815
YAL034C	YLR057W	5.4815
YLL034C	YOR272W	5.4812
YJL138C	YMR186W	5.4811
YGL112C	YOR174W	5.4807
YER091C	YPR074C	5.4803
YCR035C	YKR025W	5.4796
YDR171W	YLL004W	5.4793
YIL115C	YNL153C	5.4774
YFR019W	YOR019W	5.4774
YIL035C	YNL206C	5.4761
YOR326W	YPR188C	5.4759
YDL082W	YFR031C-A	5.4756
YJR063W	YLR143W	5.4754
YDR359C	YML007W	5.4754
YBR107C	YPR046W	5.4754
YMR213W	YMR240C	5.4748
YDR418W	YDR465C	5.4745
YDR224C	YML127W	5.4744
YER006W	YLR276C	5.473
YNL151C	YNL232W	5.4718
YDR201W	YKL138C-A	5.4714
YDR127W	YPR010C	5.4705
YJL137C	YMR091C	5.4701
YKR081C	YLR196W	5.4689
YJL066C	YML124C	5.4684
YGR116W	YLR199C	5.4684
YCL054W	YDR087C	5.468
YDR301W	YGR187C	5.4679
YHR011W	YIL063C	5.4678
YDR353W	YNL180C	5.4673
YDR229W	YGL212W	5.4673
YHR200W	YMR191W	5.4672
YLL005C	YML057W	5.4664
YEL055C	YLR243W	5.4663
YDL055C	YEL055C	5.4663
YER148W	YGL112C	5.466
YBR198C	YDR224C	5.4658
YJL081C	YPL139C	5.4654
YHR064C	YOR335C	5.4652
YDL043C	YKL012W	5.4647
YHR147C	YKR006C	5.4643
YHR147C	YKL167C	5.4643
YDR496C	YPL220W	5.4638
YMR261C	YPR035W	5.4636
YDL160C	YNL248C	5.4636
YDR471W	YGR034W	5.4635
YDL066W	YER151C	5.4635
YBL038W	YNL177C	5.4627
YLR386W	YPL083C	5.4625
YDR028C	YDR475C	5.4624
YER063W	YMR308C	5.4621
YBR017C	YLR259C	5.4612
YBL036C	YMR158W	5.4604
YDR155C	YLR249W	5.4602
YBL092W	YGL103W	5.4601
YGR063C	YNL201C	5.4595
YOR287C	YPR137W	5.4594
YKL114C	YPL127C	5.4593
YLR043C	YOL109W	5.4591
YIL142W	YJL008C	5.4582
YFL045C	YGL137W	5.458
YGR161C	YOR157C	5.4578
YBR055C	YDR235W	5.4573
YGL206C	YIR006C	5.4571
YDR418W	YGL123W	5.457
YBR247C	YER082C	5.4568
YGR123C	YLR069C	5.4555
YDR439W	YLL029W	5.4554
YCR086W	YLL029W	5.4554
YGL156W	YNL248C	5.4553
YDR028C	YGL208W	5.4552
YBR121C	YMR205C	5.4551
YLR276C	YMR049C	5.4544
YAL019W	YJR119C	5.4535
YCR057C	YOR310C	5.4532
YDR394W	YJR109C	5.4531
YBR080C	YDL097C	5.4531
YJL034W	YPR181C	5.4529
YCR052W	YPL082C	5.4527
YBR189W	YJR123W	5.4526
YHR027C	YLR180W	5.4524
YER088C	YNL246W	5.4524
YDR432W	YHR086W	5.4521
YDL043C	YGR075C	5.4521
YDR293C	YLR433C	5.4514
YDL148C	YPL266W	5.4514
YBL056W	YNL250W	5.4512
YBR143C	YIL074C	5.4511
YCR071C	YHR147C	5.4509
YAR002C-A	YLR342W	5.4508
YMR290C	YNL112W	5.4507
YLR223C	YOR061W	5.4506
YKL064W	YOR061W	5.4506
YKR025W	YOR340C	5.4502
YDL083C	YOR096W	5.449
YDR280W	YPR190C	5.4485
YBL033C	YLL024C	5.4482
YGR078C	YIL125W	5.4481
YDR283C	YKR096W	5.4481
YDR283C	YIL151C	5.4481
YBR069C	YNL064C	5.4481
YKL188C	YNL256W	5.4478
YJL074C	YLL023C	5.4476
YNL178W	YOR293W	5.4474
YOR069W	YOR259C	5.4473
YBL003C	YIL056W	5.4473
YJR074W	YMR235C	5.4469
YDL112W	YIL112W	5.4469
YDR240C	YHR165C	5.4464
YGR145W	YLR197W	5.446
YDR069C	YMR263W	5.4457
YEL054C	YJR145C	5.4448
YJR084W	YPR178W	5.4447
YBL037W	YDR098C	5.4447
YFR051C	YLR051C	5.4436
YDL105W	YPR169W	5.4431
YIL133C	YLR029C	5.443
YBR251W	YPR185W	5.443
YBR251W	YGL180W	5.443
YDL047W	YHR084W	5.4429
YDL047W	YGL212W	5.4429
YDL047W	YDR480W	5.4429
YKL126W	YNL104C	5.4428
YLR196W	YPL012W	5.4422
YER094C	YIL009C-A	5.4419
YLL010C	YLL024C	5.4418
YEL012W	YMR140W	5.4418
YBL058W	YNL155W	5.4417
YGL133W	YPL128C	5.4416
YGR162W	YNL016W	5.4409
YCR079W	YIL021W	5.4407
YML058W	YMR083W	5.4399
YLR006C	YLR233C	5.4399
YGR047C	YGR238C	5.4399
YDR131C	YFL009W	5.4399
YDL040C	YMR083W	5.4399
YDL040C	YML058W	5.4399
YIL108W	YLR300W	5.4398
YOR063W	YPL093W	5.4397
YOL128C	YOR185C	5.4397
YNR031C	YOR208W	5.4392
YIL133C	YNL301C	5.4388
YGL135W	YIL133C	5.438
YNL039W	YOR116C	5.4378
YNL138W	YPL031C	5.4369
YLR436C	YNR047W	5.436
YDR538W	YNR047W	5.436
YDL066W	YNR047W	5.436
YCR014C	YHR200W	5.436
YIL112W	YOL068C	5.4359
YAL003W	YJL034W	5.4357
YDR021W	YJL138C	5.4351
YBR048W	YDR365C	5.4351
YDR188W	YKL029C	5.4347
YIL133C	YKL180W	5.4344
YDR436W	YER133W	5.4342
YGL067W	YKR037C	5.434
YDR171W	YPR162C	5.4337
YDR075W	YGR192C	5.4332
YFR031C-A	YKL180W	5.433
YDR488C	YNR058W	5.4329
YBL064C	YNR058W	5.4329
YDR379W	YML010W	5.4328
YGR162W	YLR175W	5.4327
YHR023W	YMR299C	5.4326
YDR032C	YFL039C	5.4325
YEL003W	YJL061W	5.4321
YLR354C	YNL222W	5.4319
YGR043C	YNL222W	5.4319
YBR017C	YML029W	5.4318
YDL156W	YDR499W	5.4309
YHR027C	YLR278C	5.4298
YHL038C	YLR432W	5.4295
YDR044W	YOR093C	5.4282
YDL145C	YLR051C	5.4281
YNL092W	YOR159C	5.4279
YJL026W	YKL029C	5.4275
YGR159C	YML049C	5.4262
YHR066W	YKL172W	5.4256
YER165W	YJL050W	5.4256
YKL218C	YPR010C	5.4252
YNL137C	YOR243C	5.4239
YDR198C	YPL169C	5.4235
YLR449W	YMR121C	5.423
YGL120C	YLR196W	5.4229
YER089C	YIL104C	5.4228
YPL012W	YPR112C	5.4227
YGL151W	YOL148C	5.4225
YHR099W	YNL136W	5.4223
YHR039C-A	YOR270C	5.4218
YLR002C	YOR063W	5.4216
YDL060W	YOR145C	5.4214
YDR238C	YLR051C	5.4205
YGR002C	YJR082C	5.4199
YBR011C	YPL169C	5.4198
YLR276C	YOR206W	5.4196
YAL015C	YDR214W	5.4196
YLL034C	YLR289W	5.4193
YNL254C	YPL173W	5.4187
YFL023W	YPR133C	5.4187
YBR094W	YJL204C	5.4187
YEL019C	YPR169W	5.418
YBR160W	YIR031C	5.4178
YBR136W	YHR164C	5.4178
YBR135W	YIR031C	5.4178
YGR252W	YOL135C	5.4167
YJR065C	YOR141C	5.4165
YDL031W	YPL043W	5.416
YLL045C	YPL178W	5.4156
YHR191C	YNL290W	5.4148
YJR045C	YKR082W	5.4143
YBR221C	YPR152C	5.4126
YBR181C	YGR027C	5.4126
YAL036C	YLL045C	5.4115
YJL069C	YJR002W	5.4112
YBR234C	YJR064W	5.4109
YKR028W	YLR310C	5.4099
YDR054C	YIL148W	5.4095
YMR165C	YPL031C	5.4093
YHR156C	YLR424W	5.409
YDR074W	YNL076W	5.409
YDR171W	YLL022C	5.4074
YML056C	YPR067W	5.4073
YGR062C	YML056C	5.4073
YAR075W	YML056C	5.4073
YAR007C	YDR190C	5.4071
YLR196W	YMR229C	5.4065
YAL062W	YPL203W	5.4055
YDR045C	YKL179C	5.4045
YGR034W	YJL177W	5.4039
YEL026W	YKR092C	5.4033
YGR218W	YHR020W	5.4031
YHR191C	YPR175W	5.403
YGL252C	YNL267W	5.403
YBR001C	YGL252C	5.403
YDL134C	YMR167W	5.4026
YDR216W	YHR174W	5.4021
YLR196W	YNL061W	5.402
YDR301W	YOR179C	5.4017
YEL054C	YHR170W	5.4013
YDL087C	YMR288W	5.401
YER006W	YFR001W	5.4008
YBR025C	YDR427W	5.4002
YDL126C	YKL039W	5.3999
YHR204W	YNL312W	5.3996
YCL043C	YNL312W	5.3996
YAL024C	YNL312W	5.3996
YLR340W	YOL039W	5.3994
YJL136C	YLR388W	5.3992
YJL099W	YKR027W	5.3992
YOR023C	YPR119W	5.3991
YMR116C	YOR056C	5.3991
YJL187C	YPR119W	5.3991
YLR433C	YPR159W	5.399
YKL190W	YPR159W	5.399
YAL016W	YOR162C	5.3988
YDL003W	YLR347C	5.3984
YDR028C	YMR120C	5.398
YLR134W	YPL258C	5.3973
YDL101C	YDR217C	5.3973
YLR382C	YPR101W	5.3968
YBL104C	YPR101W	5.3968
YJR132W	YMR308C	5.3967
YER172C	YMR268C	5.3963
YJL154C	YNL071W	5.3962
YGR252W	YMR227C	5.3962
YIL126W	YPR106W	5.3959
YBR205W	YMR145C	5.3959
YHL001W	YJL177W	5.3958
YDL043C	YDR473C	5.3954
YBR264C	YIL075C	5.3954
YBL006C	YDR335W	5.3952
YNL301C	YOR086C	5.3944
YDR224C	YLR109W	5.3933
YLR075W	YMR290C	5.3931
YDR496C	YLR276C	5.3926
YDL014W	YOR061W	5.3925
YBR245C	YIR008C	5.3924
YJL026W	YKL191W	5.3913
YIL103W	YJL026W	5.3913
YBR109C	YNL106C	5.3913
YDR141C	YPL219W	5.3906
YFL037W	YLR401C	5.3905
YBR119W	YHR165C	5.3888
YBL004W	YOR310C	5.3886
YHL034C	YMR189W	5.3878
YBR221C	YOR157C	5.3877
YGL061C	YMR308C	5.3874
YDR471W	YOL127W	5.3861
YPL262W	YPR165W	5.3859
YJR016C	YPL262W	5.3859
YFR044C	YPL262W	5.3859
YDR002W	YPL111W	5.3859
YOR154W	YOR220W	5.3857
YDR237W	YMR098C	5.3857
YCR073C	YNL250W	5.3857
YCL014W	YDR237W	5.3857
YHR076W	YMR137C	5.3852
YBL041W	YKL145W	5.3849
YGR252W	YML007W	5.384
YBR253W	YGR252W	5.384
YJL138C	YOR323C	5.3837
YHR156C	YLL036C	5.3837
YGR054W	YOR145C	5.3834
YFR031C-A	YML073C	5.3832
YDR124W	YPL118W	5.3831
YGR209C	YJR045C	5.3828
YER146W	YNL092W	5.3828
YEL037C	YKL191W	5.3827
YHR088W	YNL110C	5.3825
YKL099C	YLR129W	5.3811
YAL035W	YER102W	5.3805
YMR064W	YMR282C	5.3799
YDR350C	YMR064W	5.3799
YDR023W	YMR064W	5.3799
YBR177C	YMR109W	5.3798
YDR012W	YPR080W	5.3797
YPL116W	YPL220W	5.3796
YGL061C	YKL138C-A	5.3795
YNL192W	YNL201C	5.3794
YMR019W	YPL254W	5.3794
YKL045W	YNL201C	5.3794
YHR082C	YNL201C	5.3794
YGR002C	YPL254W	5.3794
YER015W	YPL254W	5.3794
YDR341C	YIR005W	5.3794
YDR146C	YPL254W	5.3794
YLL029W	YNL096C	5.3784
YDL047W	YMR028W	5.3779
YFL024C	YNL330C	5.3773
YHR041C	YOL148C	5.377
YHR023W	YLR429W	5.3764
YDL014W	YKR024C	5.3747
YKL142W	YLR099C	5.3742
YDL019C	YKL057C	5.3742
YDL019C	YDR150W	5.3742
YNL187W	YOR267C	5.3741
YNL101W	YOR267C	5.3741
YLL045C	YLR029C	5.3741
YAL003W	YIR033W	5.3741
YGR180C	YLR259C	5.3739
YLL045C	YOR253W	5.3736
YCR012W	YMR173W	5.3735
YCR012W	YGR279C	5.3735
YCR012W	YEL031W	5.3735
YER179W	YPR183W	5.3729
YMR024W	YNL284C	5.3723
YDR227W	YGL121C	5.3723
YHR030C	YIL113W	5.3721
YAR019C	YNL223W	5.3721
YDR099W	YNR014W	5.3718
YJL020C	YOR124C	5.3705
YGR211W	YPL013C	5.3705
YDR383C	YER069W	5.3705
YDR499W	YIR002C	5.3704
YDR097C	YDR499W	5.3704
YNL059C	YPL053C	5.3703
YHR034C	YNL059C	5.3703
YDR334W	YNL059C	5.3703
YAL032C	YLR147C	5.3702
YDR427W	YIL066C	5.3701
YNL132W	YNL178W	5.3691
YKR062W	YOR063W	5.3691
YHR009C	YOL139C	5.369
YHR089C	YMR229C	5.3687
YGL120C	YNL132W	5.3682
YKL180W	YML073C	5.368
YLR290C	YOL135C	5.3668
YBL075C	YLR345W	5.3666
YIL097W	YPL018W	5.3663
YGL099W	YGR103W	5.3661
YFL039C	YNL208W	5.3661
YDR450W	YGR034W	5.3661
YFL010C	YGR061C	5.366
YKL184W	YOR261C	5.3655
YBL022C	YGR040W	5.3655
YDL014W	YER102W	5.3651
YNL035C	YOR176W	5.3649
YPR082C	YPR101W	5.3646
YER112W	YMR288W	5.3646
YKL182W	YOL086C	5.3635
YGL031C	YLR342W	5.3629
YCR079W	YPR187W	5.3629
YBL003C	YCR084C	5.3629
YDR037W	YER146W	5.3628
YLR259C	YOL042W	5.3625
YAR042W	YGR086C	5.3609
YLL036C	YMR125W	5.3606
YDR247W	YNL210W	5.36
YML049C	YNL286W	5.3599
YDR237W	YLR189C	5.3588
YKL173W	YLR298C	5.3581
YHL033C	YMR242C	5.3579
YFR028C	YJR063W	5.3579
YER016W	YGR097W	5.3579
YEL012W	YGL013C	5.3579
YKR048C	YNL290W	5.3564
YGR083C	YMR257C	5.3561
YGR083C	YIR026C	5.3561
YER031C	YOR089C	5.3559
YHR196W	YNL075W	5.355
YER125W	YLL029W	5.3547
YBL030C	YJR077C	5.3546
YNL244C	YPL144W	5.3543
YML028W	YNR033W	5.3543
YLR021W	YNL244C	5.3543
YFR050C	YIL009C-A	5.3543
YEL039C	YNL244C	5.3543
YDR141C	YOL001W	5.3543
YHR052W	YKL014C	5.3537
YJL085W	YLR166C	5.3534
YFR024C-A	YOR003W	5.3534
YGR116W	YOR361C	5.3528
YDR235W	YML049C	5.3527
YGL133W	YLR033W	5.351
YAL038W	YCR012W	5.3504
YHL034C	YHR193C	5.3501
YJL041W	YNL153C	5.3499
YLL026W	YLR072W	5.3492
YJL177W	YNL069C	5.3492
YBR139W	YGR129W	5.3481
YDL014W	YPR112C	5.3474
YGL171W	YLR129W	5.3473
YKL138C-A	YKR037C	5.3472
YKL028W	YKL096W-A	5.347
YLR274W	YMR192W	5.3469
YER002W	YHR052W	5.3469
YML075C	YOR051C	5.346
YJL029C	YJL034W	5.346
YIL076W	YML067C	5.346
YGR143W	YML077W	5.346
YGR041W	YML072C	5.346
YDR246W	YGR143W	5.346
YDL080C	YIL142W	5.346
YAL042W	YIL076W	5.346
YAR007C	YMR234W	5.3456
YDL145C	YIL147C	5.3453
YDR295C	YML085C	5.3451
YLL005C	YNL047C	5.3445
YMR091C	YOL087C	5.3438
YDR427W	YMR191W	5.3438
YBR245C	YPL235W	5.3431
YER021W	YLR359W	5.3427
YER021W	YKL165C	5.3427
YER021W	YJR120W	5.3427
YER021W	YJR062C	5.3427
YDR449C	YKL099C	5.3426
YIL070C	YOR158W	5.3424
YBL003C	YBR009C	5.3424
YJL081C	YNL059C	5.3422
YJR140C	YOR038C	5.3419
YFL007W	YKL206C	5.3419
YHR099W	YMR236W	5.3418
YKL081W	YKR101W	5.3412
YDR225W	YLR033W	5.3405
YGR091W	YNR011C	5.3401
YDR359C	YLR399C	5.3401
YDR064W	YLR264W	5.34
YKR048C	YOR272W	5.3391
YFL039C	YKR086W	5.3391
YHR089C	YLR197W	5.339
YBR185C	YDR121W	5.3388
YML126C	YNL119W	5.3387
YDL156W	YIR002C	5.3387
YDL156W	YDR097C	5.3387
YOR284W	YPL206C	5.3378
YJL004C	YNL231C	5.3378
YEL032W	YOL146W	5.3378
YDR489W	YEL032W	5.3378
YDR083W	YLR455W	5.3378
YGR196C	YLL021W	5.3375
YBR260C	YLL021W	5.3375
YBR142W	YOL077C	5.3371
YDR240C	YDR432W	5.3369
YBL058W	YPR179C	5.3366
YBL058W	YNL021W	5.3366
YBL058W	YML064C	5.3366
YEL055C	YLR449W	5.3365
YDR385W	YML028W	5.3365
YDL060W	YDR025W	5.3363
YBL003C	YDR190C	5.3359
YPL043W	YPL211W	5.3356
YJL098W	YPL049C	5.335
YOR206W	YPR016C	5.3348
YOR185C	YPL226W	5.3348
YEL071W	YKL103C	5.3348
YCL039W	YPL018W	5.3346
YBR272C	YPL235W	5.3346
YHR152W	YNL138W	5.3345
YDR188W	YKL104C	5.3338
YHR197W	YOR272W	5.3337
YGL068W	YLR069C	5.3332
YDR502C	YER070W	5.3326
YGL173C	YMR125W	5.3324
YDL040C	YHR004C	5.3322
YLL036C	YML046W	5.3319
YGL157W	YLR429W	5.3312
YCR088W	YGR196C	5.3312
YER052C	YKL127W	5.3311
YDL059C	YER052C	5.3311
YBR129C	YER052C	5.3311
YDR318W	YPR046W	5.3306
YBR264C	YFR004W	5.3301
YBL075C	YDL095W	5.3299
YHR166C	YKR064W	5.3297
YKL085W	YLR304C	5.3291
YDL098C	YMR004W	5.328
YBR082C	YLR304C	5.328
YOL144W	YOR108W	5.3277
YOL058W	YOL144W	5.3277
YNL104C	YOL144W	5.3277
YDL160C	YHR165C	5.3277
YDR064W	YOR096W	5.3272
YFR013W	YKR001C	5.3263
YER165W	YOL120C	5.3262
YAL029C	YDR171W	5.3262
YBL030C	YIL124W	5.3261
YBR282W	YDR462W	5.3259
YLR432W	YPR088C	5.3257
YGR240C	YNL045W	5.3249
YBR191W	YCR031C	5.3237
YGL019W	YLR223C	5.3235
YGL019W	YKL064W	5.3235
YCL011C	YGL049C	5.323
YOR101W	YPL237W	5.3229
YAL034C	YPR184W	5.3228
YGL174W	YLR275W	5.3225
YDR450W	YNL301C	5.3224
YDR229W	YFL008W	5.3224
YDR131C	YML088W	5.3224
YAL047C	YFL008W	5.3224
YKR031C	YNL071W	5.3223
YHR196W	YLR409C	5.3223
YDR430C	YNL071W	5.3223
YGR264C	YHR086W	5.3222
YDR381W	YOL070C	5.3221
YER041W	YLR449W	5.3219
YAL016W	YOL113W	5.3219
YAL016W	YLR450W	5.3219
YMR315W	YNL135C	5.3215
YNL248C	YOR116C	5.3212
YOL077C	YPL012W	5.3207
YGR020C	YNL099C	5.3202
YGR020C	YNL032W	5.3202
YBR079C	YDR211W	5.3201
YJL011C	YOL021C	5.3195
YMR243C	YOL098C	5.3185
YMR209C	YOL098C	5.3185
YER177W	YOR220W	5.3175
YER177W	YLR256W	5.3175
YER177W	YGL181W	5.3175
YER172C	YIL144W	5.3175
YCR082W	YDR201W	5.3175
YBR059C	YER177W	5.3175
YLL011W	YMR128W	5.3165
YBR247C	YGR128C	5.3161
YNL107W	YPR080W	5.3153
YDR108W	YHR200W	5.3147
YDR391C	YGR192C	5.3146
YJL167W	YNL250W	5.3141
YGL173C	YPR189W	5.3134
YER094C	YIL075C	5.3117
YDR035W	YJL026W	5.3115
YER126C	YKR081C	5.3104
YBR251W	YGR061C	5.3097
YAL005C	YOR164C	5.3091
YER091C	YNR030W	5.3089
YIL107C	YOR259C	5.3088
YLR264W	YPL204W	5.3087
YGL245W	YJR109C	5.3085
YBR084W	YHR064C	5.3085
YER112W	YOR234C	5.3082
YDR069C	YMR304W	5.308
YDR069C	YLR436C	5.308
YBR202W	YOL146W	5.308
YBR202W	YEL032W	5.308
YBR202W	YDR489W	5.308
YAR014C	YLR258W	5.308
YMR033W	YNL307C	5.3069
YDL082W	YNL069C	5.3052
YLR284C	YOR133W	5.3047
YAL029C	YDR388W	5.3043
YER174C	YML036W	5.3039
YMR058W	YPL149W	5.3036
YFL039C	YMR109W	5.3036
YNL110C	YNL182C	5.3033
YBR229C	YFR051C	5.3026
YJL080C	YPL048W	5.302
YAL005C	YJL102W	5.3008
YKR081C	YOR294W	5.3006
YDR174W	YNL216W	5.3
YDR035W	YLR355C	5.3
YKL210W	YKR082W	5.2997
YIL137C	YKL210W	5.2997
YEL055C	YJL039C	5.2997
YBL004W	YLR409C	5.2984
YDR060W	YGL120C	5.2977
YHR197W	YKR081C	5.2969
YNL067W	YPL143W	5.2967
YGR195W	YNL248C	5.2964
YJL115W	YLR347C	5.2959
YBL074C	YKL012W	5.2958
YLR435W	YOR065W	5.2955
YLR435W	YNL207W	5.2955
YLR107W	YLR435W	5.2955
YGL189C	YLR435W	5.2955
YLL036C	YPR152C	5.2954
YGR090W	YMR172W	5.2949
YDR295C	YFL010C	5.2947
YPL090C	YPR175W	5.2945
YJL168C	YPR093C	5.2939
YJR041C	YOR108W	5.2921
YJR041C	YOL058W	5.2921
YJR041C	YNL104C	5.2921
YHR027C	YMR241W	5.2905
YKL157W	YLR277C	5.2902
YBL004W	YGR145W	5.2896
YMR033W	YPL082C	5.2893
YDR416W	YGR129W	5.2888
YBR247C	YHR148W	5.2887
YBR181C	YOL040C	5.2887
YJL036W	YNL243W	5.2886
YDR425W	YNL243W	5.2886
YJR134C	YNL118C	5.2883
YHR098C	YNL118C	5.2883
YGL197W	YNL118C	5.2883
YMR290C	YNL308C	5.2861
YGL241W	YMR092C	5.2861
YER172C	YLR371W	5.2861
YDR300C	YML095C	5.2861
YAL005C	YIL115C	5.2856
YPL225W	YPR173C	5.2855
YER073W	YHR087W	5.2855
YKR096W	YLR384C	5.2834
YIL151C	YLR384C	5.2834
YBL075C	YML016C	5.2834
YOR272W	YPR016C	5.2824
YDR001C	YNR047W	5.2822
YBR079C	YLR215C	5.2822
YJR135C	YKR054C	5.2821
YDL160C	YGR075C	5.2819
YKL125W	YNL119W	5.2817
YCR009C	YMR083W	5.2817
YER127W	YPL126W	5.2816
YBR258C	YKL018W	5.2814
YDR028C	YER016W	5.2805
YMR246W	YPL206C	5.2791
YLR438C-A	YMR080C	5.279
YFL003C	YKL214C	5.2782
YDR385W	YNL201C	5.2781
YDR443C	YGR252W	5.278
YBL071W-A	YHR103W	5.278
YAL020C	YBL071W-A	5.278
YAL027W	YPL004C	5.2765
YBR196C	YPL109C	5.2762
YOR319W	YPL213W	5.2759
YER078C	YPL129W	5.2753
YBL075C	YPL263C	5.2751
YDR108W	YGR143W	5.2747
YDR341C	YKL216W	5.2743
YDR170C	YER100W	5.2743
YER054C	YLR258W	5.2741
YCR057C	YGR145W	5.2741
YBR087W	YFR004W	5.2739
YFR055W	YOR375C	5.2731
YBR093C	YOR375C	5.2731
YAR071W	YOR375C	5.2731
YBR248C	YFL037W	5.273
YER052C	YML032C	5.2725
YPL131W	YPR102C	5.2723
YKR048C	YPR119W	5.2721
YHR165C	YMR268C	5.2717
YJR050W	YPL151C	5.2715
YGR090W	YJL074C	5.2715
YJL081C	YOR046C	5.2712
YGR156W	YHL033C	5.271
YLR044C	YOR245C	5.2706
YLR044C	YLR134W	5.2706
YJR002W	YOR287C	5.2706
YDL043C	YML046W	5.2703
YBR245C	YPL128C	5.2689
YLR423C	YOR361C	5.2684
YBR181C	YDL083C	5.2684
YBR081C	YMR019W	5.2684
YBR081C	YGR002C	5.2684
YBR081C	YER015W	5.2684
YBR081C	YDR146C	5.2684
YIL068C	YJL085W	5.2679
YBR155W	YGR234W	5.2679
YBL026W	YML049C	5.2672
YGR013W	YPR082C	5.2669
YDR365C	YGR145W	5.2662
YDR289C	YLR045C	5.2657
YDR240C	YGR264C	5.2656
YBR279W	YPL084W	5.2655
YDR055W	YOL117W	5.2649
YBR139W	YOR323C	5.2649
YBR001C	YOL016C	5.2649
YML028W	YNL282W	5.2646
YCR063W	YKL173W	5.2641
YBL074C	YIL061C	5.2639
YBR114W	YMR190C	5.2619
YBR114W	YLR234W	5.2619
YBR114W	YJR144W	5.2619
YER021W	YOR069W	5.2617
YER021W	YJL204C	5.2617
YDL145C	YMR304W	5.2613
YLR079W	YMR199W	5.2611
YKL028W	YNR029C	5.2611
YDR150W	YGR086C	5.2611
YDR150W	YFL016C	5.2611
YKR031C	YNL290W	5.261
YHR191C	YLL040C	5.261
YLR003C	YOR145C	5.2607
YKL022C	YLR102C	5.2594
YNL024C	YPR047W	5.259
YLR298C	YMR288W	5.259
YMR125W	YPR101W	5.2588
YAL035W	YPL009C	5.2584
YEL030W	YIL103W	5.2581
YNR016C	YPL235W	5.2577
YMR049C	YNL132W	5.2574
YGR252W	YHL047C	5.2573
YCR057C	YKL099C	5.2565
YER093C	YMR186W	5.2562
YMR229C	YNL308C	5.2561
YCL059C	YPR016C	5.256
YKL099C	YLR409C	5.2555
YER133W	YPL198W	5.2554
YGL128C	YGR074W	5.2548
YDL147W	YKL184W	5.2545
YMR185W	YPL235W	5.2544
YHR197W	YNL061W	5.2544
YPL106C	YPR031W	5.2542
YIL094C	YPL140C	5.2541
YDR155C	YMR186W	5.2539
YHR196W	YKL210W	5.2533
YGL013C	YKL113C	5.2528
YDR141C	YGR233C	5.2528
YJL087C	YOR123C	5.2527
YGR179C	YOL054W	5.2525
YML049C	YOR159C	5.2519
YHR196W	YOR145C	5.2516
YLR347C	YNL312W	5.2513
YJR064W	YNL212W	5.251
YJL128C	YNR058W	5.251
YDR300C	YNL001W	5.2508
YJR084W	YLR298C	5.2506
YIL104C	YMR205C	5.2506
YGR209C	YMR308C	5.2506
YER047C	YGR256W	5.2506
YCL028W	YOR217W	5.2505
YLL026W	YLR216C	5.2501
YNL005C	YPR100W	5.2495
YJR066W	YLR373C	5.2493
YGR204W	YOR335C	5.2488
YJL076W	YPL255W	5.2487
YER177W	YOL013C	5.2487
YER018C	YER172C	5.2487
YGR084C	YNR036C	5.2463
YLR275W	YMR132C	5.2459
YMR229C	YPL126W	5.2453
YHR102W	YKL189W	5.2452
YGR041W	YLR447C	5.2452
YFL053W	YLR447C	5.2452
YDR316W	YLR447C	5.2452
YNL307C	YNR014W	5.2448
YBR152W	YPR082C	5.2445
YNL084C	YOR181W	5.2443
YBL075C	YOL078W	5.2443
YGR074W	YJR050W	5.2441
YER062C	YKR084C	5.2427
YBL022C	YER062C	5.2427
YDR308C	YDR448W	5.2417
YJL124C	YJR145C	5.2411
YER126C	YNL002C	5.2405
YAL029C	YHR114W	5.2403
YAL029C	YBL007C	5.2403
YBR035C	YFL030W	5.2397
YBL058W	YMR276W	5.2396
YBL058W	YER057C	5.2396
YOR229W	YOR283W	5.2394
YGR270W	YOR361C	5.239
YLR325C	YPL001W	5.2389
YKR064W	YNL005C	5.2368
YJL061W	YNL153C	5.2368
YCL016C	YMR078C	5.2368
YIR012W	YOR261C	5.2367
YGR214W	YOR022C	5.2365
YGL045W	YOR253W	5.236
YML071C	YNL265C	5.2359
YGR194C	YML071C	5.2359
YBR264C	YFR052W	5.2352
YLR276C	YLR449W	5.2338
YGL137W	YLR051C	5.2335
YDL167C	YNL016W	5.2335
YBL022C	YNL329C	5.2335
YBL022C	YML029W	5.2335
YDR194C	YLR175W	5.2326
YKL081W	YLL026W	5.2319
YGR054W	YLL045C	5.2317
YDR258C	YOR326W	5.2314
YGR090W	YPL198W	5.2312
YCL054W	YKL172W	5.2309
YDL031W	YFR001W	5.2301
YLR448W	YNL317W	5.2298
YJR101W	YOR204W	5.2298
YER068W	YOR257W	5.2296
YDR059C	YOR257W	5.2296
YDR101C	YNL110C	5.2291
YDL063C	YKL009W	5.2279
YDL136W	YHL001W	5.2268
YER164W	YOR003W	5.2266
YCR081W	YGR252W	5.2266
YGR214W	YMR230W	5.2262
YKL088W	YMR172W	5.2261
YGL115W	YGR152C	5.2261
YFR002W	YIL115C	5.2261
YIL069C	YLR170C	5.2258
YKL104C	YLR180W	5.2257
YCR082W	YGL061C	5.2257
YBR245C	YMR070W	5.2257
YHR143W-A	YPL255W	5.225
YLR325C	YNR053C	5.2248
YDR412W	YKL172W	5.224
YGR254W	YNL220W	5.2239
YMR297W	YNR038W	5.2236
YIL053W	YML105C	5.2236
YFR040W	YKL029C	5.223
YGR192C	YNL155W	5.2223
YGR245C	YNL110C	5.2222
YHR064C	YNL209W	5.2221
YKL125W	YML126C	5.2212
YFL008W	YPL207W	5.2212
YHR191C	YOL094C	5.2211
YIL076W	YLR051C	5.2209
YCR009C	YCR088W	5.2209
YCR092C	YMR190C	5.2204
YCR092C	YLR234W	5.2204
YCR092C	YJR144W	5.2204
YPL161C	YPL240C	5.2201
YBR247C	YMR093W	5.22
YBR247C	YDR449C	5.22
YGL103W	YNL069C	5.2198
YGL128C	YJL203W	5.2196
YDR337W	YOR243C	5.2188
YFL024C	YPL169C	5.2179
YJL034W	YPL106C	5.2177
YDR386W	YNL312W	5.2177
YCR012W	YMR186W	5.2177
YGL117W	YJR022W	5.2173
YDR359C	YLR113W	5.2173
YDL111C	YJR022W	5.2173
YCL050C	YJR022W	5.2173
YDL193W	YOR185C	5.2171
YDR129C	YLR019W	5.2169
YMR259C	YOR261C	5.2167
YDR490C	YKL166C	5.2167
YGR278W	YPR101W	5.2165
YGL106W	YNL208W	5.2165
YBR233W-A	YKL052C	5.2157
YDL111C	YLR398C	5.2152
YCL037C	YLR430W	5.2152
YDL003W	YKR064W	5.2149
YKR061W	YML032C	5.2146
YJL139C	YML032C	5.2146
YNL252C	YNR022C	5.2134
YNL064C	YPL061W	5.2129
YJL154C	YOR048C	5.2122
YGL246C	YJL154C	5.2122
YDR432W	YPL049C	5.2122
YKR048C	YMR237W	5.2121
YER094C	YPR108W	5.2105
YDR427W	YOL022C	5.2105
YDR427W	YLR341W	5.2105
YDR427W	YGL027C	5.2105
YDR427W	YER041W	5.2105
YKL145W	YPR103W	5.2103
YDR050C	YLR355C	5.21
YDR311W	YPR025C	5.2095
YCR057C	YPR112C	5.2095
YKR006C	YOR150W	5.2086
YKL167C	YOR150W	5.2086
YGR143W	YMR218C	5.2082
YGR143W	YKR068C	5.2082
YDR407C	YGR143W	5.2082
YBR254C	YGR143W	5.2082
YGR178C	YIL053W	5.2076
YER087W	YLR430W	5.2074
YDL076C	YER088C	5.2074
YDL185W	YNR016C	5.2071
YLR072W	YOR375C	5.2066
YHR117W	YOR375C	5.2066
YGL106W	YGL141W	5.2063
YER173W	YLR413W	5.2063
YDR462W	YGR220C	5.2058
YGL008C	YPL170W	5.2053
YER125W	YJR005W	5.2053
YDR237W	YMR225C	5.2052
YDR237W	YML009C	5.2052
YBR198C	YER148W	5.2051
YER082C	YGL171W	5.2048
YDR155C	YFR053C	5.2041
YBR103W	YOR028C	5.2041
YBR159W	YDL136W	5.2038
YNL178W	YPR080W	5.2026
YBR142W	YPL211W	5.2023
YHR121W	YOL078W	5.2018
YDR172W	YLR344W	5.2018
YDR172W	YHR015W	5.2018
YDR074W	YPR035W	5.2018
YBR135W	YLR109W	5.2018
YBL003C	YMR033W	5.2017
YCL054W	YGR103W	5.2016
YBR109C	YHR023W	5.2007
YIL070C	YMR308C	5.2003
YGR274C	YOL148C	5.2003
YCL059C	YGR162W	5.2002
YDL081C	YNL069C	5.1999
YBR080C	YHR200W	5.1997
YFR010W	YLR319C	5.1995
YFL002C	YHR197W	5.1986
YKL180W	YPL143W	5.1982
YDR240C	YLR347C	5.1981
YJL095W	YNL007C	5.1978
YBR044C	YDR069C	5.197
YGL133W	YMR072W	5.1964
YDR124W	YGR165W	5.1963
YOL041C	YOR272W	5.1961
YDR060W	YNL069C	5.196
YLR192C	YOR204W	5.1945
YDL082W	YGR034W	5.1945
YHR066W	YKL082C	5.1939
YCR082W	YKR037C	5.1934
YGL195W	YML105C	5.1928
YFR002W	YJL039C	5.1923
YHR164C	YOL090W	5.1922
YDR397C	YPL082C	5.1911
YNL049C	YOR117W	5.1908
YLL026W	YOR217W	5.1908
YGL048C	YNL049C	5.1908
YER089C	YLR058C	5.1904
YGL127C	YLR071C	5.1897
YDR060W	YFR001W	5.1889
YKL206C	YML130C	5.1882
YGR284C	YKL206C	5.1882
YDR028C	YDR301W	5.1874
YLR249W	YOR165W	5.1865
YJR142W	YLR249W	5.1865
YGL171W	YPR144C	5.186
YDL075W	YGR027C	5.186
YEL019C	YOL034W	5.1859
YDR211W	YMR257C	5.1859
YDR211W	YIR026C	5.1859
YDR188W	YJR121W	5.1846
YCL059C	YKR048C	5.184
YDL126C	YGL246C	5.1839
YDR155C	YJR104C	5.1834
YLR325C	YOL054W	5.1829
YDL150W	YFR008W	5.1827
YER177W	YMR120C	5.1826
YKL022C	YPL129W	5.1822
YDR204W	YKL101W	5.182
YML057W	YOR259C	5.1813
YDL047W	YPL049C	5.1812
YGR005C	YPR190C	5.1804
YKL010C	YLR304C	5.1799
YFL016C	YGL205W	5.1792
YER182W	YFL016C	5.1792
YEL062W	YFL016C	5.1792
YDR170C	YGL208W	5.1792
YDL112W	YGR103W	5.1791
YHL033C	YIL133C	5.179
YHL034C	YJL197W	5.1777
YGR200C	YNL209W	5.1773
YGL103W	YHL001W	5.177
YBR065C	YGL120C	5.1769
YGR027C	YNL119W	5.1766
YFR030W	YJR137C	5.1764
YCR003W	YDR322W	5.1761
YER155C	YLL021W	5.1754
YDR028C	YER155C	5.1754
YKR081C	YLR175W	5.1744
YDL099W	YPR181C	5.1739
YBR048W	YOL010W	5.1733
YJR066W	YOL135C	5.1732
YGL242C	YPL198W	5.173
YMR308C	YNL189W	5.1726
YDR104C	YDR254W	5.1725
YNL230C	YOL041C	5.1722
YDR447C	YGL103W	5.1722
YDR365C	YLR314C	5.1711
YGR158C	YLR398C	5.171
YBL022C	YPL049C	5.1705
YLL024C	YLL026W	5.1704
YGL206C	YOR155C	5.1702
YKL012W	YLL036C	5.17
YBR247C	YDL063C	5.169
YIL075C	YKL007W	5.1686
YDL165W	YNR052C	5.1686
YBR245C	YHR056C	5.1682
YGR013W	YPR101W	5.1681
YFL037W	YJL159W	5.1679
YBR121C	YKL157W	5.1679
YGR240C	YKL081W	5.1678
YER002W	YLR002C	5.1671
YAL012W	YJL167W	5.1655
YKL145W	YOR069W	5.1647
YGR159C	YNL182C	5.1647
YHR016C	YPR171W	5.1642
YHR016C	YNL106C	5.1642
YER147C	YFL008W	5.1642
YEL071W	YPL262W	5.1633
YBR084W	YIL018W	5.1633
YBR081C	YHR009C	5.1633
YDL192W	YHR188C	5.1623
YDL134C	YOR386W	5.1621
YGR143W	YGR166W	5.1611
YDR180W	YJL074C	5.1606
YDR099W	YHR064C	5.1606
YLR208W	YML130C	5.1599
YHL015W	YJL080C	5.1597
YKL142W	YLR287C	5.1596
YDR314C	YPR032W	5.1596
YAL062W	YOR370C	5.1596
YGL191W	YLR335W	5.1595
YDL147W	YIR012W	5.159
YGL120C	YMR116C	5.1587
YPL064C	YPL151C	5.1585
YDL002C	YGR285C	5.1582
YDR225W	YLR357W	5.1578
YDR016C	YKR083C	5.1577
YBR245C	YDR224C	5.1572
YER116C	YOR058C	5.1569
YDR152W	YGL061C	5.1569
YDR237W	YNL284C	5.1567
YHR147C	YOR150W	5.1559
YGL135W	YGR085C	5.1551
YMR140W	YOR335C	5.1548
YBL058W	YDR330W	5.1547
YCL014W	YPR169W	5.1544
YDL175C	YER087W	5.1542
YER172C	YMR125W	5.1535
YBL041W	YDL147W	5.1533
YDR251W	YNL271C	5.1531
YDL160C	YDR473C	5.1531
YER126C	YHR052W	5.153
YHR008C	YML095C	5.1529
YLR300W	YOL126C	5.1528
YER178W	YJL128C	5.1527
YDL148C	YPL235W	5.1527
YBR105C	YDL225W	5.1527
YGL004C	YHR027C	5.1525
YBR205W	YEL002C	5.1525
YGL121C	YPL237W	5.1523
YCR079W	YOR210W	5.152
YDR171W	YJR010W	5.1519
YDL060W	YPL090C	5.1501
YDL014W	YER082C	5.1497
YAL035W	YGL123W	5.1497
YLR359W	YOR117W	5.1496
YKL165C	YOR117W	5.1496
YJR120W	YOR117W	5.1496
YJR062C	YOR117W	5.1496
YLR398C	YNL232W	5.1493
YOR158W	YPR185W	5.1488
YGL180W	YOR158W	5.1488
YJL035C	YOR308C	5.1482
YNL069C	YOR096W	5.1472
YJR032W	YNL139C	5.1461
YBR215W	YLR392C	5.1461
YGR143W	YOR115C	5.1459
YER131W	YOR299W	5.1459
YER131W	YMR237W	5.1459
YER131W	YLR330W	5.1459
YDR472W	YGR143W	5.1459
YHR164C	YML032C	5.1458
YDR028C	YOL063C	5.1458
YDR028C	YJL047C	5.1458
YOR259C	YPL088W	5.1454
YOL040C	YOR198C	5.1451
YBL072C	YDR064W	5.145
YER165W	YPL184C	5.1444
YAL038W	YHL025W	5.1444
YBL002W	YDR224C	5.1433
YOL123W	YOR176W	5.143
YMR193W	YOR150W	5.143
YIL101C	YOL004W	5.1423
YDR473C	YGL128C	5.1421
YDL105W	YDL148C	5.142
YCL032W	YNL262W	5.1419
YBL087C	YNL002C	5.1419
YKR029C	YOR028C	5.1418
YDR098C	YNL254C	5.1418
YDL175C	YOR204W	5.1415
YBR245C	YLR347C	5.1415
YMR315W	YPL154C	5.1414
YBR088C	YDL102W	5.141
YGR075C	YMR240C	5.1403
YJR084W	YPR101W	5.14
YGL106W	YGR086C	5.1386
YBR079C	YOR260W	5.138
YEL022W	YMR012W	5.1377
YFR001W	YMR049C	5.1376
YDR383C	YGR006W	5.1375
YDR104C	YDR383C	5.1375
YNL004W	YOL115W	5.1374
YIL079C	YNL004W	5.1374
YCL010C	YML024W	5.1374
YDR190C	YHR084W	5.1372
YFL045C	YHR128W	5.137
YEL002C	YKL181W	5.1369
YER159C	YLR355C	5.1365
YLR325C	YPR171W	5.1363
YIL035C	YKL064W	5.1361
YDL043C	YDL087C	5.1356
YBL075C	YKL088W	5.1354
YFR019W	YNL251C	5.1353
YJR082C	YNL136W	5.1351
YKL108W	YNL189W	5.1341
YAL029C	YKL124W	5.1338
YNL037C	YPL140C	5.1336
YHR147C	YKR085C	5.1333
YOL010W	YPR029C	5.1323
YLR328W	YPR029C	5.1323
YLR328W	YOL010W	5.1323
YLR199C	YPR103W	5.1323
YKR018C	YLR392C	5.1323
YKL135C	YOL010W	5.1323
YKL135C	YLR328W	5.1323
YIL159W	YPL032C	5.1323
YGR136W	YLR392C	5.1323
YGR136W	YKR018C	5.1323
YGR130C	YGR159C	5.1323
YFR017C	YPL032C	5.1323
YFR017C	YIL159W	5.1323
YER177W	YPL032C	5.1323
YER177W	YIL159W	5.1323
YER114C	YPL032C	5.1323
YER114C	YIL159W	5.1323
YER114C	YFR017C	5.1323
YER114C	YER177W	5.1323
YDR017C	YPL032C	5.1323
YDR017C	YIL159W	5.1323
YDR017C	YFR017C	5.1323
YDR017C	YER114C	5.1323
YDR006C	YPL032C	5.1323
YDR006C	YIL159W	5.1323
YDR006C	YFR017C	5.1323
YDR006C	YER177W	5.1323
YDR006C	YER114C	5.1323
YDR006C	YDR017C	5.1323
YDR001C	YPL032C	5.1323
YDR001C	YIL159W	5.1323
YDR001C	YFR017C	5.1323
YDR001C	YER114C	5.1323
YDR001C	YDR017C	5.1323
YDR001C	YDR006C	5.1323
YDL117W	YPL032C	5.1323
YDL117W	YIL159W	5.1323
YDL117W	YFR017C	5.1323
YDL117W	YER114C	5.1323
YDL117W	YDR017C	5.1323
YDL117W	YDR006C	5.1323
YDL117W	YDR001C	5.1323
YBL041W	YLR199C	5.1323
YBL034C	YPL032C	5.1323
YBL034C	YIL159W	5.1323
YBL034C	YFR017C	5.1323
YBL034C	YER177W	5.1323
YBL034C	YER114C	5.1323
YBL034C	YDR017C	5.1323
YBL034C	YDR006C	5.1323
YBL034C	YDR001C	5.1323
YBL034C	YDL117W	5.1323
YOL094C	YPL194W	5.132
YMR311C	YOR054C	5.132
YNL229C	YPL235W	5.1319
YER040W	YPL235W	5.1319
YDL160C	YKR022C	5.1318
YLR044C	YLR447C	5.1309
YGL120C	YMR213W	5.1301
YDR060W	YML074C	5.1301
YFL013C	YGR285C	5.1295
YKR027W	YKR048C	5.1282
YDL030W	YDR378C	5.128
YER164W	YMR172W	5.1279
YBR272C	YLL022C	5.1279
YDR071C	YDR186C	5.1277
YBL074C	YPR057W	5.1275
YJR045C	YKL117W	5.1273
YGR266W	YJR045C	5.1273
YER107C	YMR308C	5.1265
YLR197W	YPR144C	5.1251
YAL038W	YBR025C	5.1247
YLR147C	YLR424W	5.1246
YJL063C	YNL306W	5.1246
YDL005C	YNL236W	5.1245
YER172C	YOL069W	5.1239
YDR436W	YGR159C	5.1237
YLR039C	YNL244C	5.1235
YGL137W	YNR016C	5.123
YDL014W	YDR012W	5.1229
YNL056W	YNR054C	5.1224
YFL018C	YOL058W	5.1222
YBL058W	YEL037C	5.1222
YBL058W	YDL190C	5.1222
YBL038W	YHR147C	5.1221
YFR001W	YOL077C	5.122
YEL060C	YER052C	5.1219
YIL126W	YPL110C	5.1216
YOL146W	YPL153C	5.1213
YEL032W	YPL153C	5.1213
YDR489W	YPL153C	5.1213
YHR128W	YNR012W	5.1211
YDR460W	YER062C	5.121
YKL104C	YOR133W	5.1197
YER006W	YHR085W	5.1195
YDL111C	YKR025W	5.1188
YBR011C	YMR267W	5.1183
YFR001W	YLR002C	5.1177
YDL126C	YLR447C	5.1174
YDR028C	YHR052W	5.1171
YDL148C	YEL019C	5.1169
YDL005C	YLR071C	5.1168
YOR187W	YPL048W	5.1166
YMR093W	YOR145C	5.1161
YKL214C	YPR161C	5.1161
YKL214C	YLR226W	5.1161
YGR027C	YML126C	5.1161
YFL003C	YPR161C	5.1161
YFL003C	YLR226W	5.1161
YDL142C	YHR019C	5.1161
YLR075W	YLR340W	5.1154
YER012W	YHR200W	5.1154
YDL043C	YLR382C	5.1153
YER029C	YOR319W	5.1149
YDR418W	YHL001W	5.1146
YKL014C	YMR290C	5.1144
YDL105W	YLR314C	5.1144
YDL014W	YPR144C	5.1137
YBR142W	YFR001W	5.1137
YGL253W	YPR164W	5.1134
YGR020C	YNR050C	5.1132
YAL062W	YKR014C	5.1132
YDL043C	YIL061C	5.1126
YBR133C	YPR119W	5.1121
YDR328C	YIL071C	5.112
YBL056W	YGR205W	5.1119
YDR388W	YGL106W	5.1113
YDR473C	YLR438C-A	5.1105
YBL017C	YMR125W	5.1105
YAL048C	YJR009C	5.1102
Q0080	YLR172C	5.1101
YHR089C	YOL123W	5.1098
YMR098C	YNL005C	5.1097
YCL014W	YNL005C	5.1097
YDR064W	YOL127W	5.1095
YFL039C	YNR029C	5.1094
YHR196W	YJL069C	5.1092
YBR246W	YOR133W	5.1074
YFR013W	YKR048C	5.1064
YFR013W	YGL241W	5.1064
YDL147W	YMR259C	5.1063
YER029C	YJR084W	5.1059
YMR138W	YNL112W	5.1056
YLR115W	YOR250C	5.1052
YGL044C	YLR115W	5.1052
YDR064W	YOR145C	5.1042
YDL209C	YMR125W	5.1039
YLR127C	YNL005C	5.1037
YGR240C	YOR375C	5.1037
YAR014C	YCR073W-A	5.1037
YHL014C	YKR059W	5.1036
YGR215W	YNR036C	5.1036
YDL132W	YOL063C	5.1035
YDR124W	YIL093C	5.1034
YFR024C-A	YNL084C	5.103
YFL039C	YKL057C	5.103
YGL049C	YPL178W	5.1027
YDL175C	YDR394W	5.1024
YMR303C	YNL245C	5.102
YCR014C	YDL007W	5.1017
YDL040C	YGL045W	5.1015
YKL067W	YLL040C	5.1008
YBR127C	YNL064C	5.1002
YDR214W	YDR394W	5.0983
YDR430C	YFL018C	5.0972
YDR037W	YMR080C	5.0972
YBR263W	YHR193C	5.0972
YOR310C	YPL126W	5.0969
YFL039C	YLR225C	5.0968
YBR167C	YNL246W	5.0966
YBR119W	YPR178W	5.0965
YML093W	YPL126W	5.0964
YDR418W	YGL147C	5.0959
YKL182W	YLR249W	5.0958
YGL121C	YLR442C	5.0954
YJL080C	YLL027W	5.0953
YCR053W	YDR158W	5.0953
YNL002C	YOR063W	5.0952
YFR001W	YKL172W	5.095
YGL171W	YHR148W	5.0939
YLR175W	YOL139C	5.0937
Q0080	YOR160W	5.0934
YOR341W	YPL255W	5.0933
YCL008C	YOR253W	5.0933
YLR268W	YLR342W	5.093
YMR257C	YOR260W	5.0927
YIR026C	YOR260W	5.0927
YIL046W	YJR090C	5.0927
YDL164C	YJR090C	5.0927
YGR179C	YKR054C	5.0926
Q0140	YGR084C	5.0926
YOR173W	YPR184W	5.0924
YOR173W	YPL247C	5.0924
YKR001C	YOR173W	5.0924
YDR341C	YIL149C	5.0924
YLR106C	YNL329C	5.0918
YLR106C	YML029W	5.0918
YER052C	YKL081W	5.0916
YER087W	YOL115W	5.0915
YBR202W	YPL153C	5.0915
YDR416W	YJL203W	5.0912
YLR335W	YNL282W	5.0907
YIL142W	YKR048C	5.0905
YGR162W	YJL080C	5.0905
YBL041W	YDR427W	5.0903
YHL033C	YOL127W	5.09
YBR088C	YJR043C	5.0899
YER148W	YMR236W	5.0898
YEL019C	YLR314C	5.0894
YDR394W	YLR319C	5.0893
YML074C	YOR206W	5.0889
YGL207W	YLR005W	5.088
YML057W	YMR255W	5.0878
YGR205W	YOR086C	5.0873
YNL224C	YOR098C	5.0872
YGL099W	YLR074C	5.087
YCR012W	YNL192W	5.0866
YDR448W	YPL042C	5.0865
YBR189W	YGL030W	5.0863
YFL045C	YOL062C	5.0849
YJL201W	YPR171W	5.0844
YJL201W	YNL106C	5.0844
YHR114W	YPR171W	5.0844
YHR114W	YNL106C	5.0844
YHR016C	YJL201W	5.0844
YHR016C	YHR114W	5.0844
YDR283C	YML091C	5.0844
YGL122C	YOR176W	5.0842
YBR017C	YOR176W	5.0842
YGL004C	YHR200W	5.0841
YIL103W	YLR043C	5.0836
YMR241W	YOR317W	5.0834
YFR031C	YJR032W	5.0831
YLR189C	YNL005C	5.083
YIL036W	YNL094W	5.0828
YIL036W	YMR201C	5.0828
YJR002W	YPR112C	5.0822
YFR019W	YNL187W	5.0822
YFR019W	YNL101W	5.0822
YLL036C	YMR240C	5.082
YMR268C	YNL118C	5.0817
YGR129W	YMR213W	5.0817
YBR188C	YGR129W	5.0817
YBR205W	YFR044C	5.0815
YJL011C	YNL232W	5.081
YLR249W	YPL231W	5.0807
YGL120C	YML049C	5.0804
YLR276C	YPL043W	5.0794
YLL036C	YLR147C	5.0794
YDR460W	YHR042W	5.079
YIL133C	YMR142C	5.0787
YFR052W	YKL184W	5.0787
YER002W	YKL009W	5.0787
YBR193C	YGR252W	5.0785
YHR027C	YLR359W	5.0784
YHR027C	YKL165C	5.0784
YHR027C	YJR120W	5.0784
YHR027C	YJR062C	5.0784
YMR209C	YPR040W	5.0778
YGL020C	YML048W	5.0778
YDR060W	YPL093W	5.0778
YBL052C	YNL107W	5.0778
YER120W	YLR002C	5.0776
YGR054W	YPL266W	5.0775
YKL152C	YLR153C	5.0773
YER110C	YPL050C	5.0772
YDR137W	YER136W	5.0772
YLR175W	YOR310C	5.0771
YKL166C	YOR089C	5.0771
YJR002W	YKL035W	5.077
YER048C	YKL089W	5.0763
YGR034W	YMR194W	5.0761
YDL185W	YMR027W	5.0761
YDL185W	YLR090W	5.0761
YIL105C	YNL106C	5.0754
YMR205C	YMR262W	5.0753
YML019W	YML130C	5.0751
YBL035C	YIL046W	5.075
YBL035C	YDL164C	5.075
YBL058W	YOR201C	5.0749
YCL011C	YLR432W	5.0738
YMR158W	YNL247W	5.0737
YDR032C	YNL250W	5.0737
YER172C	YNL290W	5.0735
YDR142C	YKL103C	5.0731
YBR245C	YOR116C	5.0725
YBL002W	YDR174W	5.0714
YGR097W	YLR436C	5.071
YDR538W	YGR097W	5.071
YDL066W	YGR097W	5.071
YOR056C	YOR123C	5.0709
YML024W	YMR223W	5.0709
YBL071W-A	YGR258C	5.0709
YAL035W	YJR007W	5.0709
YHR165C	YNL246W	5.0696
YJR084W	YPL012W	5.0695
YJL204C	YOR117W	5.0692
YDR450W	YLR287C-A	5.0686
YLR288C	YOR005C	5.0683
YDR002W	YJR074W	5.0683
YDR471W	YHL033C	5.0679
YDR427W	YFR050C	5.067
YMR128W	YNR046W	5.0669
YFR028C	YJL076W	5.0669
YER016W	YER177W	5.0669
YHR033W	YLR216C	5.0667
YHL030W	YKL152C	5.0665
YFR052W	YOR027W	5.0665
YDL014W	YNR054C	5.0662
YBR212W	YIR037W	5.0656
YDR415C	YLL024C	5.0642
YGL179C	YGL241W	5.0635
YIL129C	YLR447C	5.0634
YFR021W	YGL190C	5.0634
YDR477W	YMR104C	5.0634
YDR477W	YGL158W	5.0634
YDR237W	YPL183W-A	5.0634
YBR142W	YOR310C	5.0634
YBL080C	YDR477W	5.0634
YER020W	YPL036W	5.0633
YDR353W	YNL278W	5.0629
YHR014W	YOR136W	5.0624
YOL006C	YOR003W	5.0623
YDR052C	YHR200W	5.0623
YGR056W	YKR001C	5.061
YJL039C	YJL041W	5.0591
YGR027C	YKL125W	5.0591
YBR272C	YEL056W	5.0591
YAL035W	YOL078W	5.0584
YJL183W	YLR450W	5.0577
YJL183W	YKR031C	5.0577
YCL010C	YOR023C	5.0576
YDL147W	YPR103W	5.0575
YDR448W	YGR274C	5.0571
YDL060W	YER102W	5.0561
YDR313C	YLL039C	5.056
YJL026W	YJL066C	5.055
YHR082C	YLR022C	5.055
YGR162W	YML017W	5.055
YAL062W	YFR055W	5.055
YAL062W	YBR093C	5.055
YAL062W	YAR071W	5.055
YDR304C	YHR185C	5.0547
YJL008C	YJL014W	5.0546
YDR225W	YFR037C	5.0544
YDR129C	YJL092W	5.0544
YDL112W	YGR054W	5.0538
YAR073W	YLR383W	5.0537
YOL010W	YPR144C	5.0532
YGL206C	YHR108W	5.0527
YGL206C	YGR149W	5.0527
YGL207W	YNL088W	5.0526
YCR079W	YOR151C	5.0526
YMR190C	YNL312W	5.0522
YLR234W	YNL312W	5.0522
YGR129W	YJR050W	5.0511
YGL122C	YHR089C	5.051
YBR017C	YHR089C	5.051
YIL078W	YMR205C	5.0505
YDR155C	YPL007C	5.05
YLR197W	YPR112C	5.0491
YBR191W	YDR450W	5.049
YGL190C	YOR162C	5.0489
YLR340W	YPL220W	5.0481
YDR172W	YJL016W	5.0481
YDR238C	YLR172C	5.0479
YBR114W	YDR499W	5.0474
YDR293C	YKL190W	5.047
YDL015C	YNL250W	5.0468
YDR335W	YFL008W	5.0465
YDL076C	YGL019W	5.046
YGR232W	YNL115C	5.0446
YDR235W	YPR178W	5.0442
YDR228C	YDR301W	5.0434
YHR060W	YOR270C	5.0433
YJL130C	YLR216C	5.0432
YER132C	YLR216C	5.0432
YIL075C	YPL002C	5.0424
YGL087C	YKL210W	5.0421
YDL240W	YPL009C	5.0413
YLR320W	YOR259C	5.041
YFR008W	YOR207C	5.041
YFR050C	YPR108W	5.0407
YER098W	YHR044C	5.0398
YBL104C	YGR041W	5.0398
YBL104C	YFL053W	5.0398
YBL104C	YDR316W	5.0398
YDR045C	YOR210W	5.0396
YDL115C	YGR140W	5.039
YCL044C	YDL115C	5.039
YBR160W	YPL031C	5.0387
YFR002W	YJL061W	5.0386
YBR143C	YKL152C	5.0382
YDR427W	YOL018C	5.0376
YGR090W	YPL153C	5.0375
YKL010C	YMR012W	5.037
YER127W	YLR264W	5.0367
YER155C	YGL120C	5.0362
YBR119W	YER172C	5.0361
YJR140C	YMR083W	5.0354
YJR140C	YML058W	5.0354
YHR186C	YNL192W	5.0354
YHR186C	YKL045W	5.0354
YHR082C	YHR186C	5.0354
YGR238C	YML006C	5.0354
YGR047C	YML006C	5.0354
YER167W	YLR154C	5.0354
YDL040C	YJR140C	5.0354
YBL016W	YLR354C	5.0354
YDL140C	YPR093C	5.0353
YER029C	YNL147W	5.0351
YKR081C	YLR074C	5.0348
YDR394W	YGL004C	5.0344
YDR129C	YJR065C	5.0341
YBL041W	YIL075C	5.0338
YKL179C	YOR116C	5.0334
YDL017W	YDR052C	5.0334
YHR121W	YIL112W	5.0323
YKR081C	YNL308C	5.0322
YEL055C	YNL075W	5.0322
YEL055C	YKL035W	5.0322
YEL037C	YHR103W	5.0321
YAL020C	YEL037C	5.0321
YDL097C	YER094C	5.0312
YLR200W	YPR133C	5.0303
YLR243W	YOR257W	5.0295
YGL067W	YOR257W	5.0295
YDL055C	YOR257W	5.0295
YGR165W	YPR185W	5.029
YGL180W	YGR165W	5.029
YLR143W	YOR341W	5.0289
YDR312W	YNL308C	5.0288
YFL018C	YGR078C	5.0284
YGR158C	YNR024W	5.0277
YML130C	YOL038W	5.0276
YLL011W	YLR109W	5.0276
YDR165W	YHL034C	5.0273
YDL201W	YHL034C	5.0273
YBL047C	YGR155W	5.0273
YMR145C	YOR176W	5.0271
YMR145C	YNL035C	5.0271
YGR159C	YHR197W	5.027
YIL035C	YLR354C	5.0268
YGL173C	YNL118C	5.0268
YCR033W	YNL189W	5.0259
YHL007C	YMR049C	5.0256
YNR054C	YOR061W	5.0252
YER069W	YLR315W	5.0251
YER136W	YGR270W	5.0239
YBR130C	YFL039C	5.0239
YDL112W	YDR496C	5.0238
YBR006W	YGR084C	5.0238
YGR209C	YLL060C	5.0237
YOR159C	YOR305W	5.0235
YER086W	YKL104C	5.0234
YFR053C	YLR262C-A	5.0225
YGL120C	YGR145W	5.022
YMR121C	YPL207W	5.0211
YDR050C	YNL127W	5.0211
YDR050C	YGL251C	5.0211
YDR050C	YGL131C	5.0211
YER047C	YPL126W	5.021
YGL234W	YOL094C	5.0202
YER062C	YJR014W	5.0202
YHR042W	YPR056W	5.02
YGR061C	YOR124C	5.02
YLR305C	YNL106C	5.0188
YBR103W	YDR155C	5.0185
YBR044C	YNL071W	5.0177
YAL048C	YKL007W	5.0177
YLR241W	YNL040W	5.0171
YDR148C	YOL058W	5.0171
YDL014W	YHR089C	5.0171
YBL058W	YOL012C	5.0171
YGL173C	YNL251C	5.0167
YBR245C	YPR080W	5.0161
YBR114W	YDL156W	5.0158
YGR061C	YOR158W	5.0157
YKL054C	YNL315C	5.0156
YJR041C	YMR125W	5.0155
YHR196W	YOR310C	5.0152
YDL014W	YDL040C	5.0151
YKR024C	YLL008W	5.0149
YGL200C	YOR261C	5.0145
YCR052W	YOR290C	5.014
YDR168W	YJR007W	5.0137
YFR004W	YGL004C	5.0136
YDR171W	YLR259C	5.0129
YDL006W	YDR170C	5.0126
YGR255C	YIL084C	5.011
YGR130C	YNR029C	5.0105
YDR005C	YDR045C	5.0105
YCL011C	YMR229C	5.0105
YBR097W	YNL250W	5.0105
YLR221C	YML030W	5.0104
YER165W	YIL079C	5.0104
YIL107C	YLR347C	5.01
YJL005W	YPL031C	5.0098
YBR221C	YPR140W	5.0098
YHR156C	YLR117C	5.0097
YGR090W	YGR145W	5.0087
YJL128C	YLR058C	5.0086
YDL006W	YLR058C	5.0086
YBR009C	YOR304W	5.0086
YKL007W	YLR225C	5.0084
YLR275W	YOR319W	5.0083
YCR063W	YGR074W	5.0082
YKR026C	YMR257C	5.0074
YIR026C	YKR026C	5.0074
YMR076C	YMR284W	5.0059
YCR092C	YDR499W	5.0059
YKL110C	YPL204W	5.0051
YBR088C	YKR081C	5.005
YHR114W	YJL201W	5.0046
YHR059W	YOR205C	5.0046
YGR150C	YOR205C	5.0046
YGR150C	YHR059W	5.0046
YDL167C	YIR001C	5.0046
YDL045W-A	YOR205C	5.0046
YDL045W-A	YGR150C	5.0046
YDR025W	YDR365C	5.0045
YBL104C	YNR022C	5.0043
YLR432W	YOR360C	5.0039
YGL066W	YNL236W	5.0026
YIR007W	YOL086C	5.0025
YER070W	YOL086C	5.0025
YDR101C	YHR085W	5.0025
YDL225W	YMR190C	5.002
YDL225W	YLR234W	5.002
YDL225W	YJR144W	5.002
YGL251C	YNL246W	5.0016
YBL016W	YNL246W	5.0016
YKL182W	YMR089C	5.0011
YFL007W	YFL045C	5.0011
YDL042C	YFL045C	5.0011
YAL005C	YJL197W	5.0011
YBL058W	YKR054C	5.001
YER094C	YHL030W	5.0003
YGL049C	YLR175W	5.0002
YGR016W	YOL139C	4.9998
YHR008C	YML022W	4.9992
YHL015W	YOL040C	4.9992
YER179W	YPL235W	4.9991
YLR196W	YPL207W	4.9987
YER155C	YOR267C	4.9987
YDR496C	YGL111W	4.9987
YHR059W	YMR188C	4.9986
YDR166C	YMR323W	4.9986
YOR063W	YOR204W	4.9984
YDR012W	YDR320C	4.9983
YLR197W	YPL266W	4.9982
YHR027C	YOR069W	4.9982
YHR027C	YJL204C	4.9982
YHR174W	YML121W	4.9978
YEL031W	YHR174W	4.9978
YER021W	YML057W	4.9973
YDL148C	YDR365C	4.9972
YLR449W	YMR290C	4.9959
YHL014C	YOR133W	4.9954
YBR135W	YLL050C	4.9954
YBR088C	YJR006W	4.9949
YOL054W	YOR178C	4.9948
YGL174W	YLR086W	4.9948
YDL086W	YHR169W	4.9948
YBR152W	YKL170W	4.9948
YAL025C	YDR198C	4.9948
YPR119W	YPR120C	4.9947
YLR226W	YPR119W	4.9947
YGL003C	YPR119W	4.9947
YCL014W	YNL182C	4.9947
YGR047C	YMR072W	4.9941
YDL126C	YGL108C	4.9927
YDL126C	YDR322C-A	4.9927
YMR004W	YNL244C	4.9924
YML114C	YMR227C	4.9922
YDR516C	YOR270C	4.9922
YCR042C	YMR227C	4.9922
YBR094W	YOR061W	4.9918
YIL062C	YNL073W	4.9912
YBL041W	YDR394W	4.9911
YDR335W	YDR457W	4.9903
YDL124W	YLR153C	4.9897
YDL014W	YOR039W	4.9892
YDL097C	YLR319C	4.9882
YCR012W	YDR385W	4.9875
YDR155C	YFL014W	4.9874
YDR155C	YDR510W	4.9874
YDR113C	YNL189W	4.9874
YBR267W	YER006W	4.9874
YER127W	YOR312C	4.9873
YHL014C	YPR074C	4.9862
YJL141C	YMR304W	4.9861
YKR028W	YOR267C	4.9858
YAL003W	YDR314C	4.9858
YJR050W	YPL213W	4.9857
YFR001W	YGL147C	4.9842
YDR432W	YML056C	4.9842
YHL033C	YNL317W	4.9834
YHR103W	YIL103W	4.9831
YER074W	YPL090C	4.9831
YAL020C	YIL103W	4.9831
YBR289W	YGL013C	4.983
YDL007W	YDR108W	4.9817
YML095C	YPL262W	4.9815
YER048C	YER090W	4.9813
YCR066W	YMR205C	4.9813
YGR103W	YLL034C	4.9809
YGL120C	YPL213W	4.9804
YCR088W	YGR086C	4.98
YBR079C	YER155C	4.9799
YNL186W	YOR205C	4.9794
YHR059W	YNL186W	4.9794
YGR150C	YNL186W	4.9794
YDL045W-A	YNL186W	4.9794
YBR159W	YOR038C	4.9794
YOL078W	YPL228W	4.9793
YNL064C	YPL233W	4.9793
YGL130W	YOL078W	4.9793
YER139C	YJR072C	4.9793
YCR030C	YDR348C	4.9793
YGR083C	YMR309C	4.9789
YMR116C	YOR196C	4.9788
YKL015W	YLR019W	4.9788
YIL071C	YOL088C	4.9788
YDR045C	YDR285W	4.9783
YGR180C	YKR090W	4.9781
YDR168W	YLR398C	4.9779
YER069W	YJR135C	4.9776
YMR033W	YOR304W	4.9774
YLR387C	YLR449W	4.9774
YLL011W	YNL075W	4.9772
YEL055C	YPL207W	4.9767
YDL140C	YDR251W	4.9756
YDR443C	YGL127C	4.975
YCR081W	YGL127C	4.975
YOR098C	YPL020C	4.9743
YCR092C	YDL156W	4.9743
YDR181C	YKL212W	4.974
YIL070C	YPL118W	4.9738
YDR025W	YPL198W	4.9736
YDL014W	YGR128C	4.9728
YGR233C	YLR186W	4.9725
YLR106C	YNR053C	4.9723
YBR079C	YKR026C	4.9721
YLR293C	YNL035C	4.9718
YLR055C	YML024W	4.9715
YBR136W	YLR347C	4.9713
YDR388W	YLR447C	4.9712
YER092W	YOR304W	4.9709
YFR008W	YJL011C	4.9707
YGL171W	YLR409C	4.9705
YKR059W	YML010W	4.9703
YAL038W	YOR133W	4.9692
YDR378C	YNR011C	4.9691
YDR322W	YLR360W	4.9691
YDL134C	YHR132W-A	4.9685
YLR223C	YMR224C	4.968
YBR017C	YPL169C	4.968
YHR030C	YNL053W	4.9678
YDR025W	YJL033W	4.9677
YNL107W	YPR031W	4.9669
YKR092C	YPL085W	4.9667
YGR104C	YLR290C	4.9667
YDR416W	YER029C	4.9666
YHR197W	YLR449W	4.9662
YMR255W	YNL047C	4.9661
YIL053W	YIL090W	4.966
YCR016W	YOL041C	4.9659
YBR081C	YNL236W	4.9654
YAL038W	YGL223C	4.9654
YDR101C	YOR252W	4.9651
YHL033C	YJR093C	4.965
YDR452W	YGR090W	4.9644
YDL043C	YPR082C	4.9642
YKL193C	YOR054C	4.9637
YFL001W	YKL060C	4.9637
YDR299W	YDR365C	4.9637
YHR170W	YMR323W	4.9629
YDL014W	YKL078W	4.9629
YDL082W	YDR418W	4.9621
YER177W	YPL160W	4.9619
YER021W	YPL088W	4.9618
YCR053W	YDL124W	4.9614
YGL043W	YJL176C	4.9613
YER177W	YNL267W	4.9602
YDR523C	YPL254W	4.9593
YDR124W	YDR175C	4.9593
YDR490C	YFR019W	4.959
YBR048W	YPR112C	4.959
YCL008C	YDL040C	4.9589
YOR116C	YPL254W	4.9586
Q0140	YOR158W	4.9582
YCL037C	YIL104C	4.9577
YKR062W	YOR312C	4.9567
YBR044C	YPL259C	4.9566
YAR042W	YDR150W	4.9566
YBR081C	YML007W	4.9561
YAL035W	YML056C	4.9561
YLR262C	YMR235C	4.9557
YOR116C	YPR010C	4.9556
YGR250C	YNL016W	4.9556
YLR003C	YPL266W	4.9554
YBR114W	YMR315W	4.9554
YBR114W	YIR002C	4.9554
YBR114W	YDR097C	4.9554
YBR169C	YNL127W	4.9553
YBR169C	YGL251C	4.9553
YBR169C	YGL131C	4.9553
YBL056W	YER052C	4.9552
YBL026W	YCR063W	4.9552
YDL031W	YNL132W	4.9544
YGR085C	YOL127W	4.9543
YNL061W	YOR310C	4.9541
YJL096W	YMR286W	4.9541
YGR161C	YKR027W	4.9541
YGR155W	YIL109C	4.9541
YDR427W	YPR103W	4.954
YER172C	YOL094C	4.9538
YEL055C	YOL077C	4.9533
YGL048C	YLR319C	4.9532
YLR430W	YPL022W	4.9527
YHR041C	YNL189W	4.9523
YDL147W	YER012W	4.9516
YDR328C	YEL051W	4.95
YDR166C	YLR027C	4.95
YNL227C	YOL012C	4.9498
YDL014W	YNL230C	4.9493
YAL024C	YOR014W	4.9491
YDR295C	YOR124C	4.9488
YLR277C	YOR250C	4.9484
YGL044C	YLR277C	4.9484
YIL128W	YNL249C	4.9479
YJL076W	YPR187W	4.9475
YAL038W	YOR347C	4.9471
YGL133W	YPR052C	4.9467
YDR172W	YPL083C	4.9467
YDR172W	YLR386W	4.9467
YDR381W	YOL041C	4.9451
YML123C	YOR204W	4.9436
YKL119C	YOR270C	4.9436
YHL015W	YOR198C	4.9435
YGL245W	YPR048W	4.9435
YGL245W	YKR071C	4.9435
YGL207W	YGR217W	4.9434
YHR081W	YNL189W	4.9433
YEL012W	YIL017C	4.9429
YBR065C	YNL250W	4.9429
YLR357W	YPR106W	4.9425
YMR086W	YNL208W	4.9424
YDR394W	YML057W	4.942
YCR031C	YJL177W	4.9419
YBL024W	YNL284C	4.9417
Q0140	YNL137C	4.9416
YLR342W	YML012W	4.9414
YBL076C	YDR457W	4.9412
YCR012W	YFR048W	4.9409
YJL130C	YPL240C	4.9408
YIL053W	YKL152C	4.9398
YDL060W	YLR435W	4.9398
YPL032C	YPR030W	4.9388
YIL159W	YPR030W	4.9388
YFR017C	YPR030W	4.9388
YER177W	YPR030W	4.9388
YER114C	YPR030W	4.9388
YDR017C	YPR030W	4.9388
YDR006C	YPR030W	4.9388
YDR001C	YPR030W	4.9388
YDL117W	YPR030W	4.9388
YBL034C	YPR030W	4.9388
YKL193C	YML074C	4.9387
YBL104C	YGL128C	4.9386
YDR245W	YEL022W	4.9385
YDL014W	YHR148W	4.9384
YHR132W-A	YMR273C	4.9382
YKL081W	YLR192C	4.938
YGL008C	YLR342W	4.9377
YGR116W	YKL206C	4.9376
YBR039W	YOR027W	4.9376
YDL166C	YER127W	4.9368
YAL005C	YPR033C	4.9368
YAL005C	YOR358W	4.9368
YIL142W	YPR119W	4.9353
YAR014C	YLR388W	4.9344
YAR014C	YJL136C	4.9344
YBR049C	YLR176C	4.9343
YBL076C	YBR121C	4.9341
YDL124W	YPL028W	4.9336
YDL124W	YDR353W	4.9336
YFL008W	YKR064W	4.9335
YCL016C	YOR217W	4.9335
YJR154W	YMR233W	4.9324
YJR017C	YOR224C	4.9322
YER179W	YIL021W	4.9318
YFR052W	YMR259C	4.9314
YDR225W	YDR303C	4.9314
YDL175C	YOR117W	4.9309
YDL175C	YGL048C	4.9309
YAL014C	YKL196C	4.9306
YJL001W	YOR299W	4.9304
YMR225C	YNL005C	4.9303
YML100W	YMR276W	4.9303
YML009C	YNL005C	4.9303
YER057C	YML100W	4.9303
YDR049W	YML100W	4.9303
YBR170C	YML100W	4.9303
YDR293C	YOR007C	4.9302
YAR007C	YPL082C	4.9302
YDR071C	YGR205W	4.9301
YBR272C	YGL004C	4.9301
YBR205W	YPL050C	4.9301
YBR205W	YML115C	4.9301
YGR218W	YIL063C	4.9299
YGL115W	YMR086W	4.9299
YDR238C	YPL222W	4.9299
YGR218W	YHL030W	4.9297
YGR148C	YKL157W	4.9295
YFL052W	YKL157W	4.9295
YBR139W	YDR074W	4.9295
YIL036W	YIL150C	4.9291
YDL014W	YML056C	4.9291
YLR069C	YOR014W	4.9289
YAR014C	YGR270W	4.9289
YML024W	YOL148C	4.9287
YCL032W	YGL251C	4.9287
YLR441C	YNR051C	4.9278
YDR171W	YGR171C	4.9269
YMR304W	YNR047W	4.9266
YCL039W	YLR430W	4.9266
YLR438C-A	YPR178W	4.9264
YDR460W	YLR005W	4.9254
YDR365C	YOR310C	4.9253
YER021W	YJL001W	4.9246
YER050C	YHR059W	4.9237
YDR494W	YHR059W	4.9237
YDR069C	YGR193C	4.9234
YLR106C	YOR151C	4.9233
YGL016W	YHL030W	4.9233
YFL034W	YHR023W	4.9233
YKR048C	YNL178W	4.9226
YOR304W	YPL128C	4.9224
YIL063C	YNL084C	4.9219
YOL002C	YPL022W	4.9212
YDR372C	YPL022W	4.9212
YER022W	YGR252W	4.9207
YDR012W	YLL045C	4.9206
YDR055W	YKL145W	4.9201
YOR181W	YPR171W	4.9198
YNL106C	YOR181W	4.9198
YLR384C	YML091C	4.9198
YHR016C	YOR181W	4.9198
YLR259C	YNL071W	4.9197
YER155C	YHL015W	4.9191
YGR145W	YPR137W	4.9188
YLR432W	YOL041C	4.9183
YBR247C	YKR060W	4.9183
YJR090C	YPR164W	4.9173
YJR063W	YMR173W	4.9173
YDR254W	YPR046W	4.9173
YDR016C	YGR113W	4.9173
YDR001C	YGR097W	4.9173
YCL059C	YGR159C	4.9173
YDR041W	YGR185C	4.9165
YAL021C	YJR011C	4.9162
YDR190C	YNL215W	4.9151
YER131W	YLR435W	4.9143
YMR315W	YOL097C	4.9138
YCR092C	YIR002C	4.9138
YCR092C	YDR097C	4.9138
YFR050C	YLR035C	4.9137
YKR081C	YOR272W	4.9135
YKL142W	YOR051C	4.913
YBL002W	YKR048C	4.913
YLR115W	YOR179C	4.9129
YKL104C	YKL161C	4.9126
YDL134C	YPL152W	4.9116
YBR031W	YIL018W	4.9116
YBR119W	YJR084W	4.9109
YJL124C	YOR234C	4.9105
YBL036C	YLL029W	4.9104
YBL008W	YML063W	4.9104
YDR050C	YMR052W	4.9102
YDL030W	YHR156C	4.9098
YER017C	YML075C	4.9091
YHR062C	YLR335W	4.909
YFL039C	YPL129W	4.9087
YBR136W	YMR190C	4.9085
YBR136W	YLR234W	4.9085
YBR136W	YJR144W	4.9085
YFR048W	YGL086W	4.9082
YKL056C	YKL081W	4.9078
YKL017C	YLR399C	4.9078
YGL233W	YPR055W	4.9073
YHR158C	YOR319W	4.9065
YBR122C	YCL014W	4.9065
YGR214W	YHL015W	4.9057
YHR158C	YOR023C	4.9056
YHR158C	YJL187C	4.9056
YFL039C	YKL130C	4.9056
YCR012W	YHR164C	4.9052
YIL124W	YKR046C	4.905
YOL108C	YOR373W	4.9049
YJR045C	YPL171C	4.9049
YHR179W	YJR045C	4.9049
YGL061C	YOR373W	4.9049
YGL061C	YOL108C	4.9049
YGL056C	YJR045C	4.9049
YDR294C	YKL008C	4.9049
YBR003W	YJR045C	4.9049
YNL138W	YOL001W	4.9043
YCR059C	YPR080W	4.9043
YIL090W	YMR004W	4.9037
YGL194C	YNL189W	4.9033
YDR002W	YMR235C	4.9018
YDL051W	YER126C	4.9018
YBR143C	YGL106W	4.9018
YHR167W	YPL179W	4.9014
YBR098W	YKL105C	4.9014
YGR186W	YJL176C	4.9012
YGL173C	YGL213C	4.9012
YER012W	YHR027C	4.9009
YDL098C	YML046W	4.9009
YJL069C	YOR145C	4.8998
YGL111W	YLR449W	4.8997
YDR016C	YMR083W	4.8997
YGL055W	YNL064C	4.8996
YCL054W	YLL034C	4.8996
YER110C	YOL034W	4.8991
YBR251W	YDR124W	4.8988
YFR051C	YML067C	4.8984
YBL007C	YPR171W	4.8984
YBL007C	YNL106C	4.8984
YBL007C	YHR016C	4.8984
YAL042W	YFR051C	4.8984
YDR279W	YNL106C	4.8976
YDR155C	YKR029C	4.8976
YJL066C	YNR001C	4.8972
YDR044W	YMR227C	4.8972
YGR090W	YPL128C	4.897
YER111C	YGR090W	4.897
YER089C	YGR090W	4.897
YBL047C	YHR174W	4.897
YCR077C	YLR147C	4.8966
YDR194C	YKR024C	4.8963
YBL099W	YDL192W	4.8961
YGR061C	YGR165W	4.896
YBR079C	YOR204W	4.8957
YBL093C	YLR071C	4.8956
YGR091W	YLR117C	4.8955
YDL082W	YGL103W	4.8955
YIL126W	YOR290C	4.8945
YER086W	YFR030W	4.8945
YGL098W	YLR342W	4.8941
YMR315W	YOR332W	4.8937
YMR086W	YOR220W	4.8934
YLR256W	YMR086W	4.8934
YGL181W	YMR086W	4.8934
YER126C	YLR325C	4.8934
YBR059C	YMR086W	4.8934
YGR245C	YMR049C	4.8923
YAL024C	YDL134C	4.8914
YJR031C	YOR019W	4.8913
YDR145W	YER148W	4.8912
YJL069C	YMR128W	4.8909
YER100W	YKL205W	4.8897
YCL042W	YLR058C	4.8897
YBL091C	YLR058C	4.8897
YFL008W	YLR347C	4.8896
YBR272C	YGR232W	4.8894
YMR001C	YPL129W	4.889
YBL036C	YNL306W	4.889
YLR175W	YNL186W	4.8875
YCL029C	YOR332W	4.8867
YKL142W	YLL026W	4.8862
YNL096C	YNL207W	4.8861
YDL083C	YMR230W	4.8859
YHR156C	YOR308C	4.8857
YNL248C	YOL142W	4.8853
YEL015W	YMR268C	4.8846
YLR115W	YLR448W	4.8841
YJL052W	YKL085W	4.8835
YER007W	YHR027C	4.8824
YDR383C	YPR046W	4.8824
YNL005C	YNL284C	4.882
YGR238C	YNR052C	4.8818
YGR047C	YNR052C	4.8818
YAL032C	YPL213W	4.8813
YDL185W	YKR101W	4.881
YJL074C	YJR109C	4.8802
YIL018W	YNL178W	4.88
YBR196C	YDR381W	4.8798
YOL086C	YOR075W	4.8796
YBR143C	YER103W	4.8795
YNL232W	YNR003C	4.8793
YGR002C	YPL169C	4.8791
YGL173C	YLR398C	4.8791
YBR211C	YER069W	4.8785
YBL041W	YDL097C	4.8783
YIL112W	YOR028C	4.8779
YDR175C	YHR197W	4.8779
YER083C	YML048W	4.8778
YDL100C	YML048W	4.8778
YDL097C	YDR055W	4.8778
YBR260C	YNL073W	4.8778
YBR260C	YHR060W	4.8778
YBR260C	YHR043C	4.8778
YLR448W	YOL127W	4.8776
YBL076C	YHR128W	4.8775
YNL250W	YNR051C	4.8774
YAR002W	YPR174C	4.8774
YDR101C	YLR002C	4.8772
YGR061C	YOR138C	4.8763
YJL122W	YMR308C	4.876
YHL030W	YOL038W	4.8756
YKL060C	YLR249W	4.8755
YGL049C	YJL190C	4.874
YKL173W	YMR125W	4.873
YJL080C	YOL013C	4.8729
YBR006W	YNL137C	4.8729
YGR175C	YNL183C	4.8728
YBR196C	YLR088W	4.872
YBR196C	YHR036W	4.872
YGL207W	YPL235W	4.8712
YHL034C	YNR051C	4.8711
YBL045C	YKL095W	4.8708
YEL036C	YPL094C	4.8704
YDR416W	YOR279C	4.8703
YDR416W	YDR515W	4.8703
YDR074W	YDR416W	4.8703
YDR424C	YKL210W	4.8702
YDR341C	YGL174W	4.87
YBR249C	YLR086W	4.87
YLR016C	YOR159C	4.8699
YMR135C	YPL018W	4.8697
YLR449W	YOR272W	4.8696
YAL005C	YDR068W	4.8696
YIL104C	YLR175W	4.8692
YBR155W	YLR355C	4.8692
YLR196W	YMR109W	4.8689
YDR087C	YOL077C	4.8683
YDR225W	YKR048C	4.8675
YBR278W	YMR224C	4.8675
YCL010C	YPL011C	4.8673
YER103W	YGR270W	4.8671
YHR069C	YLR347C	4.8669
YLR196W	YNL132W	4.8665
YCR063W	YPL064C	4.8664
YMR005W	YOR244W	4.8661
YKL150W	YOR032C	4.8661
YKL016C	YOR032C	4.8661
YKL016C	YKL150W	4.8661
YJR139C	YLR243W	4.8661
YJR017C	YML010W	4.8661
YIL156W	YLR243W	4.8661
YIL156W	YJR139C	4.8661
YHR128W	YOR032C	4.8661
YHR128W	YKL150W	4.8661
YHR128W	YKL016C	4.8661
YGR186W	YJR017C	4.8661
YGL017W	YOR244W	4.8661
YGL017W	YMR005W	4.8661
YFL024C	YMR005W	4.8661
YFL024C	YGL017W	4.8661
YDR457W	YML010W	4.8661
YDR457W	YJR017C	4.8661
YDR457W	YGR186W	4.8661
YDR394W	YNL290W	4.8661
YDR372C	YLL041C	4.8661
YDR298C	YOR032C	4.8661
YDR298C	YKL150W	4.8661
YDR298C	YHR128W	4.8661
Q0050	YER142C	4.8661
YGR218W	YKL145W	4.866
YDR170C	YGL195W	4.8659
YAL032C	YKL095W	4.8659
YKL145W	YPL088W	4.8658
YNL068C	YOL004W	4.8656
YGL150C	YOL004W	4.8656
YIL104C	YPL061W	4.8648
YOR155C	YOR232W	4.8646
YDR266C	YOR232W	4.8646
YDR314C	YDR394W	4.8645
YGR167W	YPL106C	4.8634
YCR063W	YER172C	4.8631
YJL039C	YOR257W	4.863
YBL030C	YGR282C	4.863
YGL137W	YKL081W	4.8624
YBR205W	YBR247C	4.8623
YDL097C	YFR050C	4.8621
YDL031W	YKR081C	4.8617
YGR128C	YPL012W	4.861
YBR146W	YOR204W	4.861
YGL097W	YHR193C	4.8608
YMR001C	YNL209W	4.8606
YJR132W	YOR160W	4.8604
YDL126C	YDL156W	4.8603
YHR023W	YML015C	4.8599
YDL043C	YJR084W	4.8599
YCL039W	YDL176W	4.8594
YDR166C	YLR023C	4.8593
YDR021W	YDR166C	4.8593
YKR027W	YOR299W	4.8592
YJR064W	YNR035C	4.8592
YJR064W	YLR370C	4.8592
YJR064W	YKL013C	4.8592
YIL062C	YJR064W	4.8592
YER127W	YLR197W	4.8592
YAL034C	YPR174C	4.8592
YDR050C	YPL019C	4.8591
YDR050C	YKL065C	4.8591
YDR050C	YFR048W	4.8591
YDR028C	YMR251W	4.8591
YDR028C	YLR411W	4.8591
YDR028C	YIR032C	4.8591
YDR028C	YIL177C	4.8591
YDR028C	YDR049W	4.8591
YBL022C	YHR084W	4.8588
YER120W	YKL009W	4.8585
YCR063W	YLR424W	4.8583
YHR044C	YOL111C	4.8581
YDR267C	YHR044C	4.8581
YER021W	YLR320W	4.858
YLR119W	YOR253W	4.8577
YBR031W	YPL090C	4.8577
YNL004W	YOR204W	4.857
YLR009W	YOR227W	4.8569
YJR063W	YPR032W	4.8569
YJL098W	YNL187W	4.8569
YJL098W	YNL101W	4.8569
YDR175C	YPR185W	4.8568
YDR175C	YGL180W	4.8568
YKL157W	YMR205C	4.8567
YHR166C	YNL005C	4.8567
YIL035C	YPL198W	4.8564
YDL098C	YLR382C	4.8562
YBL104C	YDL098C	4.8562
YHL033C	YJL190C	4.8559
YJR065C	YLR429W	4.8558
YMR284W	YNL031C	4.8557
YDR392W	YGR274C	4.8556
YDR394W	YPR103W	4.8553
YDR283C	YML057W	4.855
YDL168W	YLR002C	4.8547
YDR172W	YOR069W	4.8546
YLR185W	YNL163C	4.8545
YLL034C	YOL133W	4.8543
YHR199C	YNL323W	4.8543
YHR199C	YLR326W	4.8543
YJR077C	YML085C	4.854
YER048C	YOR064C	4.854
YBR105C	YDL082W	4.8539
YKL173W	YLR438C-A	4.8538
YHR024C	YOR133W	4.8535
YBR011C	YJR042W	4.8535
YKL129C	YNL232W	4.8534
YEL051W	YOR270C	4.8529
YGR161C	YHR132W-A	4.8523
YJL069C	YOR078W	4.852
YKR081C	YLR002C	4.8517
YBL045C	YDL179W	4.8511
YMR213W	YPL064C	4.8507
YGR240C	YOR124C	4.8507
YEL002C	YNL055C	4.8507
YDR457W	YDR463W	4.8507
YBR072W	YML111W	4.8507
YBR072W	YDR213W	4.8507
YDR174W	YPR104C	4.8501
YER155C	YPR088C	4.8497
YER155C	YPL210C	4.8497
YER155C	YML105C	4.8497
YDL092W	YER155C	4.8497
YLR187W	YNL007C	4.8495
YHR023W	YPL011C	4.8492
YGL190C	YMR167W	4.8489
YDR416W	YLR382C	4.8489
YBL104C	YDR416W	4.8489
YBL019W	YCR028C-A	4.8481
YPL126W	YPL266W	4.848
YKR059W	YOR039W	4.8479
YER036C	YGL123W	4.8479
YLR147C	YPL178W	4.8478
YGL171W	YLR186W	4.8476
YJL050W	YJL179W	4.8475
YIL007C	YLR304C	4.8472
YJL190C	YJR123W	4.8471
YMR109W	YOR035C	4.8468
YKL129C	YMR109W	4.8468
YDR356W	YMR109W	4.8468
YBR175W	YDR069C	4.8468
YDR324C	YPR112C	4.8457
YGR091W	YPR101W	4.8455
YCR052W	YPR106W	4.8449
YBR169C	YMR052W	4.8445
YNL047C	YOR259C	4.8444
YLR192C	YPL237W	4.8439
YMR093W	YNL178W	4.8435
YKL173W	YLR382C	4.8434
YBL072C	YDL040C	4.8434
YOR046C	YPL169C	4.8433
YJR109C	YOR086C	4.8433
YJL026W	YLR259C	4.8431
YHR069C	YNR003C	4.8428
YJR113C	YNR036C	4.842
YDR394W	YJL008C	4.8418
YLR275W	YOR305W	4.8417
YJL034W	YPL088W	4.8417
YJL034W	YMR316W	4.8417
YGL028C	YJL034W	4.8417
YMR072W	YOL067C	4.8409
YBL103C	YMR072W	4.8409
YDR156W	YPL255W	4.8405
YMR205C	YOR335C	4.8404
YJL201W	YOR181W	4.8401
YDR283C	YLR384C	4.8401
YHR069C	YKL129C	4.84
YBR055C	YIL144W	4.8399
YMR229C	YNL175C	4.8397
YFR031C-A	YHL001W	4.8397
YGR135W	YOR261C	4.8396
YNL262W	YOL094C	4.8395
YDL185W	YLL040C	4.839
YDL087C	YPR057W	4.8387
YCL059C	YMR128W	4.8381
YHR197W	YPL013C	4.8379
YHR027C	YKL104C	4.8379
YDR499W	YNL312W	4.8379
YBR233W-A	YMR083W	4.8374
YBR196C	YDR050C	4.8374
YBR109C	YBR177C	4.8366
YGR075C	YJL203W	4.8365
YGR104C	YNL189W	4.8364
YDR448W	YMR112C	4.8364
YCL054W	YDR365C	4.8362
YLR241W	YOR173W	4.8354
YBL058W	YJR052W	4.8354
YIL076W	YPL222W	4.8349
YFL034W	YPL110C	4.8349
YLL021W	YOR373W	4.8346
YLL021W	YOL108C	4.8346
YGL061C	YLL021W	4.8346
YFR013W	YPL128C	4.8346
YBR031W	YNL182C	4.8344
YDL060W	YDR023W	4.8343
YHR141C	YIL133C	4.8341
YER157W	YIL063C	4.8327
YDR519W	YGR214W	4.8324
YDR214W	YER052C	4.8323
YKR048C	YOR207C	4.8317
YDL047W	YNR016C	4.8317
YML095C	YPL183C	4.8316
YBR154C	YLR200W	4.8316
YDR245W	YPR006C	4.8315
YDR245W	YPL183C	4.8315
YPL012W	YPL126W	4.8305
YBR025C	YOR259C	4.8305
YDL145C	YPL031C	4.8302
YGR245C	YKR081C	4.8297
YGR192C	YNR033W	4.8292
YNR038W	YOL144W	4.8285
YOL010W	YPL259C	4.8279
YLR328W	YPL259C	4.8279
YGR130C	YML049C	4.8279
YDR300C	YPL262W	4.8279
YHR074W	YOR220W	4.8278
YHR090C	YLR385C	4.8277
YDL150W	YNL039W	4.8272
YDR299W	YER082C	4.8259
YBL002W	YDR225W	4.8257
YJL074C	YKR064W	4.8253
YLR385C	YOL052C	4.8252
YEL037C	YGR258C	4.8252
Q0140	YPL118W	4.8252
YGR013W	YOR159C	4.8249
YHR052W	YOL127W	4.8244
YCR057C	YGL019W	4.8244
YJL148W	YOR224C	4.824
YBR080C	YLR421C	4.8238
YLR432W	YNL253W	4.8237
YAL032C	YNR011C	4.8236
YBR158W	YGR285C	4.8235
YMR312W	YPL204W	4.8234
YMR108W	YNL128W	4.8233
YDR452W	YNL112W	4.8232
YDL136W	YHR193C	4.8232
YEL044W	YER092W	4.8231
YEL012W	YIL097W	4.8231
YAL032C	YGR129W	4.8227
YML049C	YPL178W	4.8221
YLR074C	YOL127W	4.8219
YDR174W	YPL022W	4.8219
YDR174W	YML095C	4.8219
YGL135W	YKL180W	4.821
YGL049C	YGL173C	4.8201
YDR369C	YLR223C	4.8201
YDL126C	YJR077C	4.82
YMR093W	YOR310C	4.8198
YDR449C	YOR310C	4.8198
YHL001W	YIL133C	4.8193
YBR142W	YKL014C	4.8192
YBR114W	YPL154C	4.8187
YBL007C	YJL201W	4.8187
YHR196W	YPL266W	4.8183
YDR235W	YHR165C	4.8179
YDL043C	YOR308C	4.8175
YIL070C	YNL030W	4.8173
YNR011C	YPR182W	4.8169
YNR007C	YOR160W	4.8169
YHR156C	YPR182W	4.8169
YBR218C	YOL088C	4.8169
YBL046W	YJL138C	4.8162
YIL133C	YKR062W	4.8157
YDL014W	YMR014W	4.8157
YOR136W	YOR261C	4.8153
YDL126C	YOR153W	4.8152
YDR324C	YGR145W	4.8151
YDR099W	YKR098C	4.8151
YER048C	YKL211C	4.8149
YGR204W	YIL137C	4.8148
YJL052W	YLR355C	4.8142
YCR012W	YNL271C	4.8141
YJL034W	YPL091W	4.814
YKL213C	YOR341W	4.8139
YPL127C	YPL129W	4.8131
YLR233C	YOR201C	4.8131
YLR154C	YNL088W	4.8131
YGL179C	YKR096W	4.813
YER161C	YNL016W	4.813
YEL037C	YML100W	4.813
YDL190C	YML100W	4.813
YBL016W	YDR477W	4.813
YKR048C	YOR116C	4.8126
YGR103W	YOR312C	4.8126
YJR122W	YPL128C	4.8119
YLR347C	YOL021C	4.8117
YNL084C	YNL113W	4.8116
YDR041W	YDR453C	4.8116
YML072C	YOR086C	4.8109
YOR091W	YPR045C	4.8107
YGR162W	YLR023C	4.8107
YNL248C	YPR032W	4.8106
YDR044W	YGR094W	4.8105
YBR265W	YDR028C	4.8105
YBL023C	YDR028C	4.8105
YBL004W	YKR060W	4.8104
YKL060C	YMR301C	4.8102
YER174C	YKL060C	4.8102
YDL090C	YKL152C	4.8102
YBL006C	YJL137C	4.8092
YJL065C	YML062C	4.8086
YDR408C	YOR361C	4.8081
YJL002C	YML130C	4.8078
YGL022W	YML130C	4.8078
YGL241W	YPR190C	4.8077
YGL167C	YJL183W	4.8073
YJL008C	YJL074C	4.8072
YMR145C	YOL123W	4.8067
YER142C	YOL090W	4.8057
Q0050	YOL090W	4.8057
YFL033C	YPL203W	4.8056
YFL033C	YJL164C	4.8056
YBL104C	YMR240C	4.8056
YDR381W	YML034W	4.8054
YDL089W	YDR381W	4.8054
YGL120C	YLR002C	4.8052
YDR295C	YOR138C	4.8051
YGL245W	YGR254W	4.8049
YNL106C	YNL208W	4.8046
YJL197W	YJR031C	4.8046
YDL132W	YJL149W	4.8041
YBL018C	YLR335W	4.8041
YAL062W	YPL228W	4.8041
YAL062W	YGL130W	4.8041
YAL033W	YLR335W	4.8041
YDR055W	YHR027C	4.8034
YDL075W	YNL096C	4.8033
YFR001W	YKL014C	4.803
YDR312W	YPL012W	4.8026
YGR060W	YML029W	4.8025
YMR194W	YPR025C	4.8023
YGR135W	YIL009C-A	4.8022
YGL241W	YOR038C	4.8021
YDR142C	YIL068C	4.8021
YCR020W-B	YDR335W	4.8015
YBL008W	YLR392C	4.8015
YDR337W	YJL063C	4.8014
YML007W	YNR023W	4.8012
YHL030W	YOR341W	4.8012
YJL167W	YPL149W	4.8011
YGL135W	YLR448W	4.8007
YMR078C	YPR175W	4.7987
YGL031C	YGL103W	4.7985
YEL044W	YGL150C	4.7983
YGL008C	YGL147C	4.7974
YBR253W	YHR202W	4.7974
YFR010W	YHL030W	4.7968
YDR194C	YGL035C	4.7968
YAL005C	YJL026W	4.7968
YDR235W	YLL036C	4.7966
YOR069W	YPL070W	4.7964
YBR136W	YJL016W	4.7956
YGL043W	YGR063C	4.7951
YMR075W	YNL107W	4.795
YFL039C	YNL218W	4.7948
YFL039C	YJR031C	4.7948
YAR007C	YOR056C	4.7948
YHL004W	YOR204W	4.7945
YKL189W	YMR058W	4.7944
YGL100W	YPL215W	4.7944
YLR175W	YNL132W	4.7943
YJR041C	YNR038W	4.7929
YBR044C	YFL018C	4.7929
YDR104C	YLR315W	4.7924
YNL132W	YOR310C	4.7922
YBR289W	YJL081C	4.792
YLR429W	YPL004C	4.7917
YDL147W	YJL008C	4.7914
YGR159C	YJL076W	4.7913
YGL103W	YML072C	4.7913
YDR243C	YDR388W	4.7912
YDR093W	YPL009C	4.7909
YGR103W	YLR106C	4.7908
YIL075C	YLR180W	4.7907
YLR274W	YPL106C	4.7905
YDL153C	YIL091C	4.7904
YDR343C	YDR516C	4.7896
YNL022C	YOR257W	4.7892
YNL005C	YPL183W-A	4.7892
YBR011C	YIL161W	4.7892
YHR015W	YOR141C	4.7888
YGL133W	YOL006C	4.7888
YKL104C	YOR136W	4.7883
YGR084C	YGR211W	4.7883
YER069W	YGR179C	4.7883
YBR245C	YKR048C	4.7883
YML075C	YMR246W	4.7882
YIL142W	YNL187W	4.7881
YIL142W	YNL101W	4.7881
YLL024C	YMR080C	4.7879
YAR007C	YPR065W	4.7878
YDL225W	YDR499W	4.7876
YDL176W	YLR430W	4.7873
YDL225W	YPL235W	4.7872
YER071C	YLR216C	4.7863
YJL173C	YPL075W	4.7861
YBL072C	YDL060W	4.7852
YEL018W	YLR455W	4.7849
YDL031W	YDR101C	4.7849
YDL143W	YJL014W	4.7845
YBR009C	YIL070C	4.7841
YDL160C	YGL121C	4.784
YDR419W	YPR160W	4.7839
YDR190C	YJR053W	4.7836
YER165W	YKL025C	4.7832
YBL041W	YHL030W	4.7832
YGR034W	YGR085C	4.7831
YDL097C	YPR103W	4.783
YDR049W	YGL246C	4.7829
YBL017C	YDR432W	4.7829
YAL032C	YER013W	4.7829
YMR191W	YOR259C	4.7823
YDR447C	YGL031C	4.7822
YDR364C	YLR424W	4.7815
YBL061C	YLR355C	4.7811
YML028W	YPR074C	4.7807
YER088C	YPL181W	4.7807
YDR430C	YER178W	4.7807
YGR175C	YNL116W	4.7803
YDR434W	YGR192C	4.7801
YJL138C	YOL086C	4.779
YDR235W	YDR515W	4.7788
YNL186W	YOR204W	4.7787
YFR024C-A	YPR171W	4.7787
YFR024C-A	YNL106C	4.7787
YFR024C-A	YHR016C	4.7787
YHR066W	YPL219W	4.7773
YBL030C	YMR145C	4.7773
YOL097C	YPL154C	4.7772
YFL034C-A	YIL094C	4.777
YHR147C	YJL063C	4.7769
YDR300C	YGL146C	4.7768
YBR114W	YOL012C	4.7768
YFL034C-B	YLR447C	4.7767
YDR075W	YHR011W	4.7767
YBL046W	YHR011W	4.7767
YDR306C	YNL138W	4.7766
YDL193W	YGL195W	4.7764
YDL098C	YKR022C	4.7763
YGR258C	YIL103W	4.7762
YDR353W	YOL128C	4.7762
YDR156W	YLR143W	4.7762
YBR189W	YKL143W	4.7762
YER023W	YIL070C	4.7761
YLR057W	YPL181W	4.7758
YDR424C	YNL138W	4.7756
YAR002C-A	YKL192C	4.7756
YAL043C	YLR448W	4.7756
YAL017W	YDR099W	4.7756
YOR259C	YPL235W	4.7754
YIL115C	YKL210W	4.7753
YGR078C	YIL115C	4.7753
YDR462W	YNL177C	4.7753
YDL124W	YOR335C	4.7749
YDL014W	YPR143W	4.7746
YDR427W	YKL184W	4.7739
YDR188W	YPL226W	4.7737
YML070W	YPR110C	4.7735
YEL048C	YPR110C	4.7735
YGR104C	YJR066W	4.7732
YLR342W	YOR075W	4.7726
YJL168C	YOR293W	4.7724
YDR263C	YLR421C	4.7724
YBR127C	YFL037W	4.7715
YBR055C	YER018C	4.7712
YDR101C	YMR308C	4.7711
YBR189W	YGL147C	4.771
YDL043C	YGR013W	4.7705
YAL015C	YER052C	4.7704
YGR195W	YLR398C	4.77
YDL060W	YGR145W	4.7698
YDL148C	YKL211C	4.7695
YBR189W	YOR145C	4.7691
YIL035C	YJR007W	4.7684
YJL042W	YOL012C	4.7682
YGL099W	YPL093W	4.7679
YBL004W	YHR196W	4.7667
YOR304W	YPR052C	4.7664
YOL090W	YOR323C	4.7663
YLR044C	YMR083W	4.7661
YBR198C	YFR037C	4.7652
YAL016W	YHR132W-A	4.7648
YDR385W	YIL053W	4.7639
YER103W	YPL240C	4.7632
YER088C	YMR263W	4.7631
YLL040C	YOR326W	4.7628
YKL145W	YLR320W	4.7624
YER127W	YOR204W	4.7623
YCL032W	YGL157W	4.7623
YGL100W	YKL186C	4.7619
YBR160W	YIL053W	4.7615
YKR081C	YNL132W	4.7613
YKL172W	YMR229C	4.7613
YML123C	YMR319C	4.7607
YKL101W	YPL048W	4.7606
YGL019W	YLR129W	4.7606
YDL029W	YEL060C	4.7604
YBR143C	YLR344W	4.7601
YBR143C	YHR015W	4.7601
Q0080	YDR238C	4.7595
YKR048C	YMR284W	4.7594
YDR198C	YOR005C	4.7592
YAL025C	YOR005C	4.7592
YHR127W	YKL012W	4.7588
YGR205W	YHL030W	4.7588
YGR158C	YNL307C	4.7584
YBR160W	YMR304W	4.7584
YDL192W	YMR094W	4.7583
YBL045C	YIL061C	4.7582
YBR025C	YLR249W	4.7575
YOR332W	YPL154C	4.7571
YLR277C	YOR179C	4.7568
YMR116C	YMR323W	4.7565
YHL004W	YNR036C	4.7564
YBR279W	YJL087C	4.7563
YDL043C	YMR125W	4.7562
YAL005C	YJL061W	4.7562
YGL045W	YHR013C	4.756
YDL156W	YDL225W	4.756
YDR141C	YGR270W	4.7557
YKL155C	YOR204W	4.7554
YHL033C	YJL177W	4.7525
YCR014C	YOR259C	4.7518
YLR293C	YOL123W	4.7517
YOR039W	YOR054C	4.7516
YML067C	YML130C	4.7509
YAL042W	YML130C	4.7509
YNL044W	YPR080W	4.7508
YDL236W	YPR080W	4.7508
YPL219W	YPR143W	4.7507
YDR198C	YNL097C	4.7507
YDR392W	YML024W	4.7506
YIL148W	YOL133W	4.7505
YIL148W	YOL063C	4.7505
YGR140W	YIL021W	4.7502
YCL044C	YIL021W	4.7502
YJL050W	YJR145C	4.7501
YDR028C	YGR156W	4.7493
YNL151C	YNL248C	4.7492
YER027C	YML006C	4.7488
YDR359C	YMR005W	4.7488
YDR359C	YGL017W	4.7488
YDR247W	YDR507C	4.7488
YOR185C	YPL111W	4.7486
YDL204W	YKL212W	4.7486
YDR091C	YER025W	4.7485
YGL122C	YMR145C	4.7483
YBR017C	YMR145C	4.7483
YOR230W	YOR283W	4.7482
YBR272C	YOL054W	4.7479
YGL120C	YMR290C	4.7476
YBR098W	YDR381W	4.7476
YLR103C	YNL330C	4.7472
YDR450W	YGR085C	4.7461
YPL013C	YPR166C	4.7459
YNL081C	YPL013C	4.7459
YKL003C	YPR166C	4.7459
YKL003C	YNL081C	4.7459
YER050C	YKL003C	4.7459
YGR034W	YHL001W	4.7455
YKL143W	YOR145C	4.7454
YPL247C	YPR184W	4.7453
YLR368W	YOL059W	4.7453
YLR309C	YNL271C	4.7453
YLR270W	YPR184W	4.7453
YKR001C	YPR184W	4.7453
YJR089W	YLR231C	4.7453
YHR051W	YPL009C	4.7453
YHL007C	YOL059W	4.7453
YHL007C	YLR368W	4.7453
YGR066C	YML078W	4.7453
YGL151W	YLR231C	4.7453
YGL151W	YJR089W	4.7453
YDR299W	YLR231C	4.7453
YDR299W	YJR089W	4.7453
YDR299W	YGL151W	4.7453
YDL101C	YMR224C	4.7453
YDL080C	YLR231C	4.7453
YDL080C	YJR089W	4.7453
YDL080C	YGL151W	4.7453
YDL080C	YDR299W	4.7453
YDL022W	YOL059W	4.7453
YDL022W	YLR368W	4.7453
YDL022W	YHL007C	4.7453
YDL017W	YLR231C	4.7453
YDL017W	YJR089W	4.7453
YDL017W	YGL151W	4.7453
YDL017W	YDR299W	4.7453
YDL017W	YDL080C	4.7453
YBR252W	YML078W	4.7453
YBR252W	YGR066C	4.7453
YBR202W	YNL271C	4.7453
YBR202W	YLR309C	4.7453
YDR104C	YJR135C	4.7449
YML102W	YPL153C	4.7435
YNR003C	YOR001W	4.7434
YDR234W	YLR249W	4.7426
YCR063W	YDR473C	4.7426
YLR389C	YMR037C	4.7419
YBR006W	YGL129C	4.7419
YLR455W	YNL201C	4.7418
YDR083W	YNL201C	4.7418
YDR069C	YHR119W	4.7418
YDR069C	YDR469W	4.7418
YOR176W	YPL215W	4.7415
YDR422C	YOR267C	4.7415
YHR069C	YPR110C	4.7413
YHR066W	YOL001W	4.7411
YER048C	YMR117C	4.7411
YER048C	YIR010W	4.7411
YGR204W	YOR184W	4.741
YAR007C	YKR001C	4.7402
YJL076W	YMR104C	4.7396
YAL013W	YIL035C	4.7392
YBR065C	YMR240C	4.7389
YGR104C	YGR252W	4.7387
YCR002C	YDR158W	4.7384
YOL005C	YPR180W	4.7378
YER052C	YGL137W	4.7373
YML120C	YOR160W	4.7372
YDR060W	YKL172W	4.737
YAL005C	YER023W	4.7364
YBL075C	YHR121W	4.7361
YAL024C	YMR273C	4.7358
YLR029C	YNL069C	4.7355
YJL069C	YLL011W	4.7347
YMR012W	YMR198W	4.7346
YDR299W	YGR128C	4.7344
YLR310C	YNL187W	4.7343
YLR310C	YNL101W	4.7343
YGL197W	YNL187W	4.7343
YGL197W	YNL101W	4.7343
YER133W	YFR015C	4.7337
YDR130C	YER133W	4.7337
YDL030W	YMR268C	4.7335
YDR283C	YNL047C	4.7334
YBL024W	YER102W	4.7334
YBR127C	YGR155W	4.7332
YBR025C	YKL145W	4.7325
YDL007W	YDR052C	4.7323
YIL104C	YMR286W	4.7318
YIL104C	YJL096W	4.7318
YNL071W	YNL107W	4.7316
YDR044W	YGR264C	4.7308
YCL014W	YDL148C	4.7301
YIL128W	YPR025C	4.73
YDL108W	YIL128W	4.73
YBR184W	YIL128W	4.73
YPR054W	YPR191W	4.7287
YMR229C	YOR039W	4.7286
YDR299W	YPL217C	4.7285
YER125W	YLR392C	4.7282
YER125W	YKR018C	4.7282
YER125W	YGR136W	4.7282
YDR436W	YGR130C	4.7282
YJR070C	YLR105C	4.7279
YMR125W	YNL139C	4.7276
YBL016W	YDR171W	4.727
YHR158C	YOR014W	4.7269
YER146W	YGR091W	4.7268
YKL099C	YLR222C	4.7267
YER021W	YOR108W	4.7262
YER117W	YKL172W	4.7261
YLR033W	YMR072W	4.726
YJL080C	YKL081W	4.7257
YMR304W	YOR204W	4.7252
YBR009C	YLR357W	4.7252
YPL088W	YPL219W	4.7249
YAL029C	YML057W	4.7249
YJL081C	YOR116C	4.7248
YDL209C	YGR091W	4.7248
YOL086C	YOR133W	4.7245
YFR002W	YHR170W	4.7239
YAL024C	YML109W	4.7239
YDR175C	YGR061C	4.7238
YDR158W	YJL074C	4.7238
YJR045C	YLR143W	4.7234
YIR006C	YJR045C	4.7234
YDL040C	YLR119W	4.7234
YCR054C	YJR045C	4.7234
YMR314W	YNR003C	4.7227
YLR196W	YNR038W	4.7223
YGR074W	YLR424W	4.7223
YIL018W	YOR063W	4.7222
YMR188C	YNL242W	4.7221
YAL059W	YLR074C	4.7221
YHR197W	YIL093C	4.7219
YDL007W	YER162C	4.7218
YHR027C	YHR119W	4.7216
YKR011C	YLR314C	4.7215
YDR341C	YLR314C	4.7215
YLR196W	YNL308C	4.7214
YBR009C	YJR052W	4.721
YGL253W	YNL139C	4.7202
YFR053C	YHR064C	4.7202
YNL243W	YOR181W	4.72
YDR212W	YJL106W	4.7198
YCR053W	YLR153C	4.7198
YLR057W	YMR263W	4.7197
YBR278W	YDR369C	4.7197
YGL064C	YGR076C	4.7195
YDR115W	YGR076C	4.7195
YDR115W	YGL064C	4.7195
YDR141C	YPL031C	4.7183
YAR073W	YGL120C	4.7179
YIR035C	YOR076C	4.7178
YKR002W	YOR250C	4.7176
YGL044C	YKR002W	4.7176
YLR289W	YNL037C	4.7175
YFR008W	YKR025W	4.7174
YBR251W	YPR166C	4.717
YBR251W	YNL081C	4.717
YLL050C	YLR429W	4.7168
YDL006W	YOL148C	4.7168
YLR359W	YOR361C	4.7167
YDR299W	YHR148W	4.7166
YKR048C	YLR058C	4.716
YGR090W	YPL266W	4.716
YDR314C	YGL048C	4.7158
YHR072W-A	YLR175W	4.7153
YGL128C	YPR082C	4.7148
YMR297W	YOL144W	4.7146
YOL001W	YPR143W	4.7145
YER154W	YGR132C	4.7143
YDR224C	YEL024W	4.7138
YDL200C	YDR224C	4.7138
YBL002W	YMR044W	4.7131
YKL182W	YMR205C	4.713
YGR087C	YLR355C	4.713
YGR087C	YLL050C	4.7124
YOR344C	YPR107C	4.712
YER091C	YLR304C	4.7118
YKL081W	YMR186W	4.7114
YHR174W	YPR159W	4.7112
YHR174W	YNL192W	4.7112
YGR266W	YHR174W	4.7112
YER168C	YHR174W	4.7112
YER165W	YML117W	4.7111
YMR093W	YPR112C	4.7107
YDR449C	YPR112C	4.7107
YDL055C	YJL034W	4.7105
YDL076C	YDR207C	4.7102
YDR127W	YOR151C	4.7095
YKL192C	YML012W	4.7092
YDR382W	YGR034W	4.7086
YDR235W	YLR382C	4.7085
YAL036C	YGR285C	4.7084
YMR199W	YPR119W	4.7081
YLL019C	YOR178C	4.7081
YLL019C	YOL054W	4.7081
YIL128W	YOR116C	4.7081
YHR010W	YOL127W	4.7081
YGL200C	YPR159W	4.7081
YFR015C	YGL081W	4.7081
YDR477W	YLR113W	4.7081
YDR198C	YGL146C	4.7081
YBR079C	YOL087C	4.7081
YAL025C	YGL146C	4.7081
YLR027C	YMR116C	4.708
YHR096C	YMR028W	4.7078
YLR433C	YML057W	4.7077
YER090W	YGL115W	4.7077
YFR040W	YOR267C	4.7073
YDR381W	YOL012C	4.7066
YER053C	YGR130C	4.7063
YER048C	YJR112W	4.7062
YEL060C	YGL137W	4.7058
YHL015W	YHR018C	4.7056
YJL011C	YNL084C	4.7053
YEL034W	YLR044C	4.7051
YBR058C	YIL126W	4.705
YPL129W	YPL153C	4.7049
YFR021W	YPL258C	4.7046
YOR204W	YPL118W	4.7034
YDL175C	YLR347C	4.7033
YDL047W	YNL187W	4.7033
YDL047W	YNL101W	4.7033
YCL059C	YNR054C	4.7027
YCL011C	YGR162W	4.7018
YIL115C	YJL050W	4.7015
YCR082W	YDR448W	4.7014
YGL245W	YGR285C	4.7012
YHR027C	YPL088W	4.7011
YER056C-A	YHR119W	4.7005
YDL031W	YJR145C	4.7005
YIR003W	YLR216C	4.6997
YIL093C	YNR036C	4.6995
YDL029W	YOR141C	4.6995
YDL075W	YPL249C-A	4.6991
YFR024C-A	YJL201W	4.699
YFR024C-A	YHR114W	4.699
YER177W	YPR049C	4.6989
YDR382W	YDR471W	4.6986
YBL007C	YNL243W	4.6986
YBR169C	YML112W	4.6985
YBR169C	YKL139W	4.6985
YBR169C	YJL006C	4.6985
YDL042C	YOR116C	4.6982
YOR173W	YPR025C	4.6976
YDL108W	YOR173W	4.6976
YOR069W	YOR261C	4.6972
YKR024C	YMR229C	4.6972
YHR010W	YPR189W	4.6968
YDL087C	YMR240C	4.6967
YDL192W	YLR109W	4.696
YCR012W	YIL053W	4.696
YGL103W	YOL040C	4.6959
YDL225W	YIR002C	4.6957
YDL225W	YDR097C	4.6957
YHR147C	YML025C	4.6954
YGR282C	YOR317W	4.6954
YIL062C	YPR093C	4.6951
YBR103W	YOR279C	4.6951
YBL037W	YPL016W	4.6951
YDR395W	YOR207C	4.6943
YFR019W	YKR028W	4.6942
YDR314C	YKL101W	4.6942
YBR136W	YPL083C	4.6942
YBR136W	YLR386W	4.6942
YBR136W	YDR499W	4.6942
YLR087C	YOR270C	4.6933
YJL012C	YOR270C	4.6933
YGL122C	YLR293C	4.6933
YBR017C	YLR293C	4.6933
YCL059C	YLL011W	4.6931
YPL018W	YPL243W	4.6928
YDL192W	YER092W	4.6928
YIL075C	YNL049C	4.6923
YDR353W	YLR153C	4.6921
YCL014W	YLR314C	4.6914
YBR031W	YGR090W	4.6912
YDR012W	YOL021C	4.6908
YBL079W	YFL039C	4.6908
YBL084C	YFR036W	4.6904
YBL092W	YDL136W	4.6903
YNL061W	YOL120C	4.6901
YML127W	YMR125W	4.69
YER104W	YLR347C	4.6897
YHL034C	YOL077C	4.6892
YKR001C	YOL072W	4.6888
YDR397C	YKR001C	4.6888
YDR159W	YKR001C	4.6888
YDR081C	YKR001C	4.6888
YOL001W	YPL088W	4.6887
YDL164C	YDR328C	4.6887
YBR264C	YNL115C	4.6887
YHR052W	YHR197W	4.6886
YGR196C	YOR061W	4.6886
YDR058C	YOL012C	4.6885
YBR267W	YOL012C	4.6885
YAL027W	YOL059W	4.6884
YAL027W	YLR368W	4.6884
YAL027W	YHL007C	4.6884
YAL027W	YDL022W	4.6884
YLR275W	YNL024C	4.6882
YNL030W	YNL088W	4.6881
YDR432W	YIL061C	4.688
YJL203W	YLR424W	4.6878
YIL018W	YLR029C	4.6875
YBL076C	YGL245W	4.6864
YBL076C	YKL029C	4.6861
YDR385W	YHR174W	4.6858
YDL076C	YIL035C	4.6858
YCL059C	YDL112W	4.6858
YDR234W	YNL079C	4.6846
YGL103W	YMR242C	4.6843
YLR421C	YMR314W	4.684
YLR397C	YPL093W	4.684
YDR194C	YOR310C	4.684
YER133W	YHR158C	4.6837
YDL101C	YDR369C	4.6837
YMR190C	YOL090W	4.6832
YLR234W	YOL090W	4.6832
YJR144W	YOL090W	4.6832
YGR193C	YPL259C	4.6832
YDR295C	YOL090W	4.6832
YFR037C	YPR106W	4.6827
YNL307C	YOL142W	4.6826
YMR072W	YOR304W	4.6816
YCR066W	YML085C	4.6814
YLR335W	YMR308C	4.6812
YLR134W	YNL124W	4.6812
YDL052C	YEL002C	4.6812
YLL060C	YLR043C	4.6811
YDR416W	YLR132C	4.681
YDL014W	YLL011W	4.6805
YGL173C	YKL003C	4.6799
YNL208W	YPL004C	4.6793
YKL012W	YLR424W	4.6792
YDL160C	YER035W	4.6792
YJR041C	YMR297W	4.679
YMR146C	YNL287W	4.6789
YDR432W	YNL132W	4.6787
YDL060W	YDR064W	4.6786
Q0055	YLL004W	4.6784
YCR008W	YLR305C	4.6778
YDR099W	YMR296C	4.6776
YDR062W	YDR099W	4.6776
YLR341W	YOL022C	4.6774
YGL027C	YOL022C	4.6774
YGL027C	YLR341W	4.6774
YER041W	YOL022C	4.6774
YER041W	YLR341W	4.6774
YER041W	YGL027C	4.6774
YKL060C	YOR155C	4.6772
YKL060C	YMR055C	4.6772
YER102W	YJR007W	4.6772
YBR247C	YNL308C	4.6771
YDR303C	YOL002C	4.6767
YDR303C	YDR372C	4.6767
YJL081C	YML007W	4.6765
YGL137W	YNL255C	4.6759
YDR069C	YIL084C	4.6759
YMR309C	YNL040W	4.6754
YGL081W	YMR309C	4.6754
YCR084C	YDL076C	4.6752
YGR017W	YLL024C	4.6739
YLR197W	YML093W	4.6736
YBL035C	YPL106C	4.6732
YKL172W	YOR310C	4.6727
YLR259C	YOR356W	4.6717
YLR259C	YMR189W	4.6717
YLR089C	YLR259C	4.6717
YKL116C	YLR259C	4.6717
YGL236C	YLR259C	4.6717
YDR267C	YLR259C	4.6717
YCR060W	YGR270W	4.6711
YDR347W	YJR060W	4.671
YCL054W	YFL002C	4.6696
YHR066W	YKR081C	4.6692
YDL134C	YHR158C	4.6691
YBR195C	YPL022W	4.6687
YAL029C	YLR092W	4.6684
YKL085W	YKL157W	4.6682
YGL019W	YOL086C	4.6682
YGL244W	YJL087C	4.6677
YBL075C	YNL055C	4.6677
YDL213C	YLL008W	4.6672
YGL019W	YLR409C	4.6671
YMR223W	YOL067C	4.667
YBL103C	YMR223W	4.667
YEL034W	YLR262C-A	4.666
YJR077C	YOL086C	4.6658
YNL096C	YNL244C	4.6655
YLR335W	YNL221C	4.6649
YJL107C	YJL138C	4.6649
YDR099W	YFR053C	4.6648
YBL046W	YDL112W	4.6644
YCR053W	YPL028W	4.6641
YCR053W	YDR353W	4.6641
YDL084W	YLR432W	4.6636
YAR014C	YLR028C	4.6636
YGL058W	YOL016C	4.6635
YDR166C	YNL172W	4.6635
YDR074W	YMR213W	4.6635
YDL095W	YEL036C	4.6635
YCR043C	YDL095W	4.6635
YBR188C	YDR074W	4.6635
YIL076W	YNL255C	4.6633
YER021W	YNL047C	4.6629
YJL183W	YLR342W	4.6628
YDL051W	YLR074C	4.6628
YBR136W	YDL156W	4.6626
YKR092C	YOR195W	4.6625
YIL159W	YKR092C	4.6625
YAL005C	YJR052W	4.6622
YDR155C	YLR044C	4.6616
YGL097W	YPL037C	4.6615
YDR448W	YNR010W	4.6615
YJR104C	YMR315W	4.6609
YGL030W	YOR234C	4.6608
YJL047C	YLR289W	4.6596
YGR196C	YNL094W	4.6594
YDR156W	YFR028C	4.659
YBL046W	YPL106C	4.6586
YML074C	YNL209W	4.6585
YLR172C	YNL287W	4.6585
YBR031W	YLR075W	4.6581
YAL003W	YBR118W	4.6579
YGR209C	YLR043C	4.6578
YGL120C	YMR288W	4.6574
YGR123C	YOR027W	4.6572
YLL040C	YMR078C	4.657
YGL200C	YKL192C	4.657
YGR252W	YLR071C	4.6566
YDR044W	YOR257W	4.6563
YBR122C	YLR360W	4.6563
YGL066W	YML024W	4.6562
YCR088W	YLL026W	4.6562
YDR490C	YNL071W	4.656
YLR221C	YOR108W	4.6552
YLR221C	YOL058W	4.6552
YLR221C	YNL104C	4.6552
YGR026W	YML072C	4.6552
YFR004W	YOR086C	4.655
YGR192C	YML103C	4.6547
YBR048W	YER025W	4.6547
YDL065C	YGL121C	4.6545
YGR211W	YOR158W	4.6541
YBR118W	YML111W	4.6539
YIL070C	YLR354C	4.6533
YLL045C	YLR196W	4.6526
YOR244W	YPL139C	4.6521
YLR432W	YNL262W	4.6518
YCR035C	YNL307C	4.6518
YBR189W	YGR081C	4.6518
YHR089C	YNL009W	4.6508
YDR460W	YIL128W	4.6504
YHL030W	YPR103W	4.6485
YJL081C	YJR082C	4.6484
YDR174W	YKL112W	4.6483
YDL019C	YMR109W	4.6483
YDR418W	YJR123W	4.6482
YDR328C	YJL149W	4.6482
YDR328C	YHR122W	4.6482
YDR219C	YDR328C	4.6482
YJL041W	YKL210W	4.648
YGR078C	YJL041W	4.648
YER151C	YJR066W	4.6479
YMR303C	YOR217W	4.6473
YGL133W	YPR110C	4.647
YGL167C	YGR231C	4.6469
YDR044W	YDR167W	4.6469
YDL014W	YPL012W	4.6469
YAR007C	YPL075W	4.6469
YER155C	YNL201C	4.6467
YBR055C	YOL069W	4.6466
YBL075C	YHR021C	4.6464
YFL017W-A	YKR022C	4.6459
YCR060W	YER136W	4.6459
YGR215W	YJR060W	4.6458
YGR145W	YMR093W	4.6458
YBR211C	YDR104C	4.6458
YBL058W	YJR053W	4.6458
YDR071C	YFR009W	4.6451
YAR042W	YLL024C	4.6447
YML109W	YPL152W	4.6442
YLL022C	YOR303W	4.6442
YHR200W	YLR278C	4.644
YBR229C	YDR238C	4.644
YMR092C	YNL138W	4.6437
YLR447C	YMR307W	4.6437
YAL026C	YLR447C	4.6437
YML026C	YOR056C	4.6433
YGR210C	YLR222C	4.6432
YER016W	YMR086W	4.6432
YDR121W	YPL090C	4.6424
YDR473C	YNR011C	4.6423
YOL041C	YPL009C	4.6422
YLL011W	YOR310C	4.6421
YGR161C	YLR179C	4.6419
YBR121C	YGR204W	4.6408
YBR227C	YER025W	4.6407
YBR039W	YJR007W	4.6405
YDR064W	YGR054W	4.6402
YAL036C	YCL037C	4.6399
YGR233C	YHR066W	4.6398
YDR272W	YKR054C	4.6387
Q0140	YGR165W	4.6387
YML105C	YPR172W	4.6384
YML096W	YML105C	4.6384
YFR009W	YML105C	4.6384
YOR370C	YPR017C	4.6377
YIL021W	YLR450W	4.6377
YGR211W	YNL137C	4.6375
YGL241W	YPL106C	4.6371
YLR005W	YNL023C	4.637
YDR311W	YNL023C	4.637
YML032C	YMR190C	4.6369
YLR234W	YML032C	4.6369
YJR144W	YML032C	4.6369
YBR152W	YLR275W	4.6367
YLR129W	YNL075W	4.6365
YDR353W	YPL028W	4.6364
YGR234W	YPL129W	4.6362
YBR025C	YOL022C	4.6359
YBR025C	YLR341W	4.6359
YBR025C	YGL027C	4.6359
YBR025C	YER041W	4.6359
YMR226C	YNL172W	4.6358
YGL062W	YOL088C	4.6354
YDR179C	YOL088C	4.6354
YMR064W	YOL123W	4.6352
YHR099W	YLR399C	4.6349
YGL207W	YGR203W	4.6346
YGL207W	YGR163W	4.6346
YGL207W	YGR098C	4.6346
YCR015C	YGL207W	4.6346
YGR240C	YMR262W	4.6341
YLR072W	YLR249W	4.634
YDR108W	YOR259C	4.6335
YDR228C	YNL317W	4.6334
YKR029C	YOR279C	4.6329
YDR098C	YPL016W	4.6329
YDR074W	YJR050W	4.6329
YGL201C	YPR103W	4.6327
YGL019W	YJL024C	4.6327
YDR179W-A	YPR103W	4.6327
YDR011W	YPR103W	4.6327
YIL034C	YOL146W	4.632
YER004W	YFR009W	4.632
YEL032W	YIL034C	4.632
YDR489W	YIL034C	4.632
YGR142W	YKR007W	4.6315
YGL023C	YNL002C	4.6315
YEL013W	YLR371W	4.6315
YDR131C	YOL005C	4.6315
YCR076C	YKR007W	4.6315
YCR076C	YGR142W	4.6315
YBR264C	YGR232W	4.6315
YBR187W	YKR007W	4.6315
YBR187W	YGR142W	4.6315
YBR187W	YCR076C	4.6315
YNL230C	YPL001W	4.6314
YDL100C	YOR326W	4.6314
YGL207W	YLR410W	4.6313
YFL039C	YOR326W	4.6313
YLR197W	YPL009C	4.631
YOR151C	YPR110C	4.6307
YHL030W	YIL128W	4.6303
YJL122W	YPL093W	4.6295
YDL014W	YOR145C	4.6293
YBR191W	YLR441C	4.6292
YBR135W	YPR119W	4.6284
YLR197W	YPL012W	4.6283
YDR427W	YMR259C	4.6283
YLR319C	YPR108W	4.6282
YNL090W	YOR089C	4.6279
YDL195W	YJR042W	4.6279
YDL195W	YGL092W	4.6279
YDL116W	YDL195W	4.6279
YDR093W	YIL112W	4.6277
YMR093W	YOL086C	4.6276
YJL076W	YMR173W	4.6268
YGR091W	YHR156C	4.6266
YDR432W	YLR432W	4.6266
YNL306W	YOR204W	4.6261
YOR335C	YOR341W	4.6251
YCL059C	YGL120C	4.6249
YBL024W	YER165W	4.6249
YMR297W	YPL048W	4.6248
YGL123W	YML073C	4.6247
YGR252W	YHR058C	4.6245
YAL036C	YHR064C	4.6245
YER006W	YLR325C	4.6241
YGR192C	YMR113W	4.6239
YGR192C	YML086C	4.6239
YGR270W	YPL235W	4.6238
YPL031C	YPL128C	4.6234
YDR127W	YMR186W	4.6234
YLR075W	YLR342W	4.6233
YAL043C	YDR228C	4.6232
YFL007W	YGR253C	4.6226
YGR192C	YML041C	4.6224
YDL150W	YJL148W	4.6223
YCR016W	YKR081C	4.6223
YAL017W	YMR227C	4.6221
YDR382W	YLR448W	4.622
YCL059C	YDR496C	4.6219
YEL051W	YMR224C	4.6216
YBR142W	YGR103W	4.6215
YDR163W	YDR482C	4.6213
YBL046W	YGR063C	4.6211
YBL004W	YDR174W	4.6209
YHR020W	YJR068W	4.6207
YLR244C	YPL213W	4.62
YBR030W	YPL213W	4.62
YDR454C	YLR262C-A	4.6199
YAR073W	YPR067W	4.6197
YAR073W	YGR062C	4.6197
YAR073W	YAR075W	4.6197
YBR133C	YHR158C	4.6191
YLR197W	YOL093W	4.619
YER151C	YLR373C	4.619
YHR027C	YLR106C	4.6177
YPL181W	YPR184W	4.6175
YBL090W	YDR494W	4.6175
YAL034C	YPL181W	4.6175
YLR455W	YOR217W	4.6173
YDR083W	YOR217W	4.6173
YOR001W	YPR110C	4.6171
YBL002W	YGL150C	4.6169
YGR013W	YGR091W	4.6168
YGR054W	YOR290C	4.6164
YER179W	YKL211C	4.616
YDL140C	YDR343C	4.6156
YLR069C	YMR186W	4.6155
YJL011C	YNL039W	4.6155
YDR171W	YGR130C	4.6151
YBR025C	YPL225W	4.6149
YDL111C	YIR035C	4.6148
YJL173C	YMR190C	4.6146
YJL173C	YLR234W	4.6146
YJL173C	YJR144W	4.6146
YNL312W	YPL075W	4.6138
YKR002W	YLR448W	4.6137
YML117W	YPL237W	4.6136
YLR276C	YNL061W	4.6136
YCL008C	YHR013C	4.6135
YGR233C	YPR143W	4.6132
YGL245W	YMR297W	4.6132
YDR477W	YOR018W	4.6132
YGL100W	YLR359W	4.6129
YKL008C	YKL092C	4.6124
YDR294C	YKL092C	4.6124
YDR473C	YLR424W	4.6118
YDR060W	YLR196W	4.6106
YMR145C	YOR317W	4.61
YBR121C	YLL018C	4.61
YER088C	YIL084C	4.6096
YFR048W	YLR044C	4.6095
YIR001C	YNL138W	4.6093
YGL061C	YOR304C-A	4.609
YGL190C	YOR386W	4.6089
YKL009W	YKL172W	4.6088
YER083C	YKL082C	4.6088
YGL055W	YLR342W	4.6082
YDR394W	YNL047C	4.6082
YDR285W	YOR116C	4.6077
YOR370C	YOR375C	4.6067
YGR130C	YKL028W	4.6067
YER095W	YML075C	4.6067
YBR143C	YJL016W	4.6066
YOR061W	YPR133C	4.6062
YAR007C	YMR072W	4.6059
YDL134C	YKR027W	4.6049
YDR162C	YLR058C	4.6048
YBR080C	YLR246W	4.6048
YBR039W	YLR398C	4.6048
YDR304C	YHL008C	4.6046
YDR248C	YDR304C	4.6046
YBR187W	YOL041C	4.6044
YER070W	YNL134C	4.6041
YJL109C	YPL266W	4.6039
YDL043C	YLL036C	4.6036
Q0140	YHL004W	4.603
YDL087C	YDR432W	4.6026
YER052C	YER155C	4.6025
YIL009C-A	YMR314W	4.6024
YIL061C	YNL189W	4.6023
YFR019W	YIL077C	4.6023
YBR202W	YIL034C	4.6023
YKL172W	YLR196W	4.6019
YER021W	YMR191W	4.6012
YDR412W	YMR078C	4.6011
YKR060W	YPR137W	4.6009
YGL190C	YJR138W	4.6
YGR145W	YMR128W	4.5999
YER126C	YNL182C	4.5994
YMR106C	YOR027W	4.5989
YHR027C	YLR320W	4.5985
YDR028C	YNL076W	4.5979
YDR028C	YGR048W	4.5979
YCR042C	YMR089C	4.5973
YGL234W	YHR020W	4.5972
YBL058W	YBR155W	4.5972
YOR209C	YOR257W	4.596
YAL054C	YOR257W	4.596
YLR298C	YML049C	4.5957
YIL068C	YKL103C	4.5953
YGR159C	YHR186C	4.5953
YGR130C	YHR186C	4.5953
YBR114W	YJR052W	4.5953
YDR299W	YLR409C	4.595
YLR045C	YML085C	4.5948
YKL015W	YLL010C	4.5948
YKL009W	YLL034C	4.5948
YOR373W	YPL020C	4.5945
YOL108C	YPL020C	4.5945
YGL061C	YPL020C	4.5945
YKL155C	YPR185W	4.5943
YGR084C	YPR185W	4.5943
YGL180W	YKL155C	4.5943
YGL180W	YGR084C	4.5943
YGL129C	YPR185W	4.5943
YGL129C	YGL180W	4.5943
YDR141C	YEL022W	4.5935
YBL026W	YPR057W	4.5928
YDR460W	YOR173W	4.5927
YML100W	YMR251W	4.5926
YLR411W	YML100W	4.5926
YIR032C	YML100W	4.5926
YIL177C	YML100W	4.5926
YDL006W	YMR236W	4.5924
YGR158C	YIR035C	4.5921
YAL032C	YPL064C	4.5919
YAL013W	YLR057W	4.5919
YDR311W	YGL207W	4.5918
YBL045C	YKL029C	4.5917
YLR022C	YNL163C	4.5915
YLR022C	YLR185W	4.5915
YDR055W	YOR261C	4.5915
YBR114W	YOL097C	4.5914
YBR114W	YCR092C	4.5914
YOR287C	YPR178W	4.5913
YBR088C	YDR172W	4.5911
YIL021W	YPL090C	4.591
YBR205W	YGR026W	4.591
YLR284C	YOR207C	4.5908
YBR049C	YKR001C	4.5898
YDR240C	YLL036C	4.5895
YCR057C	YGL171W	4.5891
YDL147W	YOR069W	4.589
YLR028C	YLR389C	4.5884
YKL092C	YKL142W	4.5884
YBL072C	YGR090W	4.5884
YGR090W	YOL010W	4.5882
YHR200W	YNL115C	4.5875
YNL055C	YNR010W	4.5874
YNL055C	YNL260C	4.5874
YNL008C	YNL055C	4.5874
YIL142W	YJL106W	4.5874
YGR233C	YPL088W	4.5874
YDL156W	YIL142W	4.5874
YJL080C	YPL160W	4.5865
YGR255C	YMR075W	4.5863
YDR190C	YPL115C	4.5861
YMR049C	YPR008W	4.5856
YLR114C	YMR049C	4.5856
YLR257W	YPL106C	4.5854
YER093C	YGL071W	4.5847
YMR158W	YPR166C	4.5841
YMR158W	YNL081C	4.5841
YMR064W	YMR286W	4.5841
YJL096W	YMR064W	4.5841
YEL036C	YOR254C	4.584
YEL036C	YLR292C	4.584
YBR171W	YEL036C	4.584
YKL186C	YLR208W	4.5838
YCL034W	YPL231W	4.5837
YDR364C	YKL095W	4.5836
YLR044C	YMR303C	4.5835
YDL051W	YHR197W	4.5833
YLR175W	YNL124W	4.5828
YHR066W	YNL308C	4.5828
YBR169C	YPL106C	4.5828
YBR049C	YGL133W	4.5826
YLR392C	YOR038C	4.5824
YMR146C	YNL044W	4.5823
YDR473C	YJL035C	4.5819
YDR427W	YPL002C	4.5817
YDL045W-A	YMR188C	4.5817
YIR035C	YNL232W	4.5809
YBR017C	YHR020W	4.5809
YCR063W	YNR011C	4.5806
YJL034W	YML012W	4.5803
YLR044C	YOR316C	4.5801
YCR069W	YLR044C	4.5801
YLR117C	YPL064C	4.58
YGR162W	YIL061C	4.5798
YBL004W	YBR247C	4.5793
YEL002C	YGL008C	4.5791
YCR077C	YOR234C	4.5791
YGR103W	YLR347C	4.5787
YBR080C	YDR363W-A	4.5784
YLR449W	YPL198W	4.5783
YBR247C	YGR090W	4.5778
YKL010C	YPL259C	4.5777
YGL210W	YLR262C	4.5777
YBR143C	YLR389C	4.5777
YER048C	YPL233W	4.5776
YBR109C	YDR356W	4.5776
YJR065C	YOR367W	4.5773
YHR016C	YJR065C	4.5773
YFL034C-A	YGL067W	4.5773
YDR012W	YNL232W	4.5773
YER070W	YPL137C	4.5768
YDR058C	YKL028W	4.5765
YEL056W	YOR303W	4.5756
YDL002C	YNL027W	4.5754
YHR034C	YOR141C	4.5751
YDL051W	YPR137W	4.5751
YBR279W	YOR056C	4.5749
YMR093W	YPL266W	4.5744
YJL041W	YJL050W	4.5743
YIL036W	YPL194W	4.574
YDR453C	YML028W	4.574
YLL011W	YLR449W	4.5736
YDR164C	YPR100W	4.5734
YML046W	YOR159C	4.5732
YDR001C	YGL252C	4.573
YDL031W	YER126C	4.5728
YDL063C	YKL212W	4.5717
YHR082C	YKL210W	4.5714
YLR249W	YLR424W	4.5713
YBR114W	YOR332W	4.5713
YCL054W	YMR290C	4.5706
YOL090W	YPR179C	4.5705
YNL021W	YOL090W	4.5705
YAL024C	YDL188C	4.5704
YGR165W	YJL063C	4.5701
YBR006W	YGR165W	4.5701
YJR045C	YPR065W	4.57
YGL076C	YNL075W	4.5699
YGL156W	YNL151C	4.5696
YER110C	YJL183W	4.5691
YOR201C	YOR319W	4.569
YKR101W	YMR223W	4.569
YDR143C	YOR144C	4.569
YOL139C	YPL260W	4.5685
YKR059W	YLL026W	4.5685
YIL094C	YPL240C	4.5683
YHR062C	YNL246W	4.5681
YCL059C	YNL255C	4.5676
YBR011C	YNL023C	4.5671
YDR337W	YJR060W	4.5661
YLR382C	YLR424W	4.5656
YBL104C	YLR424W	4.5656
YLR277C	YPL240C	4.5654
YFR019W	YGR086C	4.5652
YGL055W	YPL050C	4.5645
YBR162C	YDR238C	4.5644
YJR113C	YOR204W	4.5641
YER143W	YPL235W	4.5639
YDR081C	YPL235W	4.5639
YDL195W	YIL109C	4.5637
YAL021C	YDL149W	4.5637
YFR037C	YJL176C	4.5632
YBR025C	YLR153C	4.5632
YFR024C-A	YOR061W	4.5629
YBL026W	YMR213W	4.5629
YKR081C	YMR049C	4.5623
YDR449C	YPL106C	4.5623
YJR084W	YMR125W	4.5622
YGR097W	YMR304W	4.5622
YDR166C	YIR007W	4.5622
YNL136W	YOR244W	4.562
YBR048W	YCL059C	4.5619
YMR263W	YPR184W	4.5614
YLR276C	YOL127W	4.5614
YAL034C	YMR263W	4.5614
YGR061C	YLL029W	4.5606
YGR129W	YLL036C	4.5605
YGL238W	YJR077C	4.5605
YBR055C	YPR101W	4.5605
YKR014C	YOR375C	4.5604
YKL181W	YMR231W	4.5604
YKL181W	YLR148W	4.5604
YBL058W	YDR028C	4.5604
YGL156W	YOR210W	4.5603
YDR300C	YNL265C	4.5603
YDR300C	YGR194C	4.5603
YDR369C	YEL051W	4.5602
YML057W	YOR326W	4.5601
YBR121C	YDL124W	4.5601
YNL035C	YPL266W	4.56
YDR499W	YOR176W	4.56
YBR145W	YOR176W	4.56
YHR059W	YIL070C	4.5593
YJL109C	YLR175W	4.5592
YHR170W	YOR351C	4.5592
YDL014W	YOL102C	4.5592
YDR434W	YKL039W	4.5587
YER172C	YJR032W	4.5582
YDR450W	YNL069C	4.5581
YER177W	YOL063C	4.5565
YER177W	YNR034W	4.5565
YER177W	YJL047C	4.5565
YDL105W	YDR365C	4.5561
YML124C	YNL064C	4.556
YNR050C	YOR270C	4.5558
YMR080C	YNL016W	4.5558
YDR104C	YGR179C	4.5557
YJL164C	YLR275W	4.5554
YAL002W	YBR227C	4.5547
YGL016W	YKL218C	4.5546
YJL177W	YOL127W	4.5545
YLR403W	YMR190C	4.5544
YBR175W	YLR403W	4.5544
YML085C	YOR005C	4.5542
YDR496C	YHR197W	4.5539
YGL147C	YNL061W	4.5538
YGR116W	YNL049C	4.5536
YCL054W	YNL110C	4.5533
YCR009C	YPR062W	4.5531
YJL124C	YML049C	4.5528
YER062C	YPR033C	4.5522
YLL024C	YPR072W	4.5519
YBR247C	YDR064W	4.5516
YBR109C	YPR171W	4.5515
YHR034C	YLR370C	4.5514
YKR083C	YMR083W	4.551
YGR195W	YNL307C	4.5509
YBR025C	YIL075C	4.55
YDR335W	YEL022W	4.5498
YOR032C	YPR183W	4.549
YKL150W	YPR183W	4.549
YKL016C	YPR183W	4.549
YHR128W	YPR183W	4.549
YDR523C	YLL041C	4.549
YDR372C	YDR523C	4.549
YDR298C	YPR183W	4.549
YDR490C	YER178W	4.5479
YAL034W-A	YER048C	4.5479
YIL062C	YLR429W	4.5478
YBR085W	YMR056C	4.5473
YDR394W	YMR191W	4.5467
YKL213C	YOR116C	4.5465
YHR167W	YJR032W	4.5463
YML098W	YMR227C	4.5455
YML070W	YNL248C	4.5452
YDR245W	YPL094C	4.5452
YDR026C	YNL248C	4.5452
YLR432W	YOR312C	4.5442
YBR265W	YML100W	4.5441
YBL023C	YML100W	4.5441
YGL206C	YOL063C	4.544
YGL206C	YNR034W	4.544
YGL206C	YJL047C	4.544
YDR060W	YER006W	4.5438
YAL005C	YJR148W	4.5437
YFL026W	YGL238W	4.5436
YGR178C	YKR068C	4.543
YKL009W	YOL077C	4.5421
YNL039W	YNR003C	4.542
YGL076C	YKL035W	4.5415
YDR364C	YNR011C	4.5413
YGL150C	YOR304W	4.541
YGR003W	YOR326W	4.5409
YOR093C	YOR319W	4.5404
YKL204W	YPL106C	4.5402
YNL110C	YPL211W	4.5401
YDL193W	YLR293C	4.54
YCR063W	YER013W	4.54
YBL006C	YGR056W	4.5396
YDR381W	YML055W	4.5394
YPL151C	YPR029C	4.5392
YML062C	YPL179W	4.5379
YAR007C	YNL001W	4.5376
YOR108W	YOR117W	4.5375
YGL049C	YLR430W	4.5372
YBR055C	YLR117C	4.5371
YMR108W	YPL164C	4.537
YAL005C	YBR056W	4.537
YER007C-A	YPL006W	4.5369
YCL054W	YGR237C	4.5369
YOR319W	YPR178W	4.5361
YGR095C	YLR398C	4.5361
YDR496C	YGR145W	4.536
YGR034W	YPR025C	4.5359
YLR275W	YPL151C	4.5358
YLR074C	YNL182C	4.5357
YAL017W	YGR094W	4.5356
YLR355C	YOR098C	4.5355
YAR002C-A	YHR110W	4.5355
YAR002C-A	YDL018C	4.5355
YJL061W	YKL210W	4.5353
YGR078C	YJL061W	4.5353
YBR139W	YDR416W	4.5353
YGL127C	YLR290C	4.5351
YDL100C	YKL082C	4.5351
YIL068C	YOL059W	4.535
YIL068C	YLR368W	4.535
YHL007C	YIL068C	4.535
YER125W	YGL087C	4.535
YDL022W	YIL068C	4.535
YDR226W	YIL050W	4.5347
YBL036C	YDR226W	4.5347
YAL003W	YDR226W	4.5347
YBR181C	YGR034W	4.5346
YLR153C	YOR335C	4.5344
YBR006W	YHL004W	4.5343
YJL087C	YOL145C	4.5342
YLR367W	YOR056C	4.5341
YHL025W	YKR001C	4.534
YNR035C	YOR233W	4.5337
YIR037W	YNL135C	4.5337
YDR335W	YGL238W	4.5337
YBR159W	YNR035C	4.5337
YGL076C	YLL045C	4.5335
YEL002C	YMR231W	4.5331
YEL002C	YLR148W	4.5331
YAR003W	YDR069C	4.5331
YJR002W	YJR145C	4.533
YCR009C	YGL252C	4.5328
YBR088C	YIL152W	4.5326
YGR145W	YLR175W	4.5318
YHR143W-A	YNL113W	4.5317
YDR365C	YEL019C	4.5314
YKR081C	YPL012W	4.5313
YJL080C	YPR189W	4.5312
YGL252C	YOR335C	4.5306
YDR342C	YOR160W	4.5305
YGR255C	YNL330C	4.5303
YOL097C	YOR332W	4.5299
YGR155W	YLR079W	4.5299
YBR143C	YPL179W	4.5298
YBL074C	YDL098C	4.5298
YCR012W	YMR301C	4.5293
YCR012W	YER174C	4.5293
YMR128W	YPL266W	4.5287
YIL078W	YNL135C	4.5286
YBL104C	YGR074W	4.5286
YDR028C	YGR159C	4.5285
YCL031C	YDL014W	4.5275
YGR130C	YPL004C	4.5273
YMR125W	YPR023C	4.5271
YOL005C	YOR014W	4.5267
YKR007W	YPR124W	4.5267
YHR075C	YOL005C	4.5267
YGR161C	YOL005C	4.5267
YGR142W	YPR124W	4.5267
YCR076C	YPR124W	4.5267
YBR187W	YPR124W	4.5267
YGR238C	YPR159W	4.5266
YGR047C	YPR159W	4.5266
YFR003C	YFR015C	4.5266
YDR477W	YGR016W	4.5266
YDR128W	YJR141W	4.5266
YDR128W	YJL173C	4.5266
YBR245C	YOR141C	4.5263
YOR151C	YPL198W	4.5262
YBR121C	YFR009W	4.5262
YMR022W	YNR031C	4.5261
YGL147C	YNL069C	4.5261
YBR281C	YMR093W	4.5261
YBR281C	YGL171W	4.5261
YIL126W	YOR207C	4.5254
YFR019W	YPL004C	4.5254
YJR077C	YOR160W	4.5251
YHR103W	YKL191W	4.525
YAL020C	YKL191W	4.525
YDR227W	YOR116C	4.5248
YDR412W	YOL077C	4.5245
YLL029W	YNL247W	4.5244
YLL021W	YPL020C	4.5242
YER012W	YGL048C	4.5242
YDR517W	YJL099W	4.5242
YDR434W	YML032C	4.5242
YDL192W	YLR052W	4.5242
YGR232W	YKL184W	4.5239
YER111C	YPL128C	4.5239
YER089C	YPL128C	4.5239
YER089C	YER111C	4.5239
YDR465C	YMR196W	4.5239
YDL175C	YPR108W	4.5238
YIL106W	YPR160W	4.5236
YDL220C	YGR090W	4.5234
YDR037W	YNL118C	4.5233
YER178W	YNL107W	4.5232
YBL074C	YDR416W	4.5226
YDR409W	YGR126W	4.5222
YCR033W	YDL185W	4.5219
YGL246C	YGR048W	4.5217
YCR063W	YDR163W	4.5217
YML019W	YMR146C	4.5216
YGR211W	YPL118W	4.5212
YER021W	YGR135W	4.5212
YDL082W	YJL177W	4.5212
YGL122C	YPL169C	4.521
YDR155C	YER043C	4.5208
YJR053W	YOR272W	4.5205
YMR205C	YOL016C	4.519
YDL156W	YJL014W	4.519
YBL027W	YNL302C	4.5183
YAL059W	YNL110C	4.5183
YMR205C	YPR065W	4.518
YMR205C	YPL161C	4.518
YJL157C	YMR205C	4.518
YLL001W	YNL183C	4.5178
YDL075W	YNL110C	4.5173
YKL214C	YLR430W	4.517
YFL003C	YLR430W	4.517
YKR092C	YPR181C	4.5167
YBL027W	YER147C	4.516
YBL027W	YDL003W	4.516
YBR072W	YER159C	4.5158
YDR166C	YJL138C	4.5157
YNR003C	YPL082C	4.5156
YBL030C	YNR001C	4.5153
YMR224C	YNL287W	4.5151
YLR455W	YOR064C	4.5151
YDL181W	YOR159C	4.5149
YDL130W-A	YOR159C	4.5149
YHR158C	YMR273C	4.5137
YDR328C	YPR164W	4.5137
YGR186W	YPR180W	4.5129
YGR005C	YPR180W	4.5129
YGL004C	YPR108W	4.5128
YGL004C	YKL145W	4.5128
YDR328C	YPL217C	4.5128
YDR328C	YLR274W	4.5128
YDR328C	YKL176C	4.5128
YDR328C	YEL032W	4.5128
YDR312W	YDR328C	4.5128
YBL023C	YDR328C	4.5128
YAL005C	YLR177W	4.5128
YDR343C	YMR275C	4.5114
YNL221C	YNL246W	4.5113
YKL144C	YNL084C	4.5113
YKL152C	YMR186W	4.5112
YMR229C	YOR272W	4.5107
YKL157W	YML065W	4.5107
YHR099W	YPL139C	4.5104
YGR270W	YLL021W	4.5102
YDR028C	YGR270W	4.5102
YGR161C	YOR267C	4.5098
YER074W	YHL001W	4.5095
YDR485C	YKL142W	4.5093
YLR175W	YOL041C	4.5092
YBR274W	YKL104C	4.5089
YLR196W	YNL175C	4.5088
YGR234W	YLL013C	4.5086
YJR145C	YNL075W	4.5085
YOR145C	YPR112C	4.5071
YDL045W-A	YER050C	4.5068
YDL045W-A	YDR494W	4.5068
YGL129C	YJR060W	4.5067
YKL145W	YMR191W	4.5066
YBL047C	YNL243W	4.5066
YCR053W	YOR335C	4.5065
YBL006C	YFL008W	4.5064
YDR224C	YNR003C	4.5062
YBR188C	YDR163W	4.5061
YML069W	YMR172W	4.506
YJL095W	YPL140C	4.5058
YMR224C	YPR175W	4.5056
YBR143C	YPL083C	4.5054
YBR143C	YLR386W	4.5054
YDR382W	YGR027C	4.5051
YBR055C	YHR086W	4.5049
YOR244W	YPR040W	4.5048
YMR005W	YPR040W	4.5048
YGL017W	YPR040W	4.5048
YFL024C	YPR040W	4.5048
YDL229W	YGR017W	4.5047
YGR233C	YMR092C	4.5046
YCR012W	YOL126C	4.5046
YFL037W	YPL061W	4.5044
YJL203W	YLR382C	4.5042
YBL104C	YJL203W	4.5042
YHR089C	YNL061W	4.5038
YDR055W	YIL075C	4.5036
YLR371W	YPL169C	4.5033
YLR371W	YNL127W	4.5033
YLR197W	YNL088W	4.5033
YGL251C	YLR371W	4.5033
YGL131C	YLR371W	4.5033
YFL013C	YPL053C	4.5033
YFL013C	YHR034C	4.5033
YNL247W	YNL306W	4.503
YMR304W	YNR031C	4.5021
YOR370C	YPL228W	4.5019
YGL130W	YOR370C	4.5019
YKL196C	YOR212W	4.5018
YHR158C	YML109W	4.5018
YDR264C	YKL196C	4.5018
YCL014W	YDR164C	4.5018
YDR347W	YPR185W	4.5017
YDR347W	YGL180W	4.5017
YDL140C	YML124C	4.5016
YBR025C	YNL108C	4.5014
YDL126C	YMR308C	4.5011
YER103W	YOL078W	4.501
YGL100W	YML019W	4.5008
YJR007W	YPL206C	4.5007
YDR364C	YER013W	4.5007
YBR095C	YCR084C	4.5006
YBR065C	YLR424W	4.4993
YDL029W	YDR190C	4.4987
YFL002C	YNL110C	4.4985
YKL081W	YMR223W	4.4984
YKL012W	YLR382C	4.4982
YIL069C	YPR029C	4.4978
YHR052W	YIL127C	4.4978
YER082C	YNL075W	4.4978
YCR042C	YKL182W	4.4978
YJL197W	YPL237W	4.4976
YGL043W	YPR008W	4.4976
YPL206C	YPR041W	4.4973
YJR144W	YMR137C	4.4972
YLR276C	YMR308C	4.4969
YMR224C	YPL010W	4.4962
YCR077C	YMR224C	4.4962
YLL006W	YLR249W	4.4961
YFR052W	YLR359W	4.4961
YFR052W	YKL165C	4.4961
YFR052W	YJR120W	4.4961
YFR052W	YJR062C	4.4961
YBR109C	YNL208W	4.4959
YDR225W	YIL126W	4.4955
YDR054C	YIL046W	4.4955
YDL164C	YDR054C	4.4955
YGR209C	YHR179W	4.4954
YDR101C	YOR063W	4.4954
YER021W	YGL004C	4.4953
YEL013W	YPL037C	4.4953
YBR251W	YNR036C	4.4952
YML075C	YMR089C	4.4948
YKL157W	YPR162C	4.4948
YGR056W	YJL137C	4.4944
YBL003C	YGL150C	4.4943
YER177W	YGR240C	4.494
YER167W	YPL115C	4.494
YDR171W	YKL157W	4.4936
YER155C	YPL243W	4.4933
YMR089C	YNL244C	4.4925
YER027C	YER177W	4.4923
YDR422C	YER177W	4.4923
YGR245C	YLR074C	4.4915
YDR299W	YNR054C	4.4913
YDR415C	YGR204W	4.4909
YGR167W	YKL069W	4.4907
YLR424W	YML046W	4.4904
YJL033W	YPL198W	4.4902
YKR095W	YNL025C	4.4901
YDL006W	YDR145W	4.4897
YDR295C	YLL029W	4.4895
YBR283C	YLR023C	4.4894
YBR283C	YDR021W	4.4894
YHR174W	YOR032C	4.4892
YHR174W	YOL016C	4.4892
YHR174W	YMR153W	4.4892
YBL079W	YHR174W	4.4892
YDR087C	YKR081C	4.4889
YDR064W	YPR189W	4.4888
YLR241W	YPR184W	4.4887
YLR241W	YPL247C	4.4887
YLR241W	YLR270W	4.4887
YKR001C	YLR241W	4.4887
YFL016C	YPR183W	4.4887
YCR084C	YDR523C	4.4887
YDR190C	YMR284W	4.4885
YDR164C	YLR189C	4.4881
YBL072C	YLR186W	4.488
YGR180C	YMR083W	4.4877
YDR005C	YNL113W	4.4877
YDR121W	YLR432W	4.4876
YDR093W	YMR135C	4.4876
YBR148W	YER006W	4.4875
YGL011C	YIL009C-A	4.4873
YPL211W	YPR169W	4.4872
YNR014W	YOR133W	4.4869
YGL244W	YOR056C	4.4863
YOR056C	YOR178C	4.486
YOL054W	YOR056C	4.486
YGR279C	YKL205W	4.486
YDL043C	YLR058C	4.486
YJL138C	YKL060C	4.4859
YCR035C	YIR035C	4.4856
YDR482C	YPL151C	4.4854
YGR252W	YPR070W	4.4852
YJR084W	YKL173W	4.4845
YGL246C	YGR060W	4.4842
YBL058W	YGL246C	4.4842
YBR158W	YHR064C	4.4841
YOL041C	YPL129W	4.484
YDL148C	YOR310C	4.4838
YGR095C	YPR189W	4.4836
YGR075C	YPR082C	4.4834
YCL040W	YDL014W	4.4833
YKL188C	YML049C	4.4831
YKL120W	YML127W	4.4828
YIL035C	YMR075W	4.4828
YGL137W	YLR019W	4.4827
YLR274W	YPL249C	4.4811
YDL006W	YNR058W	4.4807
YGR217W	YNL088W	4.4805
YJR094W-A	YML072C	4.4804
YJL026W	YJR053W	4.4802
YER151C	YHL034C	4.4801
YGL019W	YPR133C	4.4794
YOR335C	YPL028W	4.479
YDR353W	YOR335C	4.479
YDL030W	YDR295C	4.4787
YDR450W	YIL133C	4.4786
YML006C	YNR052C	4.4781
YHR011W	YJL069C	4.4781
YHL035C	YKL059C	4.4781
YEL013W	YER172C	4.4781
YBR264C	YLR262C	4.4781
YAL027W	YIL068C	4.4781
YJL005W	YOL001W	4.478
YDL132W	YLL039C	4.4779
YCR014C	YPR160W	4.4776
YIL093C	YJL063C	4.4775
YDR190C	YJR065C	4.4775
YML091C	YPR181C	4.4771
YJR134C	YPR181C	4.4771
YGL197W	YPR181C	4.4771
YEL015W	YPR181C	4.4771
YML007W	YPL254W	4.4769
YBR261C	YDL060W	4.4769
YER087W	YML117W	4.4768
YBR044C	YER178W	4.4768
Q0140	YDR041W	4.4767
YGR071C	YPL031C	4.4766
YCR014C	YKL145W	4.4765
YLR357W	YOR304W	4.4762
YDL022W	YJL117W	4.4762
YIL143C	YNL085W	4.4758
YIL033C	YOR089C	4.4755
YDR163W	YJR050W	4.4755
YBL003C	YGR047C	4.4755
YBL003C	YHR099W	4.4754
YGR162W	YJL190C	4.4753
YDR127W	YKL104C	4.4749
YER102W	YNL132W	4.4742
YDR429C	YGR270W	4.4737
YMR116C	YPR041W	4.4736
YBR048W	YNL308C	4.4734
YOR054C	YOR061W	4.4733
YGR254W	YJR045C	4.4732
YBR087W	YKR035W-A	4.4732
YLL024C	YMR167W	4.473
YGL146C	YOR005C	4.4729
YBL049W	YIL017C	4.4729
YLR075W	YPL198W	4.4727
YKL082C	YLR002C	4.4727
YDL193W	YJR132W	4.4723
YPL146C	YPR169W	4.4722
YAL005C	YOL016C	4.4722
YBR198C	YOR304W	4.4721
YOL013C	YPL240C	4.472
YIL125W	YKL007W	4.472
YIL034C	YIL125W	4.472
YHL014C	YPL240C	4.472
YFR016C	YIL125W	4.472
YBR086C	YPR086W	4.4719
YMR173W	YOR341W	4.4718
YLR345W	YMR227C	4.4718
YKL099C	YPL126W	4.4717
YBR121C	YFL045C	4.4715
YER178W	YGR002C	4.4714
YMR125W	YOR003W	4.4713
YAL038W	YFL037W	4.4705
YAL005C	YDR122W	4.4704
YDR194C	YLR432W	4.4702
YDR499W	YOL090W	4.4692
YKL007W	YOR284W	4.4681
YKL007W	YOL146W	4.4681
YHR027C	YOR108W	4.4681
YEL032W	YKL007W	4.4681
YDR489W	YKL007W	4.4681
YBL003C	YFL013C	4.4679
YOR061W	YPL181W	4.4676
YLR424W	YMR213W	4.467
YGR196C	YIL142W	4.4669
YOR004W	YPR112C	4.4668
YEL036C	YGL167C	4.4668
YHL038C	YLR175W	4.4667
YDL160C	YEL015W	4.4667
YER029C	YJR050W	4.466
YAR003W	YJL026W	4.4654
YCR009C	YDR129C	4.4653
YLR410W	YMR140W	4.4645
YAL059W	YNR053C	4.4645
YBL058W	YPR135W	4.4644
YHR131C	YIL148W	4.4642
YDL175C	YKL214C	4.4639
YDL175C	YFL003C	4.4639
YJR022W	YPR057W	4.4638
YFR009W	YHR020W	4.4637
YBL076C	YBR058C	4.4636
YDR087C	YPL009C	4.4634
YBR087W	YCL028W	4.4625
YDR406W	YML029W	4.4624
YBR205W	YKL212W	4.4622
YDR127W	YMR299C	4.4617
YBL045C	YPR010C	4.4617
YJL050W	YJL061W	4.4616
YIL069C	YPL259C	4.4616
YGR061C	YKL155C	4.4615
YGR061C	YGR084C	4.4615
YGL129C	YGR061C	4.4615
YGL111W	YLR276C	4.4613
YOR294W	YPR043W	4.4612
YER002W	YPR043W	4.4612
YCL009C	YNR016C	4.4612
YMR167W	YOR232W	4.4611
YGL163C	YOR232W	4.4611
YBR223C	YCL028W	4.4611
YHR021C	YNL055C	4.4609
YHR020W	YOR160W	4.4609
YDL092W	YLR432W	4.46
YHL033C	YLR115W	4.4599
YHR027C	YNL004W	4.4595
YHL033C	YKL212W	4.4595
YCR057C	YOL010W	4.4594
YDR145W	YML024W	4.4593
YDL052C	YPL050C	4.4592
YDL052C	YML115C	4.4592
YLL022C	YML065W	4.459
YNR052C	YOR110W	4.4586
YNL288W	YOR110W	4.4586
YNR011C	YPR178W	4.4585
YJL180C	YKL054C	4.4584
YGL173C	YPL004C	4.4582
YAL001C	YDR155C	4.4578
YLR354C	YNL189W	4.4576
YHR139C	YOR076C	4.4575
YJL123C	YLR347C	4.4574
YBR009C	YBR114W	4.457
YDR064W	YHL015W	4.4566
YDL140C	YML123C	4.4564
YDL101C	YJL115W	4.4564
YKR048C	YPL128C	4.4559
YGL241W	YPL128C	4.4559
YAL017W	YGR264C	4.4559
YOR304W	YPR175W	4.4557
YDR235W	YJR084W	4.4557
YKR014C	YPL228W	4.4556
YJL148W	YPL255W	4.4556
YGL130W	YKR014C	4.4556
YDR050C	YOL060C	4.4556
YDR028C	YMR261C	4.4556
YGL135W	YPL220W	4.4554
YDR328C	YOR080W	4.4553
YDR155C	YPL084W	4.4551
YDR155C	YIL162W	4.4551
YDL185W	YOR136W	4.4549
YDR229W	YJL074C	4.4545
YBR280C	YPL258C	4.4545
YAL047C	YJL074C	4.4545
YNL002C	YOL127W	4.4543
YGR075C	YPL213W	4.4536
YER075C	YMR229C	4.4533
YDL047W	YDR075W	4.4533
YHR076W	YIL070C	4.4532
YGL207W	YGR270W	4.4532
YDL047W	YGR270W	4.4526
YBR039W	YOR142W	4.4523
YDL014W	YNL308C	4.452
YML017W	YOL139C	4.4514
YGR067C	YJL080C	4.4512
YOR205C	YPL013C	4.4511
YKL003C	YOR205C	4.4511
YHR059W	YKL003C	4.4511
YGR150C	YPL013C	4.4511
YGR150C	YKL003C	4.4511
YDL045W-A	YPL013C	4.4511
YDL045W-A	YKL003C	4.4511
YDL002C	YER088C	4.4509
YIR035C	YOR001W	4.4508
YNL139C	YNR031C	4.4504
YBR105C	YPL061W	4.4504
YJR075W	YLR342W	4.4502
YDL185W	YPL201C	4.4502
YER043C	YKL161C	4.4498
YFL037W	YHR020W	4.4489
YER031C	YER052C	4.4489
YNL189W	YOL004W	4.4488
YER053C	YMR203W	4.4481
YLR071C	YNL189W	4.4478
YKL015W	YOR043W	4.4472
YGL073W	YPL006W	4.4472
YGL133W	YPL235W	4.4471
YDR457W	YLR436C	4.4471
YDR457W	YDR538W	4.4471
YDL066W	YDR457W	4.4471
YFR015C	YFR030W	4.447
YDL043C	YGR250C	4.447
YDL006W	YML100W	4.447
YOR299W	YPR054W	4.4469
YBR142W	YDL168W	4.4467
YOL123W	YPL266W	4.4466
YGR282C	YKR046C	4.4463
YLR381W	YPL243W	4.4462
YDR318W	YPL243W	4.4462
YDR254W	YPL243W	4.4462
YBR009C	YMR091C	4.4462
YNL216W	YPL128C	4.446
YNL271C	YPR019W	4.4445
YLR309C	YPR019W	4.4445
YJL157C	YOL059W	4.4445
YJL157C	YLR368W	4.4445
YHR010W	YNR053C	4.4445
YHL007C	YJL157C	4.4445
YDL022W	YJL157C	4.4445
YBR202W	YPR019W	4.4445
YDL060W	YER131W	4.4436
YJR104C	YKL067W	4.4431
YDR496C	YOR312C	4.4428
YBR121C	YDL185W	4.4422
YGR218W	YMR246W	4.442
YER094C	YML022W	4.4419
YER012W	YML022W	4.4419
YCR088W	YGR080W	4.4418
YJR105W	YNL106C	4.4417
YBR055C	YMR120C	4.4417
YGL195W	YNL323W	4.4416
YGL195W	YLR326W	4.4416
YDR138W	YMR190C	4.4416
YBR175W	YDR138W	4.4416
YDR240C	YDR515W	4.4415
YBR055C	YDL209C	4.4412
YDL098C	YKL012W	4.4407
YMR286W	YPR100W	4.4406
YJL096W	YPR100W	4.4406
YDR299W	YPL198W	4.4406
YDL135C	YNL209W	4.4405
YBR143C	YCR012W	4.4405
YBL090W	YOR204W	4.44
YDR462W	YHR147C	4.4399
YIL063C	YNL041C	4.4387
YDR416W	YPR029C	4.4387
YML057W	YOR261C	4.4386
YLR406C	YNR053C	4.4385
YNL071W	YOR069W	4.4384
YDR304C	YGL164C	4.4384
YBR202W	YKL007W	4.4384
YJL034W	YNL247W	4.4382
YJL034W	YLR359W	4.4382
YHR098C	YJL034W	4.4382
YIL094C	YJL138C	4.4378
YCR072C	YNL002C	4.4378
YJR094W-A	YOR086C	4.4377
YJL098W	YKR027W	4.4377
YDL156W	YOL090W	4.4377
YMR089C	YMR205C	4.4372
YJR045C	YOR232W	4.4372
YCR079W	YIL160C	4.4365
YGR135W	YKL210W	4.4363
YDR415C	YGR135W	4.4363
YCR082W	YMR308C	4.4363
YCR060W	YMR309C	4.4358
YDL156W	YER091C	4.4357
YHR023W	YLR314C	4.4353
YDL175C	YGL049C	4.4353
YER062C	YPL247C	4.435
YER062C	YIL045W	4.435
YAL007C	YLR342W	4.435
YBR123C	YDR155C	4.4349
YHR131C	YOR039W	4.4348
YDR108W	YPR160W	4.4344
YER003C	YIR001C	4.4342
YMR263W	YOR061W	4.434
YER006W	YOL127W	4.434
YBL041W	YOR259C	4.4339
YBR278W	YLR432W	4.4337
YAL013W	YPR184W	4.4337
YAL013W	YAL034C	4.4337
YAR002W	YNL224C	4.4333
YMR146C	YPL237W	4.4331
YER112W	YLR438C-A	4.4331
YBR018C	YDL145C	4.433
YJR050W	YLR147C	4.4327
YER177W	YLR423C	4.432
YER177W	YIL095W	4.432
YDR128W	YGL205W	4.4318
YDR128W	YER182W	4.4318
YDR128W	YEL062W	4.4318
YIL076W	YIL129C	4.4314
YER070W	YLR258W	4.431
YHL001W	YMR142C	4.4309
YDR432W	YGR250C	4.4309
YLR333C	YML056C	4.4301
YDR328C	YLL050C	4.4301
YDL124W	YJL167W	4.4298
YGL120C	YKR092C	4.4297
YER165W	YOR206W	4.4295
YDR475C	YER133W	4.429
YHR099W	YMR005W	4.4283
YLR222C	YNR054C	4.4282
YGR085C	YMR142C	4.4281
YCL050C	YML095C	4.4281
YBR283C	YNL055C	4.4279
YIR001C	YJL033W	4.4274
YGL143C	YIL018W	4.4274
YDL082W	YDL136W	4.4271
YNL186W	YPL013C	4.4259
YKL003C	YNL186W	4.4259
YIL084C	YPL059W	4.4259
YBR126C	YMR251W	4.4258
YBR126C	YLR411W	4.4258
YBR126C	YIR032C	4.4258
YBR126C	YIL177C	4.4258
YEL044W	YLR052W	4.4257
YBR055C	YHR156C	4.4257
YKL172W	YPR169W	4.4251
YGL103W	YGR085C	4.4249
YDR064W	YNL207W	4.4245
YER006W	YKL172W	4.4244
YDR436W	YML049C	4.4244
YGL227W	YPL061W	4.4243
YOL108C	YPL211W	4.424
YGR234W	YIL050W	4.424
YAL003W	YGR234W	4.424
YBR079C	YER103W	4.4238
YOR158W	YPR166C	4.4237
YNL081C	YOR158W	4.4237
YDL150W	YKL179C	4.4237
YBR114W	YNL312W	4.4237
YGL049C	YGR090W	4.4233
YBL037W	YLL029W	4.4232
YDR499W	YML032C	4.423
YBR136W	YMR012W	4.423
YAL012W	YPL106C	4.4224
YGR232W	YMR259C	4.4223
YNL281W	YOL016C	4.4219
YDL200C	YEL024W	4.4219
YMR116C	YNL172W	4.4218
YLR370C	YNL040W	4.4217
YGL179C	YNL138W	4.4217
YBR103W	YMR155W	4.4217
YBR103W	YML109W	4.4217
YBR103W	YGR296W	4.4217
YBR103W	YEL064C	4.4217
YKL139W	YLR403W	4.4216
YHR119W	YLR403W	4.4216
YDR168W	YLR403W	4.4216
YCL054W	YOL077C	4.4216
YGR041W	YPR121W	4.4212
YFL053W	YPR121W	4.4212
YDR316W	YPR121W	4.4212
YAL005C	YFR053C	4.4208
YBR247C	YLL011W	4.4205
YGR245C	YLR325C	4.4203
YBL056W	YPL204W	4.4199
Q0080	YDR342C	4.4199
YPL010W	YPL219W	4.4197
YML028W	YNL249C	4.4196
YDL098C	YIL061C	4.4194
YER029C	YLL036C	4.4192
YLR274W	YOL146W	4.419
YDR489W	YLR274W	4.419
YBR011C	YDR033W	4.4187
YBR010W	YNL055C	4.4187
YAL005C	YOR275C	4.4179
YAL005C	YOR274W	4.4179
YAL005C	YOR216C	4.4179
YAL005C	YOR171C	4.4179
YAL005C	YOR074C	4.4179
YAL005C	YMR104C	4.4179
YAL005C	YLR090W	4.4179
YAL005C	YJL171C	4.4179
YAL005C	YDR481C	4.4179
YAL005C	YDR399W	4.4179
YAL005C	YDR258C	4.4179
YAL005C	YDR217C	4.4179
YAL005C	YCR036W	4.4179
YLR421C	YMR191W	4.4178
YFR052W	YOR069W	4.4177
YFR052W	YJL204C	4.4177
YCR014C	YDL097C	4.4177
YOR145C	YPL204W	4.4175
YIL094C	YJL095W	4.4171
YKR024C	YOL120C	4.417
YER165W	YNL262W	4.4166
YGL122C	YPL266W	4.4164
YBR017C	YPL266W	4.4164
YIL034C	YPL153C	4.4161
YFR019W	YFR040W	4.4161
YGL190C	YHR132W-A	4.4158
YLL005C	YNR051C	4.4157
YDR141C	YPL115C	4.4144
YBR143C	YOR069W	4.4136
YNR037C	YPR166C	4.4132
YNL081C	YNR037C	4.4132
YGR215W	YPR166C	4.4132
YGR215W	YNL081C	4.4132
YER050C	YNR037C	4.4132
YDR028C	YNR047W	4.4132
YBL003C	YJL074C	4.4131
YDR382W	YMR194W	4.4129
YBL072C	YMR093W	4.4128
YBL072C	YDR449C	4.4128
YDL229W	YPL226W	4.4126
YGL135W	YLR029C	4.4125
YDR001C	YPL216W	4.4121
YBL047C	YML091C	4.4121
YBL047C	YJR134C	4.4121
YBL047C	YHR098C	4.4121
YBL047C	YGL197W	4.4121
YBL047C	YEL015W	4.4121
YNL137C	YPR185W	4.4119
YGL180W	YNL137C	4.4119
YDL229W	YOR138C	4.4119
YJL050W	YMR125W	4.4117
YDR099W	YLR176C	4.4117
YGL195W	YLR106C	4.4115
YDR174W	YOR304W	4.4114
YBL104C	YBR039W	4.4114
YBL075C	YPL228W	4.4114
YBL075C	YGL130W	4.4114
YCR012W	YDR099W	4.4113
YGR167W	YLL005C	4.4112
YDR243C	YFL034C-A	4.4112
YNL132W	YOL041C	4.4106
YDL213C	YJR041C	4.4106
YIL061C	YJL035C	4.4099
YBR084W	YBR263W	4.4099
YBL041W	YDL007W	4.4098
YMR225C	YOR201C	4.4096
YML009C	YOR201C	4.4096
YEL013W	YGR279C	4.4095
YCR088W	YNL106C	4.4093
YDR012W	YOR001W	4.4092
YOL078W	YPR137W	4.4088
YDR190C	YER143W	4.4086
YER103W	YMR309C	4.4085
YML085C	YMR205C	4.4084
YIL035C	YMR287C	4.4079
YAL011W	YOL052C	4.4078
YCL054W	YLR449W	4.4077
YMR080C	YMR083W	4.4071
YML010W	YPR180W	4.4071
YGL141W	YMR109W	4.407
YFR013W	YOL017W	4.4062
YCL050C	YPL183C	4.4053
YJL173C	YLR347C	4.4051
YDL030W	YGR075C	4.405
YKL017C	YPL106C	4.4049
YGL039W	YPL106C	4.4049
YAL032C	YDR074W	4.4049
YER112W	YFL017W-A	4.4042
YGR047C	YNR003C	4.4041
YOR261C	YPL088W	4.4039
YNL308C	YOR206W	4.4035
YNL084C	YNL151C	4.4035
YNL323W	YOL086C	4.403
YJL094C	YOL086C	4.403
YER037W	YOL086C	4.403
YDR061W	YOL086C	4.403
YDL051W	YLR432W	4.4028
YBL072C	YLL011W	4.4028
YML119W	YOR157C	4.4023
YGL201C	YOR157C	4.4023
YDR179W-A	YOR157C	4.4023
YDR011W	YOR157C	4.4023
YKL108W	YLR354C	4.4022
YHR186C	YNL201C	4.4022
YFR003C	YLR258W	4.4021
YOL086C	YPR074C	4.4019
YGR229C	YLR342W	4.4016
YJR092W	YKL157W	4.4014
YHR052W	YPR031W	4.4012
YGL167C	YPL143W	4.4012
YDR342C	YPL143W	4.4012
YDR342C	YGL167C	4.4012
YDL055C	YIL070C	4.401
YFL049W	YKR001C	4.4009
YIL142W	YKR028W	4.4006
YDR499W	YJL173C	4.4006
YBL075C	YER103W	4.4004
YBR088C	YPR018W	4.3998
YBL047C	YLR079W	4.3996
YDR050C	YOR187W	4.3995
YBR158W	YGL207W	4.3995
YDR416W	YKL012W	4.3992
YDR382W	YOL040C	4.3992
YHR158C	YOR093C	4.3981
YDL014W	YJL033W	4.398
YHR202W	YPR168W	4.3977
YER082C	YOR145C	4.3977
YLR409C	YMR155W	4.3972
YLR409C	YML109W	4.3972
YLR129W	YPL265W	4.3972
YJL069C	YPL265W	4.3972
YGR296W	YLR409C	4.3972
YEL064C	YLR409C	4.3972
YDR106W	YLR129W	4.3972
YDR106W	YJL069C	4.3972
YBR057C	YLR129W	4.3972
YBR057C	YJL069C	4.3972
YBL026W	YGL117W	4.3972
YBL026W	YDL111C	4.3972
YBL026W	YCL050C	4.3972
YNL224C	YPL020C	4.3971
YGR175C	YNL272C	4.3971
YGR155W	YOR151C	4.3971
YKL172W	YOL127W	4.397
YGR192C	YNL201C	4.3969
YIL033C	YMR022W	4.3966
YCR012W	YMR055C	4.3966
YDR171W	YPL106C	4.3963
YKL039W	YMR004W	4.3953
YHR027C	YKL142W	4.395
YKR048C	YNL102W	4.3949
YBR169C	YKR048C	4.3949
YBR065C	YNR011C	4.3942
YLR246W	YMR191W	4.394
YML098W	YPL011C	4.3936
YLR115W	YNL050C	4.3934
YKR025W	YNL232W	4.3933
YLR117C	YOR279C	4.393
YKL182W	YNL244C	4.393
YDR074W	YLR117C	4.393
YDR138W	YPL179W	4.3929
YDR342C	YLR347C	4.3928
YLR371W	YMR052W	4.3927
YLR264W	YOR145C	4.3927
YAL051W	YIL036W	4.3927
YER103W	YKL088W	4.3925
YDR449C	YMR229C	4.3925
YDR388W	YGL252C	4.392
YDR245W	YKR061W	4.3919
YDR245W	YKR031C	4.3919
YDR245W	YJL139C	4.3919
YDR382W	YNL175C	4.3918
YDR285W	YHR081W	4.3916
YJL148W	YLR143W	4.3915
YJR064W	YJR065C	4.3914
YFL039C	YPR040W	4.3914
YFL039C	YNL108C	4.3914
YDR028C	YPR035W	4.3914
YDL156W	YML032C	4.3914
YAR014C	YLL021W	4.3903
YIL112W	YNL189W	4.3902
YDR155C	YIL112W	4.3902
YAL034C	YJR123W	4.3901
YDR171W	YEL056W	4.3898
YKR062W	YML063W	4.3897
YOL018C	YPL002C	4.3896
YGR056W	YNL307C	4.3896
YCR053W	YML056C	4.3896
YBR202W	YLR274W	4.3893
YBR081C	YJL176C	4.3892
YEL034W	YLL026W	4.3885
YMR033W	YPR106W	4.3881
YLR233C	YOR319W	4.3877
YKL204W	YKR007W	4.3877
YGR142W	YKL204W	4.3877
YDR359C	YPR040W	4.3877
YDR052C	YOR259C	4.3877
YCR076C	YKL204W	4.3877
YBR187W	YKL204W	4.3877
YAL027W	YJL157C	4.3877
YOR326W	YPL040C	4.3876
YMR071C	YOR326W	4.3876
YGR188C	YOR326W	4.3876
YIL021W	YPR093C	4.3872
YBR221C	YDR430C	4.387
YGL207W	YJL087C	4.3869
YDL051W	YJR002W	4.3869
YER070W	YKL193C	4.3868
YBL003C	YNL030W	4.3867
YDR141C	YHR066W	4.3863
YDR343C	YNL032W	4.3862
YGL175C	YIL095W	4.3859
YOR257W	YPL116W	4.3858
YBR272C	YPL001W	4.3857
YNL256W	YPR178W	4.3855
YMR205C	YOR155C	4.3853
YMR205C	YNL333W	4.3853
YGR195W	YIR035C	4.3848
YNL284C	YOR201C	4.3847
YGR264C	YPL129W	4.3847
YDR164C	YNL284C	4.3847
YDL075W	YDR064W	4.3846
YBR088C	YGR090W	4.3843
YDR447C	YMR194W	4.3838
YDR069C	YPL138C	4.3837
YDR069C	YLR015W	4.3837
YCL031C	YNL231C	4.3837
YCL031C	YJL004C	4.3837
YOL001W	YPL010W	4.3835
YCR014C	YOR117W	4.3835
YCR014C	YGL048C	4.3835
YMR128W	YPL198W	4.3829
YDL031W	YLR276C	4.3829
YCL037C	YMR230W	4.3829
YDL006W	YER178W	4.3828
YMR201C	YPL022W	4.3826
YER162C	YPL022W	4.3826
YJR017C	YPR169W	4.3825
YGR056W	YNR003C	4.3823
YCR092C	YNL312W	4.3823
YER093C	YJL130C	4.3817
YDR005C	YJL011C	4.3816
YDL014W	YOR078W	4.3816
YNL265C	YOR260W	4.3815
YHR023W	YMR236W	4.3815
YGR194C	YOR260W	4.3815
YNL069C	YPL143W	4.3812
YHR059W	YOR158W	4.3812
YGL013C	YLR410W	4.3809
YBR189W	YGL031C	4.3809
YFR040W	YNR016C	4.3808
YBR127C	YLR347C	4.3801
YOR145C	YPR144C	4.3795
YJR042W	YPL085W	4.3795
YGL092W	YPL085W	4.3795
YER053C	YLR333C	4.3795
YDL116W	YPL085W	4.3795
YHL027W	YMR190C	4.3794
YHL027W	YLR234W	4.3794
YHL027W	YJR144W	4.3794
YGL147C	YKL009W	4.3792
YDR045C	YNR024W	4.3789
YOR319W	YPR094W	4.3788
YGR278W	YKL095W	4.3787
YHR013C	YLR119W	4.3784
YGR270W	YPL128C	4.3781
YER111C	YGR270W	4.3781
YER089C	YGR270W	4.3781
YER149C	YLR249W	4.3776
YKL214C	YPR057W	4.3774
YFL003C	YPR057W	4.3774
YMR213W	YNR011C	4.3773
YBR126C	YBR265W	4.3773
YBL023C	YBR126C	4.3773
YGR080W	YOR233W	4.3772
YBR159W	YGR080W	4.3772
YDR299W	YGL171W	4.3771
YEL030W	YKL191W	4.3758
YCL050C	YDR385W	4.3749
YGR145W	YOR206W	4.3743
YER082C	YPL012W	4.3742
YLR432W	YPL012W	4.3741
YBR114W	YDL225W	4.3736
YOR187W	YPR029C	4.3733
YOL010W	YOR187W	4.3733
YLR328W	YOR187W	4.3733
YKL135C	YOR187W	4.3733
YGL115W	YGL158W	4.373
YKL120W	YLR321C	4.3729
YDR227W	YLR278C	4.3729
YDR229W	YLR436C	4.3728
YDR229W	YDR538W	4.3728
YDR156W	YOR335C	4.3728
YDL066W	YDR229W	4.3728
YKL009W	YLR449W	4.3727
YMR109W	YOL146W	4.3722
YGL106W	YOL146W	4.3722
YEL032W	YMR109W	4.3722
YEL032W	YGL106W	4.3722
YDR489W	YMR109W	4.3722
YDR489W	YGL106W	4.3722
YAL017W	YDR167W	4.3722
YHR200W	YOL022C	4.3719
YHR200W	YLR341W	4.3719
YGL027C	YHR200W	4.3719
YER041W	YHR200W	4.3719
YDL014W	YGR283C	4.3718
YHL004W	YIL070C	4.3699
YIL112W	YOR279C	4.3696
YLL008W	YNL175C	4.3693
YDR190C	YMR072W	4.3693
YHR121W	YPL228W	4.3691
YGL130W	YHR121W	4.3691
YDR394W	YKL007W	4.3691
YGR126W	YNL293W	4.3689
YDR347W	YGR061C	4.3689
YGR252W	YML024W	4.3686
YNL271C	YPR089W	4.3681
YLR259C	YNL055C	4.368
YMR091C	YPR106W	4.3662
YLL039C	YPL046C	4.3661
YCR093W	YDL165W	4.3661
YBR208C	YMR209C	4.3661
YAR003W	YLL039C	4.3661
YDL112W	YDR075W	4.3659
YDR171W	YLR304C	4.3656
YER110C	YPL078C	4.3654
YBR039W	YER062C	4.3653
YDL030W	YMR125W	4.365
YLR382C	YML046W	4.3649
YJR068W	YKR035W-A	4.3649
YBL104C	YML046W	4.3649
YML062C	YOR304W	4.3646
YGL133W	YML062C	4.3646
YJL034W	YLR243W	4.3643
YDL126C	YHR092C	4.3643
YDR388W	YOR136W	4.3638
YDR225W	YML127W	4.3638
YKL143W	YPL012W	4.3635
YDR496C	YPL012W	4.3633
YLR019W	YMR246W	4.3629
YGR135W	YLR035C	4.3627
YKR025W	YNL039W	4.3626
YJL138C	YML028W	4.3626
YHL033C	YKR002W	4.3624
YIL104C	YMR064W	4.3622
YLL026W	YOR133W	4.3619
YDR170C	YKL205W	4.3616
YKR046C	YMR145C	4.3615
YER122C	YLR246W	4.3611
YER095W	YLR425W	4.3611
YLR325C	YPR016C	4.3602
YHL033C	YIL018W	4.3602
YGL133W	YNR003C	4.3598
YDR141C	YPR143W	4.3598
YDL087C	YLR424W	4.3598
YBL074C	YDR240C	4.3598
YDR108W	YKL145W	4.3596
YDL136W	YOL127W	4.3596
YDR243C	YFL039C	4.3592
YGL174W	YOR319W	4.3591
YDR432W	YPL016W	4.3589
YBR010W	YCL029C	4.3587
YFR030W	YJL123C	4.3586
YGR180C	YPL153C	4.3585
YGL100W	YPR101W	4.3585
YDL006W	YGR252W	4.3584
YBR154C	YGR140W	4.3583
YBR154C	YCL044C	4.3583
YBR080C	YER021W	4.3581
YDR238C	YMR214W	4.3579
YBR203W	YKR048C	4.3577
YGR261C	YJL076W	4.3571
YDR293C	YLR436C	4.3571
YDR293C	YDR538W	4.3571
YDL066W	YDR293C	4.3571
YDL007W	YNL115C	4.3571
YAL029C	YKR035W-A	4.3571
YIL130W	YPR052C	4.3568
YLR039C	YNL096C	4.3567
YIL143C	YOR027W	4.3567
YGR095C	YNL265C	4.3566
YGR095C	YGR194C	4.3566
YJL191W	YPR102C	4.3563
YDR471W	YMR242C	4.3562
YNL110C	YOL127W	4.3559
YFL013C	YFL024C	4.3558
YBL084C	YKR064W	4.3558
YDR194C	YOL041C	4.3557
YLR430W	YPR161C	4.3554
YLR226W	YLR430W	4.3554
YCR030C	YIL070C	4.3549
YDR171W	YPL023C	4.3544
YDR171W	YGL125W	4.3544
YBR065C	YER013W	4.3536
YKL188C	YLR438C-A	4.3535
YBR055C	YGL173C	4.3535
YER053C	YJL066C	4.3534
YDL061C	YML109W	4.3533
YKL179C	YNL151C	4.353
YOL145C	YOR056C	4.3529
YBR189W	YDL148C	4.3529
YJR082C	YLR399C	4.3528
YDL101C	YMR275C	4.3526
YDR473C	YMR213W	4.3524
YLR347C	YML060W	4.3522
YGL026C	YOL058W	4.3522
YDR021W	YDR211W	4.3522
YNL189W	YOR244W	4.3505
YML127W	YOL002C	4.3504
YDR372C	YML127W	4.3504
YGL081W	YJL074C	4.3498
YCR052W	YFL049W	4.3498
YGL019W	YNL097C	4.3489
YER007C-A	YGL073W	4.3487
YDL209C	YLR147C	4.3487
YCR077C	YDR369C	4.3487
YDL188C	YHR158C	4.3486
YDR432W	YGR013W	4.3483
YIL149C	YLR249W	4.348
YHR074W	YPL240C	4.3476
YER132C	YPL240C	4.3476
YDR444W	YPL240C	4.3476
YDL097C	YDR314C	4.3471
YBR127C	YLR225C	4.3469
YDR171W	YFL039C	4.3462
YKL152C	YOL086C	4.346
YDL229W	YLR373C	4.346
YLR300W	YNL002C	4.3454
YGL023C	YLR300W	4.3454
YHR099W	YOL004W	4.3452
YGR155W	YPL256C	4.3452
YDR071C	YDR416W	4.3452
YDR071C	YDR150W	4.3452
YBL066C	YDR071C	4.3452
YBL061C	YDR071C	4.3452
YFL033C	YLL040C	4.3451
YDR190C	YGL207W	4.3449
YHR027C	YMR191W	4.3445
YER035W	YIL127C	4.3445
YGL147C	YLR342W	4.3439
YGR080W	YLR203C	4.3435
YGR072W	YGR080W	4.3435
YDR121W	YFL018C	4.3433
YGR034W	YIL133C	4.3427
YBR202W	YMR109W	4.3425
YBR202W	YGL106W	4.3425
YAL017W	YGL252C	4.3425
YCR030C	YHR099W	4.3422
YBR010W	YLR109W	4.3422
YLL011W	YMR229C	4.3421
YGL127C	YJR066W	4.3421
YER103W	YPL237W	4.3421
YHR156C	YML049C	4.342
Q0140	YBR251W	4.342
YLL001W	YMR108W	4.3419
YCR063W	YOR308C	4.3416
YJL081C	YPL169C	4.341
YAL003W	YKL080W	4.3404
YOR259C	YPR103W	4.3397
YNL242W	YNL306W	4.3397
YFR052W	YIL066C	4.3397
YML073C	YMR242C	4.339
YGL173C	YKL142W	4.3387
YCR002C	YCR053W	4.3385
YDR328C	YHR039C-A	4.3378
YDR025W	YMR110C	4.3376
YER013W	YMR213W	4.3368
YPL053C	YPL240C	4.3367
YHR034C	YPL240C	4.3367
YDR334W	YPL240C	4.3367
YLR335W	YPR174C	4.3365
YGR278W	YNR011C	4.3364
YDL040C	YGR254W	4.3364
YDR012W	YPL022W	4.3362
YMR093W	YPL012W	4.336
YMR192W	YNL112W	4.3359
YKL104C	YMR205C	4.3358
YGR165W	YGR211W	4.3351
YDL102W	YDR272W	4.3351
YAR007C	YMR284W	4.3348
YDL040C	YPL198W	4.3345
YDR141C	YPL088W	4.334
YML048W	YMR196W	4.3339
YDR465C	YML048W	4.3339
YDL220C	YPL128C	4.3339
YDL220C	YER111C	4.3339
YDL220C	YER089C	4.3339
YDL171C	YMR196W	4.3339
YDL171C	YDR465C	4.3339
YEL023C	YLR262C-A	4.3338
YDL126C	YLR347C	4.3335
YMR146C	YPR117W	4.3325
YMR146C	YPL206C	4.3325
YMR146C	YNL272C	4.3325
YKR029C	YMR273C	4.3325
YDR156W	YPR133C	4.3323
YCR092C	YDL225W	4.3322
YML100W	YNL076W	4.3318
YDL147W	YML057W	4.3317
YMR304W	YOR023C	4.3311
YMR304W	YOR007C	4.3311
YJL187C	YMR304W	4.3311
YBL076C	YPL160W	4.3304
YLR153C	YPR074C	4.3301
YLR057W	YNL097C	4.3301
YDL075W	YDL082W	4.3299
YGR204W	YIL018W	4.3295
YFL017W-A	YNL147W	4.3292
YDR129C	YPR074C	4.3292
YEL031W	YPR103W	4.329
YCR076C	YPR103W	4.329
YBR139W	YMR213W	4.3289
YBR139W	YBR188C	4.3289
YAR007C	YBR245C	4.3281
YIR001C	YMR080C	4.3275
YDL167C	YMR080C	4.3275
YBR130C	YOL146W	4.3275
YBR130C	YEL032W	4.3275
YBR130C	YDR489W	4.3275
YDR036C	YHR197W	4.3273
YER177W	YPL106C	4.3271
YLR276C	YPL211W	4.3269
YDR303C	YMR072W	4.3266
YDR214W	YMR186W	4.3265
YDL150W	YDR280W	4.3264
YER029C	YPL178W	4.326
YGR192C	YPR089W	4.3259
YAL021C	YOR110W	4.3259
YJL035C	YKL012W	4.3258
YDL185W	YKL003C	4.3258
YDL185W	YIR029W	4.3258
YJR121W	YKL054C	4.3257
YHR202W	YLR071C	4.3256
YDL029W	YPR019W	4.3253
YMR203W	YMR302C	4.3252
YNL025C	YNL334C	4.3249
YMR139W	YNL199C	4.3249
YLR133W	YNL199C	4.3249
YLR133W	YMR139W	4.3249
YGR154C	YNL334C	4.3249
YGR154C	YNL025C	4.3249
YGR111W	YNL334C	4.3249
YGR111W	YNL025C	4.3249
YGR111W	YGR154C	4.3249
YER132C	YNL199C	4.3249
YER132C	YMR139W	4.3249
YER132C	YLR133W	4.3249
YER123W	YNL334C	4.3249
YER123W	YNL025C	4.3249
YER123W	YGR154C	4.3249
YER123W	YGR111W	4.3249
YER099C	YNL199C	4.3249
YER099C	YMR139W	4.3249
YER099C	YLR133W	4.3249
YER099C	YER132C	4.3249
YDR326C	YJR035W	4.3249
YBR028C	YNL334C	4.3249
YBR028C	YNL025C	4.3249
YBR028C	YGR154C	4.3249
YBR028C	YGR111W	4.3249
YBR028C	YER123W	4.3249
YBL008W	YEL061C	4.3249
YJL137C	YPL016W	4.3245
YER074W	YJL177W	4.3242
YJR009C	YML028W	4.324
YBR142W	YNL002C	4.324
YDR378C	YML049C	4.3236
YLR208W	YML019W	4.323
YGL087C	YOL081W	4.3226
YBR135W	YOR080W	4.3226
YDL105W	YDR288W	4.3222
YEL046C	YML074C	4.3219
YMR226C	YMR318C	4.3218
YER177W	YPL137C	4.3215
YDR301W	YHL035C	4.3215
YLR044C	YMR315W	4.3214
YJL177W	YML073C	4.3214
YBR121C	YLR153C	4.321
YLR057W	YOL004W	4.3209
YNL102W	YNR052C	4.3206
YNL102W	YNL288W	4.3206
YMR284W	YPR052C	4.3206
YKL045W	YNR052C	4.3206
YKL045W	YNL288W	4.3206
YIR008C	YNR052C	4.3206
YIR008C	YNL288W	4.3206
YIR007W	YMR116C	4.3206
YIL130W	YMR284W	4.3206
YDR054C	YPR164W	4.3206
YBL035C	YNR052C	4.3206
YBL035C	YNL288W	4.3206
YAR002W	YPL020C	4.3206
YDL076C	YOR244W	4.3205
YCL037C	YJL162C	4.3203
YOR227W	YOR272W	4.3201
YER073W	YJR045C	4.3201
YBR087W	YCL016C	4.32
YPL255W	YPR010C	4.3195
YGR103W	YPL220W	4.3193
YHR088W	YHR197W	4.3191
YGR258C	YKL191W	4.3186
YBR221C	YML059C	4.3184
YDR395W	YIL133C	4.3182
YMR188C	YOR204W	4.3181
YML063W	YOR056C	4.3181
YER070W	YIL066C	4.3181
YBL026W	YGR013W	4.3176
YJR119C	YLL040C	4.3175
YER110C	YJR075W	4.3175
YCR032W	YLL040C	4.3175
YGR145W	YMR116C	4.3174
YDL075W	YGR034W	4.3174
YLR277C	YLR448W	4.3165
YDR293C	YNL104C	4.3165
YGL171W	YPL126W	4.3161
YKR067W	YPL010W	4.316
YEL055C	YPL093W	4.3158
YDL007W	YPR103W	4.3157
YCR053W	YLR027C	4.3157
YBR048W	YGR145W	4.3157
YHL001W	YLR448W	4.3156
YDL047W	YDL113C	4.3154
YMR049C	YNR053C	4.3153
YDL136W	YNL067W	4.3153
YDR496C	YPR016C	4.3149
YDR071C	YNL088W	4.3148
YHL033C	YKL018W	4.3147
YCR014C	YHR027C	4.3146
YJL033W	YJR145C	4.3144
YER048C	YML095C	4.3144
YDR255C	YLR430W	4.3144
YMR128W	YOR096W	4.3143
YDR194C	YOR206W	4.3143
YER088C	YNL330C	4.3142
YDR225W	YDR243C	4.3142
YLR180W	YOR261C	4.3137
YCR084C	YNL097C	4.3137
YBR198C	YML024W	4.3135
YKR079C	YML028W	4.3132
YIL036W	YNL027W	4.3132
YIL036W	YMR070W	4.3132
YDL148C	YPL012W	4.312
YKL184W	YMR259C	4.3119
YPL106C	YPR023C	4.3117
YDR404C	YPR180W	4.3114
YDL075W	YMR194W	4.3114
YGR113W	YMR083W	4.3113
YBL035C	YJR090C	4.3113
YDL043C	YPL178W	4.3111
YER127W	YOR206W	4.3109
YDR324C	YMR229C	4.3105
YCR031C	YDL083C	4.31
YAR007C	YFR013W	4.3098
YDR163W	YGL174W	4.3094
YLR243W	YOR204W	4.3092
YJR139C	YOR204W	4.3092
YIL156W	YOR204W	4.3092
YER094C	YHR008C	4.3092
YER012W	YHR008C	4.3092
YIL129C	YLR345W	4.3091
YFR009W	YNR016C	4.3091
YDR060W	YLR449W	4.3091
YGL195W	YMR092C	4.3089
YDR138W	YHR119W	4.3089
YIR002C	YJL173C	4.3088
YDR097C	YJL173C	4.3088
YKR026C	YNL265C	4.3087
YGR194C	YKR026C	4.3087
YGR159C	YNL236W	4.3087
YFR051C	YMR224C	4.3087
YER087W	YPL190C	4.3087
YFL039C	YPL155C	4.3086
YFL039C	YHR040W	4.3086
YBR097W	YFL039C	4.3086
YDR171W	YMR031C	4.3085
YDR171W	YJR046W	4.3085
YJR017C	YLL024C	4.3083
YFL001W	YHR174W	4.308
YDR342C	YLR441C	4.3076
YER178W	YFL039C	4.3073
YKR024C	YLR432W	4.3068
YLL018C	YMR205C	4.3067
YGR281W	YPL152W	4.3062
YDR190C	YLR370C	4.3058
YBR088C	YBR195C	4.3051
YDL192W	YPL218W	4.305
YLR421C	YNL020C	4.3049
YHR216W	YMR061W	4.3049
YHR066W	YNL061W	4.3049
YBL099W	YMR186W	4.3049
YJL095W	YNL192W	4.3046
YJL095W	YKL045W	4.3046
YHR082C	YJL095W	4.3046
YER133W	YNL075W	4.3046
YER133W	YKL035W	4.3046
YDR229W	YMR001C	4.3046
YAL047C	YMR001C	4.3046
YMR224C	YNL085W	4.3043
YGR165W	YPR166C	4.3043
YGR165W	YNL081C	4.3043
YHR099W	YHR121W	4.3042
YLR197W	YOL010W	4.3038
YLR131C	YMR308C	4.3036
YDR044W	YEL030W	4.3036
YLR238W	YML123C	4.3032
YIL126W	YML123C	4.3032
YER088C	YPL139C	4.3032
YEL030W	YPL256C	4.3032
YEL030W	YIL095W	4.3032
YBR155W	YEL030W	4.3032
YGR275W	YKR099W	4.303
YDL126C	YHR205W	4.303
YAR042W	YBR143C	4.303
YJR002W	YPL061W	4.3029
YDR171W	YNL064C	4.3029
YDL213C	YHR060W	4.3029
YLR320W	YOR261C	4.3026
YIL112W	YLR409C	4.3025
YDR021W	YOR260W	4.3025
YAR007C	YER078C	4.3025
YGR075C	YMR288W	4.3024
YDL175C	YPR161C	4.3024
YDL175C	YLR226W	4.3024
YBL024W	YLR264W	4.3023
YJL011C	YPR055W	4.3021
YJL176C	YLR321C	4.302
YBL075C	YOR250C	4.3018
YBL075C	YGL044C	4.3018
YCR057C	YOR061W	4.3013
YDL097C	YDR108W	4.3011
YBR105C	YLR430W	4.3011
YGL147C	YOL040C	4.3008
YBR083W	YPR010C	4.3006
YDL029W	YPL235W	4.3005
YEL022W	YPL050C	4.2998
YHL004W	YJR060W	4.2994
YGR211W	YHL004W	4.2994
YBR065C	YML046W	4.299
YDR381W	YGL122C	4.2988
YGL070C	YPL144W	4.2987
YGL070C	YLR021W	4.2987
YEL039C	YGL070C	4.2987
YJR101W	YNL242W	4.2983
YBR139W	YJR050W	4.2983
YIL128W	YPL249C	4.2982
YBR130C	YBR202W	4.2979
YGR034W	YPL097W	4.2976
YIR005W	YJR050W	4.2973
YAL003W	YNL007C	4.2972
YDL147W	YPL088W	4.2971
YCL059C	YGR103W	4.297
YLR177W	YNR052C	4.2965
YKR022C	YOR308C	4.2965
YJL087C	YLR418C	4.296
YER013W	YGR278W	4.2959
YDL126C	YGL238W	4.2958
YER149C	YMR186W	4.2957
YDR314C	YOR117W	4.2957
YER103W	YER164W	4.2947
YGR060W	YOR153W	4.2943
YHR165C	YJL035C	4.2941
YDR378C	YLR438C-A	4.2941
YAL062W	YIL033C	4.294
YIL009C-A	YOL038W	4.2938
YFR030W	YGL016W	4.2938
YDR141C	YGL016W	4.2938
YDR515W	YER165W	4.2936
YDL195W	YKL057C	4.2934
YDL145C	YMR224C	4.2933
YDL058W	YJL016W	4.2933
YBR121C	YCR053W	4.2932
YDL193W	YGL008C	4.293
YGL173C	YLR175W	4.2928
YIL035C	YPR133C	4.2927
YBR025C	YHR200W	4.2924
YDL030W	YDR515W	4.2919
YHR186C	YML049C	4.2917
YBR130C	YBR143C	4.2917
YNL250W	YOR173W	4.2916
YER048C	YPL183C	4.2916
YGR234W	YNL106C	4.2913
YBL039C	YOR303W	4.2913
YBL039C	YML072C	4.2913
YDR164C	YJL005W	4.2912
YOR207C	YPR015C	4.2911
YDR228C	YKL018W	4.2906
YDR416W	YMR125W	4.2901
YJR084W	YLL036C	4.2892
YNL076W	YOL088C	4.2891
YGR252W	YOR023C	4.2891
YGL058W	YKL040C	4.2891
YDL058W	YKL015W	4.2891
YMR033W	YOL067C	4.289
YBL103C	YMR033W	4.289
YNL297C	YPL037C	4.2889
YBL087C	YMR049C	4.288
YDL213C	YGR145W	4.2877
YBR048W	YJR145C	4.2876
YCL037C	YDL175C	4.2875
YJL024C	YLL024C	4.287
YBL004W	YDL213C	4.2862
YDL006W	YDL140C	4.2859
YGL200C	YKL145W	4.2858
YDR238C	YMR224C	4.2857
YCR020W-B	YOR290C	4.2855
YDL075W	YGR103W	4.2854
YMR242C	YOL127W	4.2852
YKL130C	YOL146W	4.2848
YEL032W	YKL130C	4.2848
YDR489W	YKL130C	4.2848
YEL034W	YHR166C	4.2845
YDL047W	YKR027W	4.2845
YLR264W	YNL132W	4.2841
YDR272W	YJR043C	4.2841
YIL108W	YML105C	4.2838
YHR156C	YIR005W	4.2836
YBR139W	YCR079W	4.2834
YDL082W	YOR234C	4.2833
YGR192C	YPR163C	4.2831
YNL227C	YOR370C	4.283
YKL204W	YPR124W	4.283
YGL179C	YGR233C	4.2828
YKL179C	YOR207C	4.2825
YGR233C	YPL010W	4.2824
YEL022W	YML085C	4.2821
YKL014C	YNL132W	4.2819
YGL241W	YLR222C	4.2814
YDR224C	YOR056C	4.2805
YDL053C	YKL114C	4.2805
YDL053C	YFR013W	4.2805
YBR114W	YBR136W	4.2804
YKL172W	YPL043W	4.28
YGR083C	YNL265C	4.2799
YGR083C	YGR194C	4.2799
YFR037C	YOR116C	4.2796
YGR061C	YNL137C	4.2792
YDR121W	YJL065C	4.2792
YNL002C	YNR053C	4.2789
YHL011C	YNL199C	4.2787
YHL011C	YMR139W	4.2787
YHL011C	YLR133W	4.2787
YER132C	YHL011C	4.2787
YER099C	YHL011C	4.2787
YML022W	YMR287C	4.2786
YBR189W	YLR264W	4.2786
YGR145W	YLR264W	4.2781
YDR293C	YJL098W	4.2776
YAL029C	YOR144C	4.2776
YPR101W	YPR178W	4.2775
YGR074W	YJR084W	4.277
YKR048C	YPL082C	4.2766
YBL058W	YDR295C	4.2764
YGL252C	YPL249C	4.2763
YDL082W	YHL033C	4.2758
YGL227W	YLR430W	4.2749
YDL105W	YPL217C	4.2749
YAL059W	YMR124W	4.2746
YFR028C	YJL148W	4.2745
YCR031C	YOR086C	4.2745
YBR263W	YPL009C	4.2744
YDL191W	YJR058C	4.2743
YBL037W	YDL191W	4.2743
YKL191W	YPR190C	4.2741
YKL152C	YLR250W	4.2741
YKL152C	YLR080W	4.2741
YKL060C	YLR250W	4.2741
YKL060C	YLR080W	4.2741
YIL103W	YPR190C	4.2741
YFL048C	YKL152C	4.2741
YFL048C	YKL060C	4.2741
YBR048W	YPL012W	4.2736
YHR170W	YJL090C	4.2732
YIR007W	YOR133W	4.2731
YLR448W	YMR142C	4.273
YDL087C	YLR382C	4.2724
YMR309C	YOL087C	4.2722
YDL078C	YER165W	4.2718
YNL007C	YPR035W	4.2717
YLR180W	YPR108W	4.2717
YDR170C	YNL007C	4.2717
YMR242C	YNL175C	4.2711
YER110C	YOR160W	4.2711
YOL021C	YPR023C	4.271
YDL126C	YOR160W	4.2709
YDR477W	YER177W	4.2705
YLR409C	YNL075W	4.2702
YBR188C	YDR416W	4.2699
YPL081W	YPL090C	4.2698
YFL034W	YLR455W	4.2698
YBL104C	YLR216C	4.2692
YDR101C	YKR081C	4.269
YKL106W	YLR259C	4.2686
YDR268W	YLR259C	4.2686
YDR232W	YLR259C	4.2686
YDL144C	YLR259C	4.2686
YBR245C	YOR046C	4.2686
YOR210W	YPL129W	4.2684
YDR171W	YML124C	4.2684
YER063W	YIL070C	4.2681
YBL075C	YOL070C	4.2679
YKR035W-A	YNL290W	4.2674
YDR108W	YOR117W	4.2671
YDR108W	YGL048C	4.2671
YJL176C	YKR008W	4.267
YER165W	YPL190C	4.2669
YPL101W	YPL204W	4.2668
YHR187W	YPL204W	4.2668
YGR200C	YPL204W	4.2668
YDR050C	YDR111C	4.2668
YKR048C	YPL090C	4.2659
YBR121C	YPL028W	4.2659
YBR121C	YDR353W	4.2659
YBR085W	YPR025C	4.2659
YBR085W	YDL108W	4.2659
YBR085W	YBR184W	4.2659
YER006W	YNL227C	4.2658
YBL072C	YLR192C	4.2657
YML057W	YPR108W	4.2656
YKL011C	YPL022W	4.2656
YER120W	YHR196W	4.2656
YDR229W	YNL175C	4.2656
YDR074W	YER120W	4.2656
YAL047C	YNL175C	4.2656
YLR342W	YNL055C	4.2651
YKL014C	YPR143W	4.2651
YDR141C	YKL008C	4.2651
YDR141C	YDR294C	4.2651
YOL115W	YOR205C	4.265
YOL078W	YOL145C	4.265
YHR059W	YOL115W	4.265
YGR150C	YOL115W	4.265
YDL045W-A	YOL115W	4.265
YBR288C	YKL007W	4.2649
YDR418W	YML073C	4.2647
YLR421C	YOL087C	4.2644
YKL143W	YKR096W	4.2644
YIL151C	YKL143W	4.2644
YDL153C	YOR287C	4.2642
YIL063C	YML071C	4.2639
YDR093W	YOR123C	4.2638
YNL049C	YOL018C	4.2633
YBR118W	YIL074C	4.2631
YDR394W	YGL011C	4.263
YBL027W	YFR024C-A	4.2627
YAL021C	YDR190C	4.2627
YFL024C	YPL106C	4.2626
YDR052C	YFR010W	4.2622
YEL019C	YPL217C	4.2621
YBR039W	YNR022C	4.2621
YBL006C	YCR020W-B	4.2621
YCR093W	YHR027C	4.2619
YFR052W	YIR012W	4.2618
YKL009W	YLR293C	4.2614
YGR204W	YKL216W	4.2614
YNL207W	YPL012W	4.2613
YBR072W	YNL085W	4.2611
YDL190C	YIL107C	4.2608
YDR328C	YPR036W	4.2603
YER087W	YNL251C	4.2602
YGR214W	YPL260W	4.2601
YDR174W	YDR507C	4.2598
YGL103W	YMR194W	4.2596
YBR009C	YOL004W	4.2596
YDL060W	YNL132W	4.2594
YDR245W	YOR254C	4.2593
YDR245W	YLR292C	4.2593
YBR171W	YDR245W	4.2593
YBR229C	YDL145C	4.2591
YMR188C	YPR166C	4.2585
YMR188C	YNL081C	4.2585
YCL011C	YCL037C	4.2584
YHR027C	YOR181W	4.2583
YHR027C	YNR065C	4.2583
YHR027C	YLR337C	4.2583
YHR027C	YLR081W	4.2583
YHR027C	YIR012W	4.2583
YEL055C	YPL061W	4.2583
YEL055C	YPL160W	4.2582
YDR480W	YPR010C	4.2578
YIL070C	YIL093C	4.2575
YER006W	YLR196W	4.2572
YHR089C	YKL212W	4.2571
YBL002W	YIL070C	4.2567
YHL015W	YJR123W	4.2566
YKL018W	YLR448W	4.2565
YGR156W	YLR448W	4.2563
YLR197W	YNL124W	4.2562
YDL060W	YJR134C	4.2559
YDL060W	YHR098C	4.2559
YDL060W	YGL197W	4.2559
YDL060W	YEL015W	4.2559
YDR156W	YPR049C	4.2558
YBR011C	YKL143W	4.2556
YLR143W	YPR010C	4.2554
YBR229C	YNL287W	4.2554
YJR053W	YMR290C	4.2553
YBR267W	YEL054C	4.2553
YHR044C	YNL073W	4.2551
YHR044C	YHR060W	4.2551
YHR043C	YHR044C	4.2551
YGL205W	YPL258C	4.2551
YER182W	YPL258C	4.2551
YEL062W	YPL258C	4.2551
YBR202W	YKL130C	4.2551
YOR326W	YPR144C	4.255
YJL002C	YMR146C	4.255
YGL022W	YMR146C	4.255
YGL106W	YKR010C	4.2547
YBR171W	YJL117W	4.2545
YBR171W	YDL022W	4.2545
YOR097C	YOR159C	4.2541
YDL051W	YPR178W	4.2538
YLR129W	YOR061W	4.2534
YNR053C	YOR294W	4.2533
YFR004W	YML057W	4.2532
YJL203W	YJR084W	4.2529
YIL126W	YPL016W	4.2528
YLR003C	YLR129W	4.2526
YKL007W	YPL153C	4.2524
YOR375C	YPL228W	4.2521
YIL056W	YPR052C	4.2521
YIL056W	YIL130W	4.2521
YGR076C	YMR098C	4.2521
YGL130W	YOR375C	4.2521
YGL064C	YMR098C	4.2521
YDR115W	YMR098C	4.2521
YCL014W	YGR076C	4.2521
YCL014W	YGL064C	4.2521
YCL014W	YDR115W	4.2521
YLR039C	YOR293W	4.252
YKL014C	YPL211W	4.252
YDR381W	YKL214C	4.252
YDR075W	YFL014W	4.252
YDL213C	YDR516C	4.252
YLR291C	YNL265C	4.2519
YGR194C	YLR291C	4.2519
YDR037W	YER112W	4.2519
YNL071W	YPL235W	4.2518
YMR075W	YPR140W	4.2518
YDR382W	YNL069C	4.2515
YBR079C	YNL096C	4.2512
YBL049W	YGL227W	4.2511
YBL072C	YHR203C	4.251
YHR143W-A	YPR110C	4.2509
YBR181C	YNL069C	4.2509
YAL035W	YDR418W	4.2506
YJR009C	YPL069C	4.2503
YGL058W	YKL210W	4.2503
YKL152C	YPR074C	4.25
YCR084C	YPL181W	4.2496
YLR258W	YPL032C	4.249
YLR231C	YPL218W	4.249
YJR089W	YPL218W	4.249
YIL159W	YLR258W	4.249
YGL151W	YPL218W	4.249
YFR017C	YLR258W	4.249
YER133W	YNL233W	4.249
YER133W	YMR120C	4.249
YER114C	YLR258W	4.249
YDR299W	YPL218W	4.249
YDR017C	YLR258W	4.249
[truncated: 3,279,481 more chars]
