# Supplementary material for: A high-accuracy consensus map of yeast protein complexes reveals modular nature of gene essentiality
Source: BMC Bioinformatics. 2007 Jul 2;8:236. doi: 10.1186/1471-2105-8-236 (PMC1940025; doi:10.1186/1471-2105-8-236)
Supplement: Additional File 3 — Each complex is shown on a single line containing the following information: Complex ID, Size, %Essential, List of Proteins(| - deliminated). If the constituent proteins are enriched for a GO Cellular Component annotation (see Methods) then subsequent lines contain the following information: Enrichment score(-ln(p)), Simpson coefficient, Annotation name/GO_ID/(Number of proteins in GO with this annotation) [file 1471-2105-8-236-S3.htm]

```
Table S2:  E-2 Complexes					
Each complex is shown on a single line containing the following information:					
ComplexID	Size	%Essential	List of Proteins(|-delimited)		
If the constituent proteins are enriched for a GO Cellular Component annotation (see Methods) then subsequent lines contain the following information:					
			Enrichment score(-ln(p))	Simpson coefficient	Annotation name/GO_ID/(Number of proteins in GO with this annotation)

C1	35	74%	RPB5|IWR1|RPO21|RPC53|RPC11|RPA14|RPB7|DST1|RPB9|TFG2|SPT4|TFG1|RPB3|RPC17|RPB4|RPA34|RPA12|RPC25|RPC37|RPC19|RPC31|RPA49|RPC34|RPB11|RPO31|RPB2|RET1|RPB10|RPB8|RPA43|RPA190|RPA135|RPC40|RPO26|RPC82|		
			42.25	0.91	"DNA-directed RNA polymerase II, core complex/5665/(12)"
			63.74	0.94	DNA-directed RNA polymerase III complex/5666/(17)
			50.7	0.92	DNA-directed RNA polymerase I complex/5736/(14)
C2	31	58%	RPT2|RPN6|RPN5|DBF4|RAD34|SEM1|RPT3|RPN9|RPN3|RPN11|UBP6|RPN12|RPT6|NAS6|ECM29|RPN1|RPN10|RPN2|PFK26|RCY1|NTA1|YJR120W|RPT1|MCD4|YLR278C|ADE13|RPN13|RPT5|RPT4|RPN8|RPN7|		
			37.53	1	"proteasome regulatory particle, base subcomplex (sensu Eukaryota)/8540/(9)"
			41.9	1	"proteasome regulatory particle, lid subcomplex (sensu Eukaryota)/8541/(10)"
C3	28	11%	MRP21|MRPS9|MRPS5|MRP10|EHD3|RSM10|RSM24|MRPS28|MRP1|RSM18|RSM23|MRP13|MRPS35|RSM27|MRP4|FYV4|RSM25|RSM26|RSM7|RSM22|MRPS8|MRPS17|NAM9|MRPS18|RSM19|PET123|MRPS16|MRP51|		
			109.98	0.92	mitochondrial small ribosomal subunit/5763/(34)
C4	27	93%	UTP20|PWP2|NOP14|UTP4|UTP5|UTP6|POL5|UTP7|UTP8|YGR210C|IMP3|UTP9|UTP18|UTP10|MPP10|UTP30|SOF1|DIP2|EMG1|UTP13|UTP21|UTP14|UTP15|ECM16|NAN1|BMS1|NOC4|		
			74.54	0.85	small nucleolar ribonucleoprotein complex/5732/(56)
C5	25	40%	ROX3|MED8|SRB6|SRB8|MED2|SSB1|SRB7|SSN2|SRB4|PGD1|SOH1|NUT1|SRB5|SRB2|MED6|RGR1|MED11|SIN4|CSE2|GAL11|MED7|MED4|SSN3|MED1|NUT2|		
			87.31	0.95	mediator complex/119/(20)
			30.63	1	Srb-mediator complex/16592/(7)
			10.57	0.6	transcription factor complex/5667/(5)
C6	25	44%	SNF5|HTL1|RSC6|RSC3|RIM15|SWP82|RSC8|DBP3|RSC1|RTT102|SNF6|RSC30|STH1|SWI3|RSC4|RSC58|SFH1|RSC2|RSC9|ARP9|NPL6|SNF12|SNF2|SWI1|ARP7|		
			40.34	0.83	SWI/SNF complex/16514/(12)
			30.12	0.72	chromatin remodeling complex/16585/(11)
			61.24	0.93	RSC complex/16586/(15)
C7	24	46%	SPT7|TAF5|SGF29|TAF12|TAF10|NGG1|SPT3|ADA2|SGF73|TAF6|GCN5|TAF1|ARN2|TRA1|SPT8|TAF11|TAF13|TAF4|UBP8|TAF7|TAF9|SPT20|TAF3|HFI1|		
			73.53	0.85	SAGA complex/124/(20)
			59.99	0.87	SLIK (SAGA-like) complex/46695/(16)
			42.65	0.73	transcription factor TFIID complex/5669/(15)
			15.8	0.8	Ada2/Gcn5/Ada3 transcription activator complex/5671/(5)
C8	24	4%	MRPL16|MRPL36|IMG1|IMG2|MRPL1|MRPL7|MHR1|MRPL35|MRP20|MRPL28|MRPL9|MRPL8|MRP49|MRPL13|MRPL20|MRPL15|MRPL4|YML6|MRPL3|MRPL24|MRP7|MRPL17|MRPL10|MRPL40|		
			88.43	0.95	mitochondrial large ribosomal subunit/5762/(44)
C9	22	27%	SWC3|SWC5|NHP10|RVB1|SWR1|VPS72|IES5|IES1|ACT1|INO80|SWC4|YNG2|ARP4|IES3|ARP6|SWC7|BDF1|VPS71|ARP5|YAF9|ARP8|RVB2|		
			43.74	0.9	INO80 complex/31011/(11)
			15.25	0.38	H4/H2A histone acetyltransferase complex/43189/(13)
			61.32	1	SWR1 complex/812/(13)
C10	20	60%	NUP60|RRP43|RRP42|RRP45|RRP46|MTR3|SKI6|PCT1|RRP4|LRP1|NUP2|SRP1|CSL4|YNR024W|DIS3|RRP40|RRP6|SKI7|NUP1|ULP1|		
			57.8	1	nuclear exosome (RNase complex)/176/(12)
			47.38	1	cytoplasmic exosome (RNase complex)/177/(10)
C11	20	75%	PTA1|BUD14|REF2|CFT1|GLC7|YPI1|PTI1|FIP1|SWD2|MPE1|SDS22|PAP1|CFT2|YSH1|GLC8|SSU72|PFS2|SYC1|GIP3|YTH1|		
			74.35	1	mRNA cleavage and polyadenylation specificity factor complex/5847/(15)
			13.39	0.44	mRNA cleavage factor complex/5849/(9)
C12	20	85%	AAR2|PRP6|SPP381|PRP9|PRP11|SNU23|PRP3|BRR2|SMX2|PRP38|PRP31|PRP8|PRP21|LSM8|SNU114|RSE1|SNU66|LEA1|DIB1|PRP4|		
			48.78	0.7	U4/U6 x U5 tri-snRNP complex/46540/(32)
			18.71	0.37	snRNP U5/5682/(16)
			15.34	0.35	snRNP U2/5686/(14)
C13	18	72%	FLC2|PRE7|GRH1|PRE1|PUP3|PRE4|SCL1|PRE9|PUP2|PRE3|OSM1|RED1|PRE8|PRE5|PRE6|PUP1|PRE10|PRE2|		
			33.34	1	"proteasome core complex, alpha-subunit complex (sensu Eukaryota)/19773/(7)"
			33.34	1	"proteasome core complex, beta-subunit complex (sensu Eukaryota)/19774/(7)"
C14	18	72%	MUD1|LUC7|PRP42|SNU56|SMB1|SNU71|SMD1|NAM8|SNP1|PRP40|SMD3|SMD2|YHC1|PRP39|STO1|SME1|BRR1|SMX3|		
			32.22	0.66	commitment complex/243/(12)
			14.8	0.33	U4/U6 x U5 tri-snRNP complex/46540/(32)
			19.44	0.37	snRNP U5/5682/(16)
			78.63	0.88	snRNP U1/5685/(18)
C15	16	19%	SUP45|PAF1|RRP7|SUP35|CHD1|CKB1|RTF1|CKA1|YLR407W|CDC73|HOT1|TOP1|CTR9|CKB2|CKA2|LEO1|		
			21.11	0.71	Cdc73/Paf1 complex/16593/(7)
			19.16	1	protein kinase CK2 complex/5956/(4)
			19.06	0.37	transcription elongation factor complex/8023/(19)
C16	16	69%	PRP45|ECM2|BUD31|CWC2|CDC40|SYF1|CWC23|SYF2|CWC22|YJU2|PRP19|CLF1|SPP382|CEF1|PRP46|SNT309|		
			42.85	0.75	spliceosome complex/5681/(32)
C17	15	20%	HHF1|YDL156W|HAT2|SPT16|ASF1|HIF1|POB3|PDS5|YKU80|YKU70|HHF2|HHT2|RTT106|PSH1|HAT1|		
C18	15	47%	COP1|SEC26|GLO3|RET2|SEC27|SSF1|PCL7|SEC28|SEC21|PHO80|RET3|PHO85|YPL088W|PCL8|RRP15|		
			40.32	1	COPI vesicle coat/30126/(8)
C19	14	64%	FUN12|RPS8A|RPG1|RLI1|TIF35|RPS8B|HCR1|TIF34|NIP1|SUI1|DED1|PRT1|YPL105C|TIF5|		
			20.91	0.62	multi-eIF complex/43614/(8)
			28.24	0.85	eukaryotic translation initiation factor 3 complex/5852/(7)
C20	13	77%	MAK5|PUF6|CIC1|DRS1|NOC3|PWP1|FPR4|ERB1|RRP5|HAS1|BRX1|NOC2|YTM1|		
C21	13	38%	LSM2|PAT1|PEX19|DHH1|LSM6|LSM4|LSM5|KEM1|LSM1|PXA2|LSM3|PRP24|LSM7|		
			17.12	0.46	U4/U6 x U5 tri-snRNP complex/46540/(32)
			32.68	0.77	snRNP U6/5688/(9)
			13.63	0.46	small nucleolar ribonucleoprotein complex/5732/(56)
			14.78	0.4	cytoplasmic mRNA processing body/932/(10)
C22	12	0%	DEP1|RXT2|RXT3|UME6|SDS3|RCO1|SAP30|PHO23|RPD3|SIN3|UME1|CTI6|		
			25.41	0.58	histone deacetylase complex/118/(21)
C23	12	58%	CDC27|APC4|SWM1|CDC26|DOC1|CDC23|MND2|CDC16|APC9|APC2|APC1|APC5|		
			62.04	1	anaphase-promoting complex/5680/(16)
C24	12	17%	IML3|AME1|CHL4|MCM21|NKP1|PRP18|OKP1|MCM22|NKP2|CTF3|CTF19|MCM16|		
			17.15	0.5	condensed nuclear chromosome kinetochore/778/(35)
			20.47	1	COMA complex/817/(4)
C25	12	58%	TRS20|POL4|GSG1|TRS23|TRS120|TRS31|KRE11|GYP6|BET3|BET5|TRS130|TRS33|		
			55.32	1	TRAPP complex/30008/(10)
C26	11	73%	GCD6|GCD11|GCD2|YVH1|SUI2|GCN3|CDC123|GCD7|PET111|GCD1|SUI3|		
			23.35	0.71	guanyl-nucleotide exchange factor complex/32045/(7)
			11.48	0.37	multi-eIF complex/43614/(8)
			15.49	1	eukaryotic translation initiation factor 2 complex/5850/(3)
			26.39	1	eukaryotic translation initiation factor 2B complex/5851/(5)
C27	10	20%	CDC9|GDH2|CDC34|MRF1|RTG2|RTT107|RTT101|GRR1|MMS22|CRT10|		
C28	10	20%	GBP2|SUB2|HPR1|YRA1|HXK2|THP2|MFT1|HRB1|RLR1|TEX1|		
			15.81	1	transcription export complex/346/(3)
			21.32	1	THO complex/347/(4)
C29	10	10%	SIF2|SNT1|TRM3|AVT2|HOS2|YRF1-3|NCP1|HOS4|SET3|YMR155W|		
			17.11	0.5	histone deacetylase complex/118/(21)
C30	10	90%	NUG1|NOP7|MRT4|EBP2|RPF2|RLP7|NOP2|NOP15|NOG1|TIF6|		
C31	10	40%	PGI1|PGK1|TDH3|ENO1|ENO2|FBA1|GPM1|PDC1|ADH2|ADH1|		
			10.42	1	phosphopyruvate hydratase complex/15/(2)
C32	10	20%	SLA1|PEP1|RVS167|BZZ1|SQT1|GAL2|VRP1|YHM2|YSN1|LAS17|		
			13.68	0.5	actin cortical patch/30479/(40)
C33	10	20%	MYO4|CMD1|SHE3|MLC1|MYO3|SHE2|CRN1|MYO5|SHE4|MYO2|		
			10.42	1	myosin V complex/31475/(2)
			10.42	1	filamentous actin/31941/(2)
C34	9	67%	RFA1|MEC1|RAD51|RTT105|RIM101|DNA2|RFA3|RAD52|RFA2|		
			16.16	1	DNA replication factor A complex/5662/(3)
			14.78	0.75	"chromosome, telomeric region/781/(4)"
C35	9	0%	TFP1|VMA8|VMA7|VMA10|RAV1|VMA5|VPH2|VMA4|VMA13|		
			38.05	0.87	hydrogen-transporting ATPase V1 domain/221/(8)
C36	9	0%	MNN10|ANP1|PHB1|PHB2|MNN11|HOC1|FKS1|VAN1|MNN9|		
			33.76	1	"alpha-1,6-mannosyltransferase complex/136/(6)"
C37	8	0%	TPD3|PPH21|PPH22|CDC55|RTS3|ZDS2|ZDS1|PHR1|		
			26.71	0.83	protein phosphatase type 2A complex/159/(6)
C38	8	88%	GLE2|NIC96|NUP49|NUP57|NUP159|NSP1|NUP82|NUP116|		
			28.99	1	nuclear pore/5643/(50)
C39	8	13%	HTB1|KAP114|NAP1|CKI1|RIM11|NIS1|HTZ1|YOL070C|		
C40	8	38%	COG3|COG7|COG1|COG2|COG8|COG6|COG5|COG4|		
			49.09	1	Golgi transport complex/17119/(8)
C41	8	88%	DAD3|DAD1|SPC19|DUO1|DAM1|ASK1|SPC34|DAD2|		
			45.28	1	DASH complex/42729/(10)
			37.35	1	spindle/5819/(20)
			11.48	0.37	spindle microtubule/5876/(11)
			25.92	0.87	condensed nuclear chromosome kinetochore/778/(35)
C42	8	100%	EXO84|SEC5|SEC3|SEC15|SEC6|EXO70|SEC10|SEC8|		
			11.28	0.5	incipient bud site/131/(38)
			49.09	1	exocyst/145/(8)
			18.58	0.75	bud tip/5934/(48)
C43	8	25%	CKS1|CDC28|CLB3|SIC1|CLB4|CLN1|CLN2|CLB2|		
C44	8	100%	SPB1|DBP10|RRP1|SPB4|NSA1|RPF1|RIX7|NIP7|		
C45	7	29%	CDC53|RRI1|CSN9|PCI8|CSI1|RRI2|HRT1|		
			27.69	0.83	signalosome complex/8180/(6)
C46	7	86%	ARC40|ARP2|ARC15|ARP3|ARC19|ARC18|ARC35|		
			43.71	1	Arp2/3 protein complex/5885/(7)
C47	7	43%	CDC10|SHS1|CDC12|CDC11|BUD4|CDC3|REV3|		
			18.29	0.57	bud neck septin ring/144/(9)
C48	7	71%	RFC5|RFC2|CTF18|RFC3|RFC4|ELG1|RFC1|		
			38.92	1	DNA replication factor C complex/5663/(10)
C49	7	29%	VID21|EAF5|EPL1|EAF6|EAF7|ESA1|EAF3|		
			21.48	0.71	histone acetyltransferase complex/123/(15)
			36.26	1	H4/H2A histone acetyltransferase complex/43189/(13)
C50	7	0%	GIM4|BUD27|PAC10|PFD1|YKE2|GIM5|GIM3|		
			36.25	1	prefoldin complex/16272/(6)
C51	7	0%	VID24|GID7|RMD5|VID30|VID28|FYV10|GID8|		
C52	7	0%	SWD1|SWD3|SHG1|SDC1|SET1|BRE2|SPP1|		
			41.63	1	COMPASS complex/48188/(8)
C53	7	100%	KIN28|TFB1|TFB3|RAD3|SSL1|CCL1|TFB4|		
			19.56	0.57	nucleotide-excision repair factor 3 complex/112/(7)
			38.92	1	transcription factor TFIIH complex/5675/(10)
C54	7	86%	LHP1|SRP14|SRP21|SEC65|SRP72|SRP68|SRP54|		
			34.3	0.85	signal recognition particle (sensu Eukaryota)/5786/(7)
C55	7	100%	POP5|POP8|POP7|POP4|RPP1|POP1|POP3|		
			37.91	1	ribonuclease MRP complex/172/(11)
			38.92	1	nucleolar ribonuclease P complex/5655/(10)
C56	7	14%	SIT4|SAP155|SAP185|SAP190|YLR179C|TAP42|HRK1|		
C57	7	57%	RPL4A|BFR2|LCP5|ENP2|RPL8B|KRE33|RPL20B|		
C58	7	57%	NUP84|NUP145|SEH1|NUP85|NUP120|SEC13|MEX67|		
			25.29	1	nuclear pore/5643/(50)
C59	7	14%	CCR4|CDC39|BTT1|CAF130|YJR011C|CAF40|POP2|		
			13.49	0.42	CCR4-NOT core complex/30015/(7)
C60	7	0%	VPS8|VAM6|VPS41|PEP3|VPS33|PEP5|VPS16|		
			36.25	1	HOPS complex/30897/(6)
C61	7	14%	ISW1|IOC3|VPS1|IOC2|IOC4|ESC8|MOT1|		
			23.12	1	ISW1 complex/16587/(4)
C62	7	29%	YPT10|YPT31|SEC4|YPT1|YPT32|YPT7|DSS4|		
C63	7	29%	LYS21|RPL4B|RPL10|RPS1B|RPS3|RPS6A|YAR1|		
C64	6	17%	PSY4|PPH3|SSD1|RRD1|SPT5|PSY2|		
C65	6	100%	ORC2|ORC6|ORC3|ORC1|ORC5|ORC4|		
			29.68	1	pre-replicative complex/5656/(15)
			38.19	1	nuclear origin of replication recognition complex/5664/(6)
C66	6	0%	ELP2|IKI1|IKI3|ELP6|ELP3|ELP4|		
			27.99	1	transcription elongation factor complex/8023/(19)
C67	6	100%	TFC3|TFC1|TFC6|TFC4|TFC7|TFC8|		
			38.19	1	transcription factor TFIIIC complex/127/(6)
C68	6	0%	YFL034W|APM2|APL2|APS1|APM1|APL4|		
			30.73	1	AP-1 adaptor complex/30121/(5)
C69	6	0%	NAT1|YGR054W|ARD1|RPS4B|RPS4A|NAT5|		
			17.6	1	NatA complex/31415/(3)
C70	6	0%	SHP1|NPL4|TDP1|YDR049W|OTU1|UBX4|		
C71	6	0%	KTI11|EFT2|HGH1|DPH1|DPH2|EFT1|		
C72	6	83%	USE1|TIP20|SEC22|SEC39|DSL1|UFE1|		
C73	6	0%	VPS29|PEP8|VPS35|VPS5|VPS17|MUK1|		
			30.73	1	retromer complex/30904/(5)
			16.29	0.83	endosome/5768/(49)
C74	6	100%	CCT4|CCT6|TCP1|CCT2|CCT3|CCT5|		
			32.06	1	chaperonin-containing T-complex/5832/(11)
C75	6	17%	TIF4631|TIF2|EAP1|TIF1|CDC33|CAF20|		
			22.35	0.8	eukaryotic translation initiation factor 4F complex/16281/(5)
			14.54	0.66	ribosome/5840/(25)
			10.43	0.66	mRNA cap complex/5845/(3)
C76	5	0%	SNF1|YEL023C|GAL83|SNF4|SIP2|		
C77	5	40%	YRB1|SRM1|MOG1|GSP1|GSP2|		
C78	5	100%	RSA4|SDA1|IPI1|RIX1|IPI3|		
C79	5	100%	MCD1|SMC1|IRR1|SMC3|CDC5|		
			22.35	0.8	nuclear cohesin complex/798/(6)
C80	5	20%	RSP5|YHR131C|ALY2|BUL1|UBP2|		
C81	5	0%	OCA4|OCA5|SIW14|OCA2|OCA1|		
C82	5	0%	PKH1|PIL1|MRP8|YMR086W|LSP1|		
C83	5	60%	GLK1|SIK1|BUD21|NOP58|RRP9|		
			12.27	0.8	small nucleolar ribonucleoprotein complex/5732/(56)
C84	5	40%	KAP104|KTR3|NAB2|YNL035C|HRP1|		
C85	5	80%	PSF1|SLD5|PSF2|PSF3|CTF4|		
			25.06	1	GINS complex/811/(4)
C86	5	100%	BRN1|YCG1|SMC2|SMC4|YCS4|		
			32.52	1	nuclear condensin complex/799/(5)
C87	5	0%	STE7|DIG2|KSS1|STE12|DIG1|		
C88	5	20%	IPP1|ACK1|ROM2|TUS1|YPL066W|		
C89	5	80%	ENP1|TSR1|LTV1|RIO2|HRR25|		
			10.83	0.66	"nucleolar preribosome, small subunit precursor/30688/(3)"
C90	4	25%	HIR1|HPC2|HIR2|RAD53|		
			17.82	0.75	HIR complex/417/(4)
C91	4	25%	AIR2|AIR1|PAP2|NOP53|		
			17.82	0.75	TRAMP complex/31499/(4)
C92	4	75%	SPC72|SPC97|TUB4|SPC98|		
			17.82	0.75	inner plaque of spindle pole body/5822/(4)
			23.96	1	outer plaque of spindle pole body/5824/(6)
			19.21	1	gamma-tubulin complex (sensu Saccharomyces)/928/(3)
C93	4	100%	SPC25|TID3|SPC24|NUF2|		
			26.67	1	Ndc80 complex/31262/(4)
			15.81	1	condensed nuclear chromosome kinetochore/778/(35)
C94	4	25%	RNR1|RNR4|RNR2|WTM1|		
			17.82	0.75	ribonucleoside-diphosphate reductase complex/5971/(4)
C95	4	0%	MSH3|MSH6|MPH1|MSH2|		
C96	4	25%	BUD32|GON7|KAE1|CGI121|		
			25.06	1	EKC/KEOPS protein complex/408/(5)
C97	4	0%	BCY1|TPK1|TPK3|TPK2|		
			19.21	1	cAMP-dependent protein kinase complex/5952/(3)
C98	4	0%	TPS1|TPS2|TSL1|TPS3|		
			26.67	1	"alpha,alpha-trehalose-phosphate synthase complex (UDP-forming)/5946/(4)"
C99	4	0%	APM3|APL6|APS3|APL5|		
			26.67	1	AP-3 adaptor complex/30123/(4)
C100	4	0%	YER071C|CAP2|YIR003W|CAP1|		
			12.44	1	F-actin capping protein complex/8290/(2)
C101	4	100%	MTW1|DSN1|NNF1|NSL1|		
			12.18	0.75	kinetochore/776/(20)
			26.67	1	MIND complex/818/(4)
			25.06	1	spindle pole/922/(5)
C102	4	0%	UBC13|MMS2|ARO9|SIP5|		
			12.44	1	ubiquitin conjugating enzyme complex/31371/(2)
C103	4	0%	RVS161|PHO84|GYL1|GYP5|		
C104	4	50%	PBY1|EDC3|DCP2|DCP1|		
			14.42	0.75	cytoplasmic mRNA processing body/932/(10)
C105	4	0%	CIS1|ATG17|ATG29|ATG11|		
C106	4	100%	POL12|PRI1|PRI2|POL1|		
			23.12	1	alpha DNA polymerase:primase complex/5658/(7)
C107	4	25%	KRR1|YGR017W|NSR1|VIP1|		
C108	4	0%	ERP2|ERP1|EMP24|ERV25|		
			18.41	1	ER to Golgi transport vesicle/30134/(19)
C109	4	0%	SKI8|YKL023W|SKI2|SKI3|		
C110	4	75%	CIA1|YHR122W|MET18|NAR1|		
C111	4	75%	SEN1|NAB6|NRD1|NAB3|		
C112	4	50%	SEC66|SEC72|SEC63|SEC62|		
			26.67	1	Sec62/Sec63 complex/31207/(4)
C113	4	50%	GDI1|YPT52|VPS21|MRS6|		
C114	4	0%	CYK3|BMH2|BMH1|YFR017C|		
C115	4	100%	PCF11|RNA15|RNA14|CLP1|		
			21.84	1	mRNA cleavage factor complex/5849/(9)
C116	4	0%	STP22|YGR206W|SRN2|VPS28|		
			14.41	1	endosome/5768/(49)
			19.21	1	ESCRT I complex/813/(3)
C117	4	0%	REG1|SIP1|PRK1|SOL1|		
C118	4	25%	EFB1|TEF4|CAM1|TEF1|		
			11.48	0.75	ribosome/5840/(25)
			17.82	0.75	eukaryotic translation elongation factor 1 complex/5853/(4)
C119	4	50%	DPB3|DPB4|POL2|DPB2|		
			13.32	0.75	replication fork/5657/(14)
			26.67	1	epsilon DNA polymerase complex/8622/(4)
C120	4	50%	SFB3|SFB2|SEC16|SEC23|		
			22.42	1	COPII vesicle coat/30127/(8)
C121	4	0%	SIR2|SIR4|BLM10|SIR3|		
			16.91	0.75	chromatin silencing complex/5677/(5)
			15.66	0.75	nuclear telomeric heterochromatin/5724/(7)
			14.11	0.75	nuclear telomere cap complex/783/(11)
C122	4	0%	RAD16|RAD4|CCE1|RAD14|		
			10.65	0.5	repairosome/108/(4)
C123	4	0%	PPT1|HSC82|STI1|HSP82|		
C124	4	75%	PPN1|TFA1|TFA2|DBP2|		
			12.44	1	transcription factor TFIIE complex/5673/(2)
C125	4	0%	KGD2|LPD1|YMR31|KGD1|		
			19.21	1	oxoglutarate dehydrogenase complex (sensu Eukaryota)/9353/(3)
C126	4	0%	SSK22|HEX3|SSK1|SSK2|		
C127	4	0%	YBR137W|MDY2|SGT2|YOR164C|		
C128	4	0%	HTB2|HTA2|HHT1|ABF2|		
			13.82	0.75	nuclear nucleosome/788/(12)
C129	4	0%	APL3|APS2|ALP1|APM4|		
			17.82	0.75	AP-2 adaptor complex/30122/(4)
C130	4	0%	RTN1|CRP1|IDS2|PEP4|		
C131	4	0%	VPS15|VPS34|VPS38|VPS30|		
C132	4	0%	YAL049C|YDL025C|YGR016W|YHR009C|		
C133	3	33%	YDR131C|SKP1|YMR258C|		
C134	3	0%	MSI1|CAC2|RLF2|		
			18.29	1	chromatin assembly complex/5678/(5)
C135	3	0%	ITC1|DLS1|ISW2|		
			19.21	1	chromatin accessibility complex/8623/(4)
C136	3	0%	PFK1|PFK2|RUP1|		
			13.13	1	6-phosphofructokinase complex/5945/(2)
C137	3	67%	ARC1|GUS1|MES1|		
			20.6	1	methionyl glutamyl tRNA synthetase complex/17102/(3)
C138	3	67%	HSH155|CUS2|HSH49|		
			14.7	1	snRNP U2/5686/(14)
C139	3	33%	PHO81|CYR1|SRV2|		
C140	3	0%	ZUO1|SSZ1|SCP160|		
C141	3	0%	RGP1|RIC1|YPT6|		
C142	3	33%	UBX7|UBX5|UFD1|		
C143	3	0%	HDA2|HDA1|HDA3|		
			13.4	1	histone deacetylase complex/118/(21)
C144	3	0%	GET3|GET2|GET1|		
			20.6	1	GET complex/43529/(3)
C145	3	0%	GSY1|GLG2|GSY2|		
C146	3	0%	BCH2|CHS5|BCH1|		
C147	3	0%	MAK31|MAK10|MAK3|		
			20.6	1	NatC complex/31417/(3)
C148	3	0%	MBP1|SWI4|SWI6|		
C149	3	33%	RTT103|RAI1|RAT1|		
C150	3	33%	CDC19|SSA2|SSE1|		
C151	3	33%	YGR071C|VID22|TBF1|		
C152	3	0%	CTK2|CTK1|CTK3|		
C153	3	33%	CDC15|YBR281C|VTH1|		
C154	3	0%	YAL027W|RAD10|RAD1|		
			12.03	0.66	nucleotide-excision repair factor 1 complex/110/(3)
C155	3	0%	CNB1|CNA1|CMP2|		
			19.21	1	calcineurin complex/5955/(4)
C156	3	67%	UTP22|DHR2|UBP10|		
C157	3	0%	YIL161W|FAP1|FPR1|		
C158	3	0%	PDB1|PDA1|LAT1|		
			18.29	1	pyruvate dehydrogenase complex (sensu Eukaryota)/5967/(5)
C159	3	0%	PTC3|PAA1|PTC2|		
C160	3	67%	CDC2|HYS2|POL32|		
			20.6	1	delta DNA polymerase complex/43625/(3)
C161	3	33%	YHB1|HSP60|MKT1|		
C162	3	33%	SEC31|YHL039W|NUP133|		
C163	3	0%	XRS2|MRE11|RAD50|		
			20.6	1	Mre11 complex/30870/(3)
C164	3	33%	NUC1|TRZ1|YMR099C|		
C165	3	0%	PSR1|PSR2|WHI2|		
C166	3	33%	YBR225W|MDS3|CDC25|		
C167	3	67%	RAM1|CDC43|RAM2|		
			13.13	1	CAAX-protein geranylgeranyltransferase complex/5953/(2)
			13.13	1	protein farnesyltransferase complex/5965/(2)
C168	3	0%	UMP1|ADD66|YLR199C|		
C169	3	0%	MLH2|MLH1|PMS1|		
C170	3	0%	LTE1|KEL2|KEL1|		
C171	3	0%	EDE1|SYP1|HTA1|		
C172	3	33%	ARX1|NSA2|ALB1|		
C173	3	33%	TAF2|TAF8|TAF14|		
			14.47	1	transcription factor TFIID complex/5669/(15)
C174	3	0%	VPS54|VPS52|VPS53|		
			19.21	1	GARP complex/938/(4)
C175	3	33%	BCP1|RPL23B|RKM1|		
C176	3	0%	VMA2|HSP42|RAV2|		
C177	3	0%	HAP3|HAP2|HAP5|		
			19.21	1	CCAAT-binding factor complex/16602/(4)
C178	3	33%	GCN20|GCN1|GFA1|		
C179	3	0%	YGR250C|SGN1|PUB1|		
C180	3	0%	SOL2|ADE16|ADE17|		
C181	3	0%	AMS1|LAP4|ATG19|		
C182	3	0%	SOD1|CCS1|IRA2|		
C183	3	0%	UBP14|ECM30|UBP15|		
C184	3	67%	RPS11A|ESF1|ESF2|		
C185	3	0%	ATP1|ATP2|ATP11|		
			13.13	1	"proton-transporting ATP synthase, catalytic core (sensu Eukaryota)/5754/(2)"
C186	3	33%	DBF2|MOB1|DBF20|		
C187	3	0%	IMD2|IMD3|IMD4|		
C188	3	0%	VPS25|VPS36|SNF8|		
			20.6	1	ESCRT II complex/814/(3)
C189	3	0%	PRX1|DYN2|PAC11|		
			11.34	0.66	cytoplasmic dynein complex/5868/(4)
C190	3	0%	RNH202|RNH203|RNH201|		
			20.6	1	ribonuclease H2 complex/32299/(3)
C191	3	0%	MEC3|SUV3|DDC1|		
C192	3	0%	AKR1|GPA1|STE4|		
			12.03	0.66	heterotrimeric G-protein complex/5834/(3)
C193	3	0%	PTC1|NBP2|PBS2|		
C194	3	0%	ABP1|SAC6|APP1|		
C195	3	67%	YGL242C|UGP1|IMP4|		
C196	3	100%	NSE4|NSE3|SMC5|		
			16.57	1	Smc5-Smc6 complex/30915/(8)
C197	3	33%	MMS21|SMC6|TSR2|		
C198	3	0%	VAB2|YGL079W|YNL086W|		
C199	3	0%	YJL144W|CLU1|YNL234W|		
C200	2	0%	COR1|QCR2|		
			10.42	1	respiratory chain complex III (sensu Eukaryota)/5750/(10)
C201	2	100%	UBA2|AOS1|		
C202	2	50%	CDC48|DOA1|		
C203	2	0%	SNX41|SNX4|		
C204	2	50%	PAB1|CBC2|		
C205	2	100%	MET30|MET4|		
C206	2	100%	PRP43|NTR2|		
C207	2	100%	MTR4|RNT1|		
C208	2	100%	FRS2|FRS1|		
			14.23	1	phenylalanine-tRNA ligase complex/9328/(2)
C209	2	0%	YJL070C|AMD1|		
C210	2	0%	BRE1|LGE1|		
C211	2	0%	ROT2|GTB1|		
C212	2	50%	ILV5|YPL225W|		
C213	2	0%	GIR2|RBG2|		
C214	2	0%	TRM8|TRM82|		
C215	2	50%	NAM2|CUS1|		
C216	2	0%	TMA20|TMA22|		
C217	2	100%	MAS2|MAS1|		
			14.23	1	mitochondrial processing peptidase complex/17087/(2)
C218	2	50%	TRR1|TRR2|		
C219	2	0%	LEU4|LEU9|		
C220	2	0%	MDM20|NAT3|		
			14.23	1	NatB complex/31416/(2)
C221	2	0%	SER3|SER33|		
C222	2	0%	NTH2|NTH1|		
C223	2	0%	RTS1|RRD2|		
C224	2	100%	CEG1|CET1|		
C225	2	0%	HSE1|VPS27|		
C226	2	0%	DCS1|DCS2|		
			10.42	1	cytoplasmic mRNA processing body/932/(10)
C227	2	0%	LSC2|LSC1|		
C228	2	0%	RPL7A|RPL7B|		
C229	2	0%	NPL3|TIF4632|		
C230	2	0%	HOG1|RCK2|		
C231	2	0%	TRP2|TRP3|		
			14.23	1	anthranilate synthase complex/5950/(2)
C232	2	100%	GCD14|GCD10|		
C233	2	0%	PAN2|PAN3|		
			14.23	1	PAN complex/31251/(2)
C234	2	0%	MET13|MET12|		
C235	2	0%	RTT109|VPS75|		
C236	2	0%	ATG18|ATG2|		
C237	2	0%	FBP26|YLR345W|		
C238	2	0%	VMA6|VPH1|		
			11.19	1	hydrogen-transporting ATPase V0 domain/220/(7)
C239	2	0%	RGD1|FYV8|		
C240	2	50%	TRM112|TRM11|		
C241	2	0%	YGR283C|YMR310C|		
C242	2	0%	ARF2|ARF1|		
			10.9	1	Golgi-associated vesicle/5798/(8)
C243	2	50%	KIC1|SOG2|		
C244	2	0%	CPA2|CPA1|		
			14.23	1	carbamoyl-phosphate synthase complex/5951/(2)
C245	2	0%	STE50|STE11|		
C246	2	50%	SPO12|PSE1|		
C247	2	50%	YDR415C|UBA1|		
C248	2	0%	KEX2|SIL1|		
C249	2	0%	PTC4|YDR186C|		
C250	2	0%	RAD18|RAD6|		
C251	2	100%	MAK21|RPL18A|		
C252	2	0%	YBR030W|MAP1|		
C253	2	50%	PTC7|SEC14|		
C254	2	0%	YDR357C|YKL061W|		
C255	2	0%	PTC6|PRO2|		
C256	2	0%	YKL215C|HSP104|		
C257	2	0%	AFG3|YTA12|		
			14.23	1	m-AAA complex/5745/(2)
C258	2	0%	ASN2|ASN1|		
C259	2	100%	TOA2|TOA1|		
			14.23	1	transcription factor TFIIA complex/5672/(2)
C260	2	100%	TUB2|TUB1|		
			13.13	1	tubulin complex/45298/(3)
			13.13	1	polar microtubule/5827/(3)
			11.52	1	kinetochore microtubule/5828/(6)
			11.52	1	nuclear microtubule/5880/(6)
C261	2	0%	UBP3|BRE5|		
C262	2	50%	MUD2|MSL5|		
C263	2	0%	SPE4|SPE3|		
C264	2	0%	EGD2|EGD1|		
			13.13	1	nascent polypeptide-associated complex/5854/(3)
C265	2	50%	BUR2|SGV1|		
C266	2	100%	SEC17|YKT6|		
C267	2	0%	CHC1|CLC1|		
			12.44	1	clathrin vesicle coat/30125/(4)
C268	2	0%	RTG3|RTG1|		
C269	2	0%	PEA2|SPA2|		
			11.93	1	polarisome/133/(5)
C270	2	0%	MAD1|MAD2|		
C271	2	0%	COX4|COX5A|		
C272	2	0%	ULA1|UBA3|		
C273	2	50%	PDI1|MNL1|		
C274	2	0%	FMC1|ATP12|		
C275	2	0%	SAS3|NTO1|		
C276	2	100%	DRE2|TAH18|		
C277	2	50%	QNS1|CPR6|		
C278	2	0%	YCK1|YCK2|		
C279	2	0%	CYC8|TUP1|		
C280	2	0%	NMA2|NMA1|		
C281	2	50%	BUD20|NOG2|		
C282	2	50%	DOP1|YNL297C|		
C283	2	100%	MOB2|CBK1|		
C284	2	100%	RRN6|RRN7|		
			11.93	1	RNA polymerase I transcription factor complex/120/(5)
C285	2	50%	BFA1|TEM1|		
C286	2	0%	TAH1|PIH1|		
C287	2	0%	CSM3|TOF1|		
C288	2	0%	UBC4|UFD4|		
C289	2	0%	SAF1|AAH1|		
C290	2	100%	NFS1|ISD11|		
C291	2	0%	RAS2|RAS1|		
C292	2	0%	NTC20|ISY1|		
C293	2	0%	CCZ1|YGL124C|		
C294	2	0%	UBP13|YOL087C|		
C295	2	0%	MRPL37|MRPL19|		
C296	2	0%	DCC1|CTF8|		
			10.42	1	DNA replication factor C complex/5663/(10)
C297	2	0%	VAC14|FIG4|		
C298	2	0%	YGR043C|TAL1|		
C299	2	100%	SRP101|SRP102|		
			14.23	1	signal recognition particle receptor complex/5785/(2)
C300	2	0%	YGR130C|YMR031C|		
C301	2	50%	RSA3|NOP8|		
C302	2	50%	YLR287C|RPP0|		
C303	2	0%	RAD7|ELC1|		
			12.44	1	nucleotide-excision repair factor 4 complex/113/(4)
C304	2	0%	NUP170|NUP53|		
C305	2	0%	SOY1|URN1|		
C306	2	0%	IDH1|IDH2|		
C307	2	0%	HIS4|FMP48|		
C308	2	0%	ALD3|ALD2|		
C309	2	0%	BUD6|YOR304C-A|		
C310	2	50%	PAN1|END3|		
C311	2	0%	LSB3|SLA2|		
C312	2	50%	SSL2|YOR352W|		
C313	2	0%	SUM1|RFM1|		
C314	2	0%	GRX3|GRX4|		
C315	2	0%	BAT2|YBT1|		
C316	2	50%	GEA2|CRM1|		
C317	2	50%	POL30|RAD27|		
C318	2	100%	URB2|URB1|		
C319	2	0%	KCC4|GIN4|		
C320	2	50%	VTI1|PEP12|		
C321	2	100%	WBP1|SWP1|		
			10.65	1	oligosaccharyl transferase complex/8250/(9)
C322	2	0%	GDH3|GDH1|		
C323	2	50%	MYO1|MLC2|		
C324	2	0%	SNF7|VPS4|		
C325	2	100%	FAS1|FAS2|		
			14.23	1	fatty acid synthase complex/5835/(2)
C326	2	50%	KCS1|PIK1|		
C327	2	100%	STT3|OST1|		
			10.65	1	oligosaccharyl transferase complex/8250/(9)
C328	2	0%	ECM1|SXM1|		
C329	2	0%	ATP5|ATP7|		
			13.13	1	"proton-transporting ATP synthase, stator stalk (sensu Eukaryota)/274/(3)"
C330	2	50%	PNO1|DIM1|		
C331	2	100%	CCT7|PLP2|		
C332	2	0%	MTF2|SLS1|		
C333	2	0%	ERV46|ERV41|		
C334	2	50%	REI1|NMD3|		
C335	2	0%	RBG1|TMA46|		
C336	2	0%	SCS2|OPI1|		
C337	2	100%	CBF5|KRI1|		
C338	2	0%	PSK1|PSK2|		
C339	2	50%	PET130|RPM2|		
C340	2	50%	SSC1|MSS51|		
C341	2	100%	SPT6|SPN1|		
C342	2	50%	MPM1|TOM40|		
C343	2	50%	CDC36|NOT5|		
			11.19	1	CCR4-NOT core complex/30015/(7)
C344	2	0%	PPZ2|VHS3|		
C345	2	50%	YBL036C|YNL247W|		
C346	2	0%	TRM7|YPL183C|		
C347	2	0%	SAM2|SAM1|		
C348	2	0%	TRX2|FMP34|		
C349	2	50%	RPP2B|RPL25|		
C350	2	0%	PMC1|GRE3|		
C351	2	0%	YDR051C|UBR2|		
C352	2	0%	CBS1|PIR1|		
C353	2	0%	PIB1|EMP70|		
C354	2	0%	MCH1|SFG1|		
C355	2	0%	YER140W|SLP1|		
C356	2	100%	BET4|BET2|		
			14.23	1	Rab-protein geranylgeranyltransferase complex/5968/(2)
C357	2	0%	HMT1|PSP2|		
C358	2	50%	YKL088W|PPZ1|		
C359	2	0%	ERP3|ERP5|		
C360	2	0%	BUD13|IST3|		
C361	2	0%	RFS1|PST2|		
C362	2	0%	DMA1|SKP2|		
C363	2	0%	PET122|YNL274C|		
C364	2	0%	YPK1|YPK2|		
C365	2	50%	PCM1|MGS1|		
C366	2	50%	CDC24|BOI2|		
C367	2	100%	LCB2|LCB1|		
			14.23	1	serine C-palmitoyltransferase complex/17059/(2)
C368	2	100%	RCL1|MRD1|		
C369	2	50%	RPL31A|RPL28|		
C370	2	0%	SAS5|WTM2|		
C371	2	0%	SWH1|OSH2|
C372	2	0%	OYE2|OYE3|
C373	2	0%	FUN19|KAP123|
C374	2	50%	YDR532C|SPC105|
C375	2	0%	GLN3|URE2|
C376	2	50%	THS1|YKR018C|
C377	2	50%	APC11|SAP1|
C378	2	0%	GIP1|YGL057C|
C379	2	100%	SLY1|SED5|
C380	2	0%	BDH1|YAL061W|
C381	2	0%	SAC3|THP1|
C382	2	0%	COX20|CHS1|
C383	2	0%	YGR093W|DBR1|
C384	2	0%	YEF1|UTR1|
C385	2	0%	UGA1|NDT80|
C386	2	0%	SNZ3|SNZ1|
C387	2	0%	YOR251C|REV1|
C388	2	0%	BUB1|BUB3|
C389	2	0%	INP52|BSP1|
C390	2	0%	URA5|URA10|
C391	1	100%	KAP95|
C392	1	100%	NOP1|
C393	1	100%	RPL3|
C394	1	100%	YEF3|
C395	1	0%	MAM33|
C396	1	0%	SGF11|
C397	1	0%	YAP1|
C398	1	0%	MRPL23|
```
