# Supplementary figures and images for: A high-accuracy consensus map of yeast protein complexes reveals modular nature of gene essentiality
Source: BMC Bioinformatics. 2007 Jul 2;8:236. doi: 10.1186/1471-2105-8-236 (PMC1940025; doi:10.1186/1471-2105-8-236)

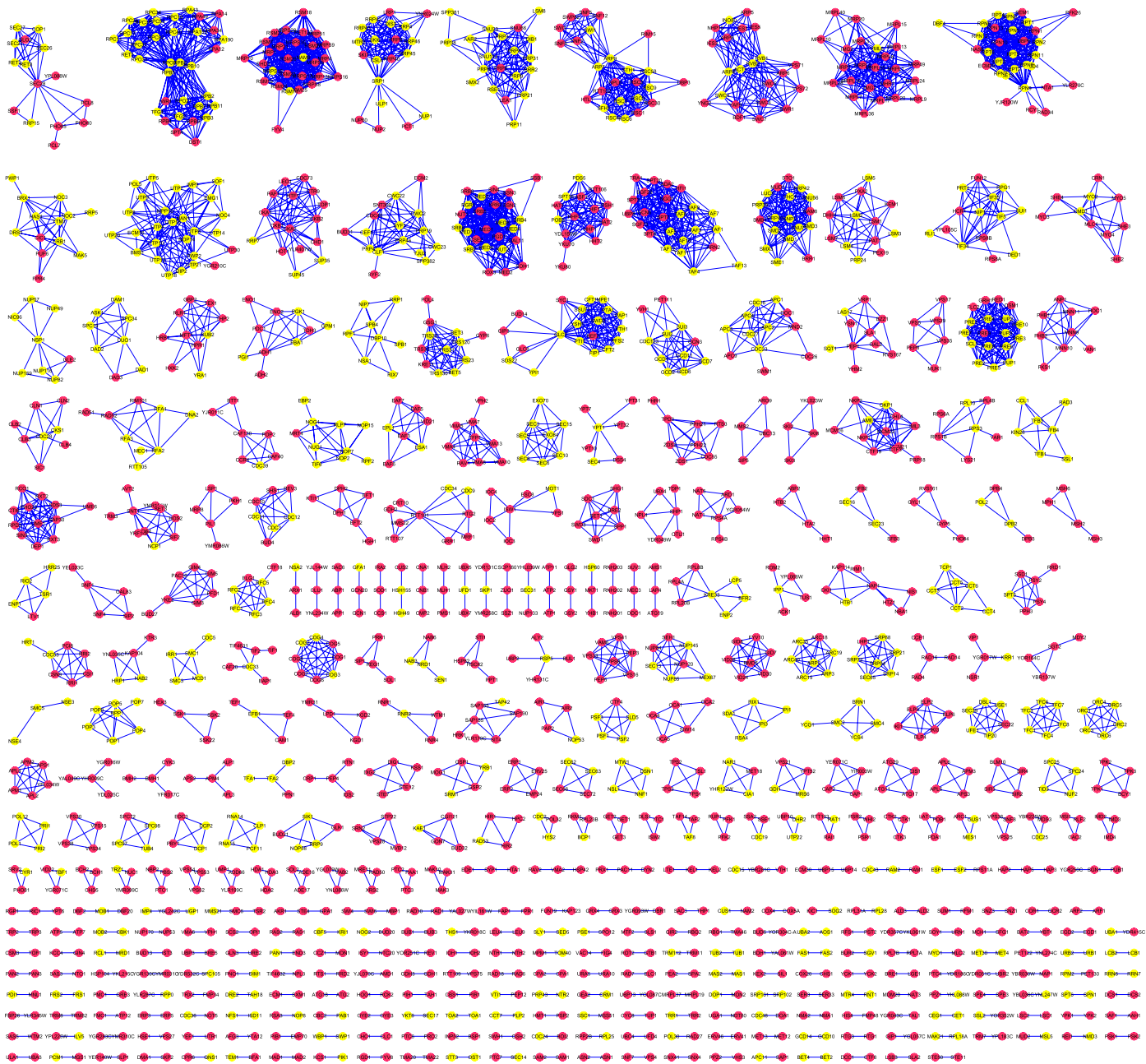

Supplement: Additional File 4 — A subset of the E-2 complex map. After applying the E = 10-2 threshold to the PICO interaction set, the subset of 5,352 interactions was clustered with MCL, using parameters that maximized correlation with a filtered set of GO component annotations. Interactions within clusters (4,411) were plotted with Cytoscape using the included "organic" layout algorithm. Interactions between clusters (941) were omitted for clarity. Yellow nodes indicate essential proteins; red, nonessential. [file 1471-2105-8-236-S4.pdf]

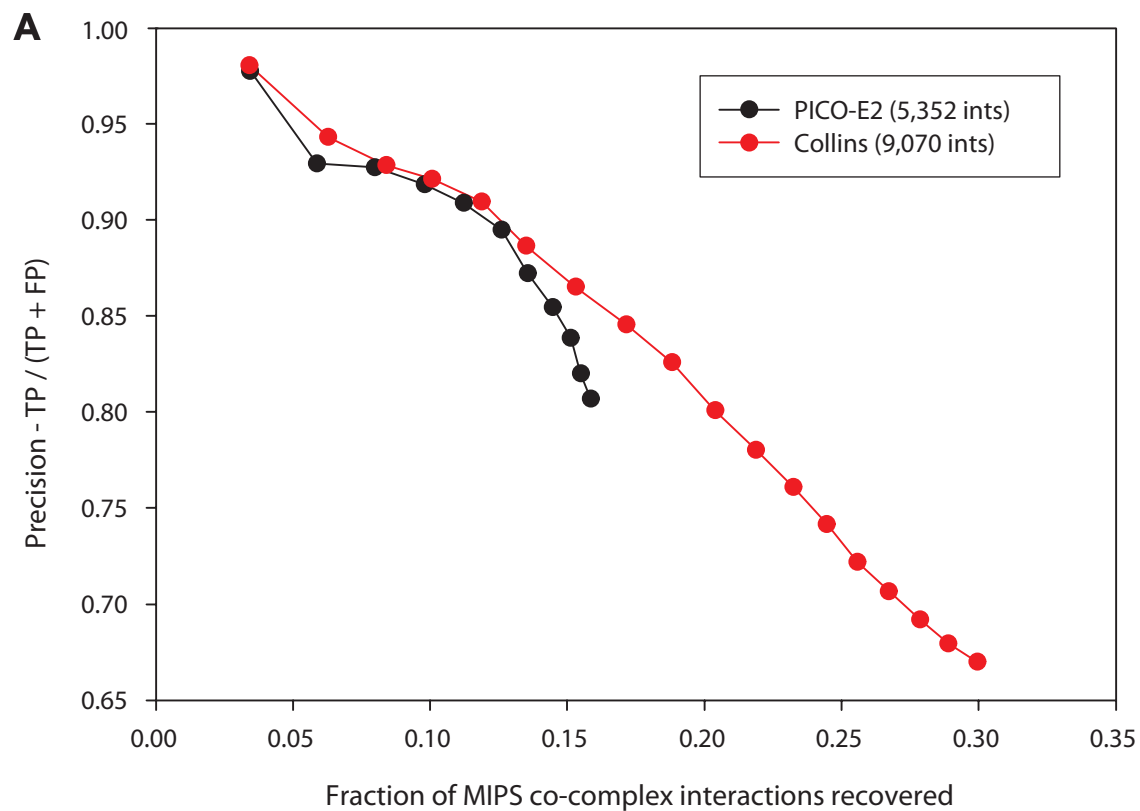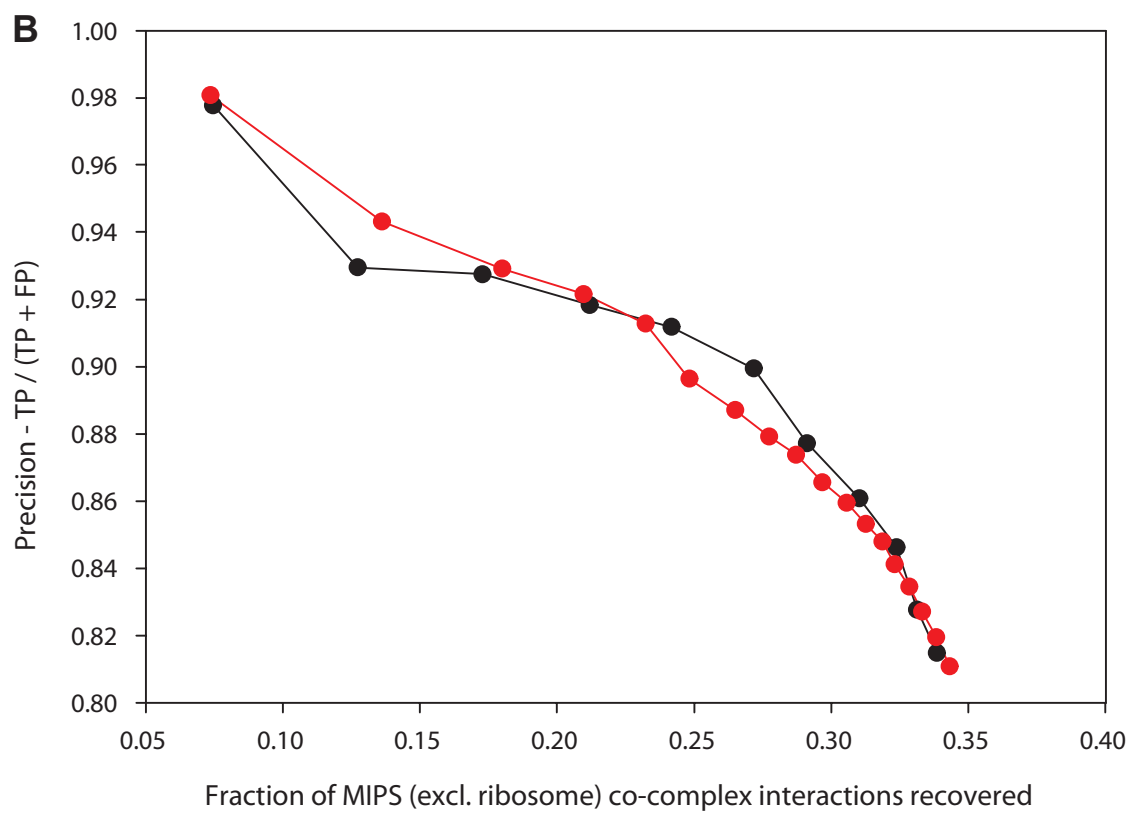

Supplement: Additional File 5 [file 1471-2105-8-236-S5.pdf]
